# Mobile elements drive recombination hotspots in the core genome of *Staphylococcus aureus*

**DOI:** 10.1038/ncomms4956

**Published:** 2014-05-23

**Authors:** Richard G. Everitt, Xavier Didelot, Elizabeth M. Batty, Ruth R Miller, Kyle Knox, Bernadette C. Young, Rory Bowden, Adam Auton, Antonina Votintseva, Hanna Larner-Svensson, Jane Charlesworth, Tanya Golubchik, Camilla L. C. Ip, Heather Godwin, Rowena Fung, Tim E. A. Peto, A. Sarah Walker, Derrick W. Crook, Daniel J. Wilson

**Affiliations:** 1 Nuffield Department of Medicine, University of Oxford, John Radcliffe Hospital, Oxford OX3 9DU, UK; 2 Department of Statistics, University of Oxford, 1 South Parks Road, Oxford OX1 3TG, UK; 3 Wellcome Trust Centre for Human Genetics, Roosevelt Drive, Oxford OX3 7BN, UK; 4 Department of Primary Care Health Sciences, University of Oxford, 23-38 Hythe Bridge Street, Oxford OX1 2ET, UK; 5 Oxford University Hospitals National Health Service Trust, John Radcliffe Hospital, Oxford OX3 9DU, UK; 6 Present addresses: Department of Mathematics and Statistics, University of Reading, Reading RG6 6AX, UK (R.G.V.); 7 Present addresses: Department of Infectious Disease Epidemiology, Imperial College, London SW7 2AZ, UK (X.D.)

## Abstract

Horizontal gene transfer is an important driver of bacterial evolution, but genetic exchange in the core genome of clonal species, including the major pathogen *Staphylococcus aureus*, is incompletely understood. Here we reveal widespread homologous recombination in *S. aureus* at the species level, in contrast to its near-complete absence between closely related strains. We discover a patchwork of hotspots and coldspots at fine scales falling against a backdrop of broad-scale trends in rate variation. Over megabases, homoplasy rates fluctuate 1.9-fold, peaking towards the origin-of-replication. Over kilobases, we find core recombination hotspots of up to 2.5-fold enrichment situated near fault lines in the genome associated with mobile elements. The strongest hotspots include regions flanking conjugative transposon ICE*6013*, the staphylococcal cassette chromosome (SCC) and genomic island νSaα. Mobile element-driven core genome transfer represents an opportunity for adaptation and challenges our understanding of the recombination landscape in predominantly clonal pathogens, with important implications for genotype–phenotype mapping.

## Abstract (web summary)

Horizontal gene transfer occurs in most bacteria, yet it is unclear whether it happens in clonal species. Here, Everitt *et al.* show widespread within-species recombination, driven by mobile elements, in the genome of the pathogen *Staphylococcus aureus*, but no recombination between closely related strains.

## Graphical abstract

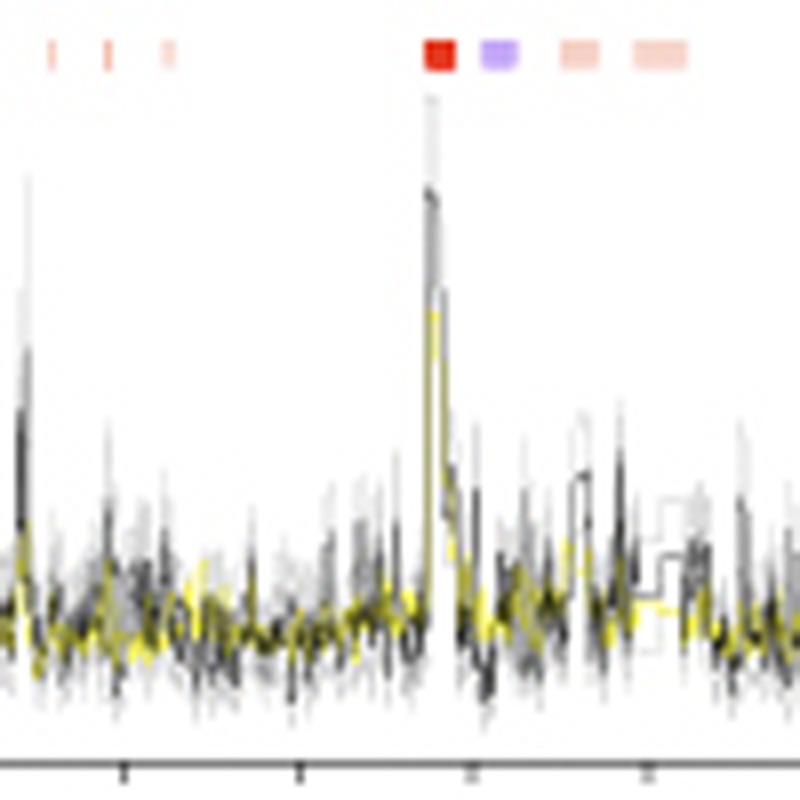

Bacteria are fundamentally clonal, reproducing by binary fission. The accessory genomes of bacteria are an important source of evolutionary novelty that facilitate rapid adaptation via horizontal gene transfer (HGT)^1^,^2^,^3^. Yet adaptation in the core genome is also critical to long-term survival and short-term response to new selection pressures: resistance to many antibiotics is conferred by substitutions in highly conserved core genes including *gyrA* and *rpoB*^4^,^5^,^6^. In the absence of homologous recombination mediated by HGT, adaptation in the core genome would be limited by the supply of new mutations and clonal interference^7^. Evidence for core genome transfer (CGT) has been reported in most studied bacteria^8^,^9^. However, the presence of CGT in apparently untransformable bacteria, among them major pathogens including *Staphylococcus aureus,* remains a paradox, and the underlying mechanisms obscure^10^,^11^,^12^.

Here we address the question of the frequency, distribution and local genomic context of HGT in the *S. aureus* core genome by examining signatures of genetic exchange among strains representative of species-level diversity, and we test whether the extreme rarity of CGT reported between highly related *S. aureus* strains^13^,^14^,^15^,^16^,^17^,^18^ applies species-wide. We discover a patchwork of hotspots and coldspots in the core genome driven by proximity to mobile elements against a backdrop of broad-scale trends in recombination rate variation that peak towards the origin-of-replication.

## Results

### Oxfordshire isolates encompass global *S. aureus* diversity

*S. aureus* is a life-threatening hospital pathogen and major cause of early mortality worldwide, but it is also a common constituent of the human microbiome, colonizing the noses of around one in three healthy adults^19^. Co-colonization rates among distinct strains are around 7%, providing ample opportunity for genetic exchange^20^. In 2009, we began a longitudinal carriage study of more than 1,100 asymptomatic adults in Oxfordshire, England, recruiting 360 nasal carriers for follow-up, in order to profile the natural reservoir of *S. aureus* colonizing humans^21^,^22^,^23^.

**Figure 1:**
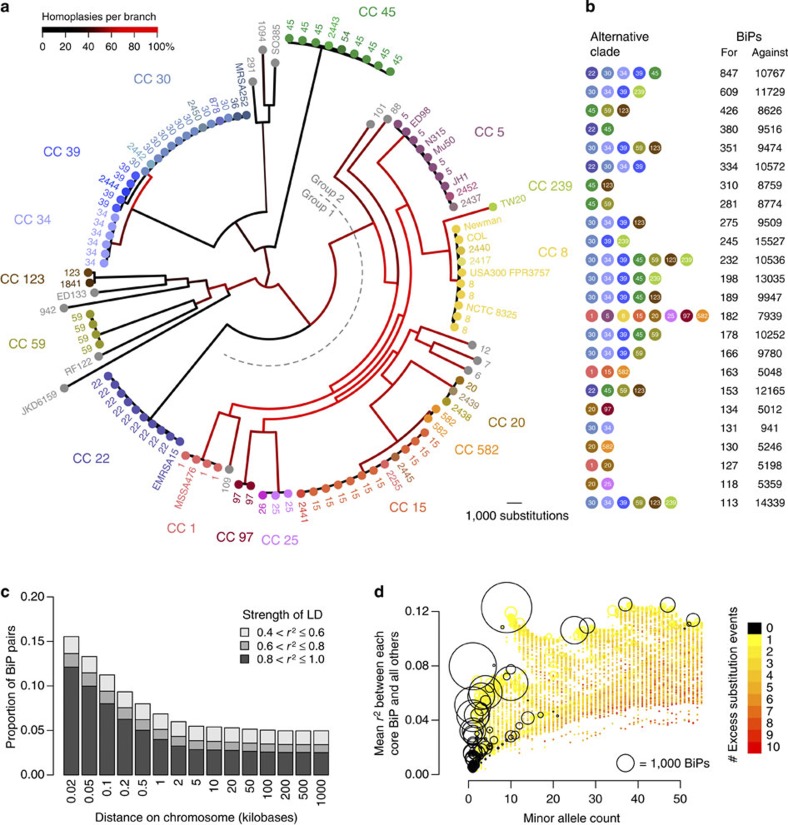
Signatures of recombination in the *S. aureus* core genome. (**a**) Maximum likelihood phylogeny of 95 isolates from Oxfordshire, England and 15 reference isolates, based on 2,114,882 core invariant and biallelic sites. The reference genomes represent international strains (Australia: JKD6159; Japan: Mu50, N315; UK: EMRSA15, MSSA476, TW20; USA: JH1, USA300), animal-associated strains (bovine: RF122; ovine: ED133; poultry: ED98, swine: SO385), and historic strains (1943: NCTC 8325; 1952: Newman; 1960s: COL). Branches are colour coded by the proportion of homoplasious substitutions. Isolates are labelled by ST or reference genome and colour coded by clonal complex. Group 1 and group 2 *S. aureus*, as previously defined^42^, are indicated. (**b**) Alternative, phylogenetically incongruent, relationships among CCs supported by some core biallelic sites but not others. (**c**) Decay in LD with increasing physical distance between pairs of core biallelic sites. LD is quantified by *r*^2^, the squared correlation coefficient. (**d**) Relationship between allele frequency, LD and number of substitutions at core biallelic sites. Each circle represents all the biallelic sites sharing a particular phylogenetic pattern, with the area proportional to the number of sites with that pattern. Circles are colour coded by the number of substitution events reconstructed along the phylogeny by maximum likelihood at each site with that pattern. Black circles correspond to the sites consistent with a unique mutation on a single branch of the phylogeny, while non-black circles represent homoplasious BiPs.

We randomly selected 89 methicillin-sensitive *S. aureus* (MSSA) and five methicillin-resistant *S. aureus* (MRSA) for Illumina whole-genome sequencing, taking the first positive nasal sample per carrier, and we resequenced MRSA252, an invasive reference isolate from Oxford^24^,^25^ (Supplementary Fig. 1). We compared these 95 genomes with 15 reference genomes representing international strains (Australia, Japan, UK and USA), animal-associated strains (bovine, ovine and poultry), and historic strains (1943, 1952 and circa-1960)^15^,^26^,^27^,^28^,^29^,^30^,^31^,^32^,^33^,^34^,^35^,^36^. A phylogeny based on 106,480 core biallelic polymorphisms (BiPs) shows the overarching relationships among the 110 genomes (Fig. 1a). Most isolates cluster into clonal complexes (CCs), differences between which dominate the phylogeny. Means of 0.13 and 7.8 BiP differences per kilobase were detected between samples of the same and different CC, respectively. The Oxfordshire isolates capture most of the species-level diversity. Hence, our collection combines global diversity and locally defined sampling, facilitating investigation of CGT throughout *S. aureus*.

### CGT is pervasive at the species level

To identify evidence for CGT, we focused our attention on BiPs, reducing confounding between signals of genetic exchange and repeat/back mutation. We classified core BiPs as follows^37^,^38^,^39^: autapomorphic (37,898 sites), sites in which the less frequent allele was observed in only one single genome; synapomorphic (34,218), sites compatible with a single substitution across the phylogeny; and homoplasious (34,364), sites requiring multiple substitutions across the tree. Autapomorphies are uninformative concerning genetic exchange, whereas synapomorphies are consistent with unique mutation and homoplasies are consistent with homologous recombination, subject to the caveat that we expect repeat/back mutation to have contributed a modest number (circa 1,500).

We detected three classic signatures of homologous recombination^39^,^40^,^41^, the first being a large excess of homoplasies. We identified 71,730 homoplasies (excess substitutions) across the 34,364 homoplasious BiPs. These homoplasies were particularly concentrated in Group 2 *S. aureus*, where they outnumbered synapomorphies more than threefold among some lineages (Fig. 1a). No other systematic differences between phylogenetic groups 1 and 2 have been identified^42^,^43^,^44^. Excess homoplasy was far greater between CCs than within (Supplementary Fig. 2), revealing that extremely low within-CC recombination rates do not generalize to the species level. The relative substitution rate due to recombination versus mutation (*r*/*m*) was estimated at 0.43 by LDhat^45^ and 0.83 (95% credible interval 0.67–1.1) by ClonalFrame^46^, higher than previously thought^9^,^14^,^42^ (Supplementary Table 1). Second, we found support among significant minorities of BiPs for alternate, phylogenetically incongruent clades (Fig. 1b). Almost every alternate configuration of Group 1 CCs was observed to have some support. CC-239, an acknowledged CC-8/CC-30 recombinant^47^, featured frequently among alternate groupings. Third, we observed a rapid decay of linkage disequilibrium (LD) with physical distance along the chromosome (Fig. 1c). Within 5 kb, LD decayed to the moderate residual levels observed between BiPs 1 Mb apart, this residual LD reflecting the strong structuring of the population into CCs. The scale of decay was consistent with mean DNA import lengths of 0.53 kb (LDhat) to 1.01 kb (ClonalFrame). Taken together, these classic signatures demonstrate frequent, widespread CGT during the long-term evolution of *S. aureus*.

The signatures of recombination are interrelated: accordingly, we observed a strong relationship between the number of homoplasies—that ranged from 0 to 10—and the strength of LD at core BiPs, having adjusted for allele frequency (Fig. 1d). We identified a similar relationship for gene presence/absence in the accessory genome, where the number of homoplasies ranged up to 35, reflecting the substantially higher rates of HGT (Supplementary Fig. 3). This relationship between homoplasy and LD allowed us to narrow our focus onto homoplasy in investigating genome-wide heterogeneity in CGT. We took as the relative positions of core BiPs those in MRSA252, supported by our observation that core gene synteny was conserved in all 110 genomes. This finding is helpful for investigating the influence of local genomic context on CGT.

### Genomic context predicts fine and broad-scale trends in CGT

**Figure 2:**
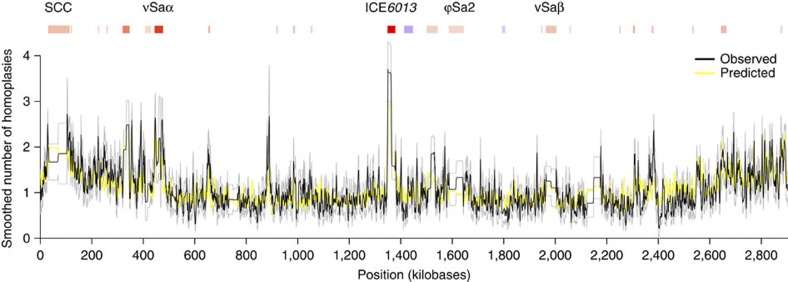
Recombination rate heterogeneity in the *Staphylococcus aureus* core genome. Genome-wide variation in the number of homoplasies per BiP, based on an exponential smoothing kernel with 1 kb bandwidth. The smoothed estimate of the mean number of observed homoplasies (black line) is shown +/− two s.e. (grey lines). Above, mobile elements that are significantly associated (*P*<0.05) with more (red) or fewer (blue) homoplasies are shown, with deeper shading for greater significance. The yellow line shows the smoothed estimate of the mean number of predicted homoplasies per BiP based on the regression model. The origin-of-replication occurs at position zero (extreme left and right side of the figure because of the circular nature of the chromosome).

Core genome homoplasy rates varied substantially, revealing a complex landscape of hot and cold regions (Fig. 2). The local homoplasy rate ranged from 0.20 to 3.68 based on a smoothing kernel with 1 kb bandwidth. We found a broad-scale trend towards greater homoplasy near the origin-of-replication. Against this trend, fine-scale variation manifesting as localized peaks of elevated homoplasy were scattered across the genome. We identified the hottest peak around 1,350 kb, the integration site of the ICE*6013* conjugative transposon in the MRSA252 genome^25^,^48^. Seven of the ten hottest regions were situated close to mobile elements or their insertion sites, including SCC and genomic island νSaα (Supplementary Table 2).

We systematically tested the influence of distance from origin, proximity to mobile elements and other local features on the number of homoplasies using negative binomial regression. After controlling for allele frequency, the first two of these were the most highly significant predictors (Table 1). Over megabase scales, homoplasy rates varied 1.9-fold, decreasing steadily with distance from origin before recovering slightly near the terminus (Supplementary Figs 4 and 5). Over kilobase scales, homoplasy was strongly associated with proximity to mobile elements. We estimated 2.5-fold and 1.9-fold enrichments in homoplasy surrounding the ICE*6013* integration site in MRSA252 and genomic island νSaα, respectively (Supplementary Table 3). In total, 20 mobile elements predicted locally elevated homoplasy, and five predicted reduced homoplasy. Reduced homoplasy was further associated with amino-acid substitutions, transversions, proximity to genes encoding translation machinery or signal transduction proteins, and high diversity, GC-poor and core-dense regions. Altogether, genomic context explained 44% of the variance in homoplasy at the kilobase scale, demonstrating the important modulatory effect of local features on CGT.

### Hotspots of CGT are associated with ICE*6013* and SCC

**Figure 3:**
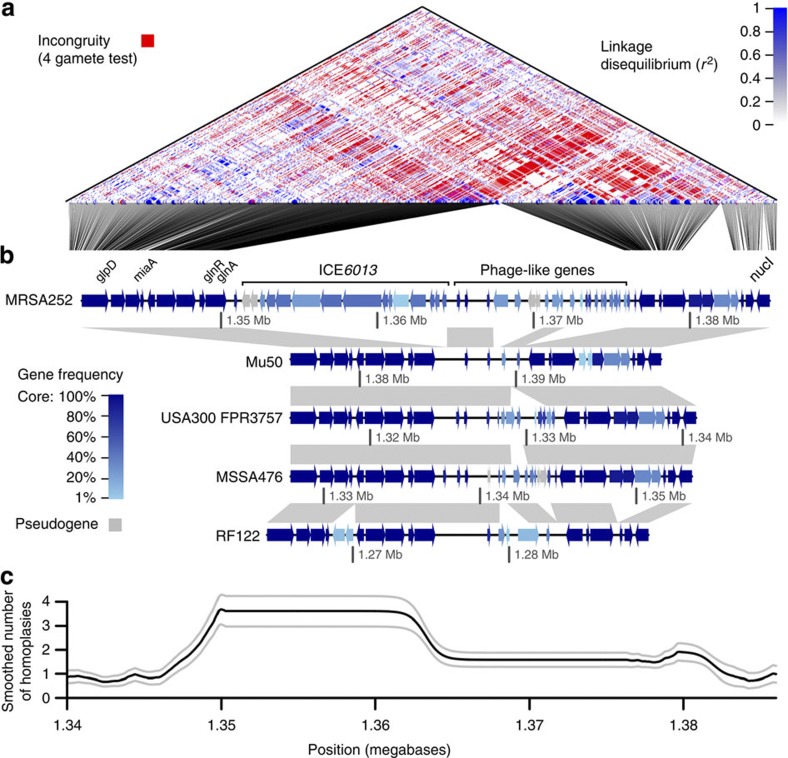
Recombination hotspot associated with the ICE*6013* conjugative transposon. (**a**) Pairwise LD plot. Red colouring indicates evidence for recombination between core BiPs, because they fail the four gamete test. Increasingly blue colouring indicates evidence against recombination, due to increasingly high *r*^2^. The positions of core BiPs in MRSA252 are indicated by corresponding black lines. (**b**) Alignment of five reference genomes in the region, with genes annotated. Dark grey bands between genomes indicate homology. Increasingly dark blue shading of genes indicates a higher frequency across the 110 genomes. Light grey shading indicates a pseudogene. (**c**) Smoothed number of observed homoplasies (black line), +/− two s.e. (grey lines). Whole-genome alignments were obtained from WebACT^71^ and plotted using genoPlotR^72^.

We examined in detail the strongest hotspot, associated with a 40 kb region spanning ICE*6013* and a cluster of phage-like genes between 1,345–1,385 kb in MRSA252, some 60 kb from the terminus-of-replication. The elevated homoplasy rate was accompanied by a marked reduction in LD and excess of phylogenetic incongruity between core BiPs spanning the region, suggesting a history of recurrent recombination (Fig. 3). An alignment of 16 reference genomes revealed substantial variability in gene content. MRSA252 alone contained conjugative machinery, while vestigial elements in the other genomes variously encoded pseudogenized phage head proteins, transposases and reverse transcriptase. Flanking the region, the highly conserved *glnA* was the hottest core gene (Supplementary Table 4). The rapid return to background levels of homoplasy within 5 kb demonstrated the diminishing influence of the mobile element with increasing physical distance.

**Figure 4:**
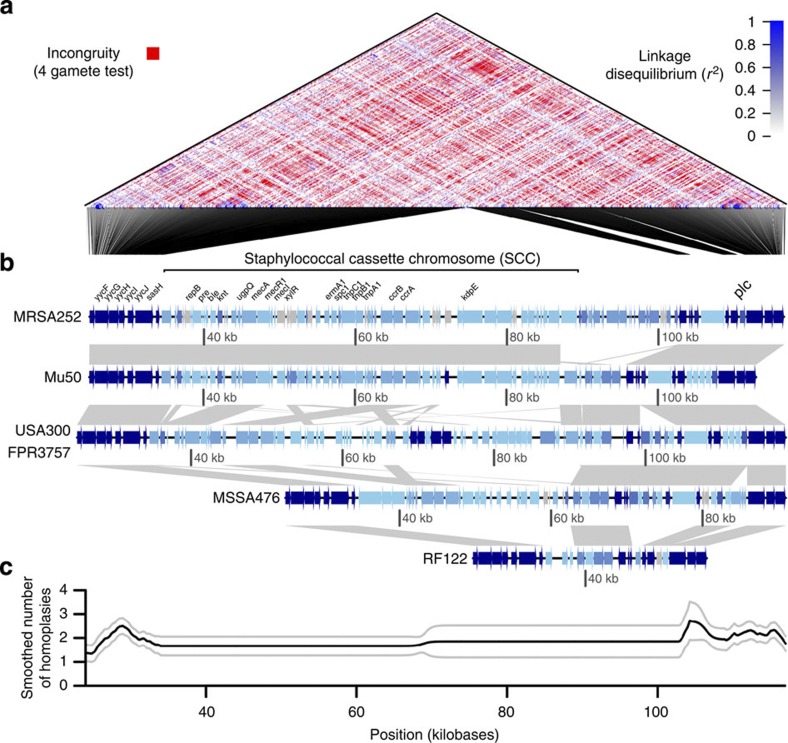
Recombination hotspot associated with the SCC. (**a**) Pairwise LD plot. Red colouring indicates evidence for recombination between core BiPs, because they fail the four gamete test. Increasingly blue colouring indicates evidence against recombination, due to increasingly high *r*^2^. The positions of core BiPs in MRSA252 are indicated by corresponding black lines. (**b**) Alignment of five reference genomes in the region, with genes annotated. Dark grey bands between genomes indicate homology. Increasingly dark blue shading of genes indicates a higher frequency across the 110 genomes. Light grey shading indicates a pseudogene. (**c**) Smoothed number of observed homoplasies (black line), +/− two s.e. (grey lines). Whole-genome alignments were obtained from WebACT^71^ and plotted using genoPlotR^72^.

Proximity to the origin-of-replication was strongly associated with excess homoplasy, particularly in the 750 kb immediately flanking the second strongest hotspot, found in SCC. The core BiPs surrounding SCC showed hallmarks of recurrent CGT, including low LD and phylogenetic incongruity (Fig. 4). In SCC, exemplified by the SCC*mec* element that confers methicillin resistance, gene content is extraordinarily variable. Homologous recombination over extended distances has been reported in the *ori*/SCC region: replacements of 244 kb in CC-34 (ref. 47) and 635 kb in CC-239 (ref. 31) span *ori*/SCC, the latter coinciding closely with the ~\n750 kb region of excess homoplasy that we identified (Fig. 2). The newly found SCC-associated hotspot represents the peak of this extended region of elevated homoplasy associated with large chromosomal replacements, indicating that mobile elements drive recurrrent CGT over scales ranging from just several kilobases extending up to nearly 1 Mb.

## Discussion

In summary, we found evidence of widespread homologous recombination in the core genome of *S. aureus*. Half of all informative sites were homoplasious, in contrast to the near-complete absence of homoplasy reported in whole-genome studies of highly related strains^13^,^14^,^15^,^16^,^17^,^18^. Strains of *S. aureus* are young relative to the species, with 60-fold less diversity within versus between CCs. Our results demonstrate a dramatically increased impact of recombination at the species versus the strain level^49^, indicating that the strong barriers to transformation in *S. aureus* do not prevent CGT over the long term. Cumulatively, rare events such as the transient emergence of hyperrecombinant lineages^50^,^51^, or low-level activation of transformation by cryptic gene expression programs^52^, could contribute to this phenomenon. We discovered broad- and fine-scale trends in homoplasy across the core genome. Similar observations of megabase-scale trends towards increased homoplasy near the origin-of-replication in *Escherichia coli* were hypothesized to arise from greater DNA copy number near the origin during exponential growth, providing more substrate for homologous recombination^53^. In *S. aureus*, the overlap between regions of elevated homoplasy and previously reported large chromosomal replacements lead us to believe that broad-scale trends in homoplasy are attributable to macro-recombination, most likely mobile element driven^31^,^47^. At fine scales, we discovered hotspots of CGT associated with proximity to mobile elements, including twofold enrichments within 1 kb of ICE*6013* and SCC integration sites, suggesting a causal role for mobile elements in generating hotspots. The size of transferred material, estimated at 500–1,000 bp, provisionally suggests different mechanisms at fine versus broad scales.

Besides the possibility of cryptic transformation^52^, generalized phage transduction and conjugative transfer represent candidate mechanisms for CGT, because they can transfer core material via accidentally mispackaged DNA, cargo genes or through imprecise excision^12^. Conjugative transposons have been shown to mobilize chromosomal DNA in *Enterococcus faecalis*^54^, *Vibrio cholerae*^55^, *Bacteroides thetaiotaomicron*^56^, *Streptococcus agalactiae*^57^ and *Clostridium difficile*^58^. Our finding that the core region immediately flanking the conjugative transposon ICE*6013* in MRSA252 is recombinationally active supports the proposition that ICE*6013* could drive CGT via a mechanism similar to Tn*GBS2* activity in *Streptococcus agalactiae*^48^. Like ICE*6013*, Tn*GBS2* integrates via a transposase rather than a site-specific recombinase, and Tn*GBS2* can mobilize chromosomal DNA through an Hfr-type mechanism that is capable of generating large chromosomal replacements, both in *cis* and in *trans*^57^,^59^. This raises the possibility that ICE*6013* could in fact be involved in broad-scale as well as fine-scale CGT.

Detecting genetic exchange in the core genome is important to understanding and monitoring the evolution of bacterial pathogens in response to selection pressures such as changing antibiotic usage. The discovery of a previously unknown landscape of hotspots in the core genome of a predominantly clonal bacterium casts new light on the prospects for genome-wide association studies, because unexpected levels of recombination improve the chances of finely mapping important phenotypes including virulence to specific loci in these important pathogens.

## Methods

### Isolate collection and sequencing

We surveyed asymptomatic nasal carriage in 1,123 adults attending general practices in Oxfordshire, England, 2009–2010 (refs 21, 22, 23). Informed consent was obtained from all participants, and ethical approval for the study was obtained from the Oxfordshire B Oxfordshire Research Ethics Committee (reference number 08/H0605/102). We selected a single colony for sequencing from the first positive nasal swab sampled from 94 carriers. Each nasal swab culture had been prepared and stored in glycerol. We incubated an inoculum of the glycerol stock on SASelect agar (Bio-Rad) overnight at 37 °C, then picked a single colony, streaked it onto Columbia blood agar and incubated it overnight at 37 °C, using a previously described protocol^21^. We grew one colony each from 89 randomly chosen MSSA carriers and five MRSA carriers, plus the clonal complex (CC)-30 reference isolate MRSA252 (ref. 25). Our sampling represents an enrichment of MRSA isolates compared with the overall frequency of 2.5% in the carriage study. For validation, we sequenced twice DNA extracted from eight of the colonies including MRSA252 by splitting the eight DNA extracts into two equal portions. In total, 103 genomes were sequenced at the Wellcome Trust Centre for Human Genetics, Oxford, using a combination of Illumina GAIIx and HiSeq 2000 with 96-fold multiplexing, paired-end reads of length of 99 or 101 bp each, insert sizes of 200 bp and mean depth 179 reads.

### Genome mapping and base calling

We used Stampy^60^ with no BWA premapping and an expected substitution rate of 0.01 to map each genome against the MRSA252 reference. A mean of 95.6% of reads mapped to MRSA252. We called bases using the SAMtools v0.1.18 mpileup command^61^ with options ‘-E -M0 -Q30 -q30 -o40 -e20 -h100 -m2 -F0.002 -g -D –S’ and the bcftools v0.1.17-dev view command with options ‘-c -g -b -A -L -t0.001 -i-1 -p0.5 –Pfull’. We masked calls that failed to meet the following criteria: at least five reads, at least one read in each direction, homozygous under a diploid model, at least 75% of reads supporting the call. Repetitive regions, defined by BLASTing the reference genome against itself and comprising 5.9% of the genome, were also masked. Following filtering, the mean proportion of the reference genome that we called by mapping was 86.4%. Out of 40,429,898 bases called independently across eight pairs of replicate genomes, there were no discrepancies.

### Pan genome

We constructed a database of the *S. aureus* pan genome from the coding sequence annotations of 16 Sanger-sequenced reference genomes. Coding sequences were appended sequentially to the database in the following order: MRSA252 (Genbank accession number BX571856.1), MSSA476 (BX571857.1), COL (CP000046.1), NCTC 8325 (CP000253.1), Mu50 (BA000017.4), N315 (BA000018.3), USA300_FPR3757 (CP000255.1), JH1 (CP000736.1), Newman (AP009351.1), TW20 (FN433596.1), S0385 (AM990992.1), JKD6159 (CP002114.2), RF122 (AJ938182.1), ED133 (CP001996.1), ED98 (CP001781.1), EMRSA15 (HE681097.1)^15^,^25^,^26^,^27^,^28^,^29^,^30^,^31^,^32^,^33^,^34^,^35^,^36^. Coding sequences that exhibited homology to other sequences already in the database, defined as 50% or greater identity in a tblastx query, were not added but noted as homologues.

### Genome assembly

We used Velvet^62^ to assemble reads into contigs *de novo* for each newly sequenced genome, with hash length chosen to optimize n50, yielding an average of 130.5 contigs per genome. We determined the presence or absence of the genes in the pan genome via a tblastx query with minimum 70% identity threshold. We identified the *S. aureus* core genome from the coding sequences that were present in all 94 carriage and 16 reference genomes.

### Synteny of the core genome

We assessed the synteny of the core genome by searching for anomalies in the expected ordering and orientation of successive core genes within contigs obtained from the Velvet assemblies of the 103 Illumina-sequenced genomes. Out of circa 200,000 pairs of successive core genes, we found 116 anomalies. Detailed inspection of mate pairs revealed that all were Velvet misassemblies. We also confirmed the conservation of core gene order and orientation in the 16 Sanger-sequenced reference genomes^49^.

### Identification of core BiPs

We defined core sites to be the 2,114,882 positions that mapped to MRSA252 and were unambiguously called in all 103 Illumina-sequenced genomes. Among those, we identified 106,480 core BiPs, 3,368 core triallelic polymorphisms and 66 core tetrallelic polymorphisms. We also identified 71,255, 4,012 and 125 non-core biallelic, triallelic and tetrallelic polymorphisms, respectively.

### Phylogenetic reconstruction

We characterized the evolutionary relationships between genomes by building a maximum likelihood tree using PhyML (version 3.0)^63^ assuming a single substitution rate under the HKY85 model and employing a combination of NNI and SPR moves in the search (options -b 0 -v 0 -c 1 -s BEST). We intentionally fitted a model without rate heterogeneity to ensure substitutions at different sites contributed equally to branch lengths. The maximum likelihood tree topology was robust to fitting a more complex GTR model with a proportion of invariant sites and gamma rate heterogeneity with four classes (options -m GTR -b 0 -v e -c 4 -s BEST). We analysed core BiPs and core invariant sites, with all non-core sites taken as invariant and identical to MRSA252. The PhyML tree was consistent with, but more fully resolved than, the 50% consensus tree constructed by ClonalFrame^46^ using the same data.

### Expected number of homoplasies caused by repeat/back mutation

We estimated the number of homoplasious BiPs that would be expected due to repeat or back mutation even in the absence of homologous recombination in two ways. (i) Using the substitution rate estimated by PhyML, we calculated the number of sites expected to experience two substitutions, offset by the probability that the second mutation did not generate a third allele, giving 1,480 homplasious BiPs. (ii) Taking the observed number of triallelic sites, we calculated the expected number of sites that would also have experienced two substitutions, but the second mutation did not generate a third allele, giving 1,684 homoplasious BiPs.

### Recombination rate estimation

We estimated recombination rate parameters, including the relative substitution rate due to recombination versus mutation (*r*/*m*) using LDhat^45^ and ClonalFrame^46^. In the ClonalFrame analysis, we ran 20,000 iterations of burn-in and 20,000 iterations of sampling, under default priors, and analysing 50% of SNPs to improve computation time. In the LDhat analysis, we maximized the composite likelihood over a grid of values of *γ* and 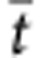
(which we refer to as *ρτ* and *τ*, respectively), focusing on the ranges 0–1.2 and 400–800, respectively following a wider initial search. We did not use ClonalFrame more widely because we observed that it did not detect recombination at a large proportion of sites where homoplasies were present.

### Detecting homoplasy

We estimated the number of substitution events at every core site across the PhyML tree using maximum likelihood ancestral state reconstruction^64^. For calculating homoplasy rates per branch of the PhyML tree, we downweighted each potential homoplasy by the probability that it was, in fact, the original mutation at that site. For this purpose, we considered each substitution at a site equally likely to have been the original mutation, rather than a homoplasy. A limitation of approaches such as ours that exploit homoplasy or genome mosaicism to detect recombination is that they rely on sampling descendants of both the recipient and donor lineage. An alternative method, but which also exploits this signal, has recently been developed^65^.

### Smoothed estimates of local homoplasy rate

We calculated a smoothed estimate of the homoplasy rate on an equally spaced grid of points every 50 bp throughout the reference genome as




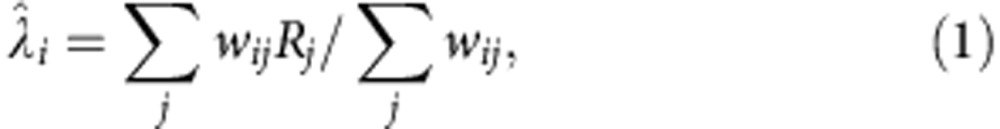




where *R*_*j*_ is the number of excess substitution events (that is, homoplasies) detected at informative BiP *j*, the summation being over all synapomorphic and homoplasious BiPs, and




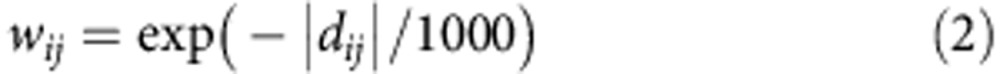




is the weighting function, designed to detect variation at the kilobase scale, where *d*_*ij*_ is the physical distance between the positions of grid point *i* and BiP *j* in MRSA252. We calculated the standard error similarly as




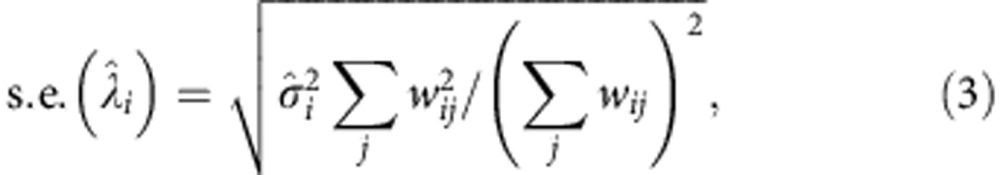




where




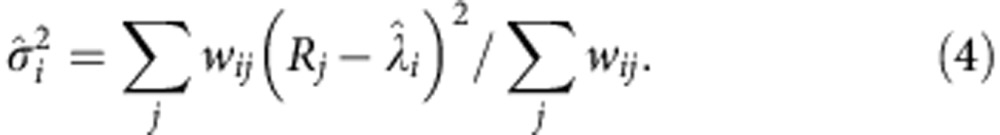




Regions with significantly increased or reduced recombination relative to the genome-wide average, 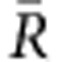, were ranked via a *Z*-score, defined at position *i* as




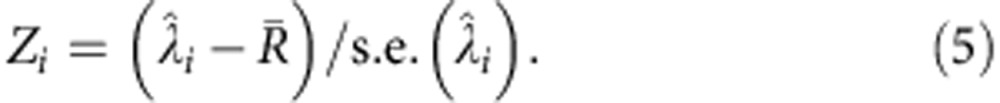




The behaviour of this smoothed estimate is such that the homoplasy rate (and s.e. and *Z*-score) at each point *i* draws on information from all BiPs, but downweights the influence of BiPs as physical distance between the positions of grid point *i* and BiP *j* increase. The strength of the weighting changes exponentially with physical distance between grid point *i* and BiP *j*. The influence of the 1 kb scale is such that if BiPs were evenly spaced every 30 bp, the total weight of BiPs within 1 and 6 kb, respectively would be 0.40 and 0.95. However, in regions where BiP density is low, more distant BiPs can contribute substantially. Smoothed homoplasy rates and LD plots for the entire genome, annotated by gene, are provided in Supplementary Data 1 and 2.

### Annotation of mobile elements

To identify the positions of mobile elements across the genomes, we used two approaches. Using blastn, we first collated sequences of known *S. aureus* mobile elements of different type (genomic island, integrated plasmid, prophage, SAPI, SCC, transposon) and searched for similar sequences in all the Oxfordshire isolates, attempting to allocate the type where possible^66^,^67^. We recorded the positions of flanking core BiPs for each. The accession numbers of the sequences used in blastn are given in Supplementary Table 5, along with the element-specific thresholds. Different approaches were taken for different elements: phages and SaPIs were located by blasting for integrase genes; SCC by blasting for genes in the *ccr* and *mec* complexes; transposons and plasmids by blasting for the whole element. We established thresholds by training the blastn queries on the published reference sequences, and the resequenced MRSA252 replicates. Lower thresholds were required when blasting for whole elements, which were often split across contigs, in comparison with the thresholds required when blasting for single genes.

We also developed an alternate method that exploits variable core BiP distance (VCBD)—that is, variability in the distance between adjacent core BiPs—to detect evidence of ancestral mobile activity. We identified the positions of core BiPs among the Velvet contigs by aligning them to the MRSA252 reference genome using the Mauve contig mover^68^. We calculated the variance in inter-BiP distance between adjacent BiPs on the same Velvet contig (or closed chromosome in the case of reference genomes) and divided by the mean distance between adjacent BiPs on the same contig to obtain a standardized variance. Applying as a threshold a standardized variance of 400 or above, we found 47 out of 65 known mobile elements, and 36 other regions of mobile activity (Supplementary Fig. 6). We manually curated all BLAST and VCBD hits to obtain a final set of 70 non-overlapping, annotated mobile elements (Supplementary Table 6).

### Regression analysis

To investigate the role of local genomic context in recombination rate heterogeneity, we analysed the number of homoplasies at informative BiPs using a negative binomial regression via the glm.nb command in the R statistical package^69^. We used the negative binomial regression because it can account for non-independence in the genome through the overdispersion parameter *θ*. We investigated the following covariates; at the individual BiP level: minor allele frequency, identity of major and minor alleles, mutation type (intergenic, synonymous, non-synonymous, read-through or nonsense), distance from the origin; as 1 kb moving averages: core region density, core region diversity, GC content; as 1 kb overlapping windows: the presence of genes in the MRSA252 reference genome encoding proteins (broken down by COG category^70^) and RNA (ribosomal RNA, transfer RNA or miscellaneous), and the presence of mobile elements identified as above (genomic island, integrated plasmid, prophage, SAPI, SCC, transposon and unclassified). We defined core region density as the local proportion of sites defined as core, which we identified above. We defined core region diversity to be the local proportion of core sites exhibiting a pairwise nucleotide difference, taken as a mean over all pairs of genomes. We assessed significance by dropping covariates or groups of covariates from the model, keeping the *θ* parameter fixed. We measured final goodness-of-fit by calculating *R*^2^, the squared correlation between mean observed and expected number of recombination events in non-overlapping 1 kb intervals. Details of all core BiPs, observed and predicted homoplasy and the predictors used for regression are provided in Supplementary Data 3.

### Poisson test

To rank individual genes for evidence of increased or reduced recombination irrespective of genomic context, we performed gene-by-gene Poisson tests for a significant difference in the number of recombination events at core BiPs compared with the genome-wide average, 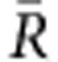, using the poisson.test command in R.

### Robustness to phylogenetic uncertainty

To test for robustness to uncertainty in the phylogeny, we conducted 100 bootstrap replicates as follows: we resampled the core invariant and biallelic sites and reconstructed the phylogeny using PhyML (options -b 100 -v 0 -c 1 -s BEST). We re-estimated the number of substitution events and homoplasies per site using maximum likelihood ancestral state reconstruction. We recalculated a smoothed estimate of genome-wide variation in homoplasy rate, and we re-fitted the negative binomial regression model to investigate the role of local genomic context on homoplasy rate variation. Supplementary Fig. 7 shows the robustness of the results to this procedure.

## Additional information

**Accession codes**: The DNA sequences have been deposited in the European Nucleotide Archive under accession code PRJEB5225 ( http://www.ebi.ac.uk/ena/data/view/PRJEB5225). Velvet assemblies were deposited in BIGSdb under project name ‘Everitt *et al.*’ ( http://pubmlst.org/rmlst/).

**How to cite this article**: Everitt, R. G. *et al.* Mobile elements drive recombination hotspots in the core genome of *Staphylococcus aureus*. *Nat. Commun.* 5:3956 doi: 10.1038/ncomms4956 (2014).

## Supplementary Material

Supplementary Information Supplementary Figures 1-7, Supplementary Tables 1-6 and Supplementary References.

Supplementary Data 1 Homoplasy and linkage disequilibrium in the *Staphylococcus* aureus core genome. Whole-genome LD plots are illustrated in 10kb windows. Each 10kb window is displayed as in Figure 3, with a single reference genome, MRSA252. Genes are color-coded by COG category or grey if unclassified. An extended coldspot can be seen between 1448-1458kb.

Supplementary Data 2 Homoplasy rates in the *Staphylococcus aureus* core genome. Smoothed estimates of the mean, standard deviation and standard error of the mean number of observed homoplasies are provided relative to positions in MRSA252. Smoothed estimates of the mean number of predicted homoplasies based on the negative binomial regression are also included.

Supplementary Data 3 Biallelic SNPs and predictors of homoplasy in the *Staphylocococcus aureus* core genome. Details of 106,480 core biallelic SNPs (BiPs) reported here, with observed homoplasy and, for the 68,582 informative (i.e. non-singleton) SNPs, the predicted number of homoplasies together with predictors used in the negative binomial regression. For CDS, Misc RNA, rRNA, tRNA and COG categories, a binary variable records whether the core BiP was within 1kb of an annotated element in the MRSA252 genome (1) or not (0). For mobile elements, a categorical variable records whether the core BiP was within 1kb, in which case the identity of that element is recorded otherwise none is recorded. Local core density, GC content and genetic diversity are calculated as 1kb-moving averages. For uninformative BiPs, predictors are generally not recorded, represented by a dash (-), because they were not used in the regression.

## Figures and Tables

**Table 1:** Predictors of homoplasy rates.

**Predictor**	**−log**_**10**_ **(** ***P*** **-value)**
BiP allele frequency	5051
Distance from origin	245
Proximity to mobile elements	96
BiP type (synonymous, non-synonymous, and so on)	52
Local genetic diversity	33
Local GC content	26
COG category of nearby genes	10
BiP identity (A→C, A→G, etc.)	9
Local core density	6

Significance (−log_10_ *P*-value) was calculated by systematically dropping each predictor group from the full negative binomial regression model.
