# Supplementary material for: Mobile elements drive recombination hotspots in the core genome of Staphylococcus aureus
Source: Nat Commun. 2014 May 23;5:3956. doi: 10.1038/ncomms4956 (PMC4036114; doi:10.1038/ncomms4956)
Supplement: Supplementary Information — Supplementary Figures 1-7, Supplementary Tables 1-6 and Supplementary References. [file ncomms4956-s1.pdf]

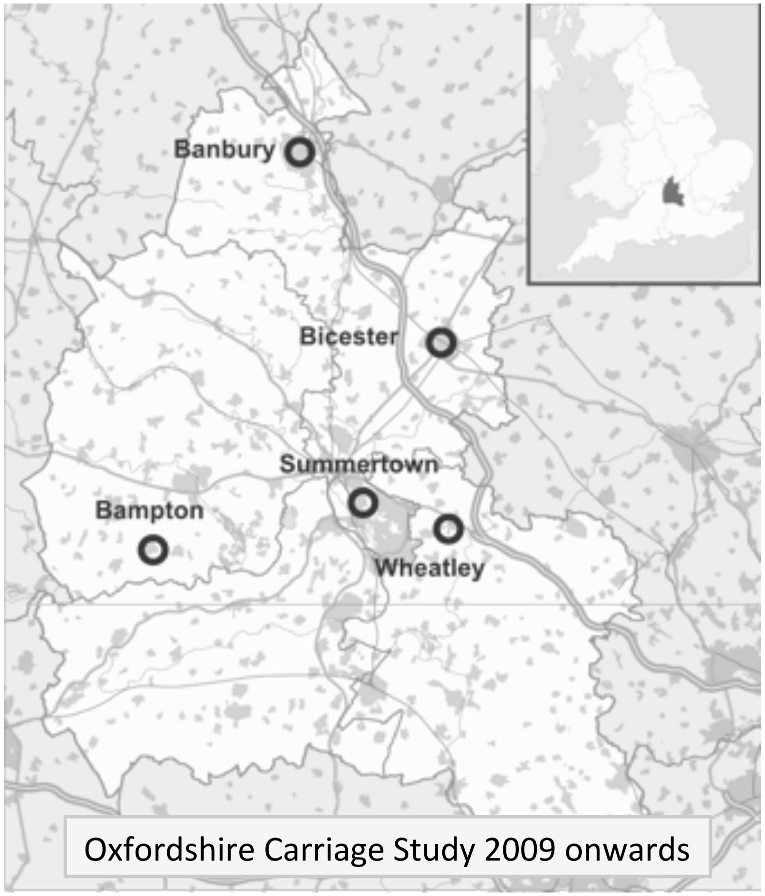

95 nasal samples: 89 MSSA swabs
5 MRSA swabs
1 reference

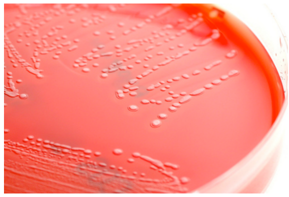

genome of one colony from one nose swab

- 1,123 people recruited for initial nose swabs
- 360 recruitment positives, and 211 of the recruitment negatives, followed longitudinally
- Swabs returned at bimonthly intervals
- 431 participants with a *S. aureus*-positive swab to August 2010
- 89 MSSA and 5 MRSA carriers selected at random
- First available *S. aureus* positive swab sampled per individual

Supplementary Figure 1 I Sampling locations and strategy. Beginning in 2009, we recruited 1,123 adults from general practices in and around Oxford, England, to take part in a survey of asymptomatic nasal carriage of *S. aureus*. As of August 2010, 431 participants had returned at least one *S. aureus*-positive swab. We randomly selected for sequencing 89 MSSA carriers and 5 MRSA carriers, taking a single colony from the first positive swab returned per individual. This represents a two-fold enrichment of MRSA compared to its prevalence in the carriage population.

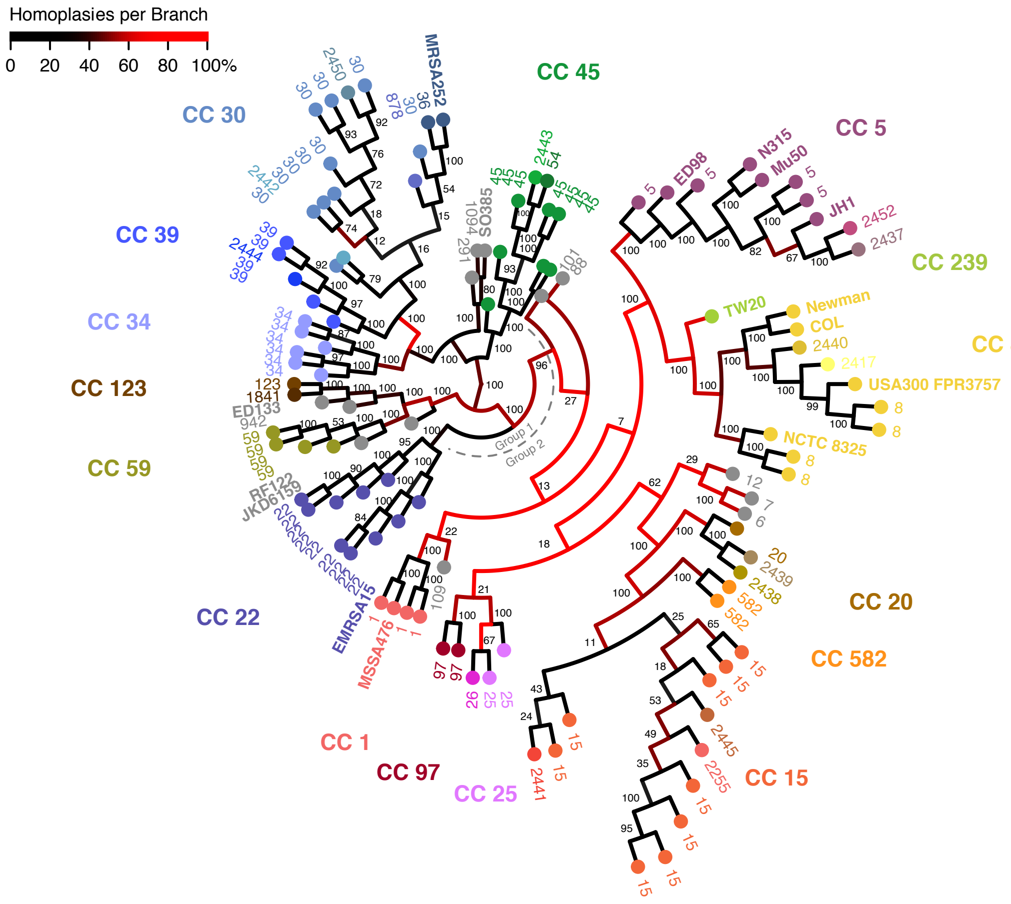

Supplementary Figure 2 | Comparison of homoplasy rates within versus between clonal complexes. Re-scaled maximum likelihood phylogeny of the 95 isolates from Oxfordshire, England and 15 reference isolates depicted in Figure 1a. Branch lengths have been equalized relative to Figure 1a to allow visualization of homoplasy rates on short branches within clonal complexes. As in Figure 1a, branches are color-coded by the proportion of homoplasious substitutions and isolates are labeled by ST or reference genome and color-coded by clonal complex. Bootstrap values for each branch are indicated in small black type, calculated from 100 simulations using PhyML.

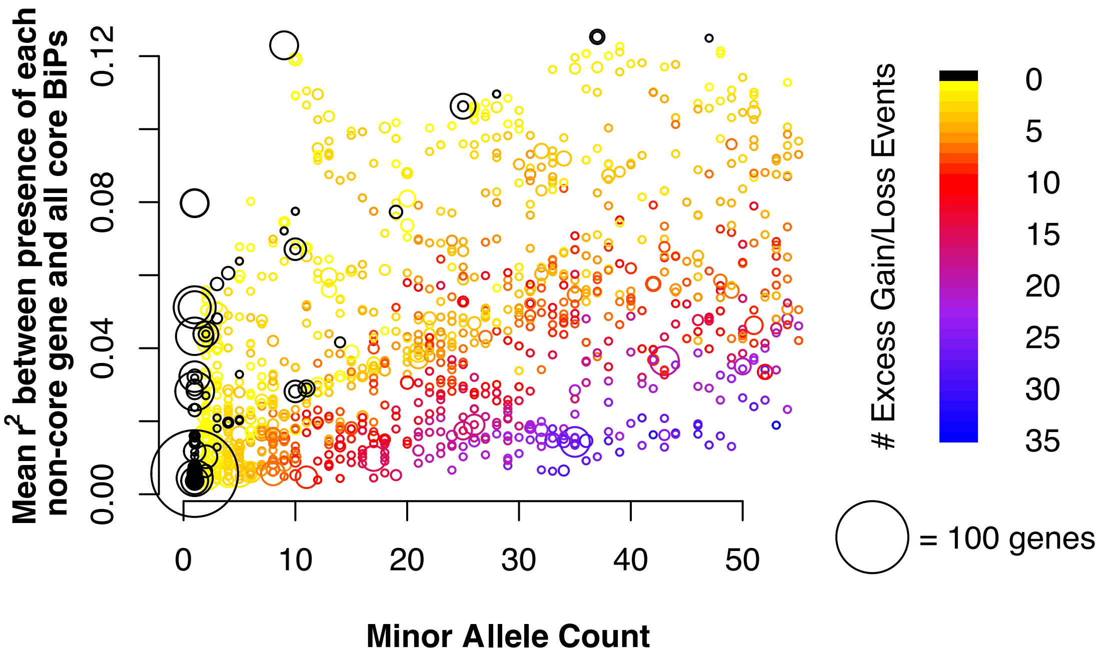

Supplementary Figure 3 l Relationship between allele frequency, LD and number of gene gain/loss events in the accessory genome. Each circle represents a unique phylogenetic pattern of gene presence/absence, with the area proportional to the number of accessory genes with that pattern. Circles are color-coded by the number of gain/loss events reconstructed along the phylogeny by maximum likelihood. Black circles correspond to genes consistent with a unique gain/loss on a single branch of the phylogeny, while non-black circles represent homoplasious genes (genes that have been gained/lost more than once). LD is measured as the mean value of r^2 between the presence/absence of the accessory gene and each core BiP.

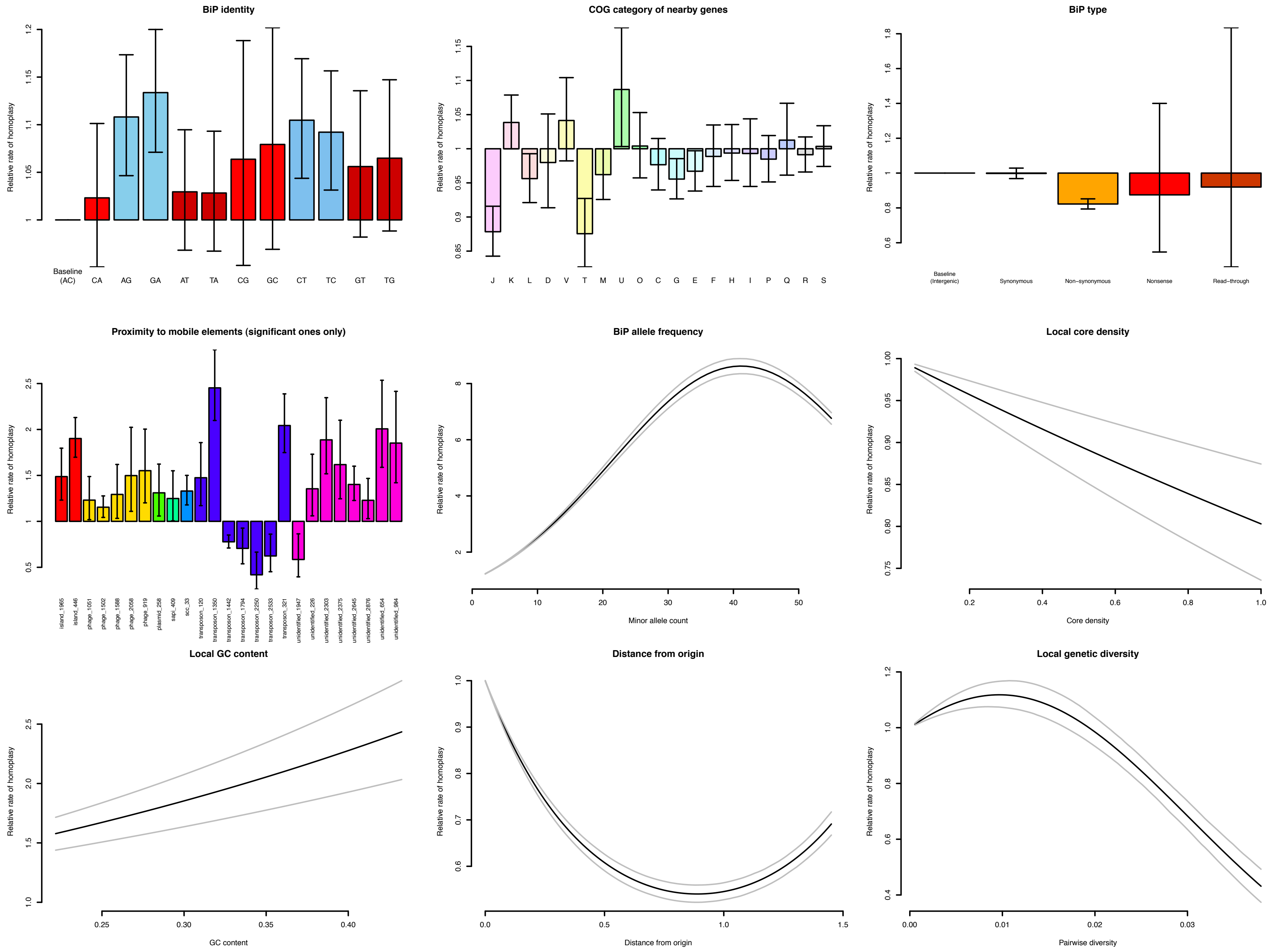

Supplementary Figure 4 | Effect of local genomic context on homoplasy rates: parameter estimates. For each group of predictors in the negative binomial regression, the point estimate +/– two standard errors is shown for individual coefficients.

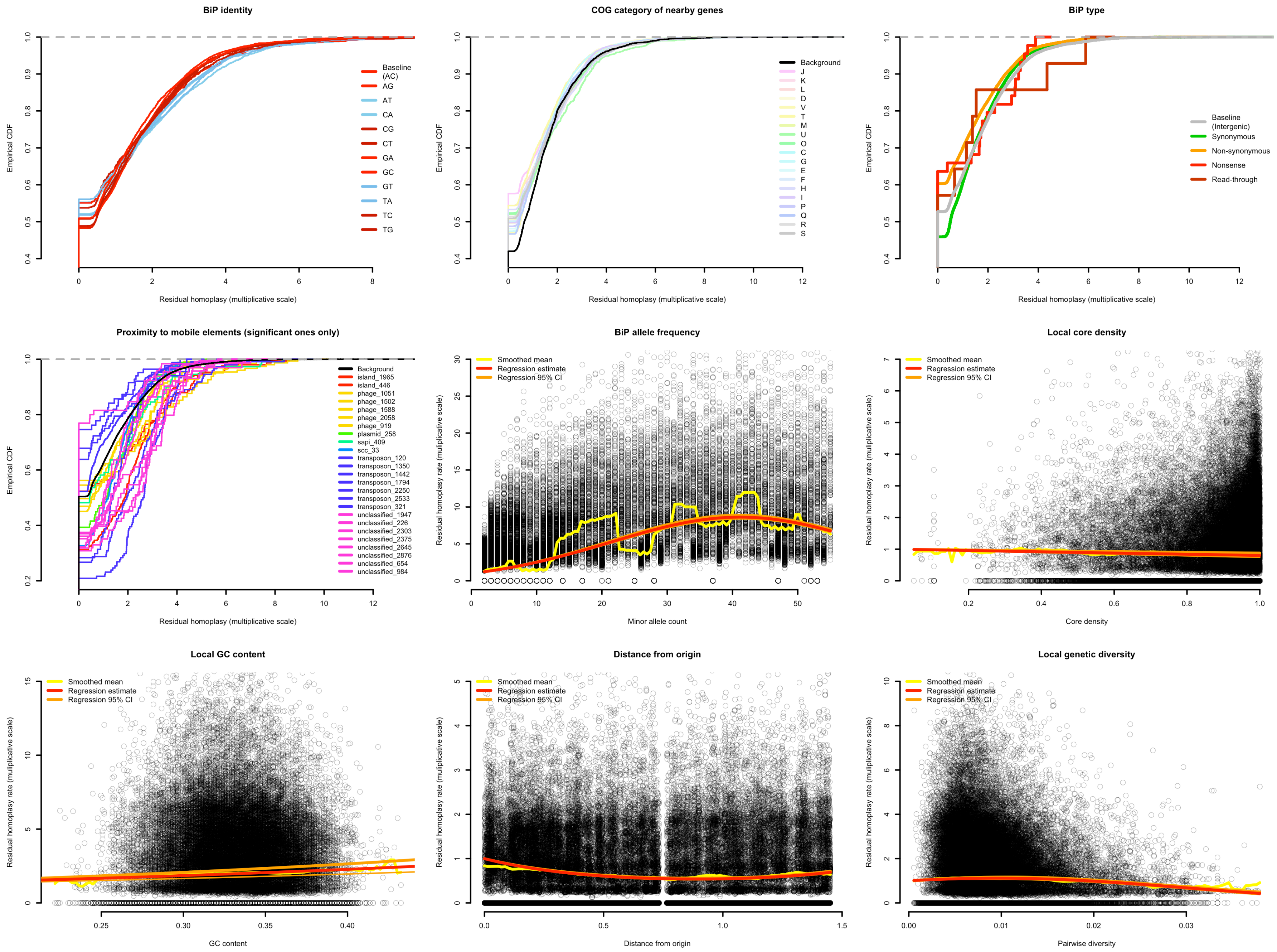
BiP identity
Empirical CDF
Residual homoplasy (multiplicative scale)
Baseline (AC)
AG
AT
CA
CG
CT
GA
GC
GT
TA
TC
TG
COG category of nearby genes
Background
J
K
L
D
V
T
M
U
O
C
G
E
F
H
I
P
Q
R
S
BiP type
Baseline (Intergenic)
Synonymous
Non-synonymous
Nonsense
Read-through
Proximity to mobile elements (significant ones only)
Background
island_1965
island_446
phage_1051
phage_1502
phage_1588
phage_2058
phage_919
plasmid_258
sapi_409
scc_33
transposon_120
transposon_1350
transposon_1442
transposon_1794
transposon_2250
transposon_2533
transposon_321
unclassified_1947
unclassified_226
unclassified_2303
unclassified_2375
unclassified_2645
unclassified_2876
unclassified_654
unclassified_984
BiP allele frequency
Smoothed mean
Regression estimate
Regression 95% CI
Residual homoplasy rate (muliplicative scale)
Minor allele count
Local core density
Core density
Local GC content
GC content
Distance from origin
Distance from origin
Local genetic diversity
Pairwise diversity

Supplementary Figure 5 | Effect of local genomic context on homoplasy rates: signal in the data. For each group of predictors in the negative binomial regression, the residual rate of homoplasy is shown in order to visualize the signal in the data driving the parameter estimates. For this purpose, we defined the residuals as the ratio of the observed number of homoplasies per BiP divided by the predicted number under the fitted model having excluded that particular group of predictors. For categorical predictors, empirical cumulative distribution functions (CDFs) are shown. Variables that predict an increased homoplasy rate relative to background or baseline shown a right-shift in the empirical CDF, and variables that predict a decreased homoplasy rate show a left-shift. For continuous predictors, a scatter plot of the residuals versus the variable is shown, with a smoothed mean residual, together with the fitted effect of the variable, +/– two standard errors. Except for BiP allele frequency, the fitted effect closely tracks the smoothed mean, indicating a reasonable choice of parametric relationship (linear in the case of core density and GC content, quadratic in the case of BiP allele frequency, distance from origin and genetic diversity). For BiP allele frequency, the smoothed mean shows periodic dips in homoplasy. These dips are associated with clonal BiPs with zero homoplasy that occur at certain frequencies. The intention of fitting a quadratic curve is to model the informativeness of BiPs regarding homoplasy as a function of allele frequency, a relationship that we expect actually varies continuously.

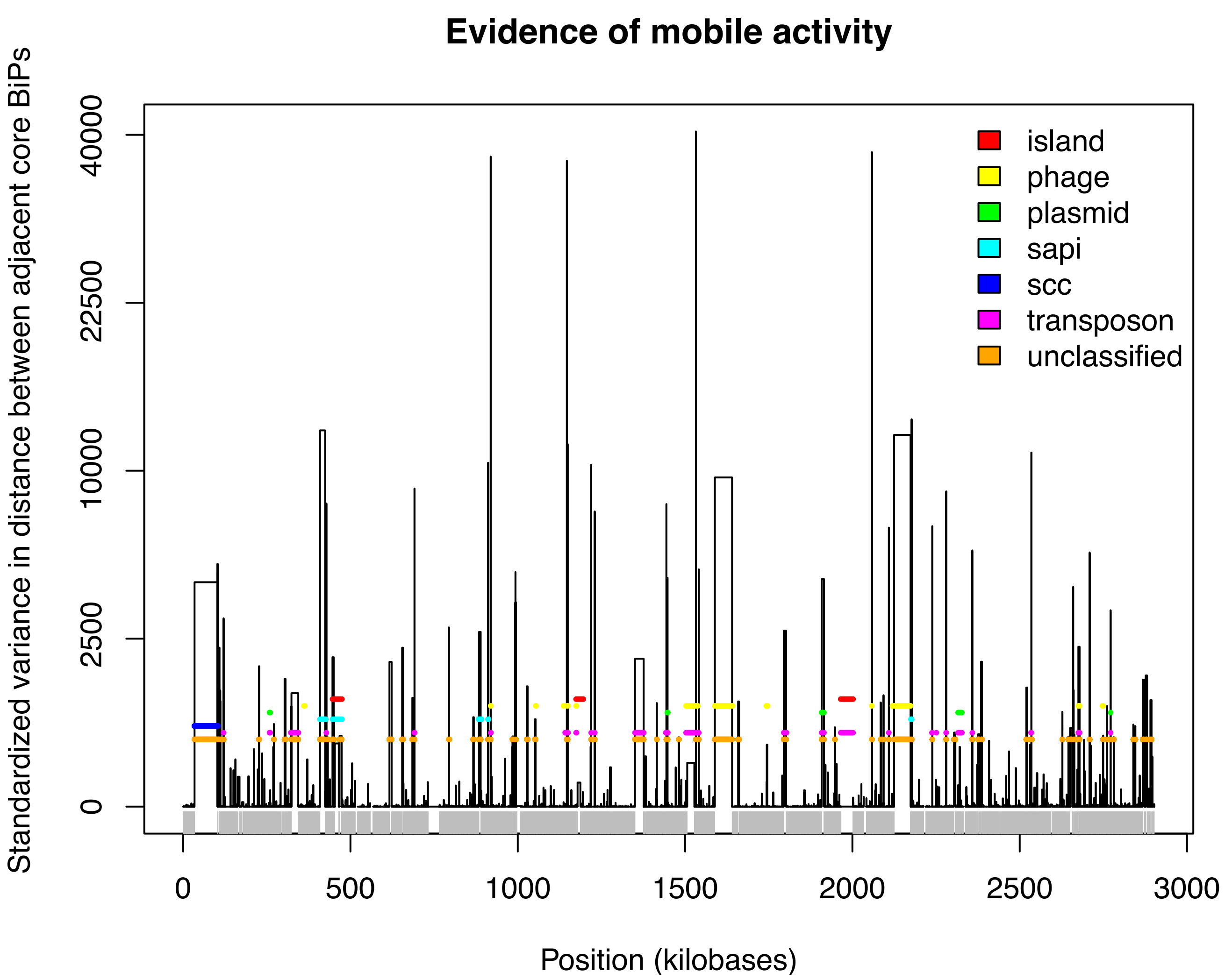

Supplementary Figure 6 | Identifying mobile activity from the standardized variance in distance between adjacent core BiPs. We calculated the variance in core BiP distance (VCBD) between adjacent BiPs on the same Velvet contig (or closed chromosome in the case of reference genomes) and divided by the mean distance to obtain a standardized variance (shown on a square root scale, solid black line). Applying a threshold of at least 400, we found 47 out of 65 known mobile elements (indicated by horizontal lines colored as follows: red: genomic island, yellow: prophage, green: integrated plasmid, cyan: *S. aureus* pathogenicity island, blue: staphylococcal cassette chromosome, magenta: transposon), and 36 other regions of mobile activity (orange horizontal lines). The positions of core BiPs are shown as vertical grey ticks below the main plot.

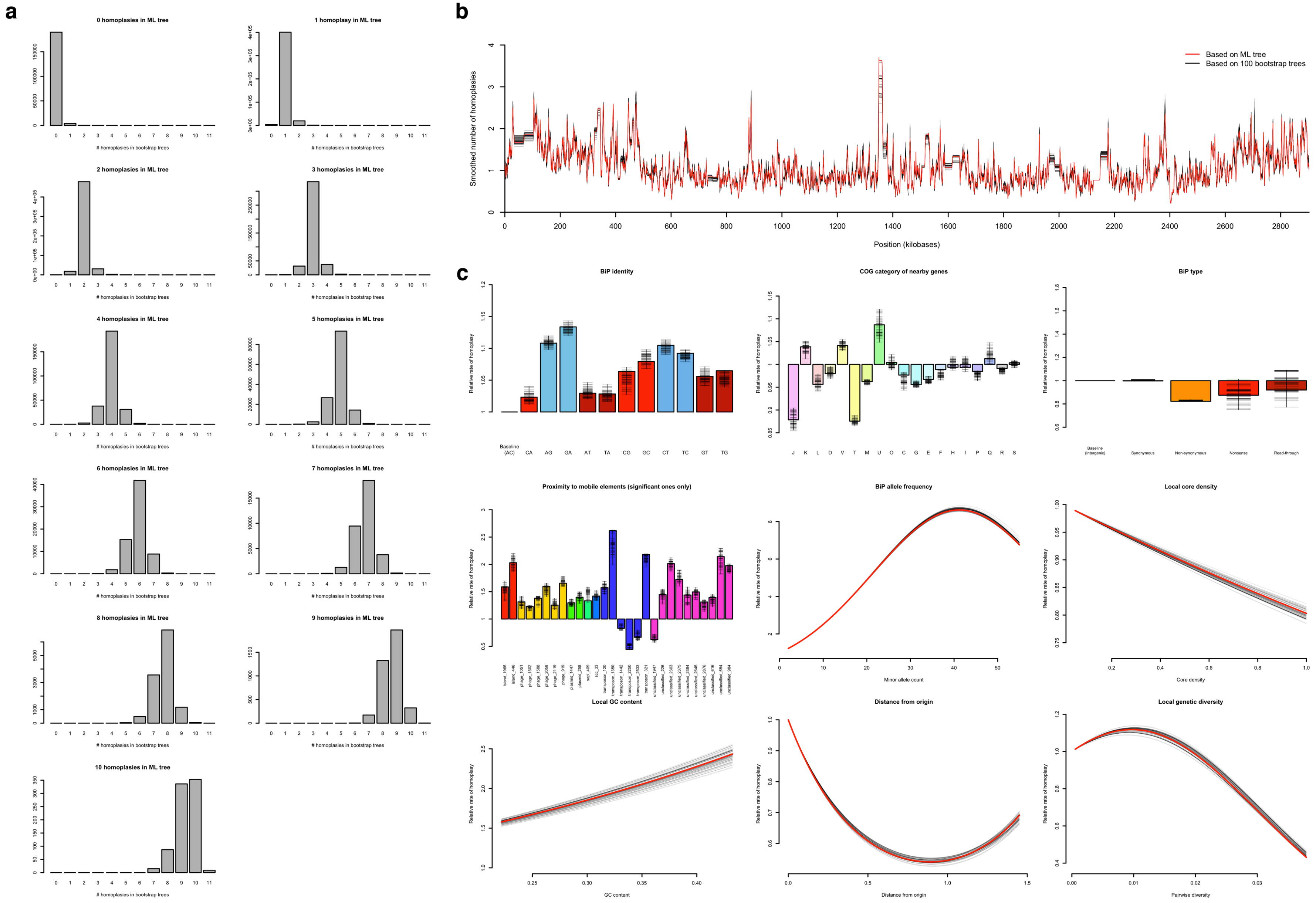

a
0 homoplasies in ML tree
1 homoplasy in ML tree
2 homoplasies in ML tree
3 homoplasies in ML tree
4 homoplasies in ML tree
5 homoplasies in ML tree
6 homoplasies in ML tree
7 homoplasies in ML tree
8 homoplasies in ML tree
9 homoplasies in ML tree
10 homoplasies in ML tree
homoplasies in bootstrap trees
b
Based on ML tree
Based on 100 bootstrap trees
Smoothed number of homoplasies
Position (kilobases)
c
BiP identity
Baseline (AC)
CA
AG
GA
AT
TA
CG
GC
CT
TC
GT
TG
COG category of nearby genes
J K L D V T M U O C G E F H I P Q R S
BiP type
Baseline (Intergenic)
Synonymous
Non-synonymous
Nonsense
Read-through
Relative rate of homoplasy
Proximity to mobile elements (significant ones only)
BiP allele frequency
Minor allele count
Local core density
Core density
Local GC content
GC content
Distance from origin
Local genetic diversity
Pairwise diversity

Supplementary Figure 7 | Robustness to phylogenetic uncertainty. (**a**) For each of the 100 bootstrap replicate phylogenies generated by PhyML, the number of homoplasies per informative BiP was re-evaluated. Bar charts show the distribution of the number of homoplasies recovered across the 100 bootstrap replicates, grouped by the number of homoplasies detected in the original ML phylogeny. In general, the distribution is tightly clustered around the number originally detected. (**b**) For each of the 100 bootstrap replicates, genome-wide variation in the number of homoplasies per BiP, was re-estimated, based on an exponential smoothing kernel with 1 kb bandwidth (black lines). There is a high degree of concordance between the 100 bootstrap replicates and the original estimate based on the ML phylogeny (red line), making them difficult to distinguish. The red line is the same as the black line in Figure 2a. (**c**) For each bootstrap replicate, the negative binomial regression model relating homoplasy rates to local genomic context was re-fitted. For each group of predictors in the negative binomial regression, the point estimate based on the original data (also shown in Supplementary Figure 4) is illustrated together with the point estimates from each of the 100 bootstrap replicates. In general, the uncertainty attributable to reconstruction of the phylogeny is small compared to inherent uncertainty in the parameter estimates (error bars in Supplementary Figure 4).

Supplementary Table 1 I Evolutionary parameters estimated by LDhat and ClonalFrame. The mutation rate, short- and long-range recombination rate, average length of DNA imported during recombination, the relative rate of recombination vs. mutation and the relative rate of substitutions attributable to recombination vs. mutation are shown, along with previous estimates for *Staphylococcus aureus*.

Parameter	LDhat[a]	ClonalFrame[b,c]	Previous estimates	
			Studies of individual strains	Studies of disparate strains
Mutation rate[d] (θ)	9.2 kb^{-1}	9.2 kb^{-1}		23 kb^{-1} ([1])
Local recombination rate (ρ)	1.6 kb^{-1}	2.1 kb^{-1} (1.7, 2.5)		5.1 kb^{-1} ([1]), 2.2 kb^{-1} ([2,e])
Average DNA import length (τ)	0.53 kb	0.99 kb (0.69, 1.5)		
Long-range recombination rate ($\rho\tau$)	0.85	2.0 (1.4, 3.1)		
Relative rate of recombination vs. mutation (ρ/θ)	0.17	0.21 (0.18, 0.26)	0.06, 0.02 ([3])	0.2 ([2,e]), 0.45-0.6 ([4])
Relative substitution rate attributable to recombination vs. mutation[f] (r/m)	0.43	0.83 (0.67, 1.1)	0.11 ([5])	0.59 ([2,e]), 0.1 ([6]), 0.07 ([7])

[a] Maximum composite likelihood estimate is shown. LDhat does not provide well-calibrated confidence intervals

[b] Point estimate and credible intervals given as the 50% and (2.5, 97.5)% posterior quantiles respectively

[c] Note that the recombination parameter R estimated by ClonalFrame (population-scaled rate of initiation of recombination) equals half the recombination parameter ρ defined by LDhat (population-scaled rate of initiation or termination of recombination)

[d] Watterson estimates used for both LDhat and ClonalFrame

[e] Assuming a mean DNA import length of 1 kb.

[f] The calculation for LDhat is $\rho\tau/2$ and for ClonalFrame $R\nu/(\delta\theta)$

Supplementary Table 2 | Hottest regions by peak homoplasy rate. Regions were ordered by the Z score described in the Methods. Contiguous regions were merged, and the top ten hottest regions reported, ordered by peak Z score within each. Positions are provided relative to the MRSA252 reference genome. Mobile elements were variably present; not all were present in MRSA252.

Rank	Start	End	Peak Z score	Features [a] (element label [b])
1	1,349,350	1,350,500	4.46	ICE6013 conjugative transposon (transposon_1350)
2	104,500	104,750	3.37	Staphylococcal cassette chromosome (scc_33)
3	343,500	344,100	3.26	ICE6013-like conjugative transposon (transposon_321)
4	2,693,450	2,693,850	3.06	Sortase *srtA*, phosphinothricin N-acetyltransferase SAR2609
5	888,950	889,300	2.97	*Staphylococcus aureus* pathogenicity island (sapi_882)
6	355,950	356,150	2.85	Sodium/solute symporter family protein SAR0311
7	2,771,850	2,772,150	2.84	Integrated plasmid, Tn552-like transposon (plasmid_2771)
8	2,322,550	2,322,600	2.80	Integrated plasmid, Tn552-like transposon (plasmid_2315)
9	391,350	391,800	2.78	Lipoprotein SAR0340, exported protein SAR0341
10	446,650	446,650	2.67	Genomic island α, *S. aureus* pathogenicity island (island_446)

[a] Note that mobile elements are variably present across genomes. Core genes are reported only in the absence of nearby mobile elements in any genomes. [b] See Supplementary Table 5.

Supplementary Table 3 l Prediction coefficients for homoplasy rate. For each predictor, the estimated coefficient in the negative binomial regression based on a $\log_e$ link function is recorded, together with the standard error, corresponding Z statistic, and an approximate p-value based on the Z statistic. For formal significance testing of groups of predictors, see Table 1. For details of the mobile elements, see Supplementary Table 6.

	Estimate	Std. Error	Z statistic	p-value	
Intercept	-1.435	0.09578	-14.978	$< 2\times10^{-16}$	***
BiP allele frequency					
Linear effect	11.51	0.1244	92.513	$< 2\times10^{-16}$	***
Quadratic effect	-15.38	0.2514	-61.16	$< 2\times10^{-16}$	***
Distance from origin (Mb)					
Linear effect	-1.385	0.04666	-29.685	$< 2\times10^{-16}$	***
Quadratic effect	0.7792	0.03222	24.181	$< 2\times10^{-16}$	***
Proximity (<1kb) to genomic islands					
446 Genomic island α	0.6428	0.0567	11.337	$< 2\times10^{-16}$	***
1174 Genomic island γ †	-0.06524	0.06528	-0.999	0.31763	
1965 Genomic island β	0.397	0.0944	4.206	0.000026	***
Proximity (<1kb) to prophages					
361 Phage type VI-like	0.07282	0.08604	0.846	0.397397	
919 Phage type I-like †	0.4392	0.1279	3.434	0.000595	***
1051 Phage type III-like	0.2078	0.09468	2.195	0.028157	*
1136 Phage type VII-like	0.0474	0.1597	0.297	0.766587	
1146 Phage type VII-like †	0.1291	0.1019	1.267	0.205319	
1502 Phage type III-like †	0.1432	0.0506	2.83	0.00465	**
1588 Phage type II-like	0.257	0.1125	2.284	0.022381	*
1742 Phage type II-like	0.1071	0.09731	1.101	0.271059	
2058 Phage type V-like	0.4041	0.1504	2.688	0.007194	**
2119 Phage type II/III-like	0.1609	0.0851	1.89	0.058725	.
2675 Phage type II-like †	0.1788	0.1273	1.404	0.160302	
2747 Phage type II-like	-0.0352	0.08619	-0.408	0.68298	
Proximity (<1kb) to integrated plasmids					
258	0.2704	0.1069	2.529	0.011452	*
1447 †	0.1946	0.1109	1.755	0.079338	.
1908	-0.07899	0.1453	-0.544	0.586608	
2315	0.07146	0.08553	0.835	0.403455	
2771	0.135	0.1133	1.191	0.233803	
Proximity (<1kb) to SAPIs					
409 †	0.2221	0.1082	2.053	0.0401	*
882	0.1774	0.1033	1.718	0.085869	.
911	-0.04986	0.1414	-0.353	0.724397	
Proximity (<1kb) to SCC	0.2852	0.06028	4.731	0.00000223	***

		Estimate	Std. Error	*Z* statistic	*p*-value	
Proximity (<1kb) to transposons						
120	ICE*6013*-like †	0.3885	0.1153	3.371	0.00075	***
321	ICE*6013*-like †	0.7145	0.07787	9.175	$< 2\times10^{-16}$	***
691	ICE*6013*-like †	-0.03107	0.1491	-0.208	0.834934	
1141	Tn*552*-like	-0.1692	0.1526	-1.109	0.267393	
1219	ICE*6013*-like †	0.02067	0.2172	0.095	0.924168	
1229	ICE*6013*-like †	-0.03102	0.1734	-0.179	0.858001	
1350	ICE*6013* †	0.8973	0.07813	11.484	$< 2\times10^{-16}$	***
1442	ICE*6013*-like †	-0.2518	0.04532	-5.557	2.74×10^{-8}	***
1794	Tn*554*-like	-0.3485	0.1359	-2.564	0.010335	*
2108	Tn*552*-like	-0.2387	0.2053	-1.163	0.245027	
2237	ICE*6013*-like †	-0.01212	0.1191	-0.102	0.918936	
2250	φTn*554*-like	-0.8714	0.2317	-3.76	0.00017	***
2279	ICE*6013*-like †	0.2576	0.1505	1.711	0.087062	.
2358	ICE*6013*-like †	0.06411	0.1357	0.472	0.636595	
2533	ICE*6013*-like †	-0.4725	0.1612	-2.932	0.003369	**
Proximity (<1kb) to unclassified mobile elements						
226		0.3034	0.1226	2.475	0.01331	*
271		-0.245	0.1536	-1.596	0.110562	
303		-0.2057	0.1494	-1.378	0.168334	
431		0.0908	0.1365	0.665	0.505928	
616		0.2673	0.1461	1.83	0.067242	.
654		0.6965	0.117	5.953	2.63×10^{-9}	***
684		-0.05557	0.2058	-0.27	0.787097	
794		0.1644	0.1747	0.941	0.34683	
866		0.1421	0.1677	0.848	0.396705	
984		0.6162	0.1327	4.644	0.00000342	***
992		-0.2637	0.1725	-1.528	0.126414	
1027		0.1876	0.1881	0.997	0.318694	
1481		-0.2016	0.1383	-1.458	0.144908	
1658		0.1992	0.1643	1.212	0.225422	
1947		-0.5362	0.1949	-2.751	0.005949	**
2084		-0.1298	0.1792	-0.724	0.468904	
2093		0.2398	0.1472	1.629	0.103301	
2303		0.635	0.1089	5.833	5.45×10^{-9}	***
2375		0.481	0.1306	3.682	0.000231	***
2384		0.2986	0.1573	1.898	0.057678	.
2519		-0.2593	0.1556	-1.667	0.095509	.
2627		0.1244	0.1274	0.976	0.329066	
2645		0.3376	0.06633	5.089	0.00000036	***
2709		-0.1466	0.1359	-1.079	0.280717	

	Estimate	Std. Error	*Z* statistic	*p*-value	
2761	0.06426	0.1504	0.427	0.669116	
2781	-0.2262	0.1723	-1.313	0.189238	
2839	-0.3097	0.2059	-1.504	0.132487	
2844	-0.04299	0.09785	-0.439	0.660392	
2867	-0.09218	0.1187	-0.777	0.437249	
2876	0.2062	0.08854	2.328	0.019894	*
2890	0.1005	0.1238	0.812	0.416527	
BiP type (cf Intergenic)					
Non-synonymous	-0.1955	0.01769	-11.056	$< 2\times10^{-16}$	***
Nonsense	-0.1336	0.2351	-0.568	0.569703	
Read-through	-0.08363	0.3453	-0.242	0.808648	
Synonymous	-0.00207	0.01506	-0.137	0.890677	
Local genetic diversity					
Linear effect	23.06	3.429	6.723	1.78×10^{-11}	***
Quadratic effect	-1191	122.8	-9.703	$< 2\times10^{-16}$	***
Local GC content	2.057	0.1916	10.735	$< 2\times10^{-16}$	***
Proximity (<1kb) to genes of COG category					
J	-0.1296	0.02082	-6.226	4.78×10^{-10}	***
K	0.03783	0.01898	1.993	0.046243	*
L	-0.04477	0.01872	-2.391	0.016804	*
D	-0.02035	0.03504	-0.581	0.561429	
V	0.04061	0.02927	1.387	0.165339	
T	-0.1329	0.02864	-4.64	0.00000348	***
M	-0.03864	0.0193	-2.002	0.045277	*
U	0.08331	0.04003	2.081	0.037422	*
O	0.004029	0.02383	0.169	0.86573	
C	-0.02368	0.01929	-1.227	0.219697	
G	-0.04546	0.01543	-2.946	0.00322	**
E	-0.03347	0.01526	-2.194	0.028244	*
F	-0.0113	0.02277	-0.496	0.619769	
H	-0.006357	0.0206	-0.309	0.757693	
I	-0.006971	0.02493	-0.28	0.779798	
P	-0.01533	0.01726	-0.888	0.374272	
Q	0.01255	0.026	0.483	0.629389	
R	-0.008745	0.01295	-0.675	0.499655	
S	0.003478	0.01486	0.234	0.814885	
BiP identity (cf A→C)					
A→G	0.1027	0.02863	3.586	0.000336	***
A→T	0.02907	0.03069	0.947	0.343643	
C→A	0.02289	0.03678	0.623	0.533607	
C→G	0.06178	0.05538	1.116	0.264604	

	Estimate	Std. Error	*Z* statistic	*p*-value	
C→T	0.09959	0.02841	3.506	0.000455	***
G→A	0.1255	0.02842	4.415	0.0000101	***
G→C	0.07627	0.05382	1.417	0.156472	
G→T	0.05451	0.03634	1.5	0.133641	
T→A	0.02787	0.03061	0.911	0.362538	
T→C	0.08814	0.02864	3.078	0.002085	**
T→G	0.0628	0.03725	1.686	0.091794	.
Local core density	-0.2194	0.04373	-5.017	0.000000524	***
Proximity (<1kb) to genes of type					
CDS	-0.00395	0.0553	-0.071	0.943062	
Miscellaneous RNA	0.04934	0.04821	1.024	0.306058	
rRNA	0.01861	0.1858	0.1	0.920231	
tRNA	-0.2378	0.09245	-2.573	0.010096	*

Significance codes: 0 *** 0.001 ** 0.01 * 0.05 . 0.1

Dispersion parameter for Negative Binomial(3.524) family taken to be 1

† Evidence of similarity to ICE*6013* based on BLAST

Supplementary Table 4 I Hottest and coldest genes by homoplasy rate. Genes were ordered by the *p*-value of a Poisson test for a deviation from the mean homoplasy rate, as described in the Online Methods. The most significantly hot genes are listed first and the most significantly cold genes are listed last. A per-locus binomial test for a deviation from the mean proportion of non-synonymous BiPs is also reported. All genes containing one or more core BiPs are reported.

Gene	Start	End	Product	COG	# Core BiPs	# Homo-plasies	Homoplasy rate	Poisson test: −log10(p)	# Non-synonymous BiPs	Non-synonymous probability	Binomial test: −log10(p)
glnA	1349006	1350346	glutamine synthetase	E	73	279	3.82	71.6	3	0.04	4.1
SAR2297	2381939	2383603	acetolactate synthase	H	84	214	2.55	30.0	17	0.20	0.0
SAR0107	111618	113855	regulatory protein	G	104	241	2.32	27.7	28	0.27	0.7
yycI	28771	29559	hypothetical protein	S	61	167	2.74	26.7	11	0.18	0.2
SAR0311	354310	355842	sodiumsolute symporter family protein	R	61	163	2.67	25.0	9	0.15	0.6
sirC	126920	127918	siderophore ABC transporter permease	P	58	153	2.64	23.1	10	0.17	0.3
clfB	2802106	2804727	fibrinogen and keratin-10 binding surface anchored protein		77	181	2.35	21.8	32	0.42	4.2
yycH	27436	28770	hypothetical protein	S	65	161	2.48	21.5	9	0.14	0.8
SAR0347	390986	391699	hypothetical protein		33	103	3.12	20.8	15	0.45	2.7
SAR0111	119046	120821	myosin-cross-reactive antigen	S	81	181	2.23	19.6	22	0.27	0.7
SAR0310	352985	354205	nucleoside permease	F	72	165	2.29	18.8	14	0.19	0.1
SAR0109	115186	116574	transporter protein	G	58	142	2.45	18.7	11	0.19	0.1
SAR2609	2693304	2693795	acetyltransferase (GNAT) family protein	M	18	69	3.83	18.6	4	0.22	0.0
guaA	444758	446299	GMP synthase	F	53	133	2.51	18.5	4	0.08	2.0
rnr	880732	883104	ribonuclease R	K	128	247	1.93	18.3	16	0.13	1.9
vraE	2888463	2890343	ABC transporter permease		73	164	2.25	18.0	18	0.25	0.3
sasA	2839031	2843086	serine-rich repeat-containing protein		114	223	1.96	17.3	24	0.21	0.0
SAR0144	157911	158684	ABC transporter ATP-binding protein	P	23	76	3.30	16.9	3	0.13	0.3
SAR0312	355882	356763	N-acetylneuraminate lyase	E	27	83	3.07	16.6	2	0.07	1.0
SAR0101	104251	104547	hypothetical protein	K	11	50	4.55	16.5	5	0.45	1.2
guaB	443267	444733	inosine-5'-monophosphate dehydrogenase	F	47	116	2.47	15.8	0	0.00	4.7
aur	2811800	2813329	zinc metalloproteinase aureolysin	E	62	140	2.26	15.7	12	0.19	0.1
ebh	1502887	1535127	hypothetical protein	D	415	603	1.45	14.7	116	0.28	2.7
SAR0303	344607	345914	amino acid transport system	E	61	134	2.20	14.2	12	0.20	0.1
plc	109547	110533	1-phosphatidylinositol phosphodiesterase		48	113	2.35	14.0	11	0.23	0.1
sasH	30932	33292	5'-nucleotidase	F	134	237	1.77	13.6	65	0.49	11.4
SAR2607	2691460	2692260	haloacid dehalogenase-like hydrolase	R	41	100	2.44	13.4	10	0.24	0.2
SAR0600	650888	652210	pyridine nucleotide-disulphide oxidoreductase protein	C	65	137	2.11	13.2	24	0.37	2.4
SAR2769	2872437	2872952	hypothetical protein	S	24	70	2.92	13.1	7	0.29	0.5
SAR0452	478818	480302	NADH dehydrogenase subunit 5	P	55	121	2.20	12.9	15	0.27	0.5
SAR2649	2737187	2738998	hypothetical protein	I	54	119	2.20	12.7	10	0.19	0.1
SAR0196	223636	226425	type I restriction enzyme	V	71	144	2.03	12.6	15	0.21	0.0
SAR2773	2876163	2876627	hypothetical protein		22	65	2.95	12.4	14	0.64	4.6
argC	209357	210391	N-acetyl-gamma-glutamyl-phosphate reductase	E	50	111	2.22	12.1	16	0.32	1.1
SAR2669	2755492	2756556	dihydroorotate dehydrogenase 2	F	27	73	2.70	12.1	7	0.26	0.2
isaB	2813743	2814270	immunodominant antigen B		26	71	2.73	11.9	5	0.19	0.0
lacR	2370769	2371524	lactose phosphotransferase system repressor	G	33	82	2.48	11.5	4	0.12	0.5
SAR0358	405254	406135	hypothetical protein	M	22	62	2.82	11.1	3	0.14	0.2
SAR2619	2703829	2705568	pyruvate oxidase	H	57	118	2.07	11.0	8	0.14	0.7
SAR0449	475651	475845	hypothetical protein		16	50	3.13	10.6	2	0.13	0.3
SAR0348	391724	392566	ABC transporter ATP-binding protein	V	44	97	2.20	10.5	17	0.39	2.0
SAR0188	213486	214043	isochorismatase	Q	38	87	2.29	10.4	17	0.45	2.9

Gene	Start	End	Product	COG	# Core BiPs	# Homo-plasies	Homoplasy rate	Poisson test: −log10(p)	# Non-synonymous BiPs	Non-synonymous probability	Binomial test: −log10(p)
SAR2598	2684721	2685317	phospholipase/carboxylesterase	R	42	93	2.21	10.3	14	0.33	1.1
SAR0108	114006	115184	peptidase	R	50	105	2.10	10.2	17	0.34	1.4
SAR0216	251020	251988	lipoprotein	P	39	88	2.26	10.1	10	0.26	0.3
SAR0302	343532	344356	formate/nitrite transporter	P	11	39	3.55	9.9	1	0.09	0.3
groEL	2176992	2178608	chaperonin GroEL	O	70	133	1.90	9.8	5	0.07	2.7
SAR2296	2381198	2381902	hypothetical protein	Q	46	98	2.13	9.8	8	0.17	0.2
SAR0447	474706	475029	hypothetical protein		18	52	2.89	9.8	8	0.44	1.4
hemY	2002952	2004352	protoporphyrinogen oxidase	H	57	114	2.00	9.8	7	0.12	1.0
SAR2728	2831252	2833642	preprotein translocase subunit SecA	U	73	137	1.88	9.8	26	0.36	2.2
clpL	2712877	2714982	ATP-dependent protease ATP-binding subunit ClpL	O	116	196	1.69	9.8	13	0.11	2.2
aroC	1570935	1572101	chorismate synthase	E	47	98	2.09	9.4	7	0.15	0.4
mqo2	2771755	2773251	malatequinone oxidoreductase	R	33	76	2.30	9.3	3	0.09	1.0
SAR2722	2820206	2823187	hypothetical protein	S	97	168	1.73	9.2	16	0.16	0.6
SAR2487	2561657	2562634	tetrapyrrole (corrin/porphyrin) methylase family protein	H	38	83	2.18	9.0	15	0.39	2.0
SAR1352	1400773	1402761	transketolase	G	45	93	2.07	8.8	11	0.24	0.2
SAR0345	390157	390546	hypothetical protein	R	22	56	2.55	8.5	5	0.23	0.1
SAR2735	2843633	2844325	hypothetical protein	R	39	83	2.13	8.5	5	0.13	0.6
SAR0123	135150	136886	siderophore biosynthesis protein	Q	79	140	1.77	8.5	23	0.29	1.0
SAR0233	273985	275130	flavohemoprotein	C	48	96	2.00	8.4	9	0.19	0.1
SAR1640	1709338	1710156	hypothetical protein	S	21	53	2.52	8.0	3	0.14	0.2
SAR0147	161678	163213	nucleotidase	F	80	139	1.74	7.8	30	0.38	3.0
pcp	2875422	2876060	pyrrolidone-carboxylate peptidase	O	42	85	2.02	7.7	16	0.38	1.9
SAR2729	2833632	2834591	hypothetical protein		27	62	2.30	7.7	13	0.48	2.7
SAR1328	1378275	1379756	cardiolipin synthase	I	61	112	1.84	7.6	12	0.20	0.1
SAR0198	227693	229285	ABC transporter ATP-binding protein	R	67	120	1.79	7.6	20	0.30	1.0
capF	173669	174778	capsular polysaccharide synthesis enzyme	M	55	103	1.87	7.5	17	0.31	1.0
SAR2730	2834569	2836137	hypothetical protein		60	110	1.83	7.4	23	0.38	2.6
narT	2549103	2550272	nitrite transport protein	P	42	84	2.00	7.3	9	0.21	0.0
SAR0012	16096	17064	hydrolase	E	47	91	1.94	7.3	9	0.19	0.1
SAR0342	387093	388805	hypothetical protein	P	55	102	1.85	7.2	19	0.35	1.7
truA	2388216	2389010	tRNA pseudouridine synthase A	J	21	51	2.43	7.2	3	0.14	0.2
dnaC	20752	22152	replicative DNA helicase	L	36	74	2.06	7.0	3	0.08	1.2
SAR0133	146499	147737	hypothetical protein		52	97	1.87	6.9	6	0.12	1.0
SAR0611	659742	661037	phosphohydrolase	R	24	55	2.29	6.8	2	0.08	0.9
SAR0453	480315	483020	hypothetical protein	S	112	176	1.57	6.8	37	0.33	2.4
SAR0170	188750	189709	cation efflux system protein	P	16	42	2.63	6.8	2	0.13	0.3
SAR2576	2657004	2658662	phosphomannomutase	G	80	134	1.68	6.7	32	0.40	3.8
SAR0202	233719	235725	gamma-glutamyltranspeptidase	E	87	143	1.64	6.7	33	0.38	3.3
SAR0277	321758	323281	hypothetical protein		45	86	1.91	6.7	12	0.27	0.4
SAR0341	385883	387112	Sec-independent exported protein	P	63	111	1.76	6.7	10	0.16	0.4
SAR0450	476030	477232	cobalamin synthesis protein	R	62	109	1.76	6.6	10	0.16	0.4
nasE	2562625	2562939	assimilatory nitrite reductase small subunit	R	19	46	2.42	6.5	4	0.21	0.0
lldP1	121907	123499	L-lactate permease 1	C	38	75	1.97	6.4	8	0.21	0.0
SAR2560	2642910	2644448	transport protein	H	58	103	1.78	6.4	9	0.16	0.5
SAR2775	2880717	2882135	sodiumsulfate symporter family protein	P	62	108	1.74	6.3	12	0.19	0.1
SAR2776	2882684	2883592	hypothetical protein	R	42	80	1.90	6.2	11	0.26	0.3
SAR2599	2685336	2686304	dioxygenase	E	50	91	1.82	6.2	14	0.28	0.5
SAR0232	273787	273960	hypothetical protein		7	24	3.43	6.2	0	0.00	0.4
SAR0338	382867	383862	hypothetical protein	S	53	95	1.79	6.2	8	0.15	0.5
uhpT	246978	248357	sugar phosphate antiporter	G	34	68	2.00	6.1	3	0.09	1.0
hisIE	2859014	2859646	bifunctional phosphoribosyl-AMP cyclohydrolase/phosphoribosyl-ATP	E	21	48	2.29	6.1	2	0.10	0.5

Gene	Start	End	Product	COG	# Core BiPs	# Homo-plasies	Homoplasy rate	Poisson test: −log10(p)	# Non-synonymous BiPs	Non-synonymous probability	Binomial test: −log10(p)
			pyrophosphatase protein								
SAR2240	2307711	2308568	haloacid dehalogenase-like hydrolase	R	49	89	1.82	6.0	8	0.16	0.3
rbgA	1271354	1272238	ribosomal biogenesis GTPase	R	27	57	2.11	6.0	3	0.11	0.6
SAR1491	1586852	1587805	hypothetical protein	S	35	69	1.97	6.0	11	0.31	0.8
SAR1253	1314914	1315627	hypothetical protein	K	11	31	2.82	5.9	3	0.27	0.1
SAR0173	191008	191748	ABC transporter ATP-binding protein	P	33	66	2.00	5.9	8	0.24	0.2
SAR0183	207318	208088	amino acid kinase	E	36	70	1.94	5.8	10	0.28	0.4
bglA	307688	309124	6-phospho-beta-glucosidase	G	51	91	1.78	5.8	17	0.33	1.2
glmS	2309650	2311455	glucosamine--fructose-6-phosphate aminotransferase	M	54	95	1.76	5.8	4	0.07	1.9
SAR2791	2894593	2895162	hypothetical protein	K	17	41	2.41	5.8	12	0.71	4.7
SAR2586	2669018	2669710	hypothetical protein	S	16	39	2.44	5.7	2	0.13	0.3
mprF	1427047	1429569	hypothetical protein	S	108	165	1.53	5.7	11	0.10	2.5
metE	397296	399524	5-methyltetrahydropteroyltriglutamate--homocysteine S-methyltransferase	E	73	120	1.64	5.7	12	0.16	0.4
SAR0176	193507	194538	hypothetical protein	I	52	92	1.77	5.7	11	0.21	0.0
fda	2770672	2771562	fructose-1,6-bisphosphate aldolase	G	29	59	2.03	5.6	6	0.21	0.0
SAR2567	2648352	2649047	short chain dehydrogenase	R	27	56	2.07	5.6	6	0.22	0.1
SAR2585	2668122	2668853	MerR family regulatory protein	K	27	56	2.07	5.6	8	0.30	0.5
SAR0121	132157	133911	siderophore biosynthesis protein	Q	71	117	1.65	5.6	22	0.31	1.2
secY	2837691	2838902	preprotein translocase subunit SecY		43	79	1.84	5.6	10	0.23	0.1
SAR2759	2862217	2863230	aminotransferase	E	39	73	1.87	5.5	16	0.41	2.3
scdA	298500	299174	cell wall biosynthesis protein ScdA	D	22	48	2.18	5.5	8	0.36	0.9
yycG	25617	27443	sensor kinase protein	T	49	87	1.78	5.5	1	0.02	3.8
SAR0422	452560	453240	superantigen-like protein		11	30	2.73	5.5	5	0.45	1.2
narH	2556112	2557671	nitrate reductase beta chain	C	80	128	1.60	5.5	6	0.08	2.8
capB	169342	170028	capsular polysaccharide synthesis enzyme	D	28	57	2.04	5.4	3	0.11	0.6
hisA	2860398	2861102	1-(5-phosphoribosyl)-5-[(5-phosphoribosylamino)methylideneamino] imidazole-4-carboxamide isomerase	E	28	57	2.04	5.4	8	0.29	0.4
SAR2779	2885811	2886611	N-acetyltransferase	Q	26	54	2.08	5.4	8	0.31	0.6
SAR2727	2829726	2831234	hypothetical protein	M	42	77	1.83	5.4	17	0.40	2.3
SAR0355	401331	402491	Cys/Met metabolism PLP-dependent enzyme	E	27	55	2.04	5.4	4	0.15	0.3
SAR1404	1461145	1462746	ABC transporter ATP-binding protein	R	45	81	1.80	5.3	1	0.02	3.4
mvaA	2709121	2710401	3-hydroxy-3-methylglutaryl-coenzyme A reductase	I	41	75	1.83	5.3	7	0.17	0.2
sirA	128926	129918	lipoprotein	P	18	41	2.28	5.2	3	0.17	0.1
SAR0455	483773	484120	hypothetical protein		11	29	2.64	5.1	5	0.45	1.2
SAR2601	2687033	2687326	acetyltransferase (GNAT) family protein	R	15	36	2.40	5.0	4	0.27	0.3
SAR0007	9753	10583	hypothetical protein	G	34	64	1.88	5.0	2	0.06	1.7
SAR0215	249467	251023	sensor kinase protein	T	53	90	1.70	5.0	11	0.21	0.0
hisH	2861095	2861673	imidazole glycerol phosphate synthase subunit HisH	E	32	61	1.91	5.0	8	0.25	0.2
SAR0354	399521	401362	bifunctional homocysteine S-methyltransferase/5,10-methylenetetrahydrofolate reductase protein	E	51	87	1.71	4.9	11	0.22	0.0
yycJ	29947	30747	metallo-beta-lactamase superfamily protein	R	42	75	1.79	4.9	1	0.02	3.0
SAR0320	366246	367247	luciferase family protein	C	46	80	1.74	4.8	12	0.26	0.3
clfA	888239	891328	clumping factor		26	52	2.00	4.8	7	0.27	0.3
SAR0222	258386	260215	staphylocoagulase precursor		15	35	2.33	4.8	7	0.47	1.6
aroB	1569845	1570909	3-dehydroquinate synthase	E	28	54	1.93	4.6	7	0.25	0.2
SAR2663	2750353	2751699	hypothetical protein	P	55	91	1.65	4.6	15	0.27	0.5

Gene	Start	End	Product	COG	# Core BiPs	# Homo-plasies	Homoplasy rate	Poisson test: −log10(p)	# Non-synonymous BiPs	Non-synonymous probability	Binomial test: −log10(p)
SAR1326	1376726	1377751	hypothetical protein	E	68	108	1.59	4.6	14	0.21	0.0
SAR0129	142399	143175	acetoin reductase	R	29	55	1.90	4.5	2	0.07	1.2
mnmA	1763791	1764909	tRNA-specific 2-thiouridylase MnmA	J	36	65	1.81	4.5	6	0.17	0.2
SAR2659	2746554	2747258	short chain dehydrogenase	R	39	69	1.77	4.4	4	0.10	0.9
SAR0205	238058	239155	ABC transporter ATP-binding protein	G	30	56	1.87	4.3	5	0.17	0.2
SAR2661	2748386	2749216	hydrolase	R	23	46	2.00	4.3	12	0.52	2.9
SAR2549	2631029	2632222	transport protein	G	33	60	1.82	4.3	3	0.09	1.0
SAR0014	18310	20277	hypothetical protein	T	56	91	1.63	4.3	6	0.11	1.3
ldh2	2765369	2766328	L-lactate dehydrogenase 2	C	20	41	2.05	4.2	0	0.00	1.9
SAR2627	2711993	2712514	6-O-methylguanine DNA methyltransferase	L	24	47	1.96	4.1	13	0.54	3.4
SAR2664	2751687	2752793	hypothetical protein	O	35	62	1.77	4.1	9	0.26	0.3
SAR1877	1963638	1965116	AMP-binding enzyme	Q	90	133	1.48	4.0	36	0.40	4.1
SAR0313	356921	357781	ROK family protein	G	30	55	1.83	4.0	5	0.17	0.2
SAR0211	244924	245892	hypothetical protein	G	26	49	1.88	4.0	7	0.27	0.3
SAR0335	381166	382227	luciferase-like monooxygenase	C	36	63	1.75	4.0	13	0.36	1.4
SAR0210	243829	244869	oxidoreductase	R	46	76	1.65	3.9	8	0.17	0.2
SAR2279	2362898	2363767	hypothetical protein	R	37	64	1.73	3.9	7	0.19	0.1
SAR0454	483182	483544	hypothetical protein	S	8	21	2.63	3.8	1	0.13	0.0
agrC	2184799	2186091	autoinducer sensor protein	T	30	54	1.80	3.8	3	0.10	0.7
SAR0939	983195	984064	LysR family regulatory protein	K	25	47	1.88	3.8	7	0.28	0.3
SAR2559	2641857	2642675	short chain dehydrogenase	R	32	57	1.78	3.8	8	0.25	0.2
SAR0331	376571	377926	hypothetical protein	V	45	74	1.64	3.8	12	0.27	0.4
SAR0448	475048	475362	hypothetical protein		13	29	2.23	3.8	4	0.31	0.3
SAR0221	257852	258196	hypothetical protein		17	35	2.06	3.7	10	0.59	3.1
SAR1488	1582499	1583485	pyridine nucleotide-disulphide oxidoreductase	O	31	55	1.77	3.7	4	0.13	0.4
fadE	265787	267292	acyl-CoA synthetase	Q	59	92	1.56	3.7	11	0.19	0.1
SAR0346	390786	390989	DNA-binding protein	K	10	24	2.40	3.7	1	0.10	0.2
priA	1235259	1237667	primosomal protein n'	L	67	102	1.52	3.6	23	0.34	1.8
SAR2630	2715235	2717229	ferrous iron transport protein B	P	77	114	1.48	3.6	25	0.32	1.6
SAR0324	369326	370348	lipoate-protein ligase A	H	36	61	1.69	3.5	18	0.50	3.9
est	879958	880698	carboxylesterase	R	36	61	1.69	3.5	8	0.22	0.1
lacB	2369443	2369958	galactose-6-phosphate isomerase subunit LacB	G	16	33	2.06	3.5	3	0.19	0.0
SAR0182	206554	207048	hypothetical protein	S	23	43	1.87	3.5	6	0.26	0.2
SAR0169	186616	188103	aldehyde dehydrogenase	C	45	73	1.62	3.5	7	0.16	0.3
SAR2481	2553344	2554378	histidine kinase	T	49	78	1.59	3.5	2	0.04	2.9
glcA	216022	218067	glucose-specific PTS transporter protein, IIABC component	G	56	87	1.55	3.4	9	0.16	0.4
groES	2178684	2178968	co-chaperonin GroES	O	4	13	3.25	3.4	0	0.00	0.2
SAR0602	653261	654655	hypothetical protein		41	67	1.63	3.4	15	0.37	1.6
SAR0214	248716	249474	response regulator	T	19	37	1.95	3.4	5	0.26	0.2
SAR2423	2492529	2493182	hypothetical protein	S	24	44	1.83	3.4	10	0.42	1.6
SAR0263	306881	307672	PTS transporter	G	27	48	1.78	3.4	8	0.30	0.5
SAR0236	278870	279805	inosine-uridine preferring nucleoside hydrolase	F	35	59	1.69	3.4	9	0.26	0.3
SAR0104	108860	109342	hypothetical protein		32	55	1.72	3.3	20	0.63	6.2
SAR0143	157109	157909	binding-protein-dependent transport system membrane component	P	15	31	2.07	3.3	3	0.20	0.0
SAR0999	1045915	1046517	hypothetical protein		10	23	2.30	3.3	2	0.20	0.0
SAR0436	465245	466732	hypothetical protein		13	28	2.15	3.3	2	0.15	0.0
SAR2597	2683537	2684616	hypothetical protein	S	39	64	1.64	3.3	7	0.18	0.2
SAR2700	2792901	2794901	hypothetical protein	M	85	122	1.44	3.3	29	0.34	2.1
SAR0359	406165	406368	hypothetical protein	S	7	18	2.57	3.3	1	0.14	0.0

Gene	Start	End	Product	COG	# Core BiPs	# Homo-plasies	Homoplasy rate	Poisson test: −log10(p)	# Non-synonymous BiPs	Non-synonymous probability	Binomial test: −log10(p)
SAR2290	2373980	2374828	aldo/keto reductase family protein	R	36	60	1.67	3.3	11	0.31	0.7
lytR	301182	301922	two-component response regulator	T	20	38	1.90	3.3	2	0.10	0.5
SAR2460	2531321	2531722	acetyltransferase (GNAT) family protein	R	16	32	2.00	3.1	6	0.38	0.9
SAR2521	2596782	2597168	hypothetical protein	S	16	32	2.00	3.1	6	0.38	0.9
lytM	317932	318882	peptidoglycan hydrolase	R	38	62	1.63	3.1	13	0.34	1.1
SAR2726	2828375	2829733	hypothetical protein		42	67	1.60	3.1	16	0.38	1.9
SAR1282	1347137	1348375	hypothetical protein	P	46	72	1.57	3.1	7	0.15	0.4
lacG	2364033	2365445	6-phospho-beta-galactosidase	G	46	72	1.57	3.1	5	0.11	1.0
isdA	1149036	1150100	iron-regulated heme-iron binding protein	M	30	51	1.70	3.1	9	0.30	0.6
SAR1930	2009937	2010302	hypothetical protein	R	8	19	2.38	2.9	0	0.00	0.7
SAR2742	2847634	2848134	acetyltransferase (GNAT) family protein	R	16	31	1.94	2.9	8	0.50	2.0
SAR0420	450865	451224	hypothetical protein	S	20	37	1.85	2.9	8	0.40	1.3
SAR0576	630788	631435	haloacid dehalogenase-like hydrolase	R	26	45	1.73	2.9	5	0.19	0.0
SAR2632	2717683	2720172	transport protein	R	90	125	1.39	2.8	19	0.21	0.0
SAR0124	136867	138645	siderophore biosynthesis protein	Q	56	83	1.48	2.8	22	0.39	2.6
SAR0394	428909	429490	phosphoglycerate mutase family protein	G	17	32	1.88	2.8	5	0.29	0.4
SAR0132	145352	146518	galactosyl transferase	M	48	73	1.52	2.8	13	0.27	0.4
SAR1227	1281260	1282567	tRNA (uracil-5-)-methyltransferase Gid	J	38	60	1.58	2.8	3	0.08	1.3
SAR2502	2574532	2575263	ABC transporter ATP-binding protein	E	31	51	1.65	2.7	4	0.13	0.4
SAR0220	255925	257688	hypothetical protein	C	61	89	1.46	2.7	15	0.25	0.3
SAR2678	2763441	2764301	2-dehydropantoate 2-reductase	H	35	56	1.60	2.7	14	0.40	1.9
narI	2554874	2555551	nitrate reductase gamma chain	C	28	47	1.68	2.7	2	0.07	1.2
SAR1127	1175227	1175544	hypothetical protein		7	17	2.43	2.7	1	0.14	0.0
nrdG	2783529	2784065	anaerobic ribonucleotide reductase activating protein	O	13	26	2.00	2.6	3	0.23	0.1
SAR2345	2416600	2419767	AcrB/AcrD/AcrF family protein	V	91	125	1.37	2.6	26	0.29	1.0
zwf	1652481	1653965	glucose-6-phosphate 1-dehydrogenase	G	29	48	1.66	2.6	2	0.07	1.2
SAR2444	2513540	2514508	hypothetical protein	S	24	41	1.71	2.6	9	0.38	1.1
SAR2674	2759510	2761192	hypothetical protein	R	24	41	1.71	2.6	11	0.46	2.0
SAR0622	670487	670993	hypothetical protein		8	18	2.25	2.6	5	0.63	1.9
SAR0356	402488	403591	Cys/Met metabolism PLP-dependent enzyme	E	45	68	1.51	2.6	7	0.16	0.3
SAR0505	543915	545441	polysaccharide biosynthesis protein	R	37	58	1.57	2.6	13	0.35	1.2
SAR2568	2649346	2649627	hypothetical protein		5	13	2.60	2.6	1	0.20	0.0
SAR0928	964308	967784	hypothetical protein	L	136	177	1.30	2.6	42	0.31	2.1
SAR2622	2706899	2707777	LysR family regulatory protein	K	16	30	1.88	2.6	2	0.13	0.3
hisG	2864489	2865103	ATP phosphoribosyltransferase catalytic subunit	E	30	49	1.63	2.5	10	0.33	0.9
SAR2503	2575260	2575979	transport system membrane protein	E	34	54	1.59	2.5	4	0.12	0.7
SAR0139	152865	154217	transporter		38	59	1.55	2.5	4	0.11	0.9
smpB	883126	883590	SsrA-binding protein	O	22	38	1.73	2.5	3	0.14	0.2
narG	2557661	2561350	nitrate reductase alpha chain	C	161	205	1.27	2.4	21	0.13	2.0
malR	1656809	1657828	maltose operon transcriptional repressor	K	30	48	1.60	2.4	9	0.30	0.6
SAR1597	1671119	1672798	DNA repair protein	L	36	56	1.56	2.4	6	0.17	0.2
sasD	150393	151058	surface anchored protein		1	5	5.00	2.4	1	1.00	0.7
SAR0401	436077	437465	sodiumdicarboxylate symporter protein	R	41	62	1.51	2.4	10	0.24	0.2
SAR0451	477501	477629	hypothetical protein		9	19	2.11	2.4	8	0.89	4.5
SAR0601	652197	652637	hypothetical protein	K	19	33	1.74	2.3	8	0.42	1.4
SAR0251	291714	292883	teichoic acid biosynthesis protein	M	35	54	1.54	2.3	9	0.26	0.3
SAR1469	1563329	1564471	glycosyl transferase	M	35	54	1.54	2.3	8	0.23	0.1
srrB	1640667	1642433	sensor kinase protein	T	54	78	1.44	2.3	8	0.15	0.5
miaA	1343887	1344822	tRNA delta(2)-isopentenylpyrophosphate transferase	J	21	36	1.71	2.3	9	0.43	1.5
csdB	915853	917094	selenocysteine lyase	E	32	50	1.56	2.3	4	0.13	0.5

Gene	Start	End	Product	COG	# Core BiPs	# Homo-plasies	Homoplasy rate	Poisson test: −log10(p)	# Non-synonymous BiPs	Non-synonymous probability	Binomial test: −log10(p)
bfmBAB	1667556	1668539	2-oxoisovalerate dehydrogenase beta subunit	C	36	55	1.53	2.3	5	0.14	0.5
asp23	2356967	2357476	alkaline shock protein 23	S	14	26	1.86	2.3	1	0.07	0.5
SAR2739	2846021	2846380	hypothetical protein		20	34	1.70	2.2	7	0.35	0.8
SAR0193	220615	222069	sucrose-specific PTS tranporter protein	G	40	60	1.50	2.2	6	0.15	0.4
SAR2696	2786159	2787529	transporter protein	C	89	119	1.34	2.2	17	0.19	0.2
SAR2723	2823398	2825257	N-acetylmuramoyl-L-alanine amidase	U	53	76	1.43	2.2	22	0.42	3.0
SAR2720	2817190	2819142	PTS transport system, IIABC component	G	90	120	1.33	2.2	29	0.32	1.7
SAR0343	389116	389817	Sec-independent protein translocase protein	U	15	27	1.80	2.2	6	0.40	1.0
uppS	1290667	1291437	undecaprenyl pyrophosphate synthase	I	10	20	2.00	2.2	2	0.20	0.0
panD	2761321	2761704	aspartate alpha-decarboxylase	H	10	20	2.00	2.2	2	0.20	0.0
vraD	2887715	2888473	ABC transporter ATP-binding protein	V	10	20	2.00	2.2	3	0.30	0.3
SAR0242	283219	284478	PTS transport system, IIC component	G	27	43	1.59	2.1	6	0.22	0.1
SAR1471	1565368	1566066	hypothetical protein	R	9	18	2.00	2.1	1	0.11	0.2
SAR2655	2744340	2744717	glyoxalase	R	9	18	2.00	2.1	1	0.11	0.2
SAR1273a	1338092	1338190	hypothetical protein		7	15	2.14	2.1	5	0.71	2.2
SAR2780	2886913	2887425	hypothetical protein	S	22	36	1.64	2.1	5	0.23	0.1
SAR0011	15405	15734	hypothetical protein	S	13	24	1.85	2.1	5	0.38	0.8
SAR2795	2896726	2897565	DNA-binding protein	K	13	24	1.85	2.1	3	0.23	0.1
femA	1444550	1445812	factor essential for expression of methicillin resistance	V	28	44	1.57	2.1	1	0.04	1.7
SAR0457	484938	485672	hypothetical protein	R	19	32	1.68	2.1	7	0.37	0.8
fadA	260802	261986	thiolase	I	33	50	1.52	2.0	10	0.30	0.7
opp-1C	2633788	2634657	oligopeptide transporter membrane permease	P	25	40	1.60	2.0	3	0.12	0.5
SAR1238	1292438	1293724	hypothetical protein	M	38	56	1.47	2.0	2	0.05	1.8
SAR0421	451243	452088	hypothetical protein	G	34	51	1.50	2.0	10	0.29	0.5
SAR1870	1957638	1958831	S-adenosylmethionine synthetase	H	34	51	1.50	2.0	4	0.12	0.7
cudA	2778913	2780403	betaine aldehyde dehydrogenase	C	52	73	1.40	1.9	7	0.13	0.7
SAR0208	241712	242551	sugar ABC transporter permease	G	17	29	1.71	1.9	4	0.24	0.1
SAR1436	1495212	1495640	methionine sulfoxide reductase B	O	14	25	1.79	1.9	0	0.00	1.3
SAR1446	1502427	1502828	hypothetical protein	L	14	25	1.79	1.9	5	0.36	0.7
ulaA	371783	373129	PTS system ascorbate-specific transporter subunit IIC	S	44	63	1.43	1.9	10	0.23	0.1
fabI	1022267	1023037	enoyl-(acyl carrier protein) reductase	I	8	16	2.00	1.9	2	0.25	0.2
SAR1860	1950015	1950458	hypothetical protein		8	16	2.00	1.9	1	0.13	0.0
SAR2636	2723784	2723984	hypothetical protein		6	13	2.17	1.9	2	0.33	0.2
SAR0268	311992	312873	sugar transport protein	G	29	44	1.52	1.9	8	0.28	0.4
SAR2589	2672796	2674073	transporter protein	G	29	44	1.52	1.9	5	0.17	0.1
opp-1B	2634654	2635589	oligopeptide transporter membrane permease	P	25	39	1.56	1.9	3	0.12	0.5
SAR2635	2722955	2723554	acetyltransferase	R	25	39	1.56	1.9	4	0.16	0.2
mvaK1	647321	648241	mevalonate kinase	I	13	23	1.77	1.9	1	0.08	0.5
ebpS	1583870	1585330	cell surface elastin binding protein		41	59	1.44	1.9	23	0.56	5.9
lacD	2367514	2368494	tagatose 1,6-diphosphate aldolase	G	61	83	1.36	1.9	7	0.11	1.2
msrA2	1495633	1496166	methionine sulfoxide reductase A	O	3	8	2.67	1.8	0	0.00	0.0
mtlA	2312136	2313674	PTS system, mannitol-specific IIBC component	G	82	108	1.32	1.8	11	0.13	1.1
SAR0267	311573	311977	D-ribose pyranase	G	12	22	1.83	1.8	3	0.25	0.1
SAR0128	141562	142188	hypothetical protein		15	26	1.73	1.8	13	0.87	6.9
otc	1187532	1188533	ornithine carbamoyltransferase	E	15	26	1.73	1.8	3	0.20	0.0
purA	22430	23713	adenylosuccinate synthetase	F	42	60	1.43	1.8	1	0.02	3.0
mvaD	648246	649229	mevalonate diphosphate decarboxylase	I	30	45	1.50	1.8	8	0.27	0.3

Gene	Start	End	Product	COG	# Core BiPs	# Homo-plasies	Homoplasy rate	Poisson test: −log10(p)	# Non-synonymous BiPs	Non-synonymous probability	Binomial test: −log10(p)
lysC	1463603	1464808	aspartate kinase	E	30	45	1.50	1.8	11	0.37	1.2
SAR0403	438584	439540	hypothetical protein		33	49	1.48	1.8	18	0.55	4.5
SAR2660	2747356	2748366	hypothetical protein	R	33	49	1.48	1.8	10	0.30	0.7
SAR0199	229413	230720	ABC transporter permease	P	38	55	1.45	1.8	15	0.39	2.0
lacE	2365463	2367175	PTS system, lactose-specific IIBC component	G	46	65	1.41	1.8	6	0.13	0.7
prs	537768	538733	ribose-phosphate pyrophosphokinase	F	11	20	1.82	1.8	0	0.00	0.9
opp-1F	2632234	2632983	oligopeptide transporter ATPase subunit	P	19	31	1.63	1.8	6	0.32	0.6
trpG	1439316	1439882	anthranilate synthase component II	H	14	24	1.71	1.8	2	0.14	0.1
SAR2547	2628283	2629860	hypothetical protein	E	44	62	1.41	1.8	6	0.14	0.6
SAR2652	2742460	2743029	TetR family regulatory protein	K	23	36	1.57	1.8	7	0.30	0.5
gntK	2665677	2667230	gluconokinase	G	59	80	1.36	1.8	24	0.41	3.1
SAR2771	2874313	2875269	hypothetical protein	R	39	56	1.44	1.8	16	0.41	2.3
sucA	1483038	1485836	2-oxoglutarate dehydrogenase E1 component	C	81	106	1.31	1.7	7	0.09	2.4
SAR1420	1477250	1479136	hypothetical protein	P	45	63	1.40	1.7	16	0.36	1.6
rsbV	2224007	2224333	anti-sigma B factor antagonist	T	2	6	3.00	1.7	0	0.00	0.0
sasC	1924195	1930764	surface anchored protein	M	236	281	1.19	1.7	87	0.37	7.2
SAR0976	1018750	1020135	divalent cation transport protein	P	35	51	1.46	1.7	4	0.11	0.7
fadX	267318	268880	acetyl-CoA transferase	I	61	82	1.34	1.7	24	0.39	2.8
SAR0248	289050	290744	hypothetical protein	M	9	17	1.89	1.7	3	0.33	0.4
phoR	1837647	1839311	alkaline phosphatase synthesis sensor protein	T	46	64	1.39	1.7	7	0.15	0.4
SAR1836	1917999	1919408	dipeptidase PepV	E	41	58	1.41	1.7	9	0.22	0.1
SAR0419	450752	450871	hypothetical protein		1	4	4.00	1.7	1	1.00	0.7
SAR0901	936769	937005	hypothetical protein	S	1	4	4.00	1.7	0	0.00	0.0
SAR1692	1754062	1754985	peptidase	O	15	25	1.67	1.7	2	0.13	0.1
SAR2404	2473371	2474006	haloacid dehalogenase-like hydrolase	R	15	25	1.67	1.7	4	0.27	0.3
SAR2594	2679257	2679952	ABC transporter ATP-binding protein	V	29	43	1.48	1.7	6	0.21	0.0
ubiE	1574072	1574797	ubiquinone/menaquinone biosynthesis methyltransferase	H	17	28	1.65	1.7	4	0.24	0.1
SAR0186	210427	211611	ornithine aminotransferase	E	57	77	1.35	1.7	17	0.30	0.8
SAR0888	926696	927079	hypothetical protein	S	7	14	2.00	1.7	1	0.14	0.0
SAR0265	309619	310380	hypothetical protein	H	34	49	1.44	1.6	8	0.24	0.1
drm	154988	156166	phosphopentomutase	G	21	33	1.57	1.6	1	0.05	1.2
argJ	208104	209345	bifunctional ornithine acetyltransferase/N-acetylglutamate synthase protein	E	69	91	1.32	1.6	18	0.26	0.4
cbiO	2390668	2391477	cobalt transporter ATP-binding subunit		35	50	1.43	1.6	16	0.46	2.9
trpS	1007784	1008773	tryptophanyl-tRNA synthetase	J	66	87	1.32	1.6	7	0.11	1.5
SAR0206	239168	240439	extracellular sugar-binding lipoprotein	G	31	45	1.45	1.6	6	0.19	0.0
SAR0458	486126	487466	sodiumneurotransmitter symporter family protein	R	31	45	1.45	1.6	3	0.10	0.9
icaC	2854437	2855489	intercellular adhesion protein C	G	31	45	1.45	1.6	5	0.16	0.2
SAR2400	2470876	2472255	amino acid permease	E	39	55	1.41	1.6	3	0.08	1.5
SAR0145	158898	159854	lipoprotein	P	20	31	1.55	1.6	4	0.20	0.0
narJ	2555544	2556119	respiratory nitrate reductase delta chain	C	20	31	1.55	1.6	1	0.05	1.0
gtaB	2659791	2660657	UTP--glucose-1-phosphate uridylyltransferase	M	20	31	1.55	1.6	1	0.05	1.0
SAR2545	2626346	2627422	hypothetical protein	G	40	56	1.40	1.5	11	0.28	0.5
SAR0120	131126	132136	ornithine cyclodeaminase	E	32	46	1.44	1.5	3	0.09	0.9
argG	959240	960445	argininosuccinate synthase	E	32	46	1.44	1.5	7	0.22	0.0
SAR0305	347637	348686	hypothetical protein	M	56	75	1.34	1.5	13	0.23	0.1
rpoB	590831	594382	DNA-directed RNA polymerase subunit beta	K	56	75	1.34	1.5	4	0.07	2.1

Gene	Start	End	Product	COG	# Core BiPs	# Homo-plasies	Homoplasy rate	Poisson test: −log10(p)	# Non-synonymous BiPs	Non-synonymous probability	Binomial test: −log10(p)
SAR0024	33660	34139	rRNA large subunit methyltransferase	S	21	32	1.52	1.5	3	0.14	0.2
SAR0530	578226	579299	hypothetical protein	R	21	32	1.52	1.5	3	0.14	0.2
SAR2588	2670496	2672319	hypothetical protein	S	77	99	1.29	1.5	24	0.31	1.3
tcaA	2511268	2512650	teicoplanin resistance associated membrane protein	S	29	42	1.45	1.5	10	0.34	1.0
hisF	2859643	2860401	imidazole glycerol phosphate synthase subunit HisF	E	29	42	1.45	1.5	9	0.31	0.6
SAR2703	2796659	2797324	response regulator protein	T	11	19	1.73	1.5	3	0.27	0.1
SAR0099	102706	103692	hypothetical protein	J	22	33	1.50	1.4	3	0.14	0.2
SAR1252	1312540	1314909	DNA translocase (FtsK/SpoIIIE family protein)	D	50	67	1.34	1.4	12	0.24	0.2
SAR0316	359792	361105	hypothetical protein	S	35	49	1.40	1.4	11	0.31	0.8
SAR0457a	485874	485963	hypothetical protein		3	7	2.33	1.4	3	1.00	2.0
SAR0180	198393	205568	non-ribosomal peptide synthetase	Q	264	307	1.16	1.4	112	0.42	13.6
metG	528410	530383	methionyl-tRNA synthetase	J	45	61	1.36	1.4	13	0.29	0.7
SAR1348	1398035	1398898	hypothetical protein	S	36	50	1.39	1.4	17	0.47	3.2
gnd	1661312	1662718	6-phosphogluconate dehydrogenase	G	19	29	1.53	1.4	0	0.00	1.7
sirB	127915	128910	siderophore ABC transporter permease	P	37	51	1.38	1.4	9	0.24	0.2
glcB	2701463	2703529	PTS system, glucose-specific IIABC component	G	47	63	1.34	1.4	7	0.15	0.4
SAR2131	2192382	2192606	hypothetical protein	O	12	20	1.67	1.4	5	0.42	0.8
gntR	2667255	2667935	gluconate operon transcriptional repressor	K	20	30	1.50	1.3	7	0.35	0.8
SAR1464	1555818	1556504	hypothetical protein	L	16	25	1.56	1.3	2	0.13	0.3
rot	1937051	1937551	repressor of toxins		16	25	1.56	1.3	4	0.25	0.1
SAR2482	2554402	2554854	hypothetical protein	T	16	25	1.56	1.3	2	0.13	0.3
uppP	769790	770665	undecaprenyl pyrophosphate phosphatase	V	28	40	1.43	1.3	5	0.18	0.1
hutH	10891	12405	histidine ammonia-lyase	E	43	58	1.35	1.3	15	0.35	1.4
SAR0269	313105	314103	LacI family regulatory protein	K	50	66	1.32	1.3	15	0.30	0.8
SAR0010	14713	15408	hypothetical protein	E	34	47	1.38	1.3	5	0.15	0.4
SAR0916	950580	951173	cyclophilin type peptidyl-prolyl cis-trans isomerase	O	13	21	1.62	1.3	1	0.08	0.5
ftsZ	1207308	1208480	cell division protein FtsZ	D	8	14	1.75	1.3	0	0.00	0.7
fabF	989991	991235	3-oxoacyl-[acyl-carrier-protein] synthase II	Q	29	41	1.41	1.3	7	0.24	0.2
adhA	662085	663095	alcohol dehydrogenase	R	17	26	1.53	1.3	3	0.18	0.0
SAR0981	1026371	1027129	hypothetical protein	P	17	26	1.53	1.3	4	0.24	0.1
hlgC	2584636	2585583	gamma-hemolysin component C precursor		17	26	1.53	1.3	3	0.18	0.0
SAR0271	315004	316380	transport protein		52	68	1.31	1.3	15	0.29	0.6
SAR0557	607311	608948	ribulokinase	C	52	68	1.31	1.3	15	0.29	0.6
dnaG	1707478	1709277	DNA primase	L	53	69	1.30	1.2	15	0.28	0.6
SAR0315	358998	359666	N-acetylmannosamine-6-phosphate 2-epimerase	G	27	38	1.41	1.2	10	0.37	1.2
SAR0418	450191	450352	hypothetical protein		10	17	1.70	1.2	6	0.60	2.0
tdcB	1538429	1539469	threonine dehydratase	E	36	49	1.36	1.2	2	0.06	1.8
SAR0278	323611	324513	hypothetical protein	R	2	5	2.50	1.2	1	0.50	0.4
SAR0435	464528	465226	superantigen-like protein		2	5	2.50	1.2	0	0.00	0.0
rplO	2398390	2398830	50S ribosomal protein L15	J	2	5	2.50	1.2	0	0.00	0.0
geh	361522	363597	lipase precursor	R	88	109	1.24	1.2	44	0.50	8.4
proS	1293744	1295447	prolyl-tRNA synthetase	J	54	70	1.30	1.2	8	0.15	0.5
SAR0389	426413	426781	hypothetical protein	S	18	27	1.50	1.2	10	0.56	2.8
SAR1187	1234060	1235259	flavoprotein	H	28	39	1.39	1.2	1	0.04	1.7
SAR2653	2743227	2743727	hypothetical protein		28	39	1.39	1.2	23	0.82	10.9
SAR0314	358058	358858	hypothetical protein	K	23	33	1.43	1.2	3	0.13	0.3
def	1111197	1111748	peptide deformylase	J	23	33	1.43	1.2	1	0.04	1.4
SAR1248	1306554	1307525	riboflavin biosynthesis protein	H	23	33	1.43	1.2	5	0.22	0.0

Gene	Start	End	Product	COG	# Core BiPs	# Homo-plasies	Homoplasy rate	Poisson test: −log10(p)	# Non-synonymous BiPs	Non-synonymous probability	Binomial test: −log10(p)
SAR2745	2849842	2850504	capsule synthesis protein	M	23	33	1.43	1.2	2	0.09	0.7
SAR0146	160083	161627	hypothetical protein		48	63	1.31	1.2	20	0.42	2.7
SAR0179	196696	197946	transporter protein		48	63	1.31	1.2	6	0.13	0.8
asd	1464872	1465861	aspartate semialdehyde dehydrogenase	E	38	51	1.34	1.2	10	0.26	0.4
SAR0244	285553	285699	hypothetical protein		6	11	1.83	1.2	1	0.17	0.0
mutL	1335498	1337507	DNA mismatch repair protein	L	57	73	1.28	1.2	12	0.21	0.0
SAR0595	645898	646734	lipoate-protein ligase A protein	H	19	28	1.47	1.2	2	0.11	0.4
SAR0793	830945	831463	hypothetical protein	S	19	28	1.47	1.2	7	0.37	0.8
SAR0181	205581	206225	4'-phosphopantetheinyl transferase superfamily protein	H	15	23	1.53	1.1	4	0.27	0.3
SAR1586	1658022	1658393	glyoxalase/bleomycin resistance protein/dioxygenase superfamily protein	E	15	23	1.53	1.1	8	0.53	2.2
SAR0507	546631	546894	S4 domain-containing protein	J	4	8	2.00	1.1	1	0.25	0.0
SARs013	1463346	1463521	NA		4	8	2.00	1.1	0	0.00	0.2
mvaK2	649242	650318	phosphomevalonate kinase	I	13	20	1.54	1.1	1	0.08	0.5
SAR1579	1650328	1651143	pyrroline-5-carboxylate reductase	E	13	20	1.54	1.1	1	0.08	0.5
SAR2793	2895878	2896231	hypothetical protein		13	20	1.54	1.1	2	0.15	0.0
SAR2548	2630056	2630805	hypothetical protein		20	29	1.45	1.1	8	0.40	1.3
bfmBC	1669547	1670968	dihydrolipoamide dehydrogenase	C	35	47	1.34	1.1	12	0.34	1.0
asnC	1556833	1558125	asparaginyl-tRNA synthetase	J	42	55	1.31	1.1	9	0.21	0.0
SAR2682	2768542	2769885	4-aminobutyrate aminotransferase	E	46	60	1.30	1.1	18	0.39	2.2
SAR1961	2053180	2054697	hypothetical protein	G	36	48	1.33	1.1	10	0.28	0.4
SAR0393	428522	428731	hypothetical protein		10	16	1.60	1.1	8	0.80	3.9
SAR2611	2695507	2696187	L-serine dehydratase, beta chain	E	10	16	1.60	1.1	3	0.30	0.3
SAR2623	2707944	2708162	hypothetical protein		1	3	3.00	1.1	1	1.00	0.7
femB	1445831	1447090	methicillin resistance expression factor	V	30	41	1.37	1.1	2	0.07	1.3
SAR0308	350958	352076	PfkB family carbohydrate kinase	G	14	21	1.50	1.1	3	0.21	0.0
mvaS	2710656	2711822	3-hydroxy-3-methylglutaryl coenzyme A synthase	I	37	49	1.32	1.1	5	0.14	0.5
SAR0761	796705	797145	hypothetical protein		7	12	1.71	1.0	1	0.14	0.0
SAR2657	2745574	2745873	hypothetical protein	S	7	12	1.71	1.0	4	0.57	1.4
SAR0207	240442	241710	sugar ABC transporter permease	G	49	63	1.29	1.0	6	0.12	0.8
SAR2647	2735390	2735887	hypothetical protein		22	31	1.41	1.0	9	0.41	1.4
SAR0131	144453	145142	sugar transferase	M	17	25	1.47	1.0	2	0.12	0.3
hisB	2861670	2862248	imidazoleglycerol-phosphate dehydratase	E	28	38	1.36	1.0	7	0.25	0.2
purM	1090697	1091725	phosphoribosylaminoimidazole synthetase	F	46	59	1.28	1.0	13	0.28	0.6
SAR1002	1047861	1049825	hypothetical protein	S	79	97	1.23	1.0	46	0.58	11.9
SAR0459	487683	488588	pyridoxal-phosphate dependent enzyme	E	32	43	1.34	1.0	13	0.41	1.8
SAR0856	900289	900870	phosphoglycerate mutase family protein	G	15	22	1.47	1.0	2	0.13	0.1
SAR0730	766363	766752	hypothetical protein		23	32	1.39	1.0	10	0.43	1.7
isaA	2739685	2740386	immunodominant antigen A		11	17	1.55	1.0	7	0.64	2.5
arcC	2805880	2806821	carbamate kinase	E	18	26	1.44	1.0	9	0.50	2.2
SAR1844	1934127	1935308	hypothetical protein	G	30	40	1.33	1.0	3	0.10	0.7
fadD	264464	265675	acyl-CoA dehydrogenase	I	34	45	1.32	1.0	11	0.32	0.9
fadB	262016	264277	fatty oxidation complex protein	I	85	103	1.21	1.0	32	0.38	3.1
SAR0930	971604	972506	fumarylacetoacetate (FAA) hydrolase family protein	Q	16	23	1.44	1.0	3	0.19	0.0
srtA	2692490	2693110	sortase	M	16	23	1.44	1.0	6	0.38	0.9
acpD	236262	236888	azoreductase	I	19	27	1.42	1.0	3	0.16	0.1
SAR2349	2422708	2423148	MarR family regulatory protein	K	19	27	1.42	1.0	5	0.26	0.2
SAR0134	147727	149157	polysaccharide biosynthesis protein	R	35	46	1.31	0.9	5	0.14	0.4
SAR1003	1049828	1050148	hypothetical protein	S	12	18	1.50	0.9	3	0.25	0.1
SAR2699	2790963	2791460	glutathione peroxidase	O	12	18	1.50	0.9	3	0.25	0.1
capO	182924	184186	capsular polysaccharide synthesis enzyme	M	48	61	1.27	0.9	20	0.42	2.7

Gene	Start	End	Product	COG	# Core BiPs	# Homo-plasies	Homoplasy rate	Poisson test: −log10(p)	# Non-synonymous BiPs	Non-synonymous probability	Binomial test: −log10(p)
SAR0217	252576	254825	formate acetyltransferase	C	20	28	1.40	0.9	1	0.05	1.0
SAR1487	1581416	1582384	L-asparaginase	E	33	43	1.30	0.9	10	0.30	0.7
opuCA	2616954	2618180	glycine betaine/carnitine/choline transport ATP-binding protein	E	61	75	1.23	0.9	13	0.21	0.0
SAR0125	138620	139396	aldolase	G	21	29	1.38	0.9	5	0.24	0.1
SAR0798	835912	836406	hypothetical protein	S	13	19	1.46	0.9	1	0.08	0.5
SAR2715	2810984	2811433	arginine repressor family protein	K	13	19	1.46	0.9	3	0.23	0.1
SAR2432	2501694	2502641	CorA-like Mg2+ transporter protein	P	18	25	1.39	0.9	5	0.28	0.2
SAR2697	2788018	2788626	precorrin-2 dehydrogenase	H	18	25	1.39	0.9	8	0.44	1.4
copA	2724224	2726632	copper importing ATPase A	P	78	94	1.21	0.9	18	0.23	0.2
SAR2778	2884553	2885569	nickel transport protein	P	52	65	1.25	0.9	10	0.19	0.1
SAR0392	428229	428483	hypothetical protein		9	14	1.56	0.9	2	0.22	0.0
SAR1646	1714756	1715100	diacylglycerol kinase	M	9	14	1.56	0.9	1	0.11	0.2
SAR2546	2627660	2628115	lipoprotein	S	9	14	1.56	0.9	4	0.44	1.0
sirR	690411	691055	metalloregulator	K	22	30	1.36	0.9	0	0.00	2.1
arlR	1487662	1488321	response regulator protein	T	22	30	1.36	0.9	1	0.05	1.2
SAR2118	2179143	2179886	hypothetical protein		22	30	1.36	0.9	3	0.14	0.2
SAR0110	117060	118727	Na+/Pi-cotransporter protein	P	54	67	1.24	0.9	5	0.09	1.5
SAR0100	104041	104202	hypothetical protein		4	7	1.75	0.9	3	0.75	1.5
SAR0401a	437550	437651	hypothetical protein		4	7	1.75	0.9	3	0.75	1.5
SAR1928	2008499	2009239	ABC transporter ATP-binding protein	V	19	26	1.37	0.8	5	0.26	0.2
SAR0245	285723	286766	zinc-binding dehydrogenase	R	50	62	1.24	0.8	17	0.34	1.4
fmhA	2573189	2574439	FemAB family protein	V	50	62	1.24	0.8	8	0.16	0.3
SAR0859	902609	903031	OsmC-like protein	O	14	20	1.43	0.8	8	0.57	2.4
SAR0344	389834	390049	Sec-independent protein translocase protein	U	6	10	1.67	0.8	0	0.00	0.5
SAR0920	955061	956305	NAD-specific glutamate dehydrogenase	E	20	27	1.35	0.8	0	0.00	1.9
isdC	1150309	1150992	surface anchored protein	M	20	27	1.35	0.8	7	0.35	0.8
SAR1392	1449150	1449851	oligopeptide transporter ATPase	P	20	27	1.35	0.8	1	0.05	1.0
SAR2666	2753410	2753778	hypothetical protein		20	27	1.35	0.8	12	0.60	3.7
gyrB	5037	6968	DNA gyrase subunit B	L	43	54	1.26	0.8	6	0.14	0.6
SAR1706	1768080	1768502	hypothetical protein	K	2	4	2.00	0.8	0	0.00	0.0
rpsI	2387118	2387510	30S ribosomal protein S9	J	2	4	2.00	0.8	0	0.00	0.0
SAR2428b	2498195	2498326	hypothetical protein		2	4	2.00	0.8	1	0.50	0.4
SAR2631	2717241	2717468	hypothetical protein	P	2	4	2.00	0.8	0	0.00	0.0
pyrP	1220499	1221806	uracil permease	F	31	40	1.29	0.8	2	0.06	1.3
SAR0397	430909	432528	hypothetical protein		10	15	1.50	0.8	6	0.60	2.0
recR	512668	513264	recombination protein RecR	L	10	15	1.50	0.8	1	0.10	0.2
SAR0902	937018	937377	hypothetical protein	S	10	15	1.50	0.8	2	0.20	0.0
SAR1096	1143099	1143488	hypothetical protein		10	15	1.50	0.8	3	0.30	0.3
SAR1572	1645345	1645851	hypothetical protein	S	24	32	1.33	0.8	10	0.42	1.6
SAR1650	1717536	1718525	hypothetical protein	S	24	32	1.33	0.8	0	0.00	2.3
SAR1066	1112113	1112739	hypothetical protein		15	21	1.40	0.8	2	0.13	0.1
pbuX	441961	443229	xanthine permease	F	36	46	1.28	0.8	7	0.19	0.0
SAR1782	1854803	1855744	hypothetical protein	R	38	48	1.26	0.8	9	0.24	0.2
SAR2590	2674624	2675235	DedA family protein	S	26	34	1.31	0.8	4	0.15	0.2
aroE	1739025	1739831	shikimate 5-dehydrogenase	E	27	35	1.30	0.7	12	0.44	2.1
SAR0171	189771	189974	hypothetical protein		7	11	1.57	0.7	2	0.29	0.2
SAR0508	546912	547304	cell division protein	D	5	8	1.60	0.7	1	0.20	0.0
SAR0349	392566	393195	hypothetical protein		17	23	1.35	0.7	5	0.29	0.4
SAR1751	1813636	1814451	hypothetical protein	O	17	23	1.35	0.7	1	0.06	0.8
opp-1A	2635602	2637200	oligopeptide transporter substrate binding protein	E	51	62	1.22	0.7	6	0.12	0.9
ribD	1942517	1943560	bifunctional riboflavin biosynthesis protein	H	28	36	1.29	0.7	10	0.36	1.0

Gene	Start	End	Product	COG	# Core BiPs	# Homo-plasies	Homoplasy rate	Poisson test: −log10(p)	# Non-synonymous BiPs	Non-synonymous probability	Binomial test: −log10(p)
trxB	860926	861861	thioredoxin reductase	O	21	28	1.33	0.7	2	0.10	0.5
SAR0851	898053	898622	hypothetical protein	S	21	28	1.33	0.7	9	0.43	1.5
SAR1193	1241201	1242508	hypothetical protein	J	42	52	1.24	0.7	16	0.38	1.9
SAR2267	2348295	2349326	FecCD transport family protein	P	42	52	1.24	0.7	10	0.24	0.2
opuCC	2615364	2616305	glycine betaine/carnitine/choline-binding lipoprotein precursor	M	42	52	1.24	0.7	11	0.26	0.3
SAR0849	897401	897619	hypothetical protein		12	17	1.42	0.7	5	0.42	0.8
SAR1146	1191522	1191749	hypothetical protein		12	17	1.42	0.7	5	0.42	0.8
malA	1655144	1656793	alpha-D-1,4-glucosidase	G	53	64	1.21	0.7	13	0.25	0.2
SAR1403	1460096	1460998	hypothetical protein	S	29	37	1.28	0.7	5	0.17	0.1
SAR2740	2846465	2847076	hypothetical protein	R	43	53	1.23	0.7	15	0.35	1.4
gltA	498641	503140	glutamate synthase, large subunit	E	110	127	1.15	0.7	24	0.22	0.0
SAR0360	406380	407477	GTP-dependent nucleic acid-binding protein EngD	J	31	39	1.26	0.7	1	0.03	2.1
SAR1577	1649167	1649415	hypothetical protein		13	18	1.38	0.7	9	0.69	3.5
SAR1731	1794253	1794468	hypothetical protein		8	12	1.50	0.7	3	0.38	0.4
upp	2267752	2268381	uracil phosphoribosyltransferase	F	8	12	1.50	0.7	1	0.13	0.0
SAR0339	384222	384767	acetyltransferase	J	32	40	1.25	0.7	16	0.50	3.5
SAR0989	1034486	1034737	hypothetical protein		6	9	1.50	0.6	4	0.67	1.7
SAR1218	1268347	1270953	hypothetical protein	S	96	111	1.16	0.6	42	0.44	6.0
fruA	786922	788880	PTS transport system, fructose-specific IIABC component	G	75	88	1.17	0.6	11	0.15	0.7
SAR0845	893407	894432	hypothetical protein		68	80	1.18	0.6	34	0.50	6.6
SAR1237	1291444	1292226	phosphatidate cytidylyltransferase	I	14	19	1.36	0.6	1	0.07	0.5
tpiA	875019	875780	triosephosphate isomerase	G	21	27	1.29	0.6	8	0.38	1.0
SAR1702	1764910	1766052	cysteine desulfurase	E	21	27	1.29	0.6	8	0.38	1.0
SAR1074	1120025	1120822	ABC transporter permease	E	26	33	1.27	0.6	2	0.08	1.0
SAR2213	2280446	2281306	fructose-bisphosphate aldolase	G	26	33	1.27	0.6	1	0.04	1.5
icaA	2852074	2853312	N-glycosyltransferase	M	26	33	1.27	0.6	4	0.15	0.2
SAR0572	628019	628378	hypothetical protein		9	13	1.44	0.6	2	0.22	0.0
SAR1944	2027140	2027292	hypothetical protein		9	13	1.44	0.6	3	0.33	0.4
SAR0484	523407	523736	hypothetical protein	S	3	5	1.67	0.6	0	0.00	0.0
SAR1212	1263787	1264119	DNA-binding protein	S	3	5	1.67	0.6	1	0.33	0.3
rplS	1267753	1268103	50S ribosomal protein L19	J	3	5	1.67	0.6	0	0.00	0.0
SAR2227	2295087	2295530	non-heme iron-containing ferritin	P	3	5	1.67	0.6	0	0.00	0.0
SAR2613	2697783	2697986	hypothetical protein		3	5	1.67	0.6	2	0.67	0.9
SAR1876	1962632	1963633	hypothetical protein	R	41	50	1.22	0.6	19	0.46	3.4
SAR0318	363839	364666	hypothetical protein	I	22	28	1.27	0.6	11	0.50	2.5
SAR0350	393564	394697	hypothetical protein	S	36	44	1.22	0.6	7	0.19	0.0
SAR0608	657373	658311	aldo/keto reductase family protein	C	37	45	1.22	0.6	9	0.24	0.2
SAR1792	1865464	1866234	hypothetical protein	R	23	29	1.26	0.6	5	0.22	0.0
SAR0235	276977	278506	PTS transport system, IIBC component	G	43	52	1.21	0.6	9	0.21	0.0
ald2	1539564	1540682	alanine dehydrogenase	E	55	65	1.18	0.6	5	0.09	1.5
SAR1073	1118938	1120032	ABC transporter ATP-binding protein	E	38	46	1.21	0.6	3	0.08	1.3
SAR0404	439658	440320	hypothetical protein		16	21	1.31	0.6	3	0.19	0.0
SAR1493	1588267	1588812	hypothetical protein	S	16	21	1.31	0.6	5	0.31	0.4
SAR2275	2357791	2358327	hypothetical protein		16	21	1.31	0.6	6	0.38	0.9
dnaA	517	1878	chromosomal replication initiation protein	L	24	30	1.25	0.6	3	0.13	0.3
SAR2458	2529046	2529561	acetyltransferase (GNAT) family protein	R	24	30	1.25	0.6	13	0.54	3.4
ahpF	432643	434166	alkyl hydroperoxide reductase subunit F	O	45	54	1.20	0.6	9	0.20	0.0
murG	1489738	1490808	undecaprenyldiphospho-muramoylpentapeptide beta-N-acetylglucosaminyltransferase	M	29	36	1.24	0.6	6	0.21	0.0
SAR2261	2342707	2343390	hypothetical protein	R	46	55	1.20	0.6	1	0.02	3.4

Gene	Start	End	Product	COG	# Core BiPs	# Homo-plasies	Homoplasy rate	Poisson test: −log10(p)	# Non-synonymous BiPs	Non-synonymous probability	Binomial test: −log10(p)
SAR0739	774312	774755	MarR family regulatory protein	K	1	2	2.00	0.6	0	0.00	0.0
SARt013	865895	865966	tRNA-Arg		1	2	2.00	0.6	0	0.00	0.0
secG	879596	879829	preprotein translocase subunit SecG	U	1	2	2.00	0.6	0	0.00	0.0
rpmI	1821758	1821958	50S ribosomal protein L35	J	1	2	2.00	0.6	0	0.00	0.0
SARs019	1882961	1883179	NA		1	2	2.00	0.6	0	0.00	0.0
SARs022	2183350	2183839	NA		1	2	2.00	0.6	1	1.00	0.7
hld	2183697	2183834	delta-hemolysin		1	2	2.00	0.6	1	1.00	0.7
SAR2519a	2596407	2596514	hypothetical protein		1	2	2.00	0.6	1	1.00	0.7
SAR2718	2814526	2814999	hypothetical protein		1	2	2.00	0.6	0	0.00	0.0
xerD	1645929	1646816	integrase/recombinase	L	30	37	1.23	0.6	4	0.13	0.4
SAR0944	987866	988300	hypothetical protein		17	22	1.29	0.6	3	0.18	0.0
SAR0523	569701	570261	glutamine amidotransferase subunit PdxT	H	11	15	1.36	0.5	1	0.09	0.3
SAR1058	1104849	1105082	glutaredoxin	O	11	15	1.36	0.5	6	0.55	1.8
SAR0522	568810	569697	pyridoxal biosynthesis lyase PdxS	H	18	23	1.28	0.5	0	0.00	1.7
trmD	1266913	1267650	tRNA (guanine-N(1)-)-methyltransferase	J	18	23	1.28	0.5	3	0.17	0.1
obgE	1788871	1790163	GTPase ObgE	R	32	39	1.22	0.5	3	0.09	0.9
SAR1342	1392055	1392369	hypothetical protein		8	11	1.38	0.5	1	0.13	0.0
SAR0470	497576	498460	LysR family regulatory protein	K	33	40	1.21	0.5	8	0.24	0.2
SAR0189	214110	215750	thiamine pyrophosphate enzyme	R	59	69	1.17	0.5	17	0.29	0.7
SAR0967	1010674	1011660	hypothetical protein	R	52	61	1.17	0.5	34	0.65	11.0
SAR1001	1047536	1047817	hypothetical protein		12	16	1.33	0.5	6	0.50	1.6
SAR2692	2780653	2781219	hypothetical protein	K	12	16	1.33	0.5	3	0.25	0.1
SAR1816	1896617	1897807	hypothetical protein		35	42	1.20	0.5	15	0.43	2.2
hemH	2004376	2005299	ferrochelatase	H	35	42	1.20	0.5	10	0.29	0.5
argS	663784	665445	arginyl-tRNA synthetase	J	55	64	1.16	0.5	7	0.13	0.9
SAR0822	864641	865585	hypothetical protein	S	20	25	1.25	0.5	0	0.00	1.9
SAR1337	1386066	1387460	aspartate kinase	E	20	25	1.25	0.5	2	0.10	0.5
leuC	2209731	2211098	isopropylmalate isomerase large subunit	E	42	50	1.19	0.5	6	0.14	0.5
SAR0307	349584	350615	hypothetical protein	G	36	43	1.19	0.5	5	0.14	0.5
SAR0861	903978	904517	nitroreductase family protein	C	9	12	1.33	0.5	3	0.33	0.4
fur	1646865	1647314	iron uptake regulatory protein	P	9	12	1.33	0.5	0	0.00	0.7
rsbW	2223526	2224005	serine-protein kinase RsbW	T	9	12	1.33	0.5	2	0.22	0.0
SAR2596	2681229	2683193	hypothetical protein	G	101	114	1.13	0.5	9	0.09	2.8
pbp4	697738	699033	penicillin-binding protein 4	M	43	51	1.19	0.5	13	0.30	0.7
SAR0527	572876	573883	ATPguanido phosphotransferase	E	13	17	1.31	0.5	1	0.08	0.5
SAR1377	1433735	1434997	ImpB/MucB/SamB family protein	L	38	45	1.18	0.5	9	0.24	0.2
SAR2006	2097271	2098740	nicotinate phosphoribosyltransferase	H	46	54	1.17	0.5	10	0.22	0.0
SAR0168	185806	186189	hypothetical protein	S	10	13	1.30	0.5	3	0.30	0.3
SAR1968	2059674	2060204	hypothetical protein	I	10	13	1.30	0.5	3	0.30	0.3
SAR2401	2472535	2472891	hypothetical protein		10	13	1.30	0.5	9	0.90	5.1
SAR2517	2591233	2591919	dethiobiotin synthetase	H	29	35	1.21	0.4	17	0.59	4.8
SAR0558	609162	610127	hypothetical protein	G	24	29	1.21	0.4	4	0.17	0.1
SAR1867	1954957	1955310	CrcB-like protein	D	24	29	1.21	0.4	10	0.42	1.6
tmRNA	883681	884039	NA		5	7	1.40	0.4	0	0.00	0.2
rpoA	2394058	2395002	DNA-directed RNA polymerase subunit alpha	K	5	7	1.40	0.4	0	0.00	0.2
SAR0127	140602	141366	hypothetical protein	K	11	14	1.27	0.4	1	0.09	0.3
SAR1336	1385422	1386012	hypothetical protein		11	14	1.27	0.4	3	0.27	0.1
dinR	1399278	1399901	LexA repressor	T	11	14	1.27	0.4	2	0.18	0.0
SAR1976	2065913	2066614	hypothetical protein	S	11	14	1.27	0.4	3	0.27	0.1
SAR0521	567324	568706	GntR family regulatory protein	E	52	60	1.15	0.4	13	0.25	0.3
fmhB	2419884	2421149	peptidoglycan pentaglycine interpeptide biosynthesis protein	V	31	37	1.19	0.4	6	0.19	0.0
fmt	1075526	1076719	autolysis and methicillin resistant-related	V	53	61	1.15	0.4	5	0.09	1.5

Gene	Start	End	Product	COG	# Core BiPs	# Homo-plasies	Homoplasy rate	Poisson test: −log10(p)	# Non-synonymous BiPs	Non-synonymous probability	Binomial test: −log10(p)
			protein								
codY	1285917	1286690	transcriptional repressor CodY	K	16	20	1.25	0.4	0	0.00	1.5
SAR1861	1950445	1950888	hypothetical protein		16	20	1.25	0.4	5	0.31	0.4
SAR0885	923393	924220	hypothetical protein	R	33	39	1.18	0.4	2	0.06	1.5
SAR0456	484227	484901	hypothetical protein	I	27	32	1.19	0.4	7	0.26	0.2
SAR1010	1053367	1053723	hypothetical protein	S	17	21	1.24	0.4	0	0.00	1.5
SAR0332	378032	378472	hypothetical protein	S	18	22	1.22	0.4	9	0.50	2.2
SAR0609	658521	659057	hypothetical protein	R	18	22	1.22	0.4	1	0.06	0.8
SAR1241	1300311	1300778	hypothetical protein	S	6	8	1.33	0.4	2	0.33	0.2
SAR0309	352051	352974	hypothetical protein	Q	37	43	1.16	0.4	13	0.35	1.2
SAR2001	2093617	2094783	staphopain protease		45	52	1.16	0.4	22	0.49	4.2
deoD1	152151	152858	purine nucleoside phosphorylase	F	19	23	1.21	0.4	4	0.21	0.0
murQ	219704	220603	N-acetylmuramic acid-6-phosphate etherase	R	19	23	1.21	0.4	4	0.21	0.0
phoP	1839311	1840012	alkaline phosphatase synthesis transcriptional regulatory protein	T	19	23	1.21	0.4	3	0.16	0.1
rpoE	2284100	2284630	DNA-directed RNA polymerase subunit delta	K	19	23	1.21	0.4	0	0.00	1.7
SAR2295	2380382	2380807	hypothetical protein		19	23	1.21	0.4	11	0.58	3.2
SAR0660	707493	708461	dihydroxyacetone kinase subunit DhaK	G	47	54	1.15	0.4	15	0.32	1.0
SAR2530	2606974	2608803	hypothetical protein	E	39	45	1.15	0.4	3	0.08	1.5
SAR1088	1133314	1136766	pyruvate carboxylase	C	101	112	1.11	0.4	15	0.15	0.9
SAR0461	490014	491039	ABC transporter ATP-binding protein	P	20	24	1.20	0.4	5	0.25	0.1
SAR2418	2487333	2488217	LysR family regulatory protein	K	21	25	1.19	0.3	2	0.10	0.5
SAR1071	1117967	1118242	hypothetical protein	S	7	9	1.29	0.3	5	0.71	2.2
SAR2707	2800529	2800984	regulatory protein	K	7	9	1.29	0.3	3	0.43	0.8
ddl	2242427	2243497	D-alanyl-alanine synthetase A	M	22	26	1.18	0.3	6	0.27	0.4
SAR0013	17309	18295	hypothetical protein	S	30	35	1.17	0.3	10	0.33	0.9
prfB	847718	848755	peptide chain release factor 2		30	35	1.17	0.3	4	0.13	0.4
SAR1059	1105280	1106641	cytochrome ubiquinol oxidase	C	30	35	1.17	0.3	4	0.13	0.4
SAR1581	1651248	1652168	hypothetical protein	R	23	27	1.17	0.3	7	0.30	0.5
SAR1616	1688475	1688972	hypothetical protein	U	23	27	1.17	0.3	17	0.74	7.0
SAR1225	1277983	1278855	SMF family protein	U	12	15	1.25	0.3	3	0.25	0.1
SAR0130	143519	144490	NAD dependent epimerase/dehydratase family protein	M	69	77	1.12	0.3	12	0.17	0.3
SAR1025	1068123	1068542	MarR family regulatory protein	K	8	10	1.25	0.3	1	0.13	0.0
trxA	1166612	1166926	thioredoxin	O	8	10	1.25	0.3	1	0.13	0.0
glnR	1348619	1348987	glutamine synthetase repressor	K	8	10	1.25	0.3	0	0.00	0.7
SAR1350	1400041	1400277	hypothetical protein		8	10	1.25	0.3	6	0.75	2.7
lacF	2367181	2367492	PTS system, lactose-specific IIA component	G	8	10	1.25	0.3	1	0.13	0.0
SAR2428	2497368	2498015	hypothetical protein	S	8	10	1.25	0.3	4	0.50	1.2
SAR2670	2756711	2756986	hypothetical protein		8	10	1.25	0.3	1	0.13	0.0
scrR	2189789	2190739	sucrose operon repressor	K	32	37	1.16	0.3	11	0.34	1.1
SAR0119	130149	131129	pyridoxal-phosphate dependent enzyme	E	13	16	1.23	0.3	2	0.15	0.0
SAR2473	2547141	2547506	hypothetical protein		13	16	1.23	0.3	2	0.15	0.0
sbi	2581308	2582621	IgG-binding protein		13	16	1.23	0.3	3	0.23	0.1
SAR0907	942821	943975	hypothetical protein	C	33	38	1.15	0.3	9	0.27	0.4
SAR0209	242725	243804	oxidoreductase	R	34	39	1.15	0.3	9	0.26	0.3
SAR2672	2758250	2758699	hypothetical protein	S	14	17	1.21	0.3	7	0.50	1.8
SAR0276	320524	321690	hypothetical protein		45	51	1.13	0.3	19	0.42	2.8
SAR1011	1054214	1055173	transport system extracellular binding lipoprotein	P	45	51	1.13	0.3	15	0.33	1.2
SAR1456	1547901	1549046	hypothetical protein	L	45	51	1.13	0.3	5	0.11	1.0

Gene	Start	End	Product	COG	# Core BiPs	# Homo-plasies	Homoplasy rate	Poisson test: −log10(p)	# Non-synonymous BiPs	Non-synonymous probability	Binomial test: −log10(p)
SAR0218	254848	255603	pyruvate formate-lyase activating enzyme	O	9	11	1.22	0.3	0	0.00	0.7
SAR2398	2468847	2469176	hypothetical protein		9	11	1.22	0.3	8	0.89	4.5
SAR2434	2503226	2503645	hypothetical protein		9	11	1.22	0.3	0	0.00	0.7
SAR2561	2644560	2644982	hypothetical protein	S	9	11	1.22	0.3	1	0.11	0.2
SAR2686	2773678	2773878	hypothetical protein		9	11	1.22	0.3	6	0.67	2.4
capG	174782	175906	capsular polysaccharide synthesis enzyme	M	28	32	1.14	0.3	6	0.21	0.0
SAR2445	2514738	2515403	ABC transporter ATP-binding protein	V	28	32	1.14	0.3	10	0.36	1.0
SAR2743	2848369	2849136	capsule synthesis protein	G	28	32	1.14	0.3	5	0.18	0.1
SAR2490	2565394	2566146	hypothetical protein	S	37	42	1.14	0.3	13	0.35	1.2
SAR2681	2767014	2768462	amino acid permease	E	38	43	1.13	0.3	7	0.18	0.1
SAR0906	942391	942765	hypothetical protein	Q	15	18	1.20	0.3	5	0.33	0.5
menB	1061322	1062143	naphthoate synthase	H	15	18	1.20	0.3	2	0.13	0.1
panB	2762550	2763368	3-methyl-2-oxobutanoate hydroxymethyltransferase	H	15	18	1.20	0.3	6	0.40	1.0
SAR0918	952327	953454	NADHflavin oxidoreductase / NADH oxidase family protein	C	39	44	1.13	0.3	15	0.38	1.8
rnhB	1272222	1272989	ribonuclease HII	L	39	44	1.13	0.3	10	0.26	0.3
SAR1952	2042677	2043627	hypothetical protein	H	39	44	1.13	0.3	21	0.54	5.0
drp35	2873098	2874072	hypothetical protein	G	62	69	1.11	0.3	18	0.29	0.8
SAR2629	2715052	2715222	hypothetical protein		10	12	1.20	0.3	2	0.20	0.0
SAR2688	2775730	2776173	hypothetical protein	R	16	19	1.19	0.3	6	0.38	0.9
arcA	2809402	2810637	arginine deiminase	E	16	19	1.19	0.3	6	0.38	0.9
SAR0575	630127	630675	6-phospho-3-hexuloisomerase	M	17	20	1.18	0.3	6	0.35	0.6
SAR2633	2720317	2720865	TetR family regulatory protein	K	17	20	1.18	0.3	5	0.29	0.4
SAR0876	913572	914333	ABC transporter ATP-binding protein	O	11	13	1.18	0.3	0	0.00	0.9
SAR1726	1790946	1791266	hypothetical protein	J	3	4	1.33	0.3	2	0.67	0.9
SAR0437	466838	467146	hypothetical protein		18	21	1.17	0.3	5	0.28	0.2
SAR1848	1938025	1938852	hypothetical protein	I	18	21	1.17	0.3	6	0.33	0.6
arsR2	1946186	1946500	arsenical resistance operon repressor 2	K	18	21	1.17	0.3	9	0.50	2.2
SAR2263	2344640	2345155	hypothetical protein	R	12	14	1.17	0.2	6	0.50	1.6
SAR2539	2618850	2619449	hypothetical protein	S	12	14	1.17	0.2	4	0.33	0.5
SAR1571	1644522	1645277	hypothetical protein	S	19	22	1.16	0.2	4	0.21	0.0
SAR1421	1479150	1479941	hypothetical protein	R	20	23	1.15	0.2	0	0.00	1.9
SAR0943	987040	987555	hypothetical protein	R	13	15	1.15	0.2	5	0.38	0.8
fhuD	706243	707259	ferrichrome transport permease	P	30	34	1.13	0.2	8	0.27	0.3
SAR2017	2110080	2111120	hypothetical protein	R	30	34	1.13	0.2	10	0.33	0.9
birA	1561164	1562135	BirA bifunctional protein [includes biotin operon repressor; biotin--[acetyl-CoA-carboxylase] synthetase	H	21	24	1.14	0.2	8	0.38	1.0
SAR2544	2625338	2626000	ABC transporter ATP-binding protein	R	21	24	1.14	0.2	5	0.24	0.1
SAR0400	435242	435997	nitroreductase family protein	C	22	25	1.14	0.2	4	0.18	0.0
SAR0821	863537	864532	hypothetical protein	S	22	25	1.14	0.2	2	0.09	0.7
SAR0666	713100	713528	hypothetical protein		14	16	1.14	0.2	5	0.36	0.7
sigB	2222781	2223551	RNA polymerase sigma factor SigB	K	14	16	1.14	0.2	1	0.07	0.5
SAR2578	2659356	2659664	hypothetical protein		23	26	1.13	0.2	14	0.61	4.3
SAR1453	1542055	1545495	hypothetical protein	R	133	143	1.08	0.2	40	0.30	1.7
dnaN	2156	3289	DNA polymerase III subunit beta	L	24	27	1.13	0.2	1	0.04	1.4
SAR0489	527036	527284	hypothetical protein	L	4	5	1.25	0.2	2	0.50	0.7
rplY	538883	539536	50S ribosomal protein L25/general stress protein Ctc	J	4	5	1.25	0.2	0	0.00	0.2
rpsG	599110	599580	30S ribosomal protein S7	J	4	5	1.25	0.2	0	0.00	0.2
SAR0588	640097	640465	hypothetical protein	S	4	5	1.25	0.2	0	0.00	0.2
nucI	1383576	1384109	thermonuclease	L	4	5	1.25	0.2	0	0.00	0.2
SAR1611	1682866	1683252	hypothetical protein	P	4	5	1.25	0.2	0	0.00	0.2

Gene	Start	End	Product	COG	# Core BiPs	# Homo-plasies	Homoplasy rate	Poisson test: −log10(p)	# Non-synonymous BiPs	Non-synonymous probability	Binomial test: −log10(p)
SAR2687	2773938	2775557	AMP-binding enzyme	I	63	69	1.10	0.2	11	0.17	0.3
SAR2303	2389015	2389821	cobalt transport protein	P	36	40	1.11	0.2	8	0.22	0.1
SAR1472	1566120	1566707	hypothetical protein	S	25	28	1.12	0.2	12	0.48	2.5
murA1	2257046	2258311	UDP-N-acetylglucosamine 1-carboxyvinyltransferase	M	37	41	1.11	0.2	4	0.11	0.8
lepA	1729531	1731354	GTP-binding protein LepA	M	52	57	1.10	0.2	3	0.06	2.4
SAR2524	2599479	2600690	transporter protein	G	53	58	1.09	0.2	10	0.19	0.1
SAR2662	2749363	2750253	cobalamin synthesis protein/P47K family protein	R	39	43	1.10	0.2	10	0.26	0.3
SAR1479	1573111	1574070	heptaprenyl diphosphate synthase component II	H	40	44	1.10	0.2	5	0.13	0.6
SAR0882	920111	921151	hypothetical protein	P	28	31	1.11	0.2	6	0.21	0.0
SAR2427	2496236	2497135	ABC transporter ATP-binding protein	R	28	31	1.11	0.2	10	0.36	1.0
gidA	2898327	2900204	tRNA uridine 5-carboxymethylaminomethyl modification enzyme GidA	D	55	60	1.09	0.2	9	0.16	0.4
SAR1653	1719939	1721285	hypothetical protein	J	29	32	1.10	0.2	1	0.03	1.9
purH	1092309	1093787	bifunctional phosphoribosylaminoimidazolecarboxamide formyltransferase/IMP cyclohydrolase	F	42	46	1.10	0.2	11	0.26	0.3
rplA	587958	588647	50S ribosomal protein L1	J	5	6	1.20	0.2	0	0.00	0.2
rplJ	588922	589422	50S ribosomal protein L10	J	5	6	1.20	0.2	1	0.20	0.0
SAR1244	1302276	1302593	ribosomal protein	J	5	6	1.20	0.2	1	0.20	0.0
SAR1824	1906791	1907102	hypothetical protein	S	5	6	1.20	0.2	2	0.40	0.5
SAR2792	2895415	2895810	hypothetical protein		5	6	1.20	0.2	1	0.20	0.0
SAR0886	924247	925566	5'-nucleotidase	F	44	48	1.09	0.2	14	0.32	1.0
SAR0653	699454	701181	ABC transporter ATP-binding protein	V	45	49	1.09	0.2	3	0.07	1.8
purR	534330	535154	pur operon repressor	F	6	7	1.17	0.2	1	0.17	0.0
SAR1325b	1376455	1376565	hypothetical protein		6	7	1.17	0.2	4	0.67	1.7
SAR1481	1574800	1575372	hypothetical protein		6	7	1.17	0.2	0	0.00	0.5
SAR2380	2447590	2447916	hypothetical protein		6	7	1.17	0.2	2	0.33	0.2
SAR2557	2639466	2640287	hypothetical protein	E	15	17	1.13	0.2	3	0.20	0.0
SAR0167	185473	185799	heme-degrading monooxygenase IsdI	R	7	8	1.14	0.2	1	0.14	0.0
SAR0231	273274	273630	hypothetical protein	S	7	8	1.14	0.2	4	0.57	1.4
SAR0850	897681	897965	hypothetical protein		7	8	1.14	0.2	3	0.43	0.8
SAR1100	1145912	1146469	hypothetical protein	R	7	8	1.14	0.2	4	0.57	1.4
SAR1131	1178361	1178711	hypothetical protein		7	8	1.14	0.2	3	0.43	0.8
SAR1168	1211996	1212592	hypothetical protein	D	7	8	1.14	0.2	1	0.14	0.0
gmk	1233003	1233626	guanylate kinase	F	7	8	1.14	0.2	2	0.29	0.2
SAR2211	2278297	2278635	hypothetical protein	K	7	8	1.14	0.2	1	0.14	0.0
SAR2724	2825522	2826082	isochorismatase family protein	Q	7	8	1.14	0.2	1	0.14	0.0
hisD	2863246	2864502	histidinol dehydrogenase	E	64	69	1.08	0.1	13	0.20	0.0
nrdD	2784062	2785912	anaerobic ribonucleoside triphosphate reductase	F	46	50	1.09	0.1	5	0.11	1.0
SAR0993	1039964	1041322	sodium transport protein	P	30	33	1.10	0.1	3	0.10	0.7
SAR2387	2455385	2455807	hypothetical protein	S	17	19	1.12	0.1	3	0.18	0.0
SAR0237	279841	279987	hypothetical protein		8	9	1.13	0.1	7	0.88	3.9
SAR0889	927158	927424	hypothetical protein		8	9	1.13	0.1	3	0.38	0.4
SAR2347	2421538	2421789	hypothetical protein		8	9	1.13	0.1	6	0.75	2.7
SAR0680	727954	728670	hypothetical protein	S	32	35	1.09	0.1	4	0.13	0.5
SAR0980	1024660	1026228	sodiumalanine symporter family protein	E	32	35	1.09	0.1	4	0.13	0.5
SAR0172	190154	190666	hypothetical protein		19	21	1.11	0.1	5	0.26	0.2
fabH	989038	989979	3-oxoacyl-(acyl carrier protein) synthase III	I	19	21	1.11	0.1	4	0.21	0.0
yycF	24897	25604	response regulator protein	T	20	22	1.10	0.1	0	0.00	1.9
SAR0204	237097	237675	peptidase	M	20	22	1.10	0.1	8	0.40	1.3

Gene	Start	End	Product	COG	# Core BiPs	# Homo-plasies	Homoplasy rate	Poisson test: −log10(p)	# Non-synonymous BiPs	Non-synonymous probability	Binomial test: −log10(p)
SAR1643	1712667	1713419	recombination protein O	L	20	22	1.10	0.1	5	0.25	0.1
SAR0503	539847	540419	peptidyl-tRNA hydrolase	J	9	10	1.11	0.1	2	0.22	0.0
SAR0973	1016417	1017052	GTP pyrophosphokinase	S	9	10	1.11	0.1	0	0.00	0.7
SAR1987	2075670	2076200	hypothetical protein		9	10	1.11	0.1	5	0.56	1.6
SAR0585	637913	638743	phosphomethylpyrimidine kinase	H	21	23	1.10	0.1	1	0.05	1.2
SAR0738	772512	774185	ABC transporter ATP-binding protein	C	58	62	1.07	0.1	20	0.34	1.6
leuD	2211099	2211671	isopropylmalate isomerase small subunit	E	38	41	1.08	0.1	6	0.16	0.3
SAR2668	2754408	2755274	hypothetical protein	G	38	41	1.08	0.1	13	0.34	1.1
SAR2744	2849133	2849825	capsule synthesis protein	D	38	41	1.08	0.1	3	0.08	1.3
SAR0137	151160	151900	GntR family regulatory protein	K	22	24	1.09	0.1	2	0.09	0.7
SAR1394	1450604	1451434	oligopeptide ABC transporter permease	P	22	24	1.09	0.1	8	0.36	0.9
SAR2701	2794898	2795653	ABC transporter ATP-binding protein	V	22	24	1.09	0.1	3	0.14	0.2
SAR0568	623079	624551	glycosyl transferase	M	61	65	1.07	0.1	21	0.34	1.7
capM	181426	181983	capsular polysaccharide synthesis enzyme	M	10	11	1.10	0.1	3	0.30	0.3
SAR0833	879071	879529	hypothetical protein		10	11	1.10	0.1	2	0.20	0.0
def	1239788	1240276	peptide deformylase		10	11	1.10	0.1	4	0.40	0.6
SAR2027	2117726	2117902	hypothetical protein		10	11	1.10	0.1	3	0.30	0.3
SAR1343	1392588	1394042	amino acid permease	E	41	44	1.07	0.1	9	0.22	0.1
SAR2111	2173762	2175069	sodium transport protein	P	41	44	1.07	0.1	14	0.34	1.3
SAR2241	2308636	2309418	ABC transporter ATP-binding protein	P	24	26	1.08	0.1	9	0.38	1.1
SAR2531	2609035	2610534	hypothetical protein	V	43	46	1.07	0.1	14	0.33	1.0
SAR0593	643989	644741	heme peroxidase	S	11	12	1.09	0.1	4	0.36	0.6
pfkA	1846675	1847643	6-phosphofructokinase	G	11	12	1.09	0.1	1	0.09	0.3
SAR1957	2049063	2049605	hypothetical protein	J	11	12	1.09	0.1	2	0.18	0.0
SAR0933	975316	976632	coenzyme A disulfide reductase	R	44	47	1.07	0.1	13	0.30	0.7
SAR1455	1547485	1547817	hypothetical protein		12	13	1.08	0.1	6	0.50	1.6
ribH	1940209	1940673	6,7-dimethyl-8-ribityllumazine synthase	H	12	13	1.08	0.1	4	0.33	0.5
SAR1699	1762441	1763109	hypothetical protein	R	27	29	1.07	0.1	9	0.33	0.8
SAR0809	849524	850363	hypothetical protein	R	28	30	1.07	0.1	12	0.43	2.0
bfmBAA	1668539	1669531	2-oxoisovalerate dehydrogenase alpha subunit	C	28	30	1.07	0.1	4	0.14	0.3
SAR2504	2575960	2576739	extracellular solute-binding lipoprotein	E	29	31	1.07	0.1	5	0.17	0.1
SAR2634	2721221	2722765	1-pyrroline-5-carboxylate dehydrogenase	C	29	31	1.07	0.1	3	0.10	0.8
SAR0769	802667	802873	hypothetical protein		13	14	1.08	0.1	6	0.46	1.4
gltD	503158	504621	glutamate synthase subunit beta	R	14	15	1.07	0.1	2	0.14	0.1
SAR0582	636375	636740	hypothetical protein		14	15	1.07	0.1	10	0.71	4.1
SAR1264	1326050	1326847	hypothetical protein	S	14	15	1.07	0.1	2	0.14	0.1
rsbU	2224452	2225453	sigma factor sigB regulation protein	T	14	15	1.07	0.1	2	0.14	0.1
panC	2761706	2762557	pantoate--beta-alanine ligase	H	14	15	1.07	0.1	5	0.36	0.7
SAR1257	1319018	1319845	hypothetical protein		15	16	1.07	0.1	1	0.07	0.7
SAR2475	2548573	2548995	small heat shock protein	O	15	16	1.07	0.1	6	0.40	1.0
opuD2	2358502	2360064	glycine betaine transporter 2	M	67	71	1.06	0.1	11	0.16	0.4
SAR2719	2815239	2817113	transcriptional regulator (antiterminator)	K	76	80	1.05	0.1	18	0.24	0.2
SAR1590	1662786	1663919	peptidase	E	47	50	1.06	0.1	20	0.43	3.0
SAR0122	133904	135160	transport protein	G	50	53	1.06	0.1	16	0.32	1.1
bioA	2589897	2591255	adenosylmethionine-8-amino-7-oxononanoate aminotransferase	H	51	54	1.06	0.1	12	0.24	0.1
SAR0319	364724	365938	NADHflavin oxidoreductase / NADH oxidase family protein	C	52	55	1.06	0.1	26	0.50	5.1
SAR0340	385032	385886	lipoprotein	P	55	58	1.05	0.1	9	0.16	0.4
prfC	1034737	1036299	peptide chain release factor 3	J	32	34	1.06	0.1	5	0.16	0.3
lytS	299425	301179	autolysin sensor kinase protein	T	33	35	1.06	0.1	4	0.12	0.5
atl	1068750	1072523	bifunctional autolysin precursor	G	134	140	1.04	0.1	30	0.22	0.1
SAR0556	606275	607153	chaperone protein HchA	R	35	37	1.06	0.1	10	0.29	0.5

Gene	Start	End	Product	COG	# Core BiPs	# Homo-plasies	Homoplasy rate	Poisson test: −log10(p)	# Non-synonymous BiPs	Non-synonymous probability	Binomial test: −log10(p)
dapA	1465863	1466750	dihydrodipicolinate synthase	E	37	39	1.05	0.1	18	0.49	3.5
SAR1873	1961088	1961858	hypothetical protein	E	37	39	1.05	0.1	8	0.22	0.0
SAR0126	139396	140598	pyridoxal-dependent decarboxylase	E	38	40	1.05	0.1	13	0.34	1.1
SAR0274	318934	319593	ABC transporter ATP-binding protein	V	41	43	1.05	0.1	9	0.22	0.1
glmM	2327307	2328662	phosphoglucosamine mutase	G	41	43	1.05	0.1	4	0.10	1.1
SAR2278	2361614	2362618	zinc-binding dehydrogenase	R	42	44	1.05	0.1	8	0.19	0.1
SAR1255	1316923	1318209	protease	R	44	46	1.05	0.1	13	0.30	0.7
SAR0464	492915	493919	hypothetical protein	R	16	17	1.06	0.0	1	0.06	0.7
SAR0475	508595	509323	GntR family regulatory protein	K	18	19	1.06	0.0	5	0.28	0.2
SAR1575	1647419	1647961	ADP-ribose pyrophosphatase	R	18	19	1.06	0.0	6	0.33	0.6
SAR1955	2045751	2046845	hypothetical protein	S	18	19	1.06	0.0	3	0.17	0.1
SAR0881	919428	919742	hypothetical protein		19	20	1.05	0.0	8	0.42	1.4
SAR2139	2201257	2201751	hypothetical protein	R	19	20	1.05	0.0	6	0.32	0.6
SAR0728	765037	765726	hypothetical protein		20	21	1.05	0.0	2	0.10	0.5
ureD	2445790	2446626	urease accessory protein UreD	O	21	22	1.05	0.0	3	0.14	0.2
SAR2436	2504881	2505519	hypothetical protein		21	22	1.05	0.0	5	0.24	0.1
SAR0468	495564	496346	hypothetical protein	S	22	23	1.05	0.0	4	0.18	0.0
SAR0488	526318	527043	hypothetical protein	R	22	23	1.05	0.0	5	0.23	0.1
SAR2266	2347330	2348298	FecCD transport family protein	P	23	24	1.04	0.0	4	0.17	0.1
SAR0630	676699	679101	monovalent cation/H+ antiporter subunit A	P	94	98	1.04	0.0	16	0.17	0.4
ffh	1264145	1265512	signal recognition particle protein	U	25	26	1.04	0.0	5	0.20	0.0
SAR1379	1436389	1437420	peptidase	G	25	26	1.04	0.0	10	0.40	1.3
tig	1818193	1819494	trigger factor	O	25	26	1.04	0.0	2	0.08	0.9
capC	170031	170795	capsular polysaccharide synthesis enzyme	G	27	28	1.04	0.0	10	0.37	1.2
SAR1674	1739845	1740945	GTP-binding protein YqeH	R	27	28	1.04	0.0	6	0.22	0.1
SAR1935	2016744	2017940	DNA repair exonuclease	L	27	28	1.04	0.0	7	0.26	0.2
SAR0262	306030	306734	GntR family regulatory protein	K	29	30	1.03	0.0	5	0.17	0.1
SAR1251	1310610	1312283	hypothetical protein	R	30	31	1.03	0.0	4	0.13	0.4
SAR1823	1905628	1906704	aminopeptidase	G	30	31	1.03	0.0	3	0.10	0.7
SAR1794	1867501	1868640	aminotransferase class-V protein	E	47	49	1.04	0.0	5	0.11	1.1
SAR0994	1041458	1042972	5'-nucleotidase	F	48	50	1.04	0.0	9	0.19	0.1
SAR0737	770884	772515	ABC transporter ATP-binding protein	C	54	56	1.04	0.0	26	0.48	4.8
SAR2615	2699186	2700154	hypothetical protein	I	57	59	1.04	0.0	13	0.23	0.1
SAR2408	2475862	2477466	PTS system, arbutin-like IIBC component	G	59	61	1.03	0.0	7	0.12	1.1
SAR0003	3670	3915	hypothetical protein	S	2	2	1.00	0.0	1	0.50	0.4
SAR0130a	143224	143337	hypothetical protein		3	3	1.00	0.0	3	1.00	2.0
capL	180210	181415	capsular polysaccharide synthesis enzyme	M	47	48	1.02	0.0	17	0.36	1.7
SAR0187	211864	213219	branched-chain amino acid transport system carrier protein	E	45	46	1.02	0.0	4	0.09	1.4
SAR0219	255707	255874	hypothetical protein		6	6	1.00	0.0	5	0.83	2.7
SAR0229	270148	271290	hypothetical protein	S	49	50	1.02	0.0	8	0.16	0.3
SAR0254	294915	296603	hypothetical protein	M	4	4	1.00	0.0	1	0.25	0.0
SAR0272	316614	317606	choloylglycine hydrolase	M	28	28	1.00	0.0	12	0.43	2.0
SAR0352	396501	397253	hypothetical protein	R	22	22	1.00	0.0	9	0.41	1.4
ssb	408315	408818	single-strand DNA-binding protein	L	5	5	1.00	0.0	0	0.00	0.2
SAR0463	491739	492581	lipoprotein	P	32	33	1.03	0.0	8	0.25	0.2
dnaX	510557	512254	DNA polymerase III, tau subunit	L	46	47	1.02	0.0	9	0.20	0.1
SAR0509	547409	547810	hypothetical protein	J	1	1	1.00	0.0	0	0.00	0.0
secE	586404	586586	preprotein translocase subunit SecE	U	1	1	1.00	0.0	1	1.00	0.7
SAR0591	642677	643441	hypothetical protein	S	18	18	1.00	0.0	4	0.22	0.0
SAR0599	650495	650836	hypothetical protein	S	3	3	1.00	0.0	2	0.67	0.9
SAR0612	661080	661586	hypothetical protein		16	16	1.00	0.0	4	0.25	0.1
SAR0618	666806	667693	transport system lipoprotein	P	28	28	1.00	0.0	7	0.25	0.2
tagD	697223	697621	glycerol-3-phosphate cytidylyltransferase	I	2	2	1.00	0.0	0	0.00	0.0

Gene	Start	End	Product	COG	# Core BiPs	# Homo-plasies	Homoplasy rate	Poisson test: −log10(p)	# Non-synonymous BiPs	Non-synonymous probability	Binomial test: −log10(p)
SAR0729	765853	766296	acetyltransferase		13	13	1.00	0.0	8	0.62	2.7
SAR0744	779163	780536	DNA photolyase	L	69	71	1.03	0.0	23	0.33	1.6
SAR0763	798108	798821	radical activating enzyme	O	22	22	1.00	0.0	4	0.18	0.0
SAR0778	815183	815725	hypothetical protein	S	16	16	1.00	0.0	3	0.19	0.0
SAR0874	911965	912159	hypothetical protein		1	1	1.00	0.0	0	0.00	0.0
mnhF	944572	944865	monovalent cation/H+ antiporter subunit F	P	6	6	1.00	0.0	0	0.00	0.5
mnhD	945346	946842	monovalent cation/H+ antiporter subunit D	P	39	40	1.03	0.0	2	0.05	2.0
SAR0995	1043461	1044030	regulatory protein	K	8	8	1.00	0.0	2	0.25	0.2
SAR1012	1055220	1055336	hypothetical protein		7	7	1.00	0.0	4	0.57	1.4
SAR1028	1073341	1073811	hypothetical protein		16	16	1.00	0.0	4	0.25	0.1
qoxD	1077062	1077352	quinol oxidase polypeptide IV	C	3	3	1.00	0.0	0	0.00	0.0
SAR1076	1121637	1122710	ABC transporter	E	41	42	1.02	0.0	8	0.20	0.0
SAR1093	1141173	1141607	hypothetical protein	S	18	18	1.00	0.0	2	0.11	0.4
isdE	1152065	1152943	transport system extracellular binding lipoprotein	P	23	23	1.00	0.0	4	0.17	0.1
SAR1109	1154736	1155059	heme-degrading monooxygenase IsdG	R	10	10	1.00	0.0	1	0.10	0.2
sdhA	1170021	1171787	succinate dehydrogenase flavoprotein subunit	C	59	60	1.02	0.0	6	0.10	1.4
SAR1148	1193259	1193456	DNA-binding protein		8	8	1.00	0.0	4	0.50	1.2
mraW	1198255	1199190	S-adenosyl-methyltransferase MraW	M	22	22	1.00	0.0	4	0.18	0.0
SAR1164	1209547	1210221	hypothetical protein	R	8	8	1.00	0.0	4	0.50	1.2
rpoZ	1233626	1233844	DNA-directed RNA polymerase subunit omega	K	3	3	1.00	0.0	0	0.00	0.0
tsf	1287981	1288862	elongation factor Ts	J	7	7	1.00	0.0	2	0.29	0.2
SAR1269	1331681	1332046	hypothetical protein	S	5	5	1.00	0.0	2	0.40	0.5
thrC	1388925	1389986	threonine synthase	E	24	24	1.00	0.0	8	0.33	0.7
SARs012	1437691	1437899	NA		2	2	1.00	0.0	0	0.00	0.0
SAR1470	1564715	1565032	hypothetical protein	R	8	8	1.00	0.0	3	0.38	0.4
SAR1583	1654195	1655061	AraC family transcription regulator	K	9	9	1.00	0.0	3	0.33	0.4
SAR1591	1664488	1665468	hypothetical protein	S	16	16	1.00	0.0	5	0.31	0.4
SAR1612	1683637	1685109	glycine dehydrogenase subunit 2	E	44	45	1.02	0.0	5	0.11	0.9
SAR1617	1688890	1689189	hypothetical protein		7	7	1.00	0.0	4	0.57	1.4
SAR1619	1689600	1689911	hypothetical protein	U	6	6	1.00	0.0	3	0.50	0.9
grpE	1726164	1726790	heat shock protein GrpE	O	10	10	1.00	0.0	4	0.40	0.6
SAR1691	1752782	1754050	peptidase	O	20	20	1.00	0.0	1	0.05	1.0
SAR1694	1755911	1756219	hypothetical protein	S	2	2	1.00	0.0	0	0.00	0.0
SAR1723	1788403	1788861	hypothetical protein	R	7	7	1.00	0.0	1	0.14	0.0
rplU	1791272	1791580	50S ribosomal protein L21	J	4	4	1.00	0.0	1	0.25	0.0
SARs017	1824382	1824557	NA		2	2	1.00	0.0	0	0.00	0.0
coaE	1831209	1831832	dephospho-CoA kinase	H	20	20	1.00	0.0	4	0.20	0.0
cycA	1843188	1844549	D-serine/D-alanine/glycine transporter	E	64	66	1.03	0.0	3	0.05	3.5
SAR1795	1869008	1870702	septation ring formation regulator EzrA	D	34	35	1.03	0.0	5	0.15	0.4
SAR1799	1873188	1873634	OsmC-like protein	O	23	23	1.00	0.0	13	0.57	3.6
SAR1804	1879480	1880097	acyltransferase	I	15	15	1.00	0.0	0	0.00	1.3
SAR1805	1880269	1881543	protease	O	31	32	1.03	0.0	3	0.10	0.9
dat	1917147	1917995	D-alanine aminotransferase	H	28	28	1.00	0.0	3	0.11	0.6
SAR1842	1931088	1931399	hypothetical protein	P	11	11	1.00	0.0	2	0.18	0.0
SAR1869	1956605	1957516	hypothetical protein		32	33	1.03	0.0	6	0.19	0.1
SAR1933	2012880	2013821	3'-5' exoribonuclease YhaM	R	18	18	1.00	0.0	3	0.17	0.1
glnQ	2029828	2030556	glutamine transport ATP-binding protein	E	11	11	1.00	0.0	1	0.09	0.3
SAR1966	2057878	2058039	hypothetical protein		3	3	1.00	0.0	0	0.00	0.0
SAR1972	2062371	2062646	hypothetical protein		4	4	1.00	0.0	1	0.25	0.0
vraS	2064873	2065916	histidine kinase sensor	T	26	26	1.00	0.0	1	0.04	1.5
SAR1984	2072841	2073341	ferritin	P	7	7	1.00	0.0	2	0.29	0.2

Gene	Start	End	Product	COG	# Core BiPs	# Homo-plasies	Homoplasy rate	Poisson test: −log10(p)	# Non-synonymous BiPs	Non-synonymous probability	Binomial test: −log10(p)
pcrA	2087996	2090188	ATP-dependent DNA helicase	L	43	44	1.02	0.0	5	0.12	0.9
SAR2003	2095431	2095604	hypothetical protein		12	12	1.00	0.0	5	0.42	0.8
SAR2010	2102771	2103868	hypothetical protein		41	42	1.02	0.0	12	0.29	0.6
SAR2011	2104241	2104801	isochorismatase family protein	Q	8	8	1.00	0.0	0	0.00	0.7
thiD	2252365	2253195	phosphomethylpyrimidine kinase	H	26	26	1.00	0.0	14	0.54	3.5
atpB	2265333	2266061	F0F1 ATP synthase subunit A	C	6	6	1.00	0.0	1	0.17	0.0
lacA	2369974	2370402	galactose-6-phosphate isomerase subunit LacA	G	6	6	1.00	0.0	0	0.00	0.5
rpsK	2395077	2395466	30S ribosomal protein S11	J	1	1	1.00	0.0	0	0.00	0.0
adk	2396434	2397081	adenylate kinase	F	2	2	1.00	0.0	0	0.00	0.0
rplR	2399564	2399923	50S ribosomal protein L18	J	4	4	1.00	0.0	2	0.50	0.7
rplN	2402072	2402440	50S ribosomal protein L14	J	2	2	1.00	0.0	0	0.00	0.0
SAR2366	2435908	2436462	BioY family protein	R	16	16	1.00	0.0	1	0.06	0.7
SAR2381	2447980	2448723	hypothetical protein		19	19	1.00	0.0	9	0.47	2.0
SAR2430	2499358	2500566	permease	E	39	40	1.03	0.0	7	0.18	0.2
SAR2467	2538418	2538801	hypothetical protein		14	14	1.00	0.0	8	0.57	2.4
SAR2491	2566374	2566910	acetyltransferase (GNAT) family protein	E	12	12	1.00	0.0	3	0.25	0.1
SAR2494	2568405	2568587	hypothetical protein		3	3	1.00	0.0	1	0.33	0.3
SAR2520	2596545	2596799	hypothetical protein		8	8	1.00	0.0	5	0.63	1.9
SAR2595	2679954	2680724	hypothetical protein		11	11	1.00	0.0	4	0.36	0.6
SAR2614	2698179	2699105	hypothetical protein	P	24	24	1.00	0.0	6	0.25	0.2
SAR2654	2743947	2744177	hypothetical protein	S	9	9	1.00	0.0	0	0.00	0.7
SAR2738	2845569	2845871	hypothetical protein		8	8	1.00	0.0	5	0.63	1.9
SAR2764	2867485	2867982	hypothetical protein	J	4	4	1.00	0.0	2	0.50	0.7
SAR2783	2890435	2890626	hypothetical protein		2	2	1.00	0.0	0	0.00	0.0
trmE	2900271	2901650	tRNA modification GTPase TrmE	R	41	42	1.02	0.0	6	0.15	0.5
SAR2009	2101004	2102566	sodiumsulfate symporter	P	46	46	1.00	0.0	8	0.17	0.2
glpK	1339478	1340974	glycerol kinase	C	45	45	1.00	0.0	8	0.18	0.1
ilvA	2211686	2212954	threonine dehydratase	E	45	45	1.00	0.0	8	0.18	0.1
pnpA	1308278	1310374	polynucleotide phosphorylase/polyadenylase	J	40	40	1.00	0.0	4	0.10	1.1
SAR1703	1766362	1767375	oxygenase	C	39	39	1.00	0.0	11	0.28	0.5
SAR0631	679088	679513	monovalent cation/H+ antiporter subunit B	P	36	36	1.00	0.0	2	0.06	1.8
SAR0334	380226	381152	dioxygenase	R	34	34	1.00	0.0	17	0.50	3.7
SAR1423	1480354	1481163	hypothetical protein		34	34	1.00	0.0	10	0.29	0.5
lldP2	2524359	2525957	L-lactate permease 2	C	32	32	1.00	0.0	4	0.13	0.5
SAR0870	908597	909622	ABC transporter ATP-binding protein	P	31	31	1.00	0.0	9	0.29	0.6
SAR2543	2624569	2625345	hypothetical protein	R	31	31	1.00	0.0	5	0.16	0.2
lip	2855881	2857926	lipase precursor	R	91	92	1.01	0.0	37	0.41	4.5
SAR1466	1558447	1561140	hypothetical protein	L	79	80	1.01	0.0	11	0.14	0.9
SAR2135	2197235	2198845	hypothetical protein	L	61	61	1.00	0.0	24	0.39	2.8
SAR0323	368401	369348	hypothetical protein	K	60	60	1.00	0.0	16	0.27	0.5
SAR0890	927522	927956	hypothetical protein	S	15	14	0.93	0.0	8	0.53	2.2
SAR1982	2070503	2071234	hypothetical protein	R	15	14	0.93	0.0	3	0.20	0.0
SAR2386	2454055	2455137	dehydrogenase	C	15	14	0.93	0.0	3	0.20	0.0
SAR2710	2805077	2805781	regulatory protein	T	15	14	0.93	0.0	5	0.33	0.5
SAR0486	524877	525680	hypothetical protein	S	14	13	0.93	0.0	3	0.21	0.0
SAR1195	1243612	1244355	protein phosphatase	T	14	13	0.93	0.0	5	0.36	0.7
SAR1267	1329709	1330002	hypothetical protein	S	14	13	0.93	0.0	5	0.36	0.7
SAR1441	1499165	1499602	hypothetical protein		14	13	0.93	0.0	4	0.29	0.3
ureG	2445176	2445790	urease accessory protein UreG	O	14	13	0.93	0.0	2	0.14	0.1
icaR	2851350	2851910	ica operon transcriptional regulator	K	14	13	0.93	0.0	0	0.00	1.3
SAR1815	1894832	1895923	bifunctional 3-deoxy-7-phosphoheptulonate synthase/chorismate mutase	E	53	53	1.00	0.0	3	0.06	2.4

Gene	Start	End	Product	COG	# Core BiPs	# Homo-plasies	Homoplasy rate	Poisson test: −log10(p)	# Non-synonymous BiPs	Non-synonymous probability	Binomial test: −log10(p)
SAR1374	1430418	1431401	hypothetical protein	K	13	12	0.92	0.1	3	0.23	0.1
ndk	1572570	1573019	nucleoside diphosphate kinase	F	13	12	0.92	0.1	4	0.31	0.3
SAR2526	2601399	2602055	hypothetical protein	S	13	12	0.92	0.1	1	0.08	0.5
tmk	522762	523379	thymidylate kinase	F	12	11	0.92	0.1	5	0.42	0.8
holB	523950	524876	DNA polymerase III, delta' subunit	L	12	11	0.92	0.1	5	0.42	0.8
SAR0536	584916	585440	hypothetical protein	R	12	11	0.92	0.1	3	0.25	0.1
gpmA	2578730	2579416	phosphoglyceromutase	G	12	11	0.92	0.1	1	0.08	0.3
tagG	694067	694900	teichoic acid ABC transporter permease	G	11	10	0.91	0.1	1	0.09	0.3
lipA	925650	926567	lipoyl synthase	H	11	10	0.91	0.1	1	0.09	0.3
SAR1929	2009373	2009795	HIT-family protein	R	11	10	0.91	0.1	5	0.45	1.2
SAR2388	2456044	2456535	hypothetical protein	R	11	10	0.91	0.1	1	0.09	0.3
SAR2587	2669941	2670429	hypothetical protein	S	11	10	0.91	0.1	7	0.64	2.5
SAR0731	766890	767189	hypothetical protein		10	9	0.90	0.1	3	0.30	0.3
SAR0863	905131	905487	hypothetical protein	P	10	9	0.90	0.1	2	0.20	0.0
gap1	872558	873568	glyceraldehyde 3-phosphate dehydrogenase 1	G	9	8	0.89	0.1	0	0.00	0.7
SAR1651	1718543	1719250	hypothetical protein	O	9	8	0.89	0.1	1	0.11	0.2
ppaC	2104848	2105783	manganese-dependent inorganic pyrophosphatase	C	9	8	0.89	0.1	0	0.00	0.7
SAR2223	2291334	2291684	hypothetical protein	S	9	8	0.89	0.1	3	0.33	0.4
SAR2465	2535763	2536707	CorA-like Mg2+ transporter protein	P	9	8	0.89	0.1	1	0.11	0.2
SAR2617	2700673	2701083	hypothetical protein	R	9	8	0.89	0.1	2	0.22	0.0
nrdR	1829334	1829804	NrdR family transcriptional regulator	K	8	7	0.88	0.1	0	0.00	0.7
SAR1962	2054712	2055026	hypothetical protein		8	7	0.88	0.1	0	0.00	0.7
ureE	2444029	2444481	urease accessory protein UreE	O	8	7	0.88	0.1	1	0.13	0.0
SAR2569	2649885	2650076	hypothetical protein		8	7	0.88	0.1	1	0.13	0.0
SAR0772	805133	807073	sulfatase	M	30	29	0.97	0.1	2	0.07	1.3
leuA	2207139	2208668	2-isopropylmalate synthase	E	30	29	0.97	0.1	8	0.27	0.3
opp-1D	2632976	2633791	oligopeptide transporter ATPase subunit	P	29	28	0.97	0.1	4	0.14	0.3
SAR2741	2847155	2847631	methionine sulfoxide reductase A	O	29	28	0.97	0.1	14	0.48	2.9
SAR1017	1058866	1060539	menaquinone biosynthesis bifunctional protein	H	64	64	1.00	0.1	10	0.16	0.5
SAR0811	851184	851420	hypothetical protein	S	7	6	0.86	0.1	1	0.14	0.0
SAR1183	1230367	1230768	hypothetical protein	S	7	6	0.86	0.1	1	0.14	0.0
SAR1333	1383249	1383437	hypothetical protein		7	6	0.86	0.1	1	0.14	0.0
SAR1592	1665482	1665919	hypothetical protein		7	6	0.86	0.1	3	0.43	0.8
efp	1679252	1679809	elongation factor P	J	7	6	0.86	0.1	0	0.00	0.4
czrA	2301090	2301410	zinc and cobalt transport repressor protein	K	7	6	0.86	0.1	2	0.29	0.2
SAR0561	612588	613250	deoxyadenosine kinase protein	F	27	26	0.96	0.1	1	0.04	1.7
nusA	1300799	1301974	transcription elongation factor NusA	K	27	26	0.96	0.1	9	0.33	0.8
SAR1834	1915805	1916596	hypothetical protein	M	27	26	0.96	0.1	2	0.07	1.0
SAR1983	2071236	2072549	Mur ligase family protein	M	27	26	0.96	0.1	8	0.30	0.5
secY	2397098	2398390	preprotein translocase subunit SecY	U	27	26	0.96	0.1	2	0.07	1.0
SAR2522	2597436	2598578	glycerate kinase	G	27	26	0.96	0.1	5	0.19	0.0
SAR0191	218652	219707	hypothetical protein	S	26	25	0.96	0.1	8	0.31	0.6
eutD	644909	645895	phosphotransacetylase	C	26	25	0.96	0.1	6	0.23	0.1
nuc	895661	896347	thermonuclease precursor	L	26	25	0.96	0.1	13	0.50	2.9
SAR0986	1030237	1031427	hypothetical protein	R	26	25	0.96	0.1	4	0.15	0.2
dnaJ	1722988	1724127	chaperone protein DnaJ	O	26	25	0.96	0.1	2	0.08	1.0
SAR0564	614544	615413	haloacid dehalogenase-like hydrolase	R	25	24	0.96	0.1	9	0.36	1.1
SAR1868	1955560	1956393	aldo/keto reductase family protein	R	24	23	0.96	0.1	8	0.33	0.7
SAR2513	2587092	2587784	6-carboxyhexanoate--CoA ligase	H	24	23	0.96	0.1	15	0.63	4.8
SAR0574	629493	630125	hexulose-6-phosphate synthase	G	6	5	0.83	0.1	1	0.17	0.0
SAR0673	720394	721011	hypothetical protein	P	6	5	0.83	0.1	0	0.00	0.5

Gene	Start	End	Product	COG	# Core BiPs	# Homo-plasies	Homoplasy rate	Poisson test: −log10(p)	# Non-synonymous BiPs	Non-synonymous probability	Binomial test: −log10(p)
mnhB	947176	947604	monovalent cation/H+ antiporter subunit B	P	6	5	0.83	0.1	0	0.00	0.5
frr	1289740	1290294	ribosome recycling factor	J	6	5	0.83	0.1	2	0.33	0.2
SAR2777	2883814	2884389	hypothetical protein		6	5	0.83	0.1	3	0.50	0.9
opuCD	2614669	2615364	glycine betaine/carnitine/choline ABC transporter permease protein	E	22	21	0.95	0.1	1	0.05	1.2
SAR0573	628658	629416	glucosamine-6-phosphate isomerase	G	21	20	0.95	0.1	5	0.24	0.1
ribE	1941878	1942510	riboflavin synthase subunit alpha	H	21	20	0.95	0.1	8	0.38	1.0
SAR0979	1023233	1024318	hypothetical protein	R	20	19	0.95	0.1	3	0.15	0.2
ilvC	2206105	2207109	ketol-acid reductoisomerase	H	20	19	0.95	0.1	2	0.10	0.5
SAR0679	727339	727764	hypothetical protein		5	4	0.80	0.1	4	0.80	2.1
SAR1041	1086123	1086386	hypothetical protein	F	5	4	0.80	0.1	2	0.40	0.5
sodA	1698789	1699388	superoxide dismutase	P	5	4	0.80	0.1	0	0.00	0.2
SAR1784	1857558	1857971	universal stress protein	T	5	4	0.80	0.1	2	0.40	0.5
SAR1946	2028075	2028545	SpoU rRNA methylase family protein	J	5	4	0.80	0.1	1	0.20	0.0
SAR2120	2181322	2181948	hypothetical protein	R	5	4	0.80	0.1	2	0.40	0.5
SAR2370	2440245	2440337	hypothetical protein		5	4	0.80	0.1	4	0.80	2.1
SAR2425	2494467	2494793	hypothetical protein	S	5	4	0.80	0.1	3	0.60	1.2
pgm	875783	877300	phosphoglyceromutase	G	44	43	0.98	0.1	3	0.07	1.8
SAR2127	2187200	2188159	fructokinase	G	41	40	0.98	0.1	21	0.51	4.6
SAR0820	862629	863540	hypothetical protein	R	18	17	0.94	0.1	1	0.06	0.8
SAR1637	1705340	1706017	hypothetical protein	R	18	17	0.94	0.1	6	0.33	0.6
SAR0243	284496	285551	zinc-binding dehydrogenase	R	39	38	0.97	0.1	16	0.41	2.3
SAR2474	2547903	2548349	MarR family regulatory protein	K	17	16	0.94	0.1	4	0.24	0.1
ddh	2689369	2690361	D-lactate dehydrogenase	R	17	16	0.94	0.1	6	0.35	0.6
SAR1268	1330136	1331680	(dimethylallyl)adenosine tRNA methylthiotransferase	J	38	37	0.97	0.1	6	0.16	0.3
purN	1091728	1092294	phosphoribosylglycinamide formyltransferase	F	16	15	0.94	0.1	6	0.38	0.9
SAR1981	2069865	2070278	hypothetical protein		16	15	0.94	0.1	13	0.81	6.2
SAR0266	310631	311545	ribokinase	G	36	35	0.97	0.1	11	0.31	0.7
SAR0405	440463	440870	hypothetical protein		4	3	0.75	0.1	1	0.25	0.0
SAR0663	709558	710055	hypothetical protein		4	3	0.75	0.1	0	0.00	0.2
saeR	795229	795915	response regulator protein	T	4	3	0.75	0.1	0	0.00	0.2
lspA	1217944	1218435	lipoprotein signal peptidase	U	4	3	0.75	0.1	1	0.25	0.0
SAR1600	1674556	1674786	exodeoxyribonuclease VII small subunit	L	4	3	0.75	0.1	1	0.25	0.0
SAR1822	1905252	1905563	thioredoxin	O	4	3	0.75	0.1	0	0.00	0.2
SAR1613	1685102	1686457	glycine dehydrogenase subunit 1	E	34	33	0.97	0.1	8	0.24	0.1
kdpD	2235309	2237966	sensor kinase protein	T	94	94	1.00	0.1	39	0.41	4.9
SAR0328	373430	373873	PTS transport system IIA component	G	33	32	0.97	0.1	9	0.27	0.4
murE	1033012	1034496	UDP-N-acetylmuramoylalanyl-D-glutamate--L-lysine ligase	M	33	32	0.97	0.1	10	0.30	0.7
SAR2541	2621574	2622926	carboxylesterase	I	58	57	0.98	0.1	28	0.48	5.2
SAR0782	819309	819809	7-cyano-7-deazaguanine reductase	R	31	30	0.97	0.1	2	0.06	1.3
SAR1786	1859142	1860197	metallopeptidase	E	31	30	0.97	0.1	11	0.35	1.1
SAR2602	2687501	2688307	glyoxalase/bleomycin resistance protein/dioxygenase superfamily protein	R	31	30	0.97	0.1	10	0.32	0.7
acsA	1889504	1891210	acetyl-CoA synthetase	I	51	50	0.98	0.1	14	0.27	0.5
coaD	1144102	1144584	phosphopantetheine adenylyltransferase	H	3	2	0.67	0.1	1	0.33	0.3
pyrH	1288999	1289721	uridylate kinase	F	3	2	0.67	0.1	0	0.00	0.0
SAR1376	1433402	1433590	4-oxalocrotonate tautomerase	R	3	2	0.67	0.1	1	0.33	0.3
SAR1416	1473529	1473798	acylphosphatase	C	3	2	0.67	0.1	1	0.33	0.3
icaD	2853276	2853581	intercellular adhesion protein D		3	2	0.67	0.1	2	0.67	0.9
thyA	1497785	1498741	thymidylate synthase	F	47	46	0.98	0.1	6	0.13	0.7
SAR0633	679844	681340	monovalent cation/H+ antiporter subunit D	P	72	71	0.99	0.1	12	0.17	0.4

Gene	Start	End	Product	COG	# Core BiPs	# Homo-plasies	Homoplasy rate	Poisson test: −log10(p)	# Non-synonymous BiPs	Non-synonymous probability	Binomial test: −log10(p)
SAR0478	512344	512661	hypothetical protein	S	2	1	0.50	0.1	0	0.00	0.0
rplL	589465	589833	50S ribosomal protein L7/L12	J	2	1	0.50	0.1	0	0.00	0.0
SAR1052	1098697	1098831	hypothetical protein		2	1	0.50	0.1	1	0.50	0.4
rbfA	1305102	1305452	ribosome-binding factor A	J	2	1	0.50	0.1	1	0.50	0.4
rpmG	1395854	1396003	50S ribosomal protein L33		2	1	0.50	0.1	1	0.50	0.4
SAR1351	1400413	1400652	hypothetical protein	S	2	1	0.50	0.1	0	0.00	0.0
SARs015	1588874	1589008	NA		2	1	0.50	0.1	0	0.00	0.0
SAR1696	1756666	1756926	hypothetical protein	S	2	1	0.50	0.1	0	0.00	0.0
SAR1809a	1887056	1887151	hypothetical protein		2	1	0.50	0.1	0	0.00	0.0
rplW	2405665	2405940	50S ribosomal protein L23	J	2	1	0.50	0.1	0	0.00	0.0
SAR2577	2658946	2659212	hypothetical protein		2	1	0.50	0.1	1	0.50	0.4
rnpA	2901794	2902141	ribonuclease P	J	2	1	0.50	0.1	0	0.00	0.0
SAR1254	1315658	1316923	hypothetical protein	R	28	26	0.93	0.1	10	0.36	1.0
SAR1954	2044169	2045458	glutamate-1-semialdehyde aminotransferase	H	43	41	0.95	0.2	6	0.14	0.6
SAR0768	801993	802601	para-aminobenzoate synthetase component	H	27	25	0.93	0.2	17	0.63	5.4
queA	1785724	1786749	S-adenosylmethioninetRNA ribosyltransferase-isomerase	J	27	25	0.93	0.2	9	0.33	0.8
SAR1878	1965275	1965757	lipoprotein		27	25	0.93	0.2	15	0.56	4.0
SAR1578	1649431	1650189	short chain dehydrogenase	R	42	40	0.95	0.2	17	0.40	2.3
papS	1562122	1563324	tRNA CCA-pyrophosphorylase	J	41	39	0.95	0.2	18	0.44	2.8
trpF	1441668	1442300	N-(5'-phosphoribosyl)anthranilate isomerase	E	26	24	0.92	0.2	14	0.54	3.5
dnaB	1827933	1829333	chromosome replication initiation/membrane attachment protein	L	40	38	0.95	0.2	12	0.30	0.7
SAR1947	2028550	2029677	iron-sulphur protein	C	40	38	0.95	0.2	9	0.23	0.1
tcaB	2509792	2511000	teicoplanin resistance associated membrane protein	G	40	38	0.95	0.2	11	0.28	0.5
SAR0626	674399	675328	hypothetical protein	S	25	23	0.92	0.2	6	0.24	0.1
pgk	873707	874897	phosphoglycerate kinase	G	25	23	0.92	0.2	4	0.16	0.2
SAR1266	1328748	1329614	2-oxoglutarate ferredoxin oxidoreductase subunit beta	C	25	23	0.92	0.2	7	0.28	0.3
ruvB	1786751	1787755	Holliday junction DNA helicase RuvB	L	25	23	0.92	0.2	3	0.12	0.5
SAR1927	2007283	2008506	transporter protein	U	56	54	0.96	0.2	11	0.20	0.1
SAR0749	783865	784329	hypothetical protein		14	12	0.86	0.2	5	0.36	0.7
msrA1	1429772	1430281	methionine sulfoxide reductase A	O	14	12	0.86	0.2	1	0.07	0.5
SAR1964	2056083	2056892	glycosyltransferase	M	14	12	0.86	0.2	3	0.21	0.0
SAR2600	2686382	2686816	MarR family regulatory protein	K	14	12	0.86	0.2	3	0.21	0.0
SAR2683	2770133	2770549	hypothetical protein		14	12	0.86	0.2	8	0.57	2.4
SAR1438	1496252	1497091	hypothetical protein	S	24	22	0.92	0.2	7	0.29	0.5
SAR1570	1643987	1644529	hypothetical protein	K	24	22	0.92	0.2	8	0.33	0.7
SAR1432	1491730	1493220	protease	M	53	51	0.96	0.2	21	0.40	2.6
fabD	1255798	1256724	malonyl CoA-acyl carrier protein transacylase	I	36	34	0.94	0.2	11	0.31	0.7
SAR2540	2619630	2621267	amino acid permease	E	36	34	0.94	0.2	9	0.25	0.3
SAR0880	917699	919096	hypothetical protein	O	23	21	0.91	0.2	2	0.09	0.7
sodM	149425	150024	superoxide dismutase	P	13	11	0.85	0.2	1	0.08	0.5
SAR1864	1953066	1953779	translaldolase	G	13	11	0.85	0.2	3	0.23	0.1
SAR2704	2797350	2797550	hypothetical protein		13	11	0.85	0.2	9	0.69	3.5
purK	1084291	1085415	phosphoribosylaminoimidazole carboxylase ATPase subunit	F	35	33	0.94	0.2	15	0.43	2.2
pepB	1011708	1013516	oligopeptidase	E	50	48	0.96	0.2	6	0.12	0.9
SAR1228	1282985	1283881	integrase/recombinase	L	22	20	0.91	0.2	9	0.41	1.4

Gene	Start	End	Product	COG	# Core BiPs	# Homo-plasies	Homoplasy rate	Poisson test: −log10(p)	# Non-synonymous BiPs	Non-synonymous probability	Binomial test: −log10(p)
SAR2480	2552668	2553321	response regulator	T	22	20	0.91	0.2	1	0.05	1.2
thrS	1824663	1826600	threonyl-tRNA synthetase	J	49	47	0.96	0.2	4	0.08	1.6
SAR0476	509964	510488	acetyltransferase (GNAT) family protein	R	12	10	0.83	0.2	3	0.25	0.1
SAR1859	1949432	1949902	DNA-binding protein	K	12	10	0.83	0.2	6	0.50	1.6
holA	1731996	1732970	DNA polymerase III subunit delta	L	21	19	0.90	0.2	6	0.29	0.4
modC	2431735	2432340	molybdenum transport ATP-binding protein	P	21	19	0.90	0.2	7	0.33	0.7
SAR2394	2465082	2466029	hypothetical protein	K	21	19	0.90	0.2	2	0.10	0.5
SAR0664	710511	711578	hypothetical protein	S	32	30	0.94	0.2	5	0.16	0.3
SAR0142	156297	157112	binding-protein-dependent transport systems membrane component	P	20	18	0.90	0.2	7	0.35	0.8
SAR0396	430210	430836	hypothetical protein		20	18	0.90	0.2	7	0.35	0.8
xpt	441383	441961	xanthine phosphoribosyltransferase	F	20	18	0.90	0.2	4	0.20	0.0
sucC	1273098	1274264	succinyl-CoA synthetase subunit beta	C	20	18	0.90	0.2	5	0.25	0.1
SAR0655	701540	702769	Na+ dependent nucleoside transporter	F	31	29	0.94	0.2	3	0.10	0.9
ribA	1940686	1941867	riboflavin biosynthesis protein	H	31	29	0.94	0.2	11	0.35	1.1
SAR2217	2284742	2285602	acetyltransferase	J	31	29	0.94	0.2	4	0.13	0.4
yabJ	535171	535551	regulatory protein	J	11	9	0.82	0.2	5	0.45	1.2
dnaI	1827013	1827933	primosomal protein DnaI	L	11	9	0.82	0.2	1	0.09	0.3
SAR2254	2329623	2330432	hypothetical protein	S	11	9	0.82	0.2	2	0.18	0.0
SAR0891	927956	928735	haloacid dehalogenase-like hydrolase	G	19	17	0.89	0.2	0	0.00	1.7
SAR0900	935388	936452	pyridine nucleotide-disulphide oxidoreductase	C	19	17	0.89	0.2	0	0.00	1.7
glpP	1337522	1338055	glycerol uptake operon antiterminator regulatory protein	K	19	17	0.89	0.2	2	0.11	0.4
SAR2422	2491672	2492358	ribose-5-phosphate isomerase A	G	19	17	0.89	0.2	2	0.11	0.4
SAR2429	2498414	2499022	3-methylpurine glycosylase	L	19	17	0.89	0.2	6	0.32	0.6
SAR2023	2114645	2115517	ABC transporter ATP-binding protein	V	18	16	0.89	0.2	5	0.28	0.2
SAR1005	1050874	1051164	hypothetical protein		10	8	0.80	0.2	4	0.40	0.6
SAR1085	1130323	1130805	hypothetical protein		10	8	0.80	0.2	3	0.30	0.3
SAR1270	1332073	1332564	hypothetical protein	S	10	8	0.80	0.2	4	0.40	0.6
SAR1327	1378021	1378218	hypothetical protein		10	8	0.80	0.2	2	0.20	0.0
SAR1615	1687718	1688242	shikimate kinase	E	10	8	0.80	0.2	3	0.30	0.3
SAR1862	1951186	1951821	CAAX amino terminal protease family protein		10	8	0.80	0.2	3	0.30	0.3
fabZ	2256572	2257012	(3R)-hydroxymyristoyl-ACP dehydratase	I	10	8	0.80	0.2	0	0.00	0.9
SAR0565	615433	615999	hypothetical protein	R	17	15	0.88	0.2	2	0.12	0.3
SARs002	15939	16037	NA		1	0	0.00	0.2	0	0.00	0.0
SAR0269a	314320	314433	hypothetical protein		1	0	0.00	0.2	1	1.00	0.7
rpsR	408870	409112	30S ribosomal protein S18	J	1	0	0.00	0.2	0	0.00	0.0
spoVG	535624	535926	regulatory protein SpoVG	M	1	0	0.00	0.2	0	0.00	0.0
rpmG	586205	586348	50S ribosomal protein L33	J	1	0	0.00	0.2	0	0.00	0.0
cspC	896704	896904	cold shock protein	K	1	0	0.00	0.2	1	1.00	0.7
SAR0855	899828	900064	hypothetical protein		1	0	0.00	0.2	0	0.00	0.0
SARs007	908405	908508	NA		1	0	0.00	0.2	0	0.00	0.0
SAR0893	930138	930290	hypothetical protein		1	0	0.00	0.2	1	1.00	0.7
spxA	1009068	1009463	Spx family transcriptional regulator	P	1	0	0.00	0.2	1	1.00	0.7
rpmB	1249123	1249311	50S ribosomal protein L28	J	1	0	0.00	0.2	0	0.00	0.0
SAR1263	1325661	1325831	hypothetical protein		1	0	0.00	0.2	0	0.00	0.0
SAR1279	1344837	1345070	hypothetical protein	R	1	0	0.00	0.2	1	1.00	0.7
SAR1353	1403039	1403281	hypothetical protein	S	1	0	0.00	0.2	0	0.00	0.0
SAR1415	1473059	1473367	hypothetical protein	S	1	0	0.00	0.2	0	0.00	0.0
rnpB	1549260	1549607	NA		1	0	0.00	0.2	0	0.00	0.0
SAR1603	1676582	1676944	hypothetical protein	S	1	0	0.00	0.2	0	0.00	0.0
rpsU	1719470	1719646	30S ribosomal protein S21	J	1	0	0.00	0.2	0	0.00	0.0

Gene	Start	End	Product	COG	# Core BiPs	# Homo-plasies	Homoplasy rate	Poisson test: −log10(p)	# Non-synonymous BiPs	Non-synonymous probability	Binomial test: −log10(p)
rpsT	1731700	1731951	30S ribosomal protein S20	J	1	0	0.00	0.2	0	0.00	0.0
SARs016	1759722	1759920	NA		1	0	0.00	0.2	0	0.00	0.0
gatC	2082481	2082783	aspartyl/glutamyl-tRNA amidotransferase subunit C	J	1	0	0.00	0.2	0	0.00	0.0
SAR2016	2109456	2109629	hypothetical protein		1	0	0.00	0.2	1	1.00	0.7
SAR2027a	2118144	2118242	hypothetical protein		1	0	0.00	0.2	0	0.00	0.0
SAR2183b	2254140	2254412	hypothetical protein		1	0	0.00	0.2	1	1.00	0.7
deoC2	2293279	2293941	deoxyribose-phosphate aldolase	F	1	0	0.00	0.2	1	1.00	0.7
rpsM	2395490	2395855	30S ribosomal protein S13	J	1	0	0.00	0.2	0	0.00	0.0
infA	2396023	2396241	translation initiation factor IF-1	J	1	0	0.00	0.2	0	0.00	0.0
rplX	2401719	2402036	50S ribosomal protein L24	J	1	0	0.00	0.2	0	0.00	0.0
SAR2384	2452192	2452386	hypothetical protein		1	0	0.00	0.2	1	1.00	0.7
fnbA	2660837	2663734	fibronectin-binding protein precursor		1	0	0.00	0.2	1	1.00	0.7
SAR2689	2776453	2776665	hypothetical protein		1	0	0.00	0.2	0	0.00	0.0
rpmH	2902268	2902405	50S ribosomal protein L34		1	0	0.00	0.2	0	0.00	0.0
ipk	533468	534316	4-diphosphocytidyl-2-C-methyl-D-erythritol kinase	I	16	14	0.88	0.2	6	0.38	0.9
SAR1874	1961839	1962318	hypothetical protein	F	16	14	0.88	0.2	8	0.50	2.0
SAR0610	659226	659702	acetyltransferase	R	9	7	0.78	0.2	3	0.33	0.4
sdhC	1169355	1169969	succinate dehydrogenase cytochrome b558	C	9	7	0.78	0.2	1	0.11	0.2
SAR2002	2094814	2095137	hypothetical protein		9	7	0.78	0.2	2	0.22	0.0
SAR2529	2604756	2606834	sodium/hydrogen exchanger family protein	P	74	71	0.96	0.2	14	0.19	0.2
SAR0734	768474	768932	hypothetical protein	S	8	6	0.75	0.2	5	0.63	1.9
SAR0970	1014805	1015170	hypothetical protein	R	8	6	0.75	0.2	3	0.38	0.4
SAR0972	1016053	1016400	hypothetical protein		8	6	0.75	0.2	5	0.63	1.9
SAR1393	1449844	1450617	oligopeptide transporter ATPase	P	8	6	0.75	0.2	3	0.38	0.4
atpI	2266082	2266435	ATP synthase protein I		8	6	0.75	0.2	6	0.75	2.7
sasF	2826250	2828133	surface anchored protein	R	70	67	0.96	0.2	45	0.64	13.9
secF	1781729	1784008	bifunctional preprotein translocase subunit SecD/SecF	U	51	48	0.94	0.2	8	0.16	0.4
SAR0469	496343	497461	hypothetical protein	S	39	36	0.92	0.2	10	0.26	0.3
pbpA	1199586	1201820	penicillin-binding protein 1	M	50	47	0.94	0.2	9	0.18	0.1
trpB	1442293	1443507	tryptophan synthase subunit beta	E	50	47	0.94	0.2	10	0.20	0.0
SAR1854	1944041	1945543	hypothetical protein	S	50	47	0.94	0.2	19	0.38	2.1
SAR0997	1044539	1045525	lipoate-protein ligase A	H	28	25	0.89	0.2	4	0.14	0.3
norA	782414	783580	fluoroquinolone resistance protein	G	38	35	0.92	0.2	4	0.11	0.9
lacC	2368498	2369430	tagatose-6-phosphate kinase	G	38	35	0.92	0.2	7	0.18	0.1
SAR1930a	2010299	2010487	hypothetical protein		7	5	0.71	0.2	6	0.86	3.3
SAR2512	2586614	2587081	hypothetical protein		7	5	0.71	0.2	1	0.14	0.0
SAR1698	1759962	1762439	hypothetical protein	L	91	88	0.97	0.2	18	0.20	0.1
SAR0671	717138	717899	ABC transporter	V	48	45	0.94	0.2	4	0.08	1.5
SAR0905	941056	942372	transporter protein	R	27	24	0.89	0.2	3	0.11	0.6
SAR1395	1451427	1452413	oligopeptide ABC transporter permease	P	27	24	0.89	0.2	7	0.26	0.2
SAR2459	2529751	2530755	zinc-binding dehydrogenase	R	27	24	0.89	0.2	14	0.52	3.3
SAR2658	2746004	2746561	TetR family regulatory protein	K	27	24	0.89	0.2	8	0.30	0.5
SAR1144	1189659	1191215	hypothetical protein	S	47	44	0.94	0.2	8	0.17	0.2
SAR0975	1017875	1018729	RNA pseudouridylate synthase	J	36	33	0.92	0.2	11	0.31	0.7
ptsI	1102894	1104612	phosphoenolpyruvate-protein phosphotransferase	G	46	43	0.93	0.3	8	0.17	0.2
SAR1205	1254819	1255805	glycerol-3-phosphate acyltransferase PlsX	I	26	23	0.88	0.3	9	0.35	0.8
SAR1667	1735786	1736463	hypothetical protein	L	26	23	0.88	0.3	16	0.62	5.0
accC	1676959	1678314	acetyl-CoA carboxylase biotin carboxylase subunit	I	35	32	0.91	0.3	6	0.17	0.2
SAR2350	2423141	2424352	transporter protein	G	35	32	0.91	0.3	13	0.37	1.4

Gene	Start	End	Product	COG	# Core BiPs	# Homo-plasies	Homoplasy rate	Poisson test: −log10(p)	# Non-synonymous BiPs	Non-synonymous probability	Binomial test: −log10(p)
pmi	2819156	2820094	mannose-6-phosphate isomerase	G	34	31	0.91	0.3	13	0.38	1.5
SAR1222	1274286	1275194	succinyl-CoA synthetase subunit alpha	C	24	21	0.88	0.3	5	0.21	0.0
xseA	1674779	1676116	exodeoxyribonuclease VII large subunit	L	24	21	0.88	0.3	2	0.08	0.9
SAR2656	2744734	2745555	hypothetical protein	G	24	21	0.88	0.3	13	0.54	3.4
SAR0628	675762	675965	hypothetical protein		6	4	0.67	0.3	1	0.17	0.0
SAR0642	688716	689552	ABC transporter permease	P	6	4	0.67	0.3	1	0.17	0.0
mnhG	944238	944594	monovalent cation/H+ antiporter subunit G	P	6	4	0.67	0.3	1	0.17	0.0
SAR1243	1301995	1302279	hypothetical protein	K	6	4	0.67	0.3	0	0.00	0.5
rpsJ	2407280	2407588	30S ribosomal protein S10	J	6	4	0.67	0.3	1	0.17	0.0
SAR2343	2415687	2415851	hypothetical protein		6	4	0.67	0.3	1	0.17	0.0
sspA	1064009	1065082	glutamyl endopeptidase precursor	E	32	29	0.91	0.3	5	0.16	0.3
cudT	2781389	2783011	choline transporter	M	32	29	0.91	0.3	0	0.00	3.1
srtB	1153983	1154717	sortase B	S	23	20	0.87	0.3	13	0.57	3.6
acuA	1891379	1892011	acetoin utilization protein		23	20	0.87	0.3	12	0.52	2.9
SAR0773	807351	809228	ABC transporter ATP-binding protein	R	72	68	0.94	0.3	18	0.25	0.3
SAR0825	868764	869666	hypothetical protein	R	29	26	0.90	0.3	10	0.34	1.0
SAR1391	1448569	1449027	hypothetical protein		29	26	0.90	0.3	13	0.45	2.3
SAR1445	1501482	1502186	hypothetical protein	S	29	26	0.90	0.3	3	0.10	0.8
SAR2708	2801229	2801990	esterase	R	29	26	0.90	0.3	13	0.45	2.3
cysJ	2788692	2790572	sulfite reductase [NADPH] flavoprotein alpha-component	P	59	55	0.93	0.3	20	0.34	1.6
SAR1027	1072751	1073185	acetyltransferase (GNAT) family protein	R	21	18	0.86	0.3	7	0.33	0.7
SAR1729	1792403	1793245	rod shape-determining protein MreC	M	21	18	0.86	0.3	5	0.24	0.1
SAR1932	2011797	2012759	peptidyl-prolyl cis-isomerase	O	21	18	0.86	0.3	2	0.10	0.5
SAR1171	1216898	1217695	glyoxalase/bleomycin resistance protein/dioxygenase superfamily protein	R	20	17	0.85	0.3	5	0.25	0.1
recX	2055004	2055822	recombination regulator RecX	R	20	17	0.85	0.3	5	0.25	0.1
SAR2620	2705608	2706297	hypothetical protein	M	20	17	0.85	0.3	3	0.15	0.2
SAR0465	494106	494375	hypothetical protein		5	3	0.60	0.3	1	0.20	0.0
rpsL	598631	599044	30S ribosomal protein S12	J	5	3	0.60	0.3	1	0.20	0.0
SAR0795	832615	832935	hypothetical protein		5	3	0.60	0.3	1	0.20	0.0
SAR0853	899027	899287	hypothetical protein		5	3	0.60	0.3	1	0.20	0.0
SAR0935	977622	977930	hypothetical protein	R	5	3	0.60	0.3	0	0.00	0.2
SAR1413	1472090	1472491	hypothetical protein		5	3	0.60	0.3	1	0.20	0.0
perR	2042134	2042580	peroxide operon regulator	P	5	3	0.60	0.3	1	0.20	0.0
SAR2667	2753793	2754071	hypothetical protein		5	3	0.60	0.3	2	0.40	0.5
SAR0925	962453	963028	hypothetical protein	S	19	16	0.84	0.3	6	0.32	0.6
SAR1004	1050145	1050786	ABC transporter ATP-binding protein	V	19	16	0.84	0.3	10	0.53	2.6
SAR1081	1126894	1127721	inositol monophosphatase family protein	G	19	16	0.84	0.3	3	0.16	0.1
pbp2	1552123	1554273	penicillin-binding protein 2	M	42	38	0.90	0.3	11	0.26	0.3
SAR2534	2612975	2614375	transport protein	G	42	38	0.90	0.3	10	0.24	0.2
sigH	585521	586090	hypothetical protein	K	18	15	0.83	0.3	9	0.50	2.2
SAR0969	1013976	1014782	hypothetical protein	Q	18	15	0.83	0.3	4	0.22	0.0
SAR1402	1458802	1459785	phosphate-binding lipoprotein	P	18	15	0.83	0.3	3	0.17	0.1
thl	395237	396418	acetyl-CoA acetyltransferase	I	39	35	0.90	0.3	8	0.21	0.0
SAR0569	624677	626167	glycosyl transferase	M	39	35	0.90	0.3	10	0.26	0.3
gpsA	1576246	1577244	NAD(P)H-dependent glycerol-3-phosphate dehydrogenase	C	39	35	0.90	0.3	5	0.13	0.6
SAR0510	547990	549285	hypothetical protein	D	48	44	0.92	0.3	19	0.40	2.4
SAR2288	2371775	2372506	NAD-dependent deacetylase	K	48	44	0.92	0.3	17	0.35	1.7
SAR2277	2360372	2361379	zinc-binding dehydrogenase	R	47	43	0.91	0.3	16	0.34	1.3
SAR0796	833360	834484	glycerate kinase	G	37	33	0.89	0.3	9	0.24	0.2
accB	1678314	1678778	biotin carboxyl carrier protein of acetyl-CoA carboxylase	I	16	13	0.81	0.3	3	0.19	0.0

Gene	Start	End	Product	COG	# Core BiPs	# Homo-plasies	Homoplasy rate	Poisson test: −log10(p)	# Non-synonymous BiPs	Non-synonymous probability	Binomial test: −log10(p)
SAR2641	2728770	2729924	aminotransferase	E	44	40	0.91	0.3	13	0.30	0.7
SAR1428	1488791	1488964	hypothetical protein		4	2	0.50	0.3	4	1.00	2.7
SAR1713	1775632	1776084	D-tyrosyl-tRNA(Tyr) deacylase	J	4	2	0.50	0.3	1	0.25	0.0
rplT	1821355	1821711	50S ribosomal protein L20	J	4	2	0.50	0.3	0	0.00	0.2
SARs018	1826732	1826959	NA		4	2	0.50	0.3	0	0.00	0.2
SAR1938	2020544	2021008	DNA-binding protein	K	4	2	0.50	0.3	0	0.00	0.2
SAR2403	2473169	2473360	hypothetical protein		4	2	0.50	0.3	3	0.75	1.5
SAR0583	636743	637012	hypothetical protein		11	8	0.73	0.3	6	0.55	1.8
SAR0760	795890	796363	hypothetical protein		11	8	0.73	0.3	2	0.18	0.0
SAR2439	2508483	2509037	TetR family regulatory protein		11	8	0.73	0.3	3	0.27	0.1
SAR1620	1689925	1690995	hypothetical protein	U	35	31	0.89	0.3	11	0.31	0.8
SAR0756	792148	792987	aldo/keto reductase family protein	R	27	23	0.85	0.3	11	0.41	1.5
SAR0801	838769	839824	glycosyl transferase	M	27	23	0.85	0.3	1	0.04	1.7
SAR0327	373144	373428	PTS transporter	G	15	12	0.80	0.4	4	0.27	0.3
SAR1462	1554823	1555164	hypothetical protein		15	12	0.80	0.4	10	0.67	3.7
SAR0672	717889	719778	ABC transporter permease		93	88	0.95	0.4	10	0.11	2.0
SAR0619	667843	668793	FecCD transport family protein	P	33	29	0.88	0.4	9	0.27	0.4
SAR0883	921165	922232	dioxygenase	R	33	29	0.88	0.4	11	0.33	0.9
hrcA	1726822	1727799	heat-inducible transcription repressor	K	26	22	0.85	0.4	10	0.38	1.3
accD	1848856	1849713	acetyl-CoA carboxylase subunit beta	I	26	22	0.85	0.4	8	0.31	0.6
SAR1184	1231031	1232728	hypothetical protein	K	58	53	0.91	0.4	17	0.29	0.8
SAR0112	121172	121645	hypothetical protein		10	7	0.70	0.4	4	0.40	0.6
SAR0391	427672	427935	hypothetical protein		10	7	0.70	0.4	2	0.20	0.0
SAR0562	613243	613860	deoxyadenosine kinase protein	F	10	7	0.70	0.4	1	0.10	0.2
SAR0627	675521	675745	hypothetical protein		10	7	0.70	0.4	2	0.20	0.0
mreB	1700061	1700924	ABC transporter permease	P	10	7	0.70	0.4	3	0.30	0.3
tpx	1864872	1865366	thiol peroxidase	O	14	11	0.79	0.4	6	0.43	1.0
recG	1251979	1254039	ATP-dependent DNA helicase RecG	L	65	60	0.92	0.4	18	0.28	0.6
SAR1049	1095324	1096145	cobalt transport protein	P	31	27	0.87	0.4	8	0.26	0.3
SAR2527	2602118	2602924	hypothetical protein	R	31	27	0.87	0.4	15	0.48	2.9
SAR2121	2182309	2183094	carbon-nitrogen hydrolase	R	30	26	0.87	0.4	14	0.47	2.6
oppB	991904	992830	oligopeptide ABC transporter permease protein	P	13	10	0.77	0.4	1	0.08	0.5
fabG	1256711	1257451	3-oxoacyl-[acyl-carrier protein] reductase	R	13	10	0.77	0.4	3	0.23	0.1
SAR1396	1452717	1453061	hypothetical protein		13	10	0.77	0.4	0	0.00	1.1
clpB	980527	983136	ATPase subunit of an ATP-dependent protease	O	52	47	0.90	0.4	8	0.15	0.4
nrdF	824083	825054	ribonucleotide-diphosphate reductase subunit beta	F	29	25	0.86	0.4	4	0.14	0.3
SAR2218	2286026	2286829	pantothenate kinase	H	23	19	0.83	0.4	3	0.13	0.3
SAR1431	1490820	1491329	acetyltransferase	J	9	6	0.67	0.4	1	0.11	0.2
SAR1443	1500780	1501031	hypothetical protein		9	6	0.67	0.4	3	0.33	0.4
tag	1807616	1808176	DNA-3-methyladenine glycosylase I	L	9	6	0.67	0.4	4	0.44	1.0
pcrB	2090185	2090877	geranylgeranylglyceryl phosphate synthase-like protein	R	9	6	0.67	0.4	5	0.56	1.6
SAR2371	2440413	2441318	hypothetical protein	E	50	45	0.90	0.4	10	0.20	0.0
SAR2342	2414646	2415554	hypothetical protein	R	28	24	0.86	0.4	9	0.32	0.8
SAR0668	714345	715268	hypothetical protein	G	22	18	0.82	0.4	4	0.18	0.0
SAR0732	767269	767811	acetyltransferase	J	22	18	0.82	0.4	11	0.50	2.5
SAR1807	1883280	1884185	transglycosylase	M	22	18	0.82	0.4	7	0.32	0.5
rlp	231906	233681	RGD-containing lipoprotein	E	49	44	0.90	0.4	12	0.24	0.2
SAR1956	2047036	2048772	ABC transporter ATP-binding protein	V	49	44	0.90	0.4	9	0.18	0.1
SAR2437	2505765	2507702	transport protein	G	110	104	0.95	0.4	14	0.13	1.6
SAR1958	2049908	2050945	HhH-GPD superfamily base excision DNA	L	41	36	0.88	0.4	14	0.34	1.3

Gene	Start	End	Product	COG	# Core BiPs	# Homo-plasies	Homoplasy rate	Poisson test: −log10(p)	# Non-synonymous BiPs	Non-synonymous probability	Binomial test: −log10(p)
			repair protein								
SAR0624	672040	672828	esterase	R	12	9	0.75	0.4	4	0.33	0.5
SAR0816	858927	859412	acetyltransferase	R	12	9	0.75	0.4	4	0.33	0.5
SAR1125	1174233	1174736	hypothetical protein	R	12	9	0.75	0.4	3	0.25	0.1
SAR1363	1415605	1416072	hypothetical protein	R	12	9	0.75	0.4	4	0.33	0.5
SAR1649	1716821	1717519	hypothetical protein		12	9	0.75	0.4	4	0.33	0.5
gidB	2897608	2898327	16S rRNA methyltransferase GidB	M	12	9	0.75	0.4	6	0.50	1.6
trpC	1440886	1441668	indole-3-glycerol-phosphate synthase	E	40	35	0.88	0.4	13	0.33	0.9
thiI	1866278	1867501	thiamine biosynthesis protein ThiI	H	40	35	0.88	0.4	3	0.08	1.5
aspS	1771256	1773022	aspartyl-tRNA synthetase	J	47	42	0.89	0.4	3	0.06	1.9
carB	1225102	1228275	carbamoyl phosphate synthase large subunit	F	83	77	0.93	0.4	13	0.16	0.6
SAR2272	2354830	2356806	hypothetical protein	Q	83	77	0.93	0.4	34	0.41	4.2
SARs001	12491	12722	NA		3	1	0.33	0.4	0	0.00	0.0
rplK	587328	587750	50S ribosomal protein L11	J	3	1	0.33	0.4	2	0.67	0.9
sdrC	616430	619150	surface anchored protein		3	1	0.33	0.4	2	0.67	0.9
nrdI	821497	821895	ribonucleotide reductase stimulatory protein	F	3	1	0.33	0.4	1	0.33	0.3
dltC	932992	933228	D-alanine--poly(phosphoribitol) ligase subunit 2	Q	3	1	0.33	0.4	0	0.00	0.0
SAR0948	991290	991661	hypothetical protein		3	1	0.33	0.4	2	0.67	0.9
SAR0996	1044231	1044458	hypothetical protein		3	1	0.33	0.4	1	0.33	0.3
SAR1082	1127875	1128066	hypothetical protein		3	1	0.33	0.4	2	0.67	0.9
SAR1109a	1155096	1155203	hypothetical protein		3	1	0.33	0.4	1	0.33	0.3
SARs020	1943687	1943822	NA		3	1	0.33	0.4	0	0.00	0.0
SARs023	2253957	2254059	NA		3	1	0.33	0.4	0	0.00	0.0
rplP	2402958	2403392	50S ribosomal protein L16	J	3	1	0.33	0.4	0	0.00	0.0
SAR2639	2727453	2727659	heavy-metal-associated protein	P	3	1	0.33	0.4	1	0.33	0.3
SAR0767	800842	801993	para-aminobenzoate synthase component	H	39	34	0.87	0.4	9	0.23	0.1
rpoC	594519	598142	DNA-directed RNA polymerase subunit beta'	K	72	66	0.92	0.4	3	0.04	4.1
SAR0643	689546	690289	ABC transporter ATP-binding protein	P	8	5	0.63	0.4	2	0.25	0.2
SAR1166	1210793	1211083	hypothetical protein		8	5	0.63	0.4	4	0.50	1.2
apt	1778713	1779231	adenine phosphoribosyltransferase	F	8	5	0.63	0.4	3	0.38	0.4
SAR2351	2424454	2424804	hypothetical protein	K	8	5	0.63	0.4	3	0.38	0.4
SAR1265	1326987	1328747	pyruvate flavodoxin/ferredoxin oxidoreductase	C	38	33	0.87	0.4	11	0.29	0.6
SAR2160	2227993	2228484	hypothetical protein	S	20	16	0.80	0.4	10	0.50	2.4
atpD	2259958	2261370	F0F1 ATP synthase subunit beta	C	20	16	0.80	0.4	3	0.15	0.2
fhs	1887397	1889064	formate--tetrahydrofolate ligase	F	44	39	0.89	0.4	9	0.20	0.0
SAR2171	2243815	2245017	hypothetical protein	D	37	32	0.86	0.4	4	0.11	0.8
pyn	2291698	2292999	pyrimidine-nucleoside phosphorylase	F	37	32	0.86	0.4	11	0.30	0.6
citC	1840402	1841670	isocitrate dehydrogenase	C	36	31	0.86	0.4	5	0.14	0.5
SAR2413	2481886	2482767	short chain dehydrogenase	R	36	31	0.86	0.4	12	0.33	1.0
SAR2348	2421781	2422545	hypothetical protein		19	15	0.79	0.4	9	0.47	2.0
SAR0774	809240	811021	ATP-dependent DNA helicase	L	73	67	0.92	0.4	8	0.11	1.5
SAR2260	2341186	2342529	transport protein	G	34	29	0.85	0.5	3	0.09	1.0
SAR2007	2098924	2100000	oxygenase	P	18	14	0.78	0.5	11	0.61	3.5
SAR2024	2115514	2116194	hypothetical protein		18	14	0.78	0.5	8	0.44	1.4
bioB	2588888	2589895	biotin synthase	H	54	48	0.89	0.5	5	0.09	1.5
hpt	549290	549829	hypoxanthine phosphoribosyltransferase	F	7	4	0.57	0.5	2	0.29	0.2
SAR1669	1737231	1737584	hypothetical protein	S	7	4	0.57	0.5	1	0.14	0.0
SAR2133	2194051	2194686	redox-sensing transcriptional repressor Rex	R	7	4	0.57	0.5	1	0.14	0.0
atpG	2261392	2262258	F0F1 ATP synthase subunit gamma	C	7	4	0.57	0.5	1	0.14	0.0
moaE	2427268	2427714	molybdopterin-synthase large subunit	H	7	4	0.57	0.5	1	0.14	0.0

Gene	Start	End	Product	COG	# Core BiPs	# Homo-plasies	Homoplasy rate	Poisson test: −log10(p)	# Non-synonymous BiPs	Non-synonymous probability	Binomial test: −log10(p)
SAR0791	829563	829877	hypothetical protein	S	17	13	0.76	0.5	2	0.12	0.3
SAR0802	839988	840629	hypothetical protein	S	17	13	0.76	0.5	5	0.29	0.4
SAR2202	2269674	2270198	hypothetical protein	S	17	13	0.76	0.5	6	0.35	0.6
SAR2412	2480830	2481399	hypothetical protein		17	13	0.76	0.5	12	0.71	4.7
SAR2365	2435066	2435836	acetyltransferase (GNAT) family protein	R	32	27	0.84	0.5	13	0.41	1.8
SAR2229	2296592	2297965	hypothetical protein	S	31	26	0.84	0.5	8	0.26	0.3
SAR2262	2343409	2344596	UTP--glucose-1-phosphate uridylyltransferase	G	31	26	0.84	0.5	8	0.26	0.3
SAR0977	1020145	1021989	cation transport protein	P	50	44	0.88	0.5	9	0.18	0.1
bfmB	1666269	1667543	lipoamide acyltransferase component of branched-chain alpha-keto acid dehydrogenase complex	C	50	44	0.88	0.5	16	0.32	1.1
SAR1463	1555169	1555828	endonuclease	L	16	12	0.75	0.5	4	0.25	0.1
modB	2432341	2433012	molybdenum ABC transporter permease protein	P	16	12	0.75	0.5	5	0.31	0.4
SAR2470	2543165	2543521	hypothetical protein		16	12	0.75	0.5	2	0.13	0.3
pdhC	1115094	1116386	branched-chain alpha-keto acid dehydrogenase subunit E2	C	30	25	0.83	0.5	7	0.23	0.1
SAR1576	1648043	1648951	aldo/keto reductase family protein	C	30	25	0.83	0.5	15	0.50	3.3
SAR1060	1106638	1107657	hypothetical protein	C	29	24	0.83	0.5	7	0.24	0.2
hslU	1284489	1285892	ATP-dependent protease ATP-binding subunit HslU	O	29	24	0.83	0.5	5	0.17	0.1
SAR2610	2694594	2695493	L-serine dehydratase, alpha chain	E	29	24	0.83	0.5	5	0.17	0.1
SAR2168	2239017	2240537	helicase	L	47	41	0.87	0.5	5	0.11	1.1
era	1713441	1714340	GTP-binding protein Era	R	24	19	0.79	0.5	3	0.13	0.3
SAR1490	1585483	1586862	DEAD/DEAH box helicase family protein	L	69	62	0.90	0.5	16	0.23	0.2
SAR2269	2350714	2351784	hypothetical protein	E	40	34	0.85	0.5	13	0.33	0.9
mreA	1700966	1701751	ABC transporter ATP-binding protein	P	15	11	0.73	0.5	4	0.27	0.3
SAR1114	1161508	1161774	hypothetical protein	S	6	3	0.50	0.5	2	0.33	0.2
moaD	2427029	2427262	molybdopterin-synthase small subunit	H	6	3	0.50	0.5	4	0.67	1.7
cudB	2776943	2778652	choline dehydrogenase	E	67	60	0.90	0.5	6	0.09	2.0
SAR2679	2764387	2765091	alpha-acetolactate decarboxylase	Q	28	23	0.82	0.5	9	0.32	0.8
capN	181983	182870	capsular polysaccharide synthesis enzyme	G	39	33	0.85	0.5	17	0.44	2.6
scrA	2536793	2538235	PTS system, sucrose-specific IIBC component	G	39	33	0.85	0.5	7	0.18	0.2
SAR0559	610462	611538	branched-chain amino acid aminotransferase	H	45	39	0.87	0.5	10	0.22	0.1
SAR0571	627340	628005	hypothetical protein	S	23	18	0.78	0.5	8	0.35	0.9
opuCB	2616322	2616957	glycine betaine/carnitine/choline ABC transporter permease protein	E	23	18	0.78	0.5	5	0.22	0.0
SAR0637	682874	684916	sodium/hydrogen exchanger family protein	P	44	38	0.86	0.5	3	0.07	1.8
gntP	2664202	2665560	gluconate permease	E	38	32	0.84	0.5	5	0.13	0.5
lysS	556960	558447	lysyl-tRNA synthetase	J	27	22	0.81	0.5	5	0.19	0.0
SAR1849	1939086	1940087	proline dehydrogenase	E	27	22	0.81	0.5	9	0.33	0.8
SAR2364	2434098	2434895	formate dehydrogenase accessory protein	C	27	22	0.81	0.5	5	0.19	0.0
SAR1129	1176949	1177455	hypothetical protein		22	17	0.77	0.5	2	0.09	0.7
SAR0757	793119	794102	glucosyl transferase	M	37	31	0.84	0.5	10	0.27	0.4
SAR2450	2519237	2519692	hypothetical protein		14	10	0.71	0.5	7	0.50	1.8
SAR2389	2456894	2457847	glycerate dehydrogenase	R	26	21	0.81	0.5	4	0.15	0.2
SAR0482	521423	522760	Orn/Lys/Arg decarboxylase family protein	E	42	36	0.86	0.5	18	0.43	2.7
SAR1341	1390960	1391763	haloacid dehalogenase-like hydrolase	R	36	30	0.83	0.5	10	0.28	0.4
SAR395a	430027	430134	hypothetical protein		2	0	0.00	0.6	2	1.00	1.3
veg	532894	533157	hypothetical protein	S	2	0	0.00	0.6	1	0.50	0.4
SAR0745	780691	780975	hypothetical protein		2	0	0.00	0.6	2	1.00	1.3

Gene	Start	End	Product	COG	# Core BiPs	# Homo-plasies	Homoplasy rate	Poisson test: −log10(p)	# Non-synonymous BiPs	Non-synonymous probability	Binomial test: −log10(p)
SAR1064	1110497	1110715	hypothetical protein	S	2	0	0.00	0.6	1	0.50	0.4
rpmF	1146549	1146722	50S ribosomal protein L32	J	2	0	0.00	0.6	0	0.00	0.0
SAR1156	1199204	1199605	cell division protein	D	2	0	0.00	0.6	1	0.50	0.4
cspA	1472688	1472888	cold shock protein	K	2	0	0.00	0.6	0	0.00	0.0
SAR2157	2226161	2226331	hypothetical protein		2	0	0.00	0.6	1	0.50	0.4
atpC	2259534	2259938	F0F1 ATP synthase subunit epsilon	C	2	0	0.00	0.6	0	0.00	0.0
atpF	2264358	2264879	F0F1 ATP synthase subunit B	C	2	0	0.00	0.6	1	0.50	0.4
SARs024	2311657	2311873	NA		2	0	0.00	0.6	0	0.00	0.0
rpsE	2399043	2399543	30S ribosomal protein S5	J	2	0	0.00	0.6	0	0.00	0.0
rplF	2399954	2400490	50S ribosomal protein L6	J	2	0	0.00	0.6	0	0.00	0.0
rpsQ	2402472	2402735	30S ribosomal protein S17	J	2	0	0.00	0.6	1	0.50	0.4
rplV	2404072	2404425	50S ribosomal protein L22	J	2	0	0.00	0.6	1	0.50	0.4
SAR2391a	2460526	2460777	hypothetical protein		2	0	0.00	0.6	0	0.00	0.0
SAR0681	728670	729143	hypothetical protein	S	21	16	0.76	0.6	10	0.48	2.2
SAR1211	1262550	1263800	cell division protein	U	25	20	0.80	0.6	5	0.20	0.0
SAR2271	2353535	2354728	hypothetical protein	R	35	29	0.83	0.6	14	0.40	1.9
SAR2245	2313709	2315841	transcriptional antiterminator	K	59	52	0.88	0.6	23	0.39	2.7
SAR2646	2733904	2735397	phytoene dehydrogenase related protein	Q	59	52	0.88	0.6	24	0.41	3.1
SAR0390	426962	427534	hypothetical protein	S	13	9	0.69	0.6	4	0.31	0.3
SAR1417	1473823	1474446	hypothetical protein	R	13	9	0.69	0.6	2	0.15	0.0
SAR1458	1550055	1550618	hypothetical protein	S	13	9	0.69	0.6	4	0.31	0.3
SAR1943	2026144	2026539	hypothetical protein		13	9	0.69	0.6	4	0.31	0.3
SAR2532	2610641	2611492	hypothetical protein	G	34	28	0.82	0.6	10	0.29	0.5
dfrB	1497106	1497585	dihydrofolate reductase	H	20	15	0.75	0.6	6	0.30	0.4
SAR0929	967785	971438	hypothetical protein	L	114	105	0.92	0.6	29	0.25	0.5
thrB	1389988	1390902	homoserine kinase	E	33	27	0.82	0.6	7	0.21	0.0
tenA	2253188	2253877	transcriptional activator	K	33	27	0.82	0.6	10	0.30	0.7
SAR0846	894763	895320	hypothetical protein		5	2	0.40	0.6	3	0.60	1.2
SAR0852	898810	898998	hypothetical protein		5	2	0.40	0.6	1	0.20	0.0
SAR0858	901716	902210	hypothetical protein	J	5	2	0.40	0.6	2	0.40	0.5
SAR0945	988557	988742	hypothetical protein		5	2	0.40	0.6	2	0.40	0.5
SAR1364	1416281	1416577	hypothetical protein	S	5	2	0.40	0.6	3	0.60	1.2
cdd	1714341	1714745	cytidine deaminase	F	5	2	0.40	0.6	2	0.40	0.5
SAR2174	2246538	2246747	hypothetical protein		5	2	0.40	0.6	3	0.60	1.2
rplM	2387530	2387967	50S ribosomal protein L13	J	5	2	0.40	0.6	1	0.20	0.0
SAR2428a	2498007	2498129	hypothetical protein		5	2	0.40	0.6	5	1.00	3.3
SAR0115	125799	126551	regulatory protein	K	19	14	0.74	0.6	9	0.47	2.0
SAR0621	669551	670351	hydrolase	R	19	14	0.74	0.6	6	0.32	0.6
glkA	1692942	1693928	glucokinase	G	19	14	0.74	0.6	2	0.11	0.4
SAR2763	2866243	2867367	hypothetical protein	G	48	41	0.85	0.6	13	0.27	0.4
SAR0966	1009834	1010553	adaptor protein	O	12	8	0.67	0.6	4	0.33	0.5
SAR1013	1055415	1055630	hypothetical protein	S	12	8	0.67	0.6	3	0.25	0.1
ctaB	1138396	1139307	protoheme IX farnesyltransferase	O	12	8	0.67	0.6	4	0.33	0.5
SAR0661	708503	709087	dihydroxyacetone kinase	G	31	25	0.81	0.6	12	0.39	1.6
SAR1995	2084777	2085976	lipoprotein	R	31	25	0.81	0.6	5	0.16	0.2
arcD	2806838	2808274	arginine/ornithine antiporter	E	31	25	0.81	0.6	8	0.26	0.3
pgi	960796	962127	glucose-6-phosphate isomerase	G	30	24	0.80	0.6	6	0.20	0.0
glmU	536269	537621	bifunctional N-acetylglucosamine-1-phosphate uridyltransferase/glucosamine-1-phosphate acetyltransferase	M	44	37	0.84	0.6	17	0.39	2.0
nusG	586599	587147	transcription antitermination protein	K	11	7	0.64	0.6	1	0.09	0.3
SAR0803	840773	841639	hypothetical protein	S	11	7	0.64	0.6	2	0.18	0.0
SAR1143	1188556	1189488	carbamate kinase	E	11	7	0.64	0.6	3	0.27	0.1
hisZ	2865121	2865939	ATP phosphoribosyltransferase regulatory	E	29	23	0.79	0.6	9	0.31	0.6

Gene	Start	End	Product	COG	# Core BiPs	# Homo-plasies	Homoplasy rate	Poisson test: −log10(p)	# Non-synonymous BiPs	Non-synonymous probability	Binomial test: −log10(p)
			subunit								
SAR0645	691135	691887	hypothetical protein		17	12	0.71	0.6	5	0.29	0.4
pyrE	1229077	1229688	orotate phosphoribosyltransferase	F	17	12	0.71	0.6	3	0.18	0.0
SAR1707	1768587	1769861	recombination factor protein RarA	L	43	36	0.84	0.6	6	0.14	0.6
murD	1203079	1204428	UDP-N-acetylmuramoyl-L-alanyl-D-glutamate synthetase	M	42	35	0.83	0.6	6	0.14	0.5
crtN	2730351	2731859	squalene synthase	Q	42	35	0.83	0.6	11	0.26	0.3
SAR2702	2795761	2796648	sensor kinase protein	T	37	30	0.81	0.7	8	0.22	0.0
fhuB	705242	706246	ferrichrome transport permease	P	36	29	0.81	0.7	15	0.42	2.2
lrgB	302471	303172	antiholin-like protein LrgB	M	16	11	0.69	0.7	0	0.00	1.5
SAR0466	494516	494920	MutT domain-containing protein	R	16	11	0.69	0.7	7	0.44	1.2
recA	1322409	1323452	recombinase A	L	16	11	0.69	0.7	1	0.06	0.7
SAR2464	2535094	2535717	TetR family regulatory protein	K	16	11	0.69	0.7	6	0.38	0.9
SAR2499	2571954	2572553	lipoprotein	O	16	11	0.69	0.7	5	0.31	0.4
SAR2204	2270721	2271767	hypothetical protein	J	40	33	0.83	0.7	15	0.38	1.7
SAR0629	676120	676680	phage integrase family protein	L	10	6	0.60	0.7	3	0.30	0.3
SAR1173	1218435	1219352	RNA pseudouridylate synthase	J	10	6	0.60	0.7	5	0.50	1.4
hslV	1283878	1284423	ATP-dependent protease peptidase subunit	O	10	6	0.60	0.7	2	0.20	0.0
czrB	2301412	2302392	zinc resistance protein	P	35	28	0.80	0.7	6	0.17	0.2
pbpF	1696594	1698669	penicillin-binding protein 3	M	55	47	0.85	0.7	12	0.22	0.1
SAR0549	598222	598533	ribosomal protein L7Ae-like	J	4	1	0.25	0.7	2	0.50	0.7
SAR0592	643752	643949	hypothetical protein		4	1	0.25	0.7	4	1.00	2.7
SAR0854	899628	899831	hypothetical protein		4	1	0.25	0.7	2	0.50	0.7
SAR1444	1501043	1501237	hypothetical protein		4	1	0.25	0.7	4	1.00	2.7
SAR1695	1756234	1756662	Holliday junction resolvase-like protein	L	4	1	0.25	0.7	1	0.25	0.0
SAR1705	1767798	1767980	hypothetical protein		4	1	0.25	0.7	1	0.25	0.0
SAR2243	2311923	2312120	hypothetical protein		4	1	0.25	0.7	4	1.00	2.7
rplQ	2393673	2394041	50S ribosomal protein L17	J	4	1	0.25	0.7	0	0.00	0.2
kdpC	2230735	2231295	potassium-transporting ATPase subunit C	P	34	27	0.79	0.7	18	0.53	4.3
SAR0674	721027	722034	phosphate transport protein	P	38	31	0.82	0.7	1	0.03	2.6
SAR0462	491043	491702	transport system membrane protein	P	15	10	0.67	0.7	2	0.13	0.1
SAR0667	713726	714232	hypothetical protein	J	15	10	0.67	0.7	1	0.07	0.7
SAR1654	1721292	1722044	16S ribosomal RNA methyltransferase RsmE	S	15	10	0.67	0.7	4	0.27	0.3
nasD	2562943	2565348	nitrite reductase large subunit	C	123	112	0.91	0.7	18	0.15	1.1
SAR1202	1250143	1251789	hypothetical protein	R	33	26	0.79	0.7	10	0.30	0.7
SAR0460	488581	489723	Cys/Met metabolism PLP-dependent enzyme	E	47	39	0.83	0.7	9	0.19	0.1
serS	12783	14069	seryl-tRNA synthetase	J	24	18	0.75	0.7	9	0.38	1.1
mnaA	2266598	2267731	UDP-GlcNAc 2-epimerase	M	24	18	0.75	0.7	4	0.17	0.1
SAR0875	912393	913244	hypothetical protein	S	32	25	0.78	0.7	8	0.25	0.2
hemE	2005357	2006394	uroporphyrinogen decarboxylase	H	32	25	0.78	0.7	2	0.06	1.5
spa	123828	125378	immunoglobulin G binding protein A precursor	R	9	5	0.56	0.7	2	0.22	0.0
SAR0614	663359	663787	hypothetical protein	S	9	5	0.56	0.7	7	0.78	3.3
SAR1647	1715103	1715570	hypothetical protein	R	9	5	0.56	0.7	5	0.56	1.6
infC	1821987	1822514	translation initiation factor IF-3	J	9	5	0.56	0.7	2	0.22	0.0
SAR1985	2073732	2074286	exonuclease	L	9	5	0.56	0.7	2	0.22	0.0
SAR2030	2121920	2123989	MHC class II analog		9	5	0.56	0.7	4	0.44	1.0
ureF	2444474	2445163	urease accessory protein UreF	O	9	5	0.56	0.7	1	0.11	0.2
SAR2184	2254402	2255097	hypothetical protein		14	9	0.64	0.7	8	0.57	2.4
SAR2203	2270305	2270724	low molecular weight protein-tyrosine-phosphatase	T	14	9	0.64	0.7	7	0.50	1.8
SAR1099	1144646	1145785	hypothetical protein	R	45	37	0.82	0.7	20	0.44	3.1

Gene	Start	End	Product	COG	# Core BiPs	# Homo-plasies	Homoplasy rate	Poisson test: −log10(p)	# Non-synonymous BiPs	Non-synonymous probability	Binomial test: −log10(p)
SAR0766	800265	800858	glutamine amidotransferase class-I protein	H	20	14	0.70	0.7	8	0.40	1.3
argH	957871	959250	argininosuccinate lyase	E	31	24	0.77	0.7	5	0.16	0.2
SAR0590	642212	642664	hypothetical protein	S	23	17	0.74	0.7	1	0.04	1.4
SAR0675	722621	723418	hypothetical protein	R	23	17	0.74	0.7	3	0.13	0.3
atpA	2262289	2263797	F0F1 ATP synthase subunit alpha	C	23	17	0.74	0.7	1	0.04	1.4
SAR2447	2516594	2517268	response regulator protein	T	23	17	0.74	0.7	5	0.22	0.0
scrB	2188156	2189640	sucrose-6-phosphate hydrolase	G	60	51	0.85	0.7	17	0.28	0.7
SAR2270	2351791	2353548	hypothetical protein	Q	59	50	0.85	0.7	18	0.31	1.0
SAR0546	590008	590616	hypothetical protein	J	22	16	0.73	0.8	10	0.45	1.8
spsB	963573	964148	signal peptidase Ib	U	22	16	0.73	0.8	3	0.14	0.2
SAR1960	2052335	2053171	hypothetical protein	G	22	16	0.73	0.8	10	0.45	1.8
purC	1085419	1086123	phosphoribosylaminoimidazole-succinocarboxamide synthase	F	13	8	0.62	0.8	5	0.38	0.8
SAR1953	2043633	2044085	AhpC/TSA family protein	O	13	8	0.62	0.8	4	0.31	0.3
SAR2228	2295730	2296143	hypothetical protein	S	13	8	0.62	0.8	7	0.54	2.0
SAR1825	1907226	1908068	metallo-beta-lactamase superfamily protein	R	29	22	0.76	0.8	10	0.34	1.0
SAR1610	1681878	1682708	lipoate-protein ligase A protein	H	21	15	0.71	0.8	5	0.24	0.1
SAR1635	1702777	1704123	helicase	L	21	15	0.71	0.8	6	0.29	0.4
tgt	1784562	1785701	queuine tRNA-ribosyltransferase	J	21	15	0.71	0.8	1	0.05	1.2
SAR2435	2503753	2504673	hypothetical protein	I	21	15	0.71	0.8	3	0.14	0.2
SAR0742	776954	777241	hypothetical protein		8	4	0.50	0.8	2	0.25	0.2
SAR0899	935005	935328	hypothetical protein	S	8	4	0.50	0.8	4	0.50	1.2
SAR0931	972832	973221	hypothetical protein		8	4	0.50	0.8	2	0.25	0.2
qoxA	1079932	1081032	quinol oxidase polypeptide II precursor	C	8	4	0.50	0.8	0	0.00	0.7
SAR1258	1319864	1320256	DNA-binding protein	S	8	4	0.50	0.8	1	0.13	0.0
SAR2297b	2384073	2384255	hypothetical protein		8	4	0.50	0.8	6	0.75	2.7
pyrF	1228382	1229077	orotidine 5'-phosphate decarboxylase	F	28	21	0.75	0.8	10	0.36	1.0
SAR0799	836424	837185	hypothetical protein	S	18	12	0.67	0.8	0	0.00	1.7
SAR1785	1858278	1858967	metal-dependent hydrolase	R	18	12	0.67	0.8	3	0.17	0.1
opuD1	1410505	1412151	glycine betaine transporter 1	M	40	32	0.80	0.8	4	0.10	1.1
SAR2235	2302658	2303749	hypothetical protein		40	32	0.80	0.8	19	0.48	3.5
SAR2367	2436583	2437524	inosine-uridine preferring nucleoside hydrolase	F	40	32	0.80	0.8	10	0.25	0.2
SAR2449	2518797	2519240	hypothetical protein	T	12	7	0.58	0.8	6	0.50	1.6
SAR2469	2542465	2542887	hypothetical protein	R	12	7	0.58	0.8	5	0.42	0.8
aroA	1568537	1569835	3-phosphoshikimate 1-carboxyvinyltransferase	E	38	30	0.79	0.8	4	0.11	0.9
SAR1803	1877886	1879352	PTS system IIBC component	G	47	38	0.81	0.8	11	0.23	0.1
SAR1770	1835659	1837146	hypothetical protein		34	26	0.76	0.8	17	0.50	3.7
SAR0473	505439	506866	sugar-specific PTS transport system, IIBC component	G	62	52	0.84	0.8	10	0.16	0.4
hslO	552594	553475	Hsp33-like chaperonin	O	17	11	0.65	0.8	8	0.47	1.8
mnhE	944865	945344	monovalent cation/H+ antiporter subunit E	P	17	11	0.65	0.8	3	0.18	0.0
SAR1728	1791873	1792403	hypothetical protein	M	17	11	0.65	0.8	6	0.35	0.6
SAR1931	2011035	2011592	hypothetical protein		17	11	0.65	0.8	3	0.18	0.0
mobB	2427728	2428213	molybdopterin-guanine dinucleotide biosynthesis protein B	H	17	11	0.65	0.8	10	0.59	3.1
SAR2392	2461053	2461526	hypothetical protein	S	17	11	0.65	0.8	3	0.18	0.0
SAR2612	2696190	2697233	hypothetical protein	G	17	11	0.65	0.8	2	0.12	0.3
SAR1163	1208738	1209529	hypothetical protein	S	26	19	0.73	0.8	10	0.38	1.3
SAR1197	1246574	1247449	hypothetical protein	R	26	19	0.73	0.8	5	0.19	0.0
SAR1621	1690967	1691941	hypothetical protein	U	26	19	0.73	0.8	6	0.23	0.1
SAR2514	2587795	2588910	8-amino-7-oxononanoate synthase	H	36	28	0.78	0.9	18	0.50	3.9
SAR1634	1701877	1702767	endonuclease IV	L	25	18	0.72	0.9	5	0.20	0.0

Gene	Start	End	Product	COG	# Core BiPs	# Homo-plasies	Homoplasy rate	Poisson test: −log10(p)	# Non-synonymous BiPs	Non-synonymous probability	Binomial test: −log10(p)
SAR1688	1750595	1751329	hypothetical protein	E	25	18	0.72	0.9	11	0.44	1.9
gltT	2545682	2546959	proton/sodium-glutamate symport protein	C	32	24	0.75	0.9	2	0.06	1.5
SAR0620	668848	669567	haloacid dehalogenase-like hydrolase	R	16	10	0.63	0.9	1	0.06	0.7
cmk	1580680	1581339	cytidylate kinase	F	16	10	0.63	0.9	3	0.19	0.0
oppC	992830	993900	oligopeptide ABC transporter permease protein	P	11	6	0.55	0.9	7	0.64	2.5
SAR1989	2077812	2078759	lipid kinase	R	11	6	0.55	0.9	3	0.27	0.1
SAR2665	2753126	2753422	hypothetical protein	S	11	6	0.55	0.9	6	0.55	1.8
cysE	581734	582381	serine acetyltransferase	E	7	3	0.43	0.9	3	0.43	0.8
nusB	1676133	1676522	transcription antitermination protein NusB	K	7	3	0.43	0.9	1	0.14	0.0
greA	1751655	1752131	transcription elongation factor GreA	K	7	3	0.43	0.9	1	0.14	0.0
SAR2230	2298638	2298868	hypothetical protein		7	3	0.43	0.9	3	0.43	0.8
SAR2274	2357539	2357778	hypothetical protein	S	7	3	0.43	0.9	3	0.43	0.8
SAR2616	2700275	2700631	hypothetical protein		7	3	0.43	0.9	2	0.29	0.2
SAR0506	545441	546634	tetrapyrrole (corrin/porphyrin) methylase	R	35	27	0.77	0.9	15	0.43	2.2
isdF	1152953	1153921	iron/heme permease	P	35	27	0.77	0.9	12	0.34	1.0
SAR0771	803655	804659	hypothetical protein	E	31	23	0.74	0.9	5	0.16	0.2
SAR2259	2340591	2341058	hypothetical protein		31	23	0.74	0.9	2	0.06	1.3
rocD	953762	954952	ornithine--oxo-acid transaminase	E	24	17	0.71	0.9	2	0.08	0.9
fumC	2024563	2025948	fumarate hydratase	C	42	33	0.79	0.9	7	0.17	0.2
SAR0492	530414	531187	TatD related DNase	L	15	9	0.60	0.9	3	0.20	0.0
SAR0634	681341	681823	monovalent cation/H+ antiporter subunit E	P	15	9	0.60	0.9	0	0.00	1.3
saeS	794174	795229	histidine kinase protein	T	15	9	0.60	0.9	7	0.47	1.6
SAR1097	1143558	1144100	methylase	L	15	9	0.60	0.9	4	0.27	0.3
agrA	2186110	2186826	autoinducer sensor protein response regulator protein	T	23	16	0.70	0.9	1	0.04	1.4
SAR0504	540419	543925	transcription-repair coupling factor	L	85	73	0.86	0.9	18	0.21	0.0
lysA	1470589	1471854	diaminopimelate decarboxylase	E	68	57	0.84	0.9	4	0.06	3.0
pyk	1844896	1846653	pyruvate kinase	G	40	31	0.78	0.9	5	0.13	0.6
SAR0641	687790	688719	ABC transporter	P	10	5	0.50	0.9	1	0.10	0.2
SAR0985	1029615	1030124	hypothetical protein	J	10	5	0.50	0.9	4	0.40	0.6
SAR2186	2256075	2256515	hypothetical protein		10	5	0.50	0.9	4	0.40	0.6
glpT	378556	379914	glycerol-3-phosphate transporter	G	39	30	0.77	0.9	3	0.08	1.5
capA	168658	169326	capsular polysaccharide synthesis enzyme	M	28	20	0.71	0.9	6	0.21	0.0
SAR1147	1192065	1193012	hypothetical protein	P	14	8	0.57	1.0	2	0.14	0.1
tagB	694999	696102	teichoic acid biosynthesis protein	M	46	36	0.78	1.0	22	0.48	4.1
SAR0623	671155	671871	hypothetical protein		21	14	0.67	1.0	5	0.24	0.1
pdhA	1112910	1114022	pyruvate dehydrogenase E1 component, alpha subunit	C	21	14	0.67	1.0	3	0.14	0.2
SAR1014	1055795	1056346	hypothetical protein	R	27	19	0.70	1.0	12	0.44	2.1
SAR0872	910328	911149	lipoprotein	P	37	28	0.76	1.0	5	0.14	0.5
SAR0241	282714	282992	PTS transport system, IIB component	G	6	2	0.33	1.0	1	0.17	0.0
rpsF	407998	408294	30S ribosomal protein S6	J	6	2	0.33	1.0	0	0.00	0.5
SAR0578	633590	633883	hypothetical protein		6	2	0.33	1.0	5	0.83	2.7
mnhC	946835	947176	monovalent cation/H+ antiporter subunit C	P	6	2	0.33	1.0	1	0.17	0.0
qoxC	1077349	1077954	quinol oxidase polypeptide III	C	6	2	0.33	1.0	0	0.00	0.5
SAR1086	1130946	1131221	hypothetical protein	S	6	2	0.33	1.0	2	0.33	0.2
rnc	1258106	1258837	ribonuclease III	K	6	2	0.33	1.0	0	0.00	0.5
SAR2264	2345332	2345592	hypothetical protein		6	2	0.33	1.0	1	0.17	0.0
SAR2407	2475266	2475796	hypothetical protein		6	2	0.33	1.0	1	0.17	0.0
SAR0200	230726	231889	ABC transporter permease	P	36	27	0.75	1.0	12	0.33	1.0
SAR0194	222069	222947	transcriptional regulator	K	13	7	0.54	1.0	1	0.08	0.5
SAR1015	1056398	1057336	1,4-dihydroxy-2-naphthoate octaprenyltransferase	H	20	13	0.65	1.0	3	0.15	0.2

Gene	Start	End	Product	COG	# Core BiPs	# Homo-plasies	Homoplasy rate	Poisson test: −log10(p)	# Non-synonymous BiPs	Non-synonymous probability	Binomial test: −log10(p)
SAR1126	1174866	1175033	hypothetical protein		9	4	0.44	1.0	6	0.67	2.4
SAR1666	1735233	1735694	deaminase	F	9	4	0.44	1.0	2	0.22	0.0
luxS	2290712	2291182	S-ribosylhomocysteinase	T	9	4	0.44	1.0	1	0.11	0.2
rir1	821858	823963	ribonucleotide-diphosphate reductase subunit alpha	F	69	57	0.83	1.0	3	0.04	3.8
SAR2438	2507715	2508362	hypothetical protein	V	35	26	0.74	1.0	4	0.11	0.7
capE	172628	173656	capsular polysaccharide synthesis enzyme	G	42	32	0.76	1.0	5	0.12	0.7
SAR1378	1435133	1436224	prephenate dehydrogenase	E	42	32	0.76	1.0	19	0.45	3.3
SAR0656	703294	704127	hypothetical protein	S	25	17	0.68	1.0	4	0.16	0.2
murB	829895	830818	UDP-N-acetylenolpyruvoylglucosamine reductase	M	41	31	0.76	1.0	7	0.17	0.2
polC	1295705	1300021	DNA polymerase III PolC	L	135	119	0.88	1.0	21	0.16	0.9
SAR2150	2219748	2220203	hypothetical protein	S	19	12	0.63	1.0	4	0.21	0.0
SAR1117	1164091	1166439	recombination and DNA strand exchange inhibitor protein	L	66	54	0.82	1.0	15	0.23	0.1
tagA	692123	692887	teichoic acid biosynthesis protein	M	24	16	0.67	1.1	8	0.33	0.7
SAR0733	767906	768472	hypothetical protein	R	24	16	0.67	1.1	3	0.13	0.3
SAR0857	900943	901560	LysE type translocator protein	R	24	16	0.67	1.1	12	0.50	2.7
SAR1051	1097538	1098113	hypothetical protein	S	24	16	0.67	1.1	0	0.00	2.3
rpsA	1578793	1579968	30S ribosomal protein S1	J	24	16	0.67	1.1	8	0.33	0.7
SAR1684	1747017	1747769	LamB/YcsF family protein	R	24	16	0.67	1.1	11	0.46	2.0
SAR1801	1874896	1876500	D-3-phosphoglycerate dehydrogenase	H	33	24	0.73	1.1	7	0.21	0.0
SAR1967	2058263	2059414	hypothetical protein	R	33	24	0.73	1.1	14	0.42	2.1
SAR2395	2466162	2466959	inositol monophosphatase family protein	G	33	24	0.73	1.1	15	0.45	2.7
SAR0776	812212	813726	ABC transporter permease	M	40	30	0.75	1.1	8	0.20	0.0
ung	638927	639583	uracil-DNA glycosylase	L	12	6	0.50	1.1	3	0.25	0.1
SAR1671	1738159	1738728	hypothetical protein	H	12	6	0.50	1.1	5	0.42	0.8
SAR2156	2225802	2226164	hypothetical protein	T	12	6	0.50	1.1	2	0.17	0.0
SAR0361	407563	407754	hypothetical protein		3	0	0.00	1.1	1	0.33	0.3
SAR0879	917084	917548	NifU-like protein	C	3	0	0.00	1.1	0	0.00	0.0
SAR0898	934662	934904	hypothetical protein	O	3	0	0.00	1.1	1	0.33	0.3
SAR0917	951588	951965	S1 RNA-binding domain-containing protein	J	3	0	0.00	1.1	1	0.33	0.3
SAR1154	1197808	1198239	cell division protein MraZ	S	3	0	0.00	1.1	0	0.00	0.0
SARs010	1212605	1212799	NA		3	0	0.00	1.1	0	0.00	0.0
rpsP	1265947	1266222	30S ribosomal protein S16	J	3	0	0.00	1.1	0	0.00	0.0
SAR1625	1693925	1694128	hypothetical protein	S	3	0	0.00	1.1	1	0.33	0.3
SAR1729a	1793445	1793564	hypothetical protein		3	0	0.00	1.1	3	1.00	2.0
rpsH	2400515	2400913	30S ribosomal protein S8	J	3	0	0.00	1.1	0	0.00	0.0
rpsS	2404454	2404732	30S ribosomal protein S19	J	3	0	0.00	1.1	1	0.33	0.3
SAR2402	2472888	2473073	hypothetical protein		3	0	0.00	1.1	2	0.67	0.9
SAR2645	2732771	2733898	glycosyl transferase	M	59	47	0.80	1.1	30	0.51	6.2
ilvD	2202229	2203917	dihydroxy-acid dehydratase	E	39	29	0.74	1.1	7	0.18	0.2
SAR0783	819829	820695	hypothetical protein		50	39	0.78	1.1	5	0.10	1.2
murC	1898446	1899759	UDP-N-acetylmuramate--L-alanine ligase	M	29	20	0.69	1.1	4	0.14	0.3
SAR1950	2032259	2033320	hypothetical protein	R	29	20	0.69	1.1	9	0.31	0.6
tagH	692947	693741	teichoic acids export protein ATP-binding subunit	G	18	11	0.61	1.1	4	0.22	0.0
SAR1866	1954517	1954960	camphor resistance protein CrcB	D	18	11	0.61	1.1	6	0.33	0.6
fni	2500614	2501663	isopentenyl pyrophosphate isomerase	C	18	11	0.61	1.1	7	0.39	1.1
SAR0467	494910	495395	acetyltransferase	R	23	15	0.65	1.1	11	0.48	2.4
SAR2232	2300273	2300938	hypothetical protein	G	23	15	0.65	1.1	6	0.26	0.2
SAR1672	1738731	1739021	hypothetical protein	J	8	3	0.38	1.1	1	0.13	0.0
SAR2462	2533019	2533450	hypothetical protein		8	3	0.38	1.1	3	0.38	0.4
capD	170815	172638	capsular polysaccharide synthesis enzyme	G	70	57	0.81	1.1	16	0.23	0.1

Gene	Start	End	Product	COG	# Core BiPs	# Homo-plasies	Homoplasy rate	Poisson test: −log10(p)	# Non-synonymous BiPs	Non-synonymous probability	Binomial test: −log10(p)
SAR0747	781776	782198	hypothetical protein	R	31	22	0.71	1.1	12	0.39	1.6
ftsH	550087	552180	cell division protein	O	28	19	0.68	1.1	2	0.07	1.2
SAR0676	723779	724423	hypothetical protein	R	28	19	0.68	1.1	6	0.21	0.0
SAR1160	1204441	1205763	cell division protein	M	48	37	0.77	1.1	22	0.46	3.8
SAR1399	1455868	1456719	phosphate transporter ATP-binding protein	P	56	44	0.79	1.1	8	0.14	0.6
sdhB	1171787	1172602	succinate dehydrogenase iron-sulfur subunit	C	37	27	0.73	1.1	3	0.08	1.3
SAR1780	1849908	1851137	NAD-dependent malic enzyme	C	37	27	0.73	1.1	4	0.11	0.8
SARs003	403755	404014	NA		5	1	0.20	1.1	0	0.00	0.2
SAR1084	1130154	1130321	hypothetical protein	S	5	1	0.20	1.1	0	0.00	0.2
SAR1095	1142842	1143096	hypothetical protein	S	5	1	0.20	1.1	0	0.00	0.2
SAR1092	1140120	1141157	hypothetical protein	S	22	14	0.64	1.1	9	0.41	1.4
SAR1262	1323806	1325365	phosphodiesterase	R	22	14	0.64	1.1	3	0.14	0.2
SAR1978	2067232	2067990	methionine aminopeptidase	J	17	10	0.59	1.1	3	0.18	0.0
murA2	2278721	2279980	UDP-N-acetylglucosamine 1-carboxyvinyltransferase	M	30	21	0.70	1.1	5	0.17	0.2
arcB	2808359	2809369	ornithine carbamoyltransferase	E	30	21	0.70	1.1	10	0.33	0.9
SAR0330	376045	376464	MarR family regulatory protein	K	11	5	0.45	1.1	3	0.27	0.1
SAR0735	768935	769618	hypothetical protein		11	5	0.45	1.1	3	0.27	0.1
SAR1693	1754988	1755626	O-methyltransferase	R	11	5	0.45	1.1	4	0.36	0.6
SAR2624	2708550	2708981	hypothetical protein	R	11	5	0.45	1.1	3	0.27	0.1
rplI	20274	20720	50S ribosomal protein L9	J	27	18	0.67	1.2	3	0.11	0.6
SAR0755	790585	791934	hypothetical protein	R	36	26	0.72	1.2	4	0.11	0.8
SAR1079	1124587	1125939	manganese transport protein MntH	P	46	35	0.76	1.2	6	0.13	0.7
arlS	1486310	1487665	sensor kinase protein	T	46	35	0.76	1.2	6	0.13	0.7
pepA	939170	940645	cytosol aminopeptidase family protein	E	53	41	0.77	1.2	17	0.32	1.2
ldh1	275703	276656	L-lactate dehydrogenase	C	21	13	0.62	1.2	8	0.38	1.0
SAR0514	553654	554586	O-acetylserine (thiol)-lyase	E	21	13	0.62	1.2	2	0.10	0.5
SAR1670	1737585	1738169	hypothetical protein	H	21	13	0.62	1.2	6	0.29	0.4
trmB	1915146	1915790	tRNA (guanine-N(7)-)-methyltransferase	R	21	13	0.62	1.2	9	0.43	1.5
SAR1838	1920053	1920748	RNA pseudouridine synthase	J	35	25	0.71	1.2	16	0.46	2.9
SAR2411	2479295	2480671	transport protein	C	35	25	0.71	1.2	6	0.17	0.2
SAR2523	2598638	2599297	hypothetical protein		16	9	0.56	1.2	4	0.25	0.1
citZ	1841719	1842840	citrate synthase	C	34	24	0.71	1.2	0	0.00	3.3
SAR1802	1876650	1877780	hypothetical protein	R	34	24	0.71	1.2	18	0.53	4.3
SAR0240	282224	282691	PTS transport system, IIA component	G	25	16	0.64	1.2	6	0.24	0.1
SAR0589	640586	642070	amino acid permease	E	25	16	0.64	1.2	3	0.12	0.5
SAR1708	1770022	1770795	hypothetical protein	H	25	16	0.64	1.2	2	0.08	0.9
SAR0826	870369	870998	hypothetical protein		20	12	0.60	1.2	11	0.55	3.0
pheS	1156564	1157622	phenylalanyl-tRNA synthetase subunit alpha	J	20	12	0.60	1.2	2	0.10	0.5
SAR0868	908051	908347	hypothetical protein		7	2	0.29	1.2	3	0.43	0.8
rpsB	1287032	1287799	30S ribosomal protein S2	J	7	2	0.29	1.2	1	0.14	0.0
SAR1817	1897881	1898372	hypothetical protein	R	7	2	0.29	1.2	2	0.29	0.2
SAR1865	1954038	1954340	hypothetical protein		7	2	0.29	1.2	1	0.14	0.0
SAR1977	2066629	2067015	hypothetical protein		7	2	0.29	1.2	4	0.57	1.4
trpE	1437913	1439319	anthranilate synthase component I	H	56	43	0.77	1.3	19	0.34	1.5
gcvH	905645	906025	glycine cleavage system protein H	E	15	8	0.53	1.3	6	0.40	1.0
SAR1280	1345292	1345768	glutathione peroxidase	O	15	8	0.53	1.3	4	0.27	0.3
SAR1618	1689176	1689610	hypothetical protein	U	15	8	0.53	1.3	5	0.33	0.5
SAR1965	2057233	2057748	ThiJ/PfpI family protein	R	15	8	0.53	1.3	5	0.33	0.5
SAR1435	1494700	1495200	PTS system, glucose-specific IIA component	G	32	22	0.69	1.3	3	0.09	0.9
gatB	2079582	2081009	aspartyl/glutamyl-tRNA amidotransferase	J	32	22	0.69	1.3	1	0.03	2.1

Gene	Start	End	Product	COG	# Core BiPs	# Homo-plasies	Homoplasy rate	Poisson test: −log10(p)	# Non-synonymous BiPs	Non-synonymous probability	Binomial test: −log10(p)
			subunit B								
SAR0175	192733	193494	ABC transporter permease	P	24	15	0.63	1.3	6	0.25	0.2
SAR0892	928768	929727	D-isomer specific 2-hydroxyacid dehydrogenase	R	38	27	0.71	1.3	15	0.39	2.0
SAR2468	2540289	2542394	AraC family transcription regulator	K	55	42	0.76	1.3	4	0.07	2.1
dapD	1467496	1468215	tetrahydrodipicolinate acetyltransferase	E	19	11	0.58	1.3	1	0.05	1.0
SAR2268	2349338	2350321	transport system binding lipoprotein	P	37	26	0.70	1.3	10	0.27	0.4
SAR1973	2062924	2064141	hypothetical protein	S	31	21	0.68	1.3	4	0.13	0.4
SAR2496	2569326	2570873	solute binding lipoprotein	R	31	21	0.68	1.3	4	0.13	0.4
gapR	871492	872505	glycolytic operon regulator	K	14	7	0.50	1.3	1	0.07	0.5
ppnK	1017069	1017878	inorganic polyphosphate/ATP-NAD kinase	G	14	7	0.50	1.3	0	0.00	1.3
fhuA	704409	705206	ferrichrome transport ATP-binding protein	P	18	10	0.56	1.3	2	0.11	0.4
SAR1091	1139332	1139793	hypothetical protein	S	18	10	0.56	1.3	4	0.22	0.0
pyrAA	1224009	1225109	carbamoyl phosphate synthase small subunit	F	18	10	0.56	1.3	4	0.22	0.0
SAR1199	1248101	1248742	hypothetical protein	H	18	10	0.56	1.3	7	0.39	1.1
SAR1347	1396883	1397860	guanosine 5'-monophosphate oxidoreductase	F	18	10	0.56	1.3	6	0.33	0.6
engB	1816036	1816626	ribosome biogenesis GTP-binding protein YsxC	R	18	10	0.56	1.3	3	0.17	0.1
capP	184233	185408	capsular polysaccharide synthesis enzyme	M	35	24	0.69	1.3	10	0.29	0.5
SAR1716	1779253	1781526	single-stranded-DNA-specific exonuclease	L	70	55	0.79	1.3	25	0.36	2.3
SAR1607	1679835	1680896	peptidase	E	29	19	0.66	1.4	3	0.10	0.8
SAR0321	367281	367613	glycine cleavage H-protein	E	10	4	0.40	1.4	1	0.10	0.2
pyrR	1219754	1220281	bifunctional pyrimidine regulatory protein PyrR uracil phosphoribosyltransferase	F	10	4	0.40	1.4	1	0.10	0.2
SAR2177	2247217	2248701	cardiolipin synthetase	I	43	31	0.72	1.4	5	0.12	0.9
pyrC	1222733	1224007	dihydroorotase	F	34	23	0.68	1.4	6	0.18	0.2
lrgA	302035	302478	murein hydrolase regulator LrgA	R	4	0	0.00	1.4	1	0.25	0.0
ptsH	1102625	1102891	phosphocarrier protein HPr	G	4	0	0.00	1.4	1	0.25	0.0
SAR1189	1238170	1239123	lipoprotein		4	0	0.00	1.4	0	0.00	0.2
SAR1434	1494479	1494700	hypothetical protein	S	4	0	0.00	1.4	0	0.00	0.2
SAR1858	1949009	1949233	hypothetical protein		4	0	0.00	1.4	0	0.00	0.2
SAR1999	2091060	2091362	hypothetical protein	S	4	0	0.00	1.4	0	0.00	0.2
rplE	2401153	2401692	50S ribosomal protein L5	J	4	0	0.00	1.4	1	0.25	0.0
SAR0587	639584	639964	hypothetical protein		17	9	0.53	1.4	9	0.53	2.4
SAR0971	1015274	1015867	hypothetical protein	S	17	9	0.53	1.4	9	0.53	2.4
SAR1198	1247450	1248094	ribulose-phosphate 3-epimerase	G	17	9	0.53	1.4	2	0.12	0.3
rpoD	1706148	1707254	RNA polymerase sigma factor RpoD	K	17	9	0.53	1.4	0	0.00	1.5
SAR2231	2298899	2299837	mannose-6-phosphate isomerase	G	17	9	0.53	1.4	6	0.35	0.6
SAR2025	2116195	2117091	ABC transporter ATP-binding protein	V	28	18	0.64	1.4	12	0.43	2.0
hutI	2484274	2485512	imidazolonepropionase	Q	42	30	0.71	1.4	5	0.12	0.7
recF	3912	5024	recombination protein F	L	21	12	0.57	1.4	6	0.29	0.4
SAR0991	1036601	1037404	hypothetical protein	P	21	12	0.57	1.4	6	0.29	0.4
prmA	1722046	1722984	ribosomal protein L11 methyltransferase	J	21	12	0.57	1.4	8	0.38	1.0
SAR0525	571840	572301	DNA-binding protein	K	13	6	0.46	1.4	1	0.08	0.5
SAR0765	799246	799914	hypothetical protein	R	13	6	0.46	1.4	3	0.23	0.1
SAR0871	909615	910310	ABC transporter permease	P	33	22	0.67	1.4	0	0.00	3.3
pheT	1157622	1160024	phenylalanyl-tRNA synthetase subunit beta	J	104	86	0.83	1.4	25	0.24	0.3
SAR0752	785996	786916	phosphofructokinase	G	41	29	0.71	1.4	13	0.32	0.9
nupC	570468	571682	nucleoside permease	F	27	17	0.63	1.4	3	0.11	0.6
purQ	1086388	1087059	phosphoribosylformylglycinamidine synthase I	F	27	17	0.63	1.4	8	0.30	0.5
SAR0775	811242	812219	ABC transporter ATP-binding protein	E	32	21	0.66	1.4	16	0.50	3.5

Gene	Start	End	Product	COG	# Core BiPs	# Homo-plasies	Homoplasy rate	Poisson test: −log10(p)	# Non-synonymous BiPs	Non-synonymous probability	Binomial test: −log10(p)
SAR0877	914431	915738	hypothetical protein	O	32	21	0.66	1.4	5	0.16	0.3
SAR0230	271601	273076	extracellular solute-binding lipoprotein	E	40	28	0.70	1.4	12	0.30	0.7
SAR0632	679510	679854	monovalent cation/H+ antiporter subunit C	P	20	11	0.55	1.5	1	0.05	1.0
ftsA	1205869	1207275	cell division protein	D	20	11	0.55	1.5	3	0.15	0.2
SAR1675	1740946	1741473	hypothetical protein	R	16	8	0.50	1.5	1	0.06	0.7
pfs	1741493	1742179	5'-methylthioadenosine/S-adenosylhomocysteine nucleosidase	F	16	8	0.50	1.5	8	0.50	2.0
SAR2344	2416096	2416413	hypothetical protein		16	8	0.50	1.5	5	0.31	0.4
sstA	825431	826402	FecCD transport family protein	P	46	33	0.72	1.5	2	0.04	2.7
SAR2409	2477797	2478561	transcription regulator	K	26	16	0.62	1.5	3	0.12	0.5
mraY	1202112	1203077	phospho-N-acetylmuramoyl-pentapeptide-transferase	M	31	20	0.65	1.5	5	0.16	0.2
SAR1800	1873749	1874909	soluble hydrogenase subunit	E	31	20	0.65	1.5	8	0.26	0.3
SAR2390	2457939	2459063	hypothetical protein	H	31	20	0.65	1.5	10	0.32	0.7
SAR2426	2495011	2496243	hypothetical protein	P	31	20	0.65	1.5	8	0.26	0.3
SAR1016	1057509	1058879	chorismate binding enzyme	Q	36	24	0.67	1.5	13	0.36	1.4
hemC	1812668	1813594	porphobilinogen deaminase	H	36	24	0.67	1.5	7	0.19	0.0
argR	1672814	1673266	arginine repressor	K	9	3	0.33	1.5	0	0.00	0.7
zur	1699664	1700074	zinc-specific metalloregulatory protein	P	9	3	0.33	1.5	1	0.11	0.2
SAR2137	2200177	2200641	acetyltransferase	R	12	5	0.42	1.5	3	0.25	0.1
SAR1113	1160194	1161132	ribonuclease HIII	L	50	36	0.72	1.5	13	0.26	0.4
SAR1054	1100703	1101875	hypothetical protein	R	35	23	0.66	1.5	12	0.34	1.0
uvrC	1167250	1169031	excinuclease ABC subunit C	L	78	61	0.78	1.5	14	0.18	0.2
acpS	2227630	2227989	4'-phosphopantetheinyl transferase	I	15	7	0.47	1.5	5	0.33	0.5
SAR2337	2407939	2408334	hypothetical protein		15	7	0.47	1.5	2	0.13	0.1
isdD	1150992	1152068	hypothetical protein		43	30	0.70	1.5	29	0.67	9.9
mqo1	2522500	2523978	malatequinone oxidoreductase	R	43	30	0.70	1.5	9	0.21	0.0
gap2	1830014	1831039	glyceraldehyde 3-phosphate dehydrogenase 2	G	29	18	0.62	1.6	5	0.17	0.1
gyrA	7005	9665	DNA gyrase subunit A	L	115	95	0.83	1.6	12	0.10	2.5
SAR1796	1870839	1871303	hypothetical protein	T	24	14	0.58	1.6	9	0.38	1.1
SAR2162	2230047	2230526	hypothetical protein	S	24	14	0.58	1.6	12	0.50	2.7
SAR1190	1239286	1239564	hypothetical protein	S	18	9	0.50	1.6	11	0.61	3.5
SAR0635	681820	682122	monovalent cation/H+ antiporter subunit F	P	6	1	0.17	1.6	1	0.17	0.0
SAR1201	1249754	1250128	hypothetical protein	S	6	1	0.17	1.6	1	0.17	0.0
SAR1704	1767612	1767758	hypothetical protein		6	1	0.17	1.6	3	0.50	0.9
SAR2015	2108998	2109399	hypothetical protein		6	1	0.17	1.6	2	0.33	0.2
SAR2189	2258422	2258655	hypothetical protein	S	6	1	0.17	1.6	2	0.33	0.2
rpsC	2403395	2404048	30S ribosomal protein S3	J	6	1	0.17	1.6	0	0.00	0.5
SAR1094	1141674	1142603	hypothetical protein	C	41	28	0.68	1.6	18	0.44	2.8
SAR1474	1567286	1568530	hypothetical protein	G	41	28	0.68	1.6	14	0.34	1.3
gltX	579856	581310	glutamyl-tRNA synthetase	J	28	17	0.61	1.6	6	0.21	0.0
sspC	1062379	1062708	hypothetical protein		11	4	0.36	1.6	1	0.09	0.3
SAR1078	1124086	1124505	hypothetical protein		11	4	0.36	1.6	7	0.64	2.5
SAR1609	1681596	1681814	hypothetical protein		11	4	0.36	1.6	0	0.00	0.9
SAR0915	950133	950516	hypothetical protein		14	6	0.43	1.6	8	0.57	2.4
SAR1115	1161775	1162296	hypothetical protein	R	14	6	0.43	1.6	6	0.43	1.0
SAR2134	2194939	2196867	ABC transporter ATP-binding protein	R	55	40	0.73	1.6	13	0.24	0.1
SAR0751	785238	785999	DeoR ramily regulatory protein	G	23	13	0.57	1.6	5	0.22	0.0
SAR2018	2111177	2112019	hypothetical protein		23	13	0.57	1.6	9	0.39	1.2
SAR1260	1321093	1322244	hypothetical protein	R	32	20	0.63	1.6	14	0.44	2.4
SAR1029	1073859	1075085	hypothetical protein	K	54	39	0.72	1.6	5	0.09	1.5
lig	2085989	2087992	DNA ligase	L	54	39	0.72	1.6	11	0.20	0.0
SAR1182	1229718	1229930	hypothetical protein		8	2	0.25	1.7	4	0.50	1.2

Gene	Start	End	Product	COG	# Core BiPs	# Homo-plasies	Homoplasy rate	Poisson test: −log10(p)	# Non-synonymous BiPs	Non-synonymous probability	Binomial test: −log10(p)
SAR1608	1681001	1681582	hypothetical protein		8	2	0.25	1.7	4	0.50	1.2
SAR1788	1861766	1862266	universal stress protein	T	8	2	0.25	1.7	0	0.00	0.7
SAR1974	2064254	2064883	response regulator	T	8	2	0.25	1.7	2	0.25	0.2
SAR2019	2112016	2112573	hypothetical protein	S	8	2	0.25	1.7	1	0.13	0.0
rplB	2404799	2405632	50S ribosomal protein L2	J	8	2	0.25	1.7	0	0.00	0.7
accA	1847912	1848856	acetyl-CoA carboxylase carboxyltransferase subunit alpha	I	39	26	0.67	1.7	2	0.05	2.0
purD	1093809	1095056	phosphoribosylamine--glycine ligase	F	44	30	0.68	1.7	17	0.39	2.0
SAR2265	2345724	2347094	hypothetical protein	S	44	30	0.68	1.7	5	0.11	0.9
qoxB	1077944	1079932	quinol oxidase polypeptide I	C	22	12	0.55	1.7	2	0.09	0.7
SAR1400	1456766	1457683	ABC transporter permease	P	38	25	0.66	1.7	7	0.18	0.1
SAR2256	2337747	2338811	hypothetical protein	D	38	25	0.66	1.7	6	0.16	0.3
topA	1279035	1281104	DNA topoisomerase I	L	43	29	0.67	1.7	5	0.12	0.9
SAR0810	850537	851187	phosphohydrolase	R	13	5	0.38	1.7	3	0.23	0.1
SAR2621	2706290	2706685	hypothetical protein	R	13	5	0.38	1.7	2	0.15	0.0
SAR1368	1422412	1423872	sodiumalanine symporter family protein	E	30	18	0.60	1.7	3	0.10	0.7
SAR1087	1131535	1132761	cell division protein	D	37	24	0.65	1.7	3	0.08	1.3
SAR1429	1489107	1489721	hypothetical protein	I	10	3	0.30	1.7	1	0.10	0.2
SAR2219	2286955	2287626	hypothetical protein		10	3	0.30	1.7	5	0.50	1.4
hemD	1811978	1812646	uroporphyrinogen III synthase	H	21	11	0.52	1.8	12	0.57	3.4
SAR2424	2493418	2494437	aldose 1-epimerase	G	21	11	0.52	1.8	11	0.52	2.8
SAR2556	2638637	2639455	hypothetical protein	R	21	11	0.52	1.8	7	0.33	0.7
SAR2461	2531758	2532792	pyridine nucleotide-disulphide oxidoreductase family protein	O	29	17	0.59	1.8	5	0.17	0.1
dnaK	1724263	1726095	molecular chaperone DnaK	O	41	27	0.66	1.8	4	0.10	1.1
SAR2495	2568673	2569140	hypothetical protein	S	17	8	0.47	1.8	5	0.29	0.4
SAR2456	2526274	2527947	hypothetical protein	M	54	38	0.70	1.8	20	0.37	2.1
SAR0174	191762	192736	lipoprotein	P	35	22	0.63	1.8	15	0.43	2.2
SAR1050	1096123	1097523	ABC transporter ATP-binding protein	R	35	22	0.63	1.8	11	0.31	0.8
SAR1857	1947879	1948733	hypothetical protein	G	24	13	0.54	1.8	3	0.13	0.3
SAR0395	429556	429939	hypothetical protein		28	16	0.57	1.8	14	0.50	3.1
SAR0777	813966	815024	histidinol-phosphate aminotransferase	E	28	16	0.57	1.8	8	0.29	0.4
yacO	584170	584916	SpoU rRNA methylase family protein	J	20	10	0.50	1.8	3	0.15	0.2
SAR0987	1031405	1032580	diacylglycerol glucosyltransferase	M	20	10	0.50	1.8	3	0.15	0.2
SAR1151	1194622	1195308	haloacid dehalogenase-like hydrolase	R	20	10	0.50	1.8	6	0.30	0.4
SAR2253	2328689	2329621	hypothetical protein	S	20	10	0.50	1.8	6	0.30	0.4
kdpA	2233361	2235037	potassium-transporting ATPase subunit A	P	69	51	0.74	1.8	28	0.41	3.5
folP	554802	555605	dihydropteroate synthase	H	47	32	0.68	1.8	11	0.23	0.1
SAR0937	978510	980324	hypothetical protein	I	44	29	0.66	1.9	11	0.25	0.2
SAR0992	1037638	1039947	protease	O	108	86	0.80	1.9	57	0.53	12.1
SAR0534	583758	584162	hypothetical protein	S	7	1	0.14	1.9	4	0.57	1.4
SAR1165	1210218	1210781	hypothetical protein	S	7	1	0.14	1.9	2	0.29	0.2
SAR1623	1692613	1692942	hypothetical protein	S	7	1	0.14	1.9	0	0.00	0.4
rpsD	1871547	1872149	30S ribosomal protein S4	J	7	1	0.14	1.9	3	0.43	0.8
SAR1335	1384252	1385106	hypothetical protein		16	7	0.44	1.9	6	0.38	0.9
SAR2291	2374898	2375314	MerR family regulatory protein	K	16	7	0.44	1.9	11	0.69	4.2
murI	1172841	1173641	glutamate racemase	M	27	15	0.56	1.9	3	0.11	0.6
eno	877430	878734	phosphopyruvate hydratase	G	23	12	0.52	1.9	2	0.09	0.7
SAR0817	859420	860859	hypothetical protein	R	38	24	0.63	1.9	6	0.16	0.3
SAR2136	2199159	2200184	DNA-binding/iron metalloprotein/AP endonuclease	O	38	24	0.63	1.9	9	0.24	0.2
SAR2555	2637343	2638644	hypothetical protein	S	51	35	0.69	1.9	15	0.29	0.8
SAR0683	730258	731124	LysR family regulatory protein	K	33	20	0.61	1.9	3	0.09	1.0
SAR0806	843813	844385	S30EA family ribosomal protein	J	5	0	0.00	1.9	1	0.20	0.0

Gene	Start	End	Product	COG	# Core BiPs	# Homo-plasies	Homoplasy rate	Poisson test: −log10(p)	# Non-synonymous BiPs	Non-synonymous probability	Binomial test: −log10(p)
rpsN	1396457	1396726	30S ribosomal protein S14	J	5	0	0.00	1.9	1	0.20	0.0
SAR1457	1549697	1550041	hypothetical protein	D	5	0	0.00	1.9	0	0.00	0.2
SAR1718	1784283	1784543	hypothetical protein	U	5	0	0.00	1.9	0	0.00	0.2
SAR2185	2255486	2255881	single strand DNA-binding protein	L	5	0	0.00	1.9	3	0.60	1.2
hlgA	2583157	2584086	gamma-hemolysin component A precursor		5	0	0.00	1.9	1	0.20	0.0
SAR1063	1108800	1110497	hypothetical protein	R	45	30	0.67	1.9	0	0.00	4.5
SAR1369	1424372	1425223	transcription antiterminator	K	19	9	0.47	1.9	3	0.16	0.1
SAR1959	2051097	2052074	hypothetical protein	R	19	9	0.47	1.9	7	0.37	0.8
SAR2399	2469621	2470493	transcription regulator	K	19	9	0.47	1.9	0	0.00	1.7
SAR0764	798825	799244	6-pyruvoyl tetrahydropterin synthase	H	9	2	0.22	1.9	3	0.33	0.4
SAR1730	1793638	1794111	hypothetical protein		9	2	0.22	1.9	4	0.44	1.0
hlgB	2585585	2586562	gamma-hemolysin component C precursor		9	2	0.22	1.9	1	0.11	0.2
proP	631938	633338	proline/betaine transporter	G	42	27	0.64	1.9	6	0.14	0.5
SAR0581	635261	636400	ketoacyl-CoA thiolase	I	42	27	0.64	1.9	15	0.36	1.4
SAR0770	802961	803671	hypothetical protein	E	15	6	0.40	2.0	4	0.27	0.3
SAR2161	2228477	2230054	hypothetical protein	S	85	64	0.75	2.0	34	0.40	4.0
ahpC	434182	434751	alkyl hydroperoxide reductase subunit C	O	12	4	0.33	2.0	1	0.08	0.3
spsA	963033	963557	signal peptidase Ia	U	12	4	0.33	2.0	6	0.50	1.6
SAR1365	1416955	1417563	hypothetical protein	S	12	4	0.33	2.0	2	0.17	0.0
SAR2410	2478619	2479125	hypothetical protein	S	12	4	0.33	2.0	3	0.25	0.1
SAR1686	1749144	1749593	biotin carboxyl carrier protein of acetyl-CoA carboxylase	I	18	8	0.44	2.0	9	0.50	2.2
mnhA	947597	950002	monovalent cation/H+ antiporter subunit A	P	53	36	0.68	2.0	16	0.30	0.9
pckA	1959203	1960795	phosphoenolpyruvate carboxykinase	C	40	25	0.63	2.0	3	0.08	1.5
sspB	1062746	1063927	cysteine protease precursor		35	21	0.60	2.0	7	0.20	0.0
pdhD	1116390	1117796	dihydrolipoamide dehydrogenase	C	21	10	0.48	2.0	0	0.00	1.9
icaB	2853578	2854450	intercellular adhesion protein B	G	21	10	0.48	2.0	7	0.33	0.7
SAR1660	1727900	1729024	coproporphyrinogen III oxidase	H	52	35	0.67	2.0	18	0.35	1.6
SAR1940	2022011	2023123	histidine kinase	T	39	24	0.62	2.0	10	0.26	0.3
SAR2132	2192666	2193745	hypothetical protein	R	39	24	0.62	2.0	14	0.36	1.3
SAR1768	1831848	1832720	formamidopyrimidine-DNA glycosylase	L	30	17	0.57	2.0	7	0.23	0.1
SAR1949	2030543	2032000	extracellular glutamine-binding protein	E	30	17	0.57	2.0	9	0.30	0.6
grlB	1417763	1419760	DNA topoisomerase IV subunit B	L	45	29	0.64	2.1	10	0.22	0.1
deoD	2294256	2294966	purine nucleoside phosphorylase	F	17	7	0.41	2.1	5	0.29	0.4
recU	1551500	1552126	Holliday junction-specific endonuclease	R	14	5	0.36	2.1	4	0.29	0.3
SAR1732	1794584	1794868	hypothetical protein		14	5	0.36	2.1	5	0.36	0.7
SAR1820	1903628	1904266	hypothetical protein	R	14	5	0.36	2.1	3	0.21	0.0
SAR0860	903179	903895	3-dehydroquinate dehydratase	E	29	16	0.55	2.1	13	0.45	2.3
SAR1110	1155443	1156183	SpoU rRNA methylase family protein	J	29	16	0.55	2.1	7	0.24	0.2
SAR1845	1935419	1936372	hypothetical protein	R	33	19	0.58	2.1	2	0.06	1.5
SAR2542	2622988	2624175	transport protein	G	44	28	0.64	2.1	7	0.16	0.3
SAR2448	2517261	2518634	sensor kinase protein	T	49	32	0.65	2.1	14	0.29	0.7
SAR1422	1480122	1480325	hypothetical protein		11	3	0.27	2.2	2	0.18	0.0
SAR1459a	1551024	1551194	hypothetical protein		11	3	0.27	2.2	9	0.82	4.5
ruvA	1787787	1788389	Holliday junction DNA helicase RuvA	L	11	3	0.27	2.2	3	0.27	0.1
rplC	2406590	2407252	50S ribosomal protein L3	J	11	3	0.27	2.2	1	0.09	0.3
SAR1256	1318209	1318913	short chain dehydrogenase	R	28	15	0.54	2.2	13	0.46	2.4
isdB	1146876	1148834	iron-regulated heme-iron binding protein	M	43	27	0.63	2.2	24	0.56	6.1
dltD	933225	934400	lipoteichoic acid biosynthesis protein	M	24	12	0.50	2.2	6	0.25	0.2
SAR2026	2117088	2117543	GntR family regulatory protein	K	24	12	0.50	2.2	5	0.21	0.0
clpP	866154	866741	ATP-dependent Clp protease proteolytic subunit	O	16	6	0.38	2.2	1	0.06	0.7
SAR1080	1126123	1126737	hypothetical protein	S	16	6	0.38	2.2	6	0.38	0.9
SAR1648	1715571	1716518	PhoH-like protein	T	16	6	0.38	2.2	0	0.00	1.5

Gene	Start	End	Product	COG	# Core BiPs	# Homo-plasies	Homoplasy rate	Poisson test: −log10(p)	# Non-synonymous BiPs	Non-synonymous probability	Binomial test: −log10(p)
SAR0750	784503	784985	hypothetical protein	S	19	8	0.42	2.2	6	0.32	0.6
SAR2396	2467344	2468036	DeoR family regulatory protein	K	19	8	0.42	2.2	6	0.32	0.6
SAR2525	2600813	2601286	hypothetical protein	S	19	8	0.42	2.2	12	0.63	4.0
purF	1089220	1090704	amidophosphoribosyltransferase	F	59	40	0.68	2.2	5	0.08	1.8
SAR1683	1745773	1747005	hypothetical protein	P	51	33	0.65	2.2	8	0.16	0.4
engA	1577261	1578571	GTP-binding protein EngA	R	41	25	0.61	2.3	0	0.00	4.1
SAR1986	2074353	2075423	DNA polymerase IV	L	23	11	0.48	2.3	4	0.17	0.1
gatA	2081022	2082479	aspartyl/glutamyl-tRNA amidotransferase subunit A	J	30	16	0.53	2.3	5	0.17	0.2
SAR2008	2100020	2100814	prephenate dehydratase	E	26	13	0.50	2.3	9	0.35	0.8
SAR2022	2113905	2114645	hypothetical protein		26	13	0.50	2.3	5	0.19	0.0
SAR1687	1749595	1750605	hypothetical protein	E	49	31	0.63	2.3	25	0.51	5.3
dltB	931760	932974	activated D-alanine transport protein	M	15	5	0.33	2.3	2	0.13	0.1
glpF	1338530	1339348	glycerol uptake facilitator protein	G	15	5	0.33	2.3	1	0.07	0.7
SAR1473	1566697	1567272	hypothetical protein	S	15	5	0.33	2.3	6	0.40	1.0
SAR1677	1742493	1742762	hypothetical protein		8	1	0.13	2.3	1	0.13	0.0
pknB	1244352	1246346	serine/threonine-protein kinase	S	56	37	0.66	2.3	10	0.18	0.2
leuB	2208671	2209717	3-isopropylmalate dehydrogenase	E	33	18	0.55	2.4	8	0.24	0.2
alr	2226416	2227564	alanine racemase	M	33	18	0.55	2.4	9	0.27	0.4
SAR0212	246251	246745	hypothetical protein	S	10	2	0.20	2.4	5	0.50	1.4
SAR0762	797425	798009	hypothetical protein		10	2	0.20	2.4	3	0.30	0.3
SAR1204	1254257	1254814	fatty acid biosynthesis transcriptional regulator	Q	10	2	0.20	2.4	3	0.30	0.3
hisS	1773038	1774300	histidyl-tRNA synthetase	J	22	10	0.45	2.4	4	0.18	0.0
kdpE	2237966	2238661	response regulator protein		22	10	0.45	2.4	8	0.36	0.9
dal	1469514	1470599	alanine racemase	M	43	26	0.60	2.4	15	0.35	1.4
SAR1419	1475682	1477025	branched-chain amino acid transporter protein	E	25	12	0.48	2.4	4	0.16	0.2
SAR1840	1922810	1924078	hypothetical protein	R	47	29	0.62	2.4	13	0.28	0.5
tcaR	2512890	2513345	MarR family regulatory protein	K	6	0	0.00	2.4	1	0.17	0.0
SAR2500	2572572	2572934	lipoprotein		6	0	0.00	2.4	4	0.67	1.7
SAR2119	2179911	2181125	membrane anchored protein		38	22	0.58	2.4	16	0.42	2.4
SAR1152	1195416	1195856	hypothetical protein		12	3	0.25	2.4	0	0.00	1.1
srrA	1642399	1643142	response regulator protein	T	12	3	0.25	2.4	1	0.08	0.3
moaC	2429532	2430026	molybdenum cofactor biosynthesis protein MoaC	H	12	3	0.25	2.4	6	0.50	1.6
sstD	828218	829246	lipoprotein	P	54	35	0.65	2.4	8	0.15	0.5
SAR0474	506930	508570	glycosyl hydrolase	G	59	39	0.66	2.4	21	0.36	2.0
pyrG	2282154	2283764	CTP synthetase	F	59	39	0.66	2.4	4	0.07	2.4
odhB	1481753	1483024	dihydrolipoamide succinyltransferase	C	37	21	0.57	2.5	13	0.35	1.2
SAR0322	367614	368414	hypothetical protein	R	41	24	0.59	2.5	13	0.32	0.9
SAR2640	2727749	2728747	D-lactate dehydrogenase	R	24	11	0.46	2.5	9	0.38	1.1
SAR1281	1345880	1347118	hypothetical protein	R	36	20	0.56	2.5	10	0.28	0.4
SAR1449	1537076	1538398	amino acid permease	E	56	36	0.64	2.5	1	0.02	4.3
SAR1083	1128168	1130015	BipA family GTPase	T	40	23	0.58	2.5	6	0.15	0.4
ald1	1860507	1861625	alanine dehydrogenase	E	40	23	0.58	2.5	9	0.23	0.1
SAR2013	2106200	2107579	aldehyde dehydrogenase	C	40	23	0.58	2.5	11	0.28	0.5
SAR1357	1405176	1408205	exonuclease	L	109	82	0.75	2.5	47	0.43	6.4
cysS	582365	583765	cysteinyl-tRNA synthetase	J	44	26	0.59	2.5	11	0.25	0.2
SAR1934	2013818	2016754	hypothetical protein	S	74	51	0.69	2.5	13	0.18	0.3
ctaA	1137034	1137945	heme A synthase	O	20	8	0.40	2.6	2	0.10	0.5
SAR1459	1550611	1550961	hypothetical protein		20	8	0.40	2.6	5	0.25	0.1
ilvB	2203945	2205714	acetolactate synthase large subunit	H	55	35	0.64	2.6	12	0.22	0.1
SAR2671	2757172	2757894	hypothetical protein	S	23	10	0.43	2.6	3	0.13	0.3

Gene	Start	End	Product	COG	# Core BiPs	# Homo-plasies	Homoplasy rate	Poisson test: −log10(p)	# Non-synonymous BiPs	Non-synonymous probability	Binomial test: −log10(p)
folK	555945	556421	2-amino-4-hydroxy-6-hydroxymethyldihydropteridine pyrophosphokinase	H	17	6	0.35	2.6	8	0.47	1.8
SAR0570	626449	627327	GTP cyclohydrolase	S	26	12	0.46	2.6	6	0.23	0.1
SAR2028	2118442	2119728	hypothetical protein	E	59	38	0.64	2.6	17	0.29	0.7
SAR1813	1892036	1893205	histone deacetylase family protein	Q	49	30	0.61	2.6	17	0.35	1.5
SAR0805	843078	843752	hypothetical protein	R	14	4	0.29	2.6	8	0.57	2.4
SAR1075	1120828	1121637	ABC transporter permease	E	14	4	0.29	2.6	0	0.00	1.3
moeA	2428210	2429469	molybdenum cofactor biosynthesis protein	H	34	18	0.53	2.6	6	0.18	0.2
SAR0682	729496	730140	hypothetical protein	S	22	9	0.41	2.7	4	0.18	0.0
SAR0794	831583	832461	hypothetical protein		22	9	0.41	2.7	8	0.36	0.9
citB	1412719	1415424	aconitate hydratase	C	57	36	0.63	2.7	7	0.12	1.0
SAR2210	2276632	2278059	aldehyde dehydrogenase family protein	C	57	36	0.63	2.7	12	0.21	0.0
SAR0357	404255	405100	DNA-binding protein	K	25	11	0.44	2.7	7	0.28	0.3
thiM	2251590	2252381	hydroxyethylthiazole kinase	H	25	11	0.44	2.7	15	0.60	4.5
SAR0306	348699	349376	ABC transporter ATP-binding protein	V	52	32	0.62	2.7	10	0.19	0.1
SAR1344	1394246	1395763	catalase	P	37	20	0.54	2.7	6	0.16	0.3
SAR1980	2068690	2069676	hypothetical protein	S	37	20	0.54	2.7	8	0.22	0.0
SAR0670	715951	716991	sensor histidine kinase protein	T	47	28	0.60	2.7	5	0.11	1.1
SAR1124	1173653	1174240	nucleoside-triphosphatase	F	29	14	0.48	2.7	5	0.17	0.1
gcvT	1686468	1687559	glycine cleavage system aminomethyltransferase T	E	29	14	0.48	2.7	9	0.31	0.6
dltA	930306	931763	D-alanine--poly(phosphoribitol) ligase subunit 1	Q	36	19	0.53	2.8	14	0.39	1.6
kdpB	2231315	2233342	potassium-transporting ATPase subunit B	P	67	44	0.66	2.8	20	0.30	1.0
SAR1642	1711125	1712516	glycyl-tRNA synthetase	J	21	8	0.38	2.8	2	0.10	0.5
hemB	1811001	1811975	delta-aminolevulinic acid dehydratase	H	32	16	0.50	2.8	2	0.06	1.5
SAR2004	2095585	2096187	hypothetical protein	S	11	2	0.18	2.8	2	0.18	0.0
SAR0867	907672	908058	hypothetical protein	L	9	1	0.11	2.8	4	0.44	1.0
SAR1355	1403460	1403927	hypothetical protein	O	9	1	0.11	2.8	5	0.56	1.6
SAR1210	1258984	1262550	chromosome partition protein	D	84	58	0.69	2.8	18	0.21	0.0
SAR1018	1060526	1061329	hydrolase	R	28	13	0.46	2.8	15	0.54	3.7
SAR1741	1802331	1803038	type III leader peptidase family protein	O	28	13	0.46	2.8	10	0.36	1.0
SAR1971	2061900	2062364	low molecular weight phosphotyrosine protein phosphatase	T	13	3	0.23	2.8	2	0.15	0.0
SAR2505	2576860	2578299	transporter	G	53	32	0.60	2.8	9	0.17	0.3
leuS	1931421	1933835	leucyl-tRNA synthetase	J	72	48	0.67	2.9	8	0.11	1.5
ksgA	531901	532794	dimethyladenosine transferase	J	48	28	0.58	2.9	8	0.17	0.3
SAR0743	777524	779077	sodiumsulfate symporter protein	P	71	47	0.66	2.9	17	0.24	0.2
SAR1685	1747769	1749130	biotin carboxylase subunit of acetyl-CoA carboxylase	I	34	17	0.50	2.9	11	0.32	0.9
SAR2021	2113278	2113841	hypothetical protein		7	0	0.00	2.9	3	0.43	0.8
SAR0741	776011	776919	aldo/keto reductase family protein	R	24	10	0.42	2.9	8	0.33	0.7
SAR1937	2019241	2020365	hypothetical protein	S	43	24	0.56	2.9	16	0.37	1.8
SAR2130	2190923	2192173	ammonium transporter family protein	P	75	50	0.67	2.9	6	0.08	2.5
SAR0740	774982	775908	cobalamin synthesis protein	R	33	16	0.48	3.0	16	0.48	3.1
SAR2340	2412296	2413183	acetyltransferase (GNAT) family protein		33	16	0.48	3.0	9	0.27	0.4
SAR2528	2603209	2604618	amino acid permease	E	33	16	0.48	3.0	5	0.15	0.3
SAR0684	731256	732476	sugar efflux transporter	G	42	23	0.55	3.0	14	0.33	1.1
SAR0865	906203	907081	hypothetical protein	S	26	11	0.42	3.0	15	0.58	4.2
SAR1338	1387639	1388919	homoserine dehydrogenase	E	26	11	0.42	3.0	7	0.27	0.3
SAR1277	1342955	1343869	hydrolase	I	41	22	0.54	3.0	16	0.39	1.9
SAR1055	1101929	1102471	hypothetical protein		20	7	0.35	3.0	1	0.05	1.0
fib	1177712	1178209	fibrinogen-binding protein precursor		20	7	0.35	3.0	5	0.25	0.1

Gene	Start	End	Product	COG	# Core BiPs	# Homo-plasies	Homoplasy rate	Poisson test: −log10(p)	# Non-synonymous BiPs	Non-synonymous probability	Binomial test: −log10(p)
SAR1167	1211166	1211972	hypothetical protein	S	20	7	0.35	3.0	12	0.60	3.7
SAR1988	2076370	2077731	RNA methyltransferase	J	37	19	0.51	3.1	16	0.43	2.4
SAR0554	603466	604641	peptidase	R	32	15	0.47	3.1	6	0.19	0.1
SAR0903	937831	939039	pyridine nucleotide-disulphide oxidoreductase	C	12	2	0.17	3.1	3	0.25	0.1
SAR1061	1107790	1108452	potassium transport protein	P	12	2	0.17	3.1	1	0.08	0.3
SAR1945	2027317	2027916	hypothetical protein	G	12	2	0.17	3.1	2	0.17	0.0
SAR2020	2112633	2113157	hypothetical protein		12	2	0.17	3.1	1	0.08	0.3
mobA	2426423	2427022	molybdopterin-guanine dinucleotide biosynthesis protein MobA	H	12	2	0.17	3.1	9	0.75	4.0
SAR0563	613927	614397	deaminase	F	10	1	0.10	3.1	3	0.30	0.3
udk	1752159	1752782	uridine kinase	F	10	1	0.10	3.1	1	0.10	0.2
SAR1875	1962378	1962635	hypothetical protein	S	10	1	0.10	3.1	3	0.30	0.3
SAR0526	572320	572886	hypothetical protein	S	14	3	0.21	3.1	3	0.21	0.0
pgsA	1320290	1320868	CDP-diacylglycerol--glycerol-3-phosphate 3-phosphatidyltransferase	I	14	3	0.21	3.1	3	0.21	0.0
SAR0255	296636	298360	glycosyl transferase	M	83	56	0.67	3.1	12	0.14	0.9
SAR0580	633883	635259	AMP-binding enzyme	Q	70	45	0.64	3.1	41	0.59	10.8
sstB	826389	827345	FecCD transport family protein	P	40	21	0.53	3.1	11	0.28	0.5
SAR1668	1736512	1737228	hypothetical protein		25	10	0.40	3.1	13	0.52	3.1
SAR1821	1904295	1905152	hypothetical protein	S	25	10	0.40	3.1	6	0.24	0.1
SAR2507	2579743	2580609	cation efflux family protein	P	25	10	0.40	3.1	5	0.20	0.0
SAR2446	2515403	2516458	permease protein	V	36	18	0.50	3.1	13	0.36	1.4
SAR1627	1695584	1696123	5-formyltetrahydrofolate cyclo-ligase family protein	H	22	8	0.36	3.1	9	0.41	1.4
SAR1941	2023286	2024107	RNA pseudouridylate synthase	J	22	8	0.36	3.1	3	0.14	0.2
grlA	1419760	1422162	DNA topoisomerase IV subunit A	L	53	31	0.58	3.2	15	0.28	0.6
pdhB	1114026	1115003	pyruvate dehydrogenase E1 component, beta subunit	C	19	6	0.32	3.2	1	0.05	1.0
SAR2533	2611814	2612749	2-dehydropantoate 2-reductase	H	35	17	0.49	3.2	14	0.40	1.9
SAR2178	2248729	2249376	hypothetical protein	R	24	9	0.38	3.2	16	0.67	5.6
hprK	857139	858071	HPr kinase/phosphorylase	T	31	14	0.45	3.3	0	0.00	3.1
SAR1798	1872365	1873108	hypothetical protein	C	31	14	0.45	3.3	15	0.48	2.9
SAR2463	2533656	2534933	hypothetical protein	S	41	21	0.51	3.3	12	0.29	0.6
SAR1194	1242511	1243605	ribosomal RNA large subunit methyltransferase N	R	18	5	0.28	3.4	3	0.17	0.1
SAR1783	1856055	1857353	DNA-binding protein	K	30	13	0.43	3.4	2	0.07	1.3
moeB	2430563	2431567	molybdopterin synthase sulfurylase	H	30	13	0.43	3.4	12	0.40	1.6
SAR0665	711729	712772	esterase	I	40	20	0.50	3.4	11	0.28	0.5
SAR0662	709080	709442	phosphotransferase mannnose-specific family component IIA	S	23	8	0.35	3.4	9	0.39	1.2
thiE	2250947	2251588	thiamine-phosphate pyrophosphorylase	H	23	8	0.35	3.4	10	0.43	1.7
SAR2169	2241054	2242412	UDP-N-acetylmuramoylalanyl-D-glutamyl-2, 6-diaminopimelate-- D-alanyl-D-alanyl ligase	M	49	27	0.55	3.4	17	0.35	1.5
SAR1452	1541157	1542035	5'-3' exonuclease	L	45	24	0.53	3.4	13	0.29	0.7
lysP	1822743	1824236	lysine-specific permease	E	79	51	0.65	3.4	7	0.09	2.3
SAR2151	2220196	2222346	RNA binding protein	K	79	51	0.65	3.4	15	0.19	0.2
glpD	1341084	1342805	aerobic glycerol-3-phosphate dehydrogenase	C	58	34	0.59	3.4	9	0.16	0.5
SAR0669	715284	715958	response regulator protein	T	20	6	0.30	3.4	2	0.10	0.5
fus	599703	601784	elongation factor G	J	26	10	0.38	3.5	0	0.00	2.5
SAR1599	1673697	1674578	geranyltranstransferase	H	26	10	0.38	3.5	10	0.38	1.3
trap	2006656	2007159	signal transduction protein		26	10	0.38	3.5	7	0.27	0.3

Gene	Start	End	Product	COG	# Core BiPs	# Homo-plasies	Homoplasy rate	Poisson test: −log10(p)	# Non-synonymous BiPs	Non-synonymous probability	Binomial test: −log10(p)
SAR1072	1118386	1118925	DNA-binding protein	K	15	3	0.20	3.5	2	0.13	0.1
glyA	2268409	2269647	serine hydroxymethyltransferase	E	15	3	0.20	3.5	3	0.20	0.0
SAR0487	525697	526044	DNA replication intiation control protein YabA	S	11	1	0.09	3.6	4	0.36	0.6
folB	555583	555948	dihydroneopterin aldolase	H	11	1	0.09	3.6	2	0.18	0.0
infB	1302598	1304715	translation initiation factor IF-2	J	46	24	0.52	3.6	5	0.11	1.0
glpQ	956700	957629	glycerophosphoryl diester phosphodiesterase	C	31	13	0.42	3.6	8	0.26	0.3
rocF	2330622	2331530	arginase	E	31	13	0.42	3.6	10	0.32	0.7
purB	2091471	2092766	adenylosuccinate lyase	F	34	15	0.44	3.6	4	0.12	0.7
relA	1776096	1778306	GTP pyrophosphokinase	T	49	26	0.53	3.6	6	0.12	0.8
rimM	1266410	1266913	16S rRNA-processing protein RimM	J	22	7	0.32	3.7	10	0.45	1.8
SAR0490	527286	528125	tetrapyrrole (corrin/porphyrin) methylase family protein	R	30	12	0.40	3.7	11	0.37	1.2
SAR0275	319607	320527	hypothetical protein		53	29	0.55	3.7	20	0.38	2.2
SAR2393	2461526	2464480	bifunctional protein	R	159	118	0.74	3.8	17	0.11	3.2
SAR1410	1468358	1469509	peptidase	R	40	19	0.48	3.8	14	0.35	1.3
arsB2	1946500	1947792	arsenical pump membrane protein 2	P	40	19	0.48	3.8	11	0.28	0.5
SAR2220	2288025	2289218	hypothetical protein	I	40	19	0.48	3.8	17	0.43	2.5
SAR0884	922532	923380	hypothetical protein	S	21	6	0.29	3.8	8	0.38	1.0
SAR2368	2437923	2438831	ferrichrome-binding lipoprotein precursor	P	21	6	0.29	3.8	4	0.19	0.0
oppF	994988	995929	oligopeptide transport ATP-binding protein	R	42	20	0.48	3.9	10	0.24	0.2
SAR2339	2409974	2412109	DNA topoisomerase III	L	58	32	0.55	3.9	17	0.29	0.8
SAR0862	904667	904987	thioredoxin	C	23	7	0.30	3.9	6	0.26	0.2
SAR2369	2439044	2440198	hypothetical protein	I	32	13	0.41	3.9	11	0.34	1.1
moaA	2425381	2426403	molybdenum cofactor biosynthesis protein A	H	35	15	0.43	3.9	10	0.29	0.5
tyrS	1881637	1882899	tyrosyl-tRNA synthetase	J	57	31	0.54	4.0	11	0.19	0.1
SAR2179	2249989	2250861	hypothetical protein	U	16	3	0.19	4.0	2	0.13	0.3
SAR1939	2021366	2021989	response regulator	T	18	4	0.22	4.0	3	0.17	0.1
SAR2397	2468061	2468798	hypothetical protein		18	4	0.22	4.0	10	0.56	2.8
folC	1803299	1804570	folylpolyglutamate synthase	H	25	8	0.32	4.0	6	0.24	0.1
hutU	2485512	2487173	urocanate hydratase	E	56	30	0.54	4.0	9	0.16	0.4
atpH	2263819	2264358	F0F1 ATP synthase subunit delta	C	12	1	0.08	4.0	5	0.42	0.8
rplD	2405940	2406563	50S ribosomal protein L4	J	12	1	0.08	4.0	2	0.17	0.0
glcU	2413364	2414227	glucose uptake protein	G	12	1	0.08	4.0	1	0.08	0.3
SAR1622	1691993	1692616	metallo-beta-lactamase superfamily protein	R	20	5	0.25	4.0	4	0.20	0.0
SAR1757	1820605	1821213	hypothetical protein	R	20	5	0.25	4.0	4	0.20	0.0
SAR1442	1499618	1500742	hypothetical protein	C	52	27	0.52	4.0	20	0.38	2.2
kbl	604812	605999	2-amino-3-ketobutyrate coenzyme A ligase	H	34	14	0.41	4.0	7	0.21	0.0
SAR2338	2408527	2409861	xanthine/uracil permease	R	37	16	0.43	4.0	4	0.11	0.8
SAR2214	2281524	2282045	hypothetical protein		22	6	0.27	4.1	8	0.36	0.9
SAR1397	1453266	1455080	peptidase	E	51	26	0.51	4.1	19	0.37	2.0
SAR1192	1240269	1241204	methionyl-tRNA formyltransferase	J	36	15	0.42	4.1	8	0.22	0.1
pepT	834672	835898	peptidase T	E	43	20	0.47	4.2	13	0.30	0.7
uvrB	851683	853674	excinuclease ABC subunit B	L	81	49	0.60	4.2	5	0.06	3.5
SAR0934	976684	977508	haloacid dehalogenase-like hydrolase	R	17	3	0.18	4.2	2	0.12	0.3
purE	1083822	1084304	phosphoribosylaminoimidazole carboxylase catalytic subunit	F	17	3	0.18	4.2	9	0.53	2.4
SAR2433	2502738	2503229	hypothetical protein	R	17	3	0.18	4.2	6	0.35	0.6
SAR1247	1305622	1306539	tRNA pseudouridine synthase B	J	27	9	0.33	4.2	5	0.19	0.0
SAR2207	2273751	2274350	thymidine kinase	F	15	2	0.13	4.2	3	0.20	0.0
SAR0780	816277	817194	diacylglycerol kinase protein	R	32	12	0.38	4.3	5	0.16	0.3
SAR1492	1587913	1588161	ferredoxin	C	13	1	0.08	4.3	3	0.23	0.1

Gene	Start	End	Product	COG	# Core BiPs	# Homo-plasies	Homoplasy rate	Poisson test: −log10(p)	# Non-synonymous BiPs	Non-synonymous probability	Binomial test: −log10(p)
SAR2385	2452560	2453960	Na+/H+ antiporter	C	45	21	0.47	4.3	20	0.44	3.1
SAR0560	611789	612472	haloacid dehalogenase-like hydrolase	R	29	10	0.34	4.3	7	0.24	0.2
clpX	1816780	1818042	ATP-dependent protease ATP-binding subunit ClpX	O	29	10	0.34	4.3	4	0.14	0.3
hemL	1809667	1810953	glutamate-1-semialdehyde aminotransferase	H	48	23	0.48	4.3	6	0.13	0.8
SAR1636	1704237	1705337	hypothetical protein	S	38	16	0.42	4.4	23	0.61	6.7
SAR1398	1455220	1455861	phosphate transporter	P	41	18	0.44	4.4	5	0.12	0.7
SAR1970	2061553	2061768	hypothetical protein	S	19	4	0.21	4.4	6	0.32	0.6
cbiO	2389811	2390671	cobalt transporter ATP-binding subunit	P	63	34	0.54	4.4	13	0.21	0.0
SAR0953	995948	997603	transport system extracellular binding lipoprotein	E	103	66	0.64	4.4	28	0.27	0.8
SAR2493	2567378	2568202	nitrite transporter	P	28	9	0.32	4.5	4	0.14	0.3
SAR1356	1404051	1405172	exonuclease	L	40	17	0.43	4.5	15	0.38	1.7
trpA	1443500	1444228	tryptophan synthase subunit alpha	E	34	13	0.38	4.5	10	0.29	0.5
SAR1819	1899783	1903607	FtsK/SpoIIIE family protein	D	150	105	0.70	4.5	82	0.55	18.2
sstC	827342	828103	ABC transporter ATP-binding protein	P	57	29	0.51	4.6	7	0.12	1.0
valS	1804583	1807213	valyl-tRNA synthetase	J	66	36	0.55	4.6	14	0.21	0.0
SAR2221	2289218	2290402	peptidase	R	39	16	0.41	4.6	8	0.21	0.0
rluB	1643257	1643994	ribosomal large subunit pseudouridine synthase B	J	53	26	0.49	4.6	3	0.06	2.4
SAR1665	1733027	1735228	hypothetical protein	R	79	46	0.58	4.6	37	0.47	6.2
polA	1832736	1835366	DNA polymerase I	L	69	38	0.55	4.6	18	0.26	0.4
SAR1023	1065610	1066764	aminotransferase	E	41	17	0.41	4.7	7	0.17	0.2
SAR1626	1694109	1695572	hypothetical protein	R	35	13	0.37	4.7	8	0.23	0.1
SAR2457	2528101	2528730	hypothetical protein		14	1	0.07	4.8	3	0.21	0.0
SAR0754	789183	790364	N-acetylglucosamine-6-phosphate deacetylase	G	54	26	0.48	4.8	16	0.30	0.9
ileS	1212813	1215566	isoleucyl-tRNA synthetase	J	133	89	0.67	4.8	21	0.16	0.9
SAR1418	1474478	1475614	hypothetical protein	P	47	21	0.45	4.8	18	0.38	2.1
SAR0800	837381	838451	hypothetical protein	T	29	9	0.31	4.8	1	0.03	1.9
modA	2433026	2433808	molybdate-binding lipoprotein precursor	P	29	9	0.31	4.8	7	0.24	0.2
SAR0304	346498	347388	hypothetical protein	R	53	25	0.47	4.9	9	0.17	0.3
SAR2391	2459728	2460504	N-acetylmuramoyl-L-alanine amidase	G	24	6	0.25	4.9	8	0.33	0.7
uvrA	853682	856528	excinuclease ABC subunit A	L	143	97	0.68	4.9	17	0.12	2.4
lgt	858077	858919	prolipoprotein diacylglyceryl transferase	M	26	7	0.27	5.0	10	0.38	1.3
dnaE	1851585	1854782	DNA polymerase III alpha subunit	L	123	80	0.65	5.0	30	0.24	0.4
moaB	2430028	2430534	molybdenum cofactor biosynthesis protein B	H	28	8	0.29	5.0	9	0.32	0.8
SAR1790	1863802	1864749	hypothetical protein	L	30	9	0.30	5.1	9	0.30	0.6
SAR1756	1819657	1820586	hypothetical protein		36	13	0.36	5.1	12	0.33	1.0
pyrB	1221834	1222715	aspartate carbamoyltransferase catalytic subunit	F	19	3	0.16	5.1	3	0.16	0.1
SAR2420	2489134	2490069	formimidoylglutamase	E	41	16	0.39	5.2	10	0.24	0.2
clpC	573897	576353	stress response-related Clp ATPase	O	60	29	0.48	5.2	7	0.12	1.1
nadE	2096457	2097278	NAD synthetase	H	43	17	0.40	5.3	6	0.14	0.6
SAR1053	1098983	1100278	hypothetical protein		32	10	0.31	5.3	5	0.16	0.3
SAR1922	2002078	2002632	hypothetical protein	R	32	10	0.31	5.3	2	0.06	1.5
SAR0493	531354	531890	hypothetical protein	L	37	13	0.35	5.3	7	0.19	0.1
SAR1116	1162369	1164081	hypothetical protein	L	52	23	0.44	5.4	10	0.19	0.1
secA	844799	847330	preprotein translocase subunit SecA	U	70	36	0.51	5.4	6	0.09	2.1
SAR1401	1457685	1458611	ABC transporter permease	P	30	8	0.27	5.7	1	0.03	1.9
dapB	1466747	1467469	dihydrodipicolinate reductase	E	40	14	0.35	5.8	10	0.25	0.2
SAR2014	2107699	2108727	hypothetical protein	G	40	14	0.35	5.8	13	0.33	0.9

Gene	Start	End	Product	COG	# Core BiPs	# Homo-plasies	Homoplasy rate	Poisson test: −log10(p)	# Non-synonymous BiPs	Non-synonymous probability	Binomial test: −log10(p)
SAR1837	1919614	1920036	hypothetical protein		25	5	0.20	5.8	8	0.32	0.7
tuf	602001	603185	elongation factor Tu	J	21	3	0.14	5.8	1	0.05	1.2
SAR2138	2200614	2201276	hypothetical protein	O	21	3	0.14	5.8	9	0.43	1.5
folD	1082761	1083621	bifunctional 5,10-methylene-tetrahydrofolate dehydrogenase/ 5,10-methylene-tetrahydrofolate cyclohydrolase	H	32	9	0.28	5.9	7	0.22	0.0
purL	1087052	1089241	phosphoribosylformylglycinamidine synthase II	F	75	38	0.51	5.9	13	0.17	0.3
prfA	2272674	2273750	peptide chain release factor 1	J	22	3	0.14	6.3	3	0.14	0.2
SAR2382	2448749	2450704	transcriptional regulator	K	45	16	0.36	6.3	27	0.60	7.6
SAR0650	696099	697160	glycosyl transferase	R	44	15	0.34	6.5	22	0.50	4.6
SAR0329	373878	375833	PTS regulator	K	90	47	0.52	6.6	28	0.31	1.5
mutS	1332867	1335485	DNA mismatch repair protein MutS	L	68	31	0.46	6.6	15	0.22	0.1
SAR1969	2060294	2061541	aminopeptidase	E	70	32	0.46	6.7	12	0.17	0.3
SAR0746	780972	781592	hypothetical protein	S	37	10	0.27	6.8	7	0.19	0.1
putP	2083150	2084688	high affinity proline permease	R	50	18	0.36	6.9	11	0.22	0.1
SAR1448	1535528	1536919	transporter protein	G	56	22	0.39	6.9	9	0.16	0.4
oppD	993916	994998	oligopeptide transport ATP-binding protein	P	32	7	0.22	6.9	8	0.25	0.2
alaS	1756989	1759619	alanyl-tRNA synthetase	J	72	32	0.44	7.2	21	0.29	0.9
SAR1153	1196051	1197664	hypothetical protein	S	60	23	0.38	7.6	31	0.52	6.6
SAR0804	842003	843085	helicase	L	47	14	0.30	8.0	20	0.43	3.0
SAR1846	1936369	1936932	hypothetical protein	M	27	3	0.11	8.2	10	0.37	1.2
SAR1839	1920745	1922406	polysaccharide biosynthesis protein	R	94	45	0.48	8.2	4	0.04	5.2
SAR1371	1425358	1426566	hypothetical protein	R	39	8	0.21	8.9	0	0.00	3.9
mscL	1409939	1410301	large-conductance mechanosensitive channel	M	30	3	0.10	9.4	4	0.13	0.4
SAR2205	2271851	2272687	hypothetical protein	J	40	7	0.18	9.9	9	0.23	0.1
SAR0781	817464	818969	proton-dependent oligopeptide transport protein	E	65	21	0.32	10.0	3	0.05	3.4
SAR2421	2490347	2491606	hypothetical protein	R	65	21	0.32	10.0	15	0.23	0.1
radA	576837	578201	DNA repair protein RadA	O	35	4	0.11	10.5	7	0.20	0.0
ccpA	1893303	1894292	catabolite control protein A	K	61	17	0.28	10.9	8	0.13	0.9
SAR2651	2740994	2742049	hypothetical protein	R	79	28	0.35	10.9	14	0.18	0.3
hemA	1814473	1815819	glutamyl-tRNA reductase	H	37	4	0.11	11.4	11	0.30	0.6
SAR1077	1122784	1123803	hypothetical protein		53	10	0.19	12.5	13	0.25	0.2
ackA	1862513	1863715	acetate kinase	C	66	17	0.26	12.5	3	0.05	3.6
rho	2275069	2276385	transcription termination factor Rho	K	67	17	0.25	12.9	3	0.04	3.6

Supplementary Table 5 | Query sequences and BLAST thresholds used for detection of mobile elements.

Mobile element	Query sequence	Accession number (bases)	Identity threshold (%)
Phage Sa1	Phage Sa1 integrase gene	BA000017.4 (918952-920001)	90
Phage Sa2	Phage Sa2 integrase gene	CP000255.1 (1590733-1591938)	90
Phage Sa3	Phage Sa3 integrase gene	FN433596.1 (2151977- 2153014)	90
Phage Sa4	Phage Sa4 integrase gene	BX571857.1 (981150-981752)	90
Phage Sa5	Phage Sa5 integrase gene	AP009351.1 (2022127-2023173)	90
Phage Sa6	Phage Sa6 integrase gene	CP000046.1 (354785-355990)	90
Phage Sa7	Phage Sa7 integrase gene	AP009351.1 (1098898-1100283)	90
Phage Sa8	Phage Sa8 integrase gene	AJ938182.1 (1886112-1887176)	90
SaPI1	SaPI1 integrase gene	U93688.2 (13875-15095)	90
SaPI2	SaPI2 integrase gene	EF010993.1 (87- 1259)	90
SaPI3	SaPI3 integrase gene	AF410775.1 (14574-15794)	90
SaPI4	SaPI4 integrase gene	BX571856.1 (410176- 411390)	90
SaPI5	SaPI5 integrase gene	CP000255.1 (881996- 883216)	90
SaPIbov1	SaPIbov1 integrase gene	AF217235.1 (14721-15857)	90
SaPIm4	SaPIm4 integrase gene	BA000017.4 (868462-869568)	90
SaPIMW2	SaPIMW2 integrase gene	BA000033.2 (839705-840820)	90
SaPIn1	SaPIn1 integrase gene	BA000017.4 (2147530-2148702)	90
SaPI122	SaPI122 integrase gene	AJ938182.1 (2037180-2038352)	90
SCC	*ccrA*	BX571856.1 (64935- 66284)	60
SCC	*ccrB*	BX571856.1 (63285-64913)	60
SCC	*ccrC*	AP006716.1 (64143-65819)	60
SCCmec	*mecA*	BX571856.1 (44919-46925)	60
SCCmec	*mecR1*	BX571856.1 (47025-48782)	60
ICE6013	ICE6013	BX571856.1 (1351109-1364463)	70
Tn552	Tn552	BX571856.1 (1908514-1914672)	50
Tn554	Tn554	BX571856.1 (57793-61150)	50
Cadmium Resistance Cassette (with Tn554 transposase)	Cadmium Resistance Cassette	HQ634347.1	70
Tn5801	Tn5801	FN433596.1 (486820- 518088)	20
Tn4001	Tn4001	AP003367.1 (16762-19397)	50
Integrated plasmid	Integrated plasmid	BX571856.1 (734448-764374)	30

Supplementary Table 6 I Locations of mobile elements detected by BLAST and variable core BiP distance. Known *S. aureus* mobile elements were queried in all 110 genomes using BLAST, and the positions of flanking core BiPs recorded. Evidence for mobile activity was detected from Velvet assemblies by calculating the standardized variance in inter-core BiP distance. Excessive variability in distance was taken as evidence for mobile activity. Hits from BLAST and VCBD were curated manually to produce non-overlapping annotations of mobile elements. Locations are given relative to the MRSA252 reference genome.

Element Name	BLAST hits to known mobile elements				Variable core BiP distance		Manual Curation	
	**Description**	**Start**	**End**	**Feature**	**Start**	**End**	**Start**	**End**
scc_33	SCCmec type II	33662	104040	SCC	34079	102709	33662	111588
	SCCmec type IV	33662	104040	SCC	102739	102856		
	SCCfar	33662	104040	SCC	102910	102940		
	Non-mec SCC with ccrA1 and ccrB3 genes	33662	104040	SCC	103019	103089		
					103115	103183		
					104386	108831		
					109316	109485		
					110662	111588		
transposon_120	ICE6013	120822	121171	transposon	120962	121039	120822	121171
unclassified_226					226433	227532	226433	227532
plasmid_258	Plasmid	258197	260396	plasmid			258197	260396
	Cadmium resistance cassette	258197	260396	transposon				
unclassified_271					271271	271437	271271	271437
unclassified_303					303178	306078	303178	306078
transposon_321	ICE6013	321691	344606	transposon	323431	323596	321691	344606
					323699	343958		
phage_361	*S. aureus* phage type VI	361106	363838	phage			361106	363838
sapi_409	SAPI	409113	426412	SAPI	409022	424303	409022	428228
	SAPI	409113	426412	SAPI	428069	428187		
	ICE6013	427936	428228	transposon				
unclassified_431					431121	432691	431121	432691
island_446	Genomic island α	446300	474705	island	446245	450048	446245	474705
	SAPI	446300	474705	SAPI	450423	450864		
					467460	473852		
unclassified_616					616498	623053	616498	623053
unclassified_654					654331	657165	654331	657165
unclassified_684					684883	686975	684883	686975
transposon_691	ICE6013	691056	692122	transposon	691376	691443	691056	692122
unclassified_794					794123	794128	794123	794128
unclassified_866					866767	868785	866767	868785
sapi_882	SAPI	882934	883125	SAPI	883970	889192	882934	891548
	SAPI	883591	891548	SAPI				
	SAPI	883591	891548	SAPI				
sapi_911	SAPI	911150	912392	SAPI	911107	911827	911107	912392
	SAPI	911150	912392	SAPI				
phage_919	*S. aureus* phage type I	919097	920112	phage	919014	919161	919014	920112
	ICE6013	919097	920112	transposon				
unclassified_984					984122	987064	984122	987064
unclassified_992					992212	992314	992212	994278
					993145	993156		
					994217	994278		

Element Name	BLAST hits to known mobile elements				Variable core BiP distance		Manual Curation	
	Description	Start	End	Feature	Start	End	Start	End
unclassified_1027					1027066	1029519	1027066	1029519
phage_1051	*S. aureus* phage type III	1053724	1055219	phage	1051140	1053361	1051140	1055219
phage_1136	*S. aureus* phage type VII	1136767	1137034	phage			1136767	1137034
transposon_1141	Tn552	1141608	1141673	transposon			1141608	1141673
	Tn552	1142604	1142841	transposon				
phage_1146	*S. aureus* phage type VII	1146470	1150308	phage	1146701	1146833	1146470	1150308
	ICE6013	1146470	1150308	transposon	1148924	1148959		
island_1174	Genome island γ	1174241	1196050	island			1174241	1196050
	S. aureus phage type II	1174241	1174232	phage				
	ICE6013	1174241	1174232	transposon				
transposon_1219	ICE6013	1219353	1219753	transposon	1219407	1219447	1219353	1219753
transposon_1229	ICE6013	1229931	1230366	transposon	1229960	1230069	1229931	1230366
transposon_1350	ICE6013	1350347	1376454	transposon	1350361	1376252	1350347	1376454
transposon_1442	ICE6013	1442301	1445830	transposon	1415465	1415559	1415465	1445830
					1444191	1444280		
plasmid_1447	ICE6013	1447090	1448568	transposon	1447084	1447717	1447084	1448568
	Plasmid	1447090	1448568	plasmid				
unclassified_1481					1481319	1481638	1481319	1481638
phage_1502	*S. aureus* phage type III	1502829	1538429	phage	1532365	1532386	1502829	1541166
	ICE6013	1502829	1538429	transposon	1540888	1541166		
	ICE6013	1539470	1541156	transposon				
phage_1588	*S. aureus* phage type II	1588813	1642398	phage	1589071	1640376	1588813	1642398
unclassified_1658					1658459	1661393	1658459	1661393
phage_1742	*S. aureus* phage type II	1742762	1747016	phage			1742762	1747016
transposon_1794	Tn554	1794869	1803298	transposon	1795037	1801838	1794869	1803298
plasmid_1908	Plasmid	1908069	1915145	plasmid	1908330	1915115	1908069	1915145
	Tn552	1908069	1915145	transposon				
unclassified_1947					1947730	1947912	1947730	1947912
island_1965	Genomic island β	1965117	2002077	island			1965117	2002077
	ICE6013	1965117	2002077	transposon				
phage_2058	*S. aureus* phage type V	2058040	2058262	phage	2058225	2058403	2058040	2058403
unclassified_2084					2084673	2084719	2084673	2084719
unclassified_2093					2093046	2093069	2093046	2093069
transposon_2108	Tn552	2108728	2108997	transposon	2109042	2109145	2108728	2109145
phage_2119	*S. aureus* phage type II	2119729	2173763	phage	2124543	2173797	2119729	2177018
	S. aureus phage type III	2119729	2173763	phage	2175232	2176944		
	SAPI	2175069	2177018	SAPI	2176976	2177006		
	SAPI	2175069	2177018	SAPI				
transposon_2237	ICE6013	2237729	2239145	transposon	2238750	2238872	2237729	2239145
transposon_2250	Cadmium resistance cassette	2250860	2250946	transposon			2250860	2250946
transposon_2279	ICE6013	2279981	2280445	transposon	2280287	2280358	2279981	2280445
unclassified_2303					2303710	2307729	2303710	2307729
plasmid_2315	Plasmid	2315842	2327306	plasmid			2315842	2327306
	Tn552	2315842	2327306	transposon				
transposon_2358	ICE6013	2358327	2358502	transposon	2358273	2358461	2358273	2358502
unclassified_2375					2375646	2380245	2375646	2380245
unclassified_2384					2384454	2387256	2384454	2387256
unclassified_2519					2519657	2522494	2519657	2522494
transposon_2533	ICE6013	2533595	2535093	transposon	2534898	2534943	2533595	2535093
unclassified_2627					2627431	2627625	2627431	2627625
unclassified_2645					2645376	2648309	2645376	2663463
					2650383	2656927		

Element Name	BLAST hits to known mobile elements				Variable core BiP distance		Manual Curation	
	Description	Start	End	Feature	Start	End	Start	End
					2659745	2659833		
					2661042	2663463		
phage_2675	ICE6013	2675236	2679256	transposon	2675223	2679059	2675223	2679256
	S. aureus phage type II	2675236	2679256	phage				
unclassified_2709					2709025	2709074	2709025	2709074
phage_2747	*S. aureus* phage type II	2747259	2749362	phage	2749205	2749384	2747259	2749384
unclassified_2761					2761235	2761407	2761235	2761407
plasmid_2771	Plasmid	2771563	2771754	plasmid	2771649	2771711	2771563	2771754
	Tn552	2771563	2771754	transposon				
unclassified_2781					2781276	2781440	2781276	2781440
unclassified_2839					2839341	2839745	2839341	2839745
unclassified_2844					2844486	2845676	2844486	2845676
unclassified_2867					2867558	2872272	2867558	2872272
unclassified_2876					2876007	2876103	2876007	2880657
					2876571	2880657		
unclassified_2890					2890485	2894115	2890485	2894115

Supplementary References

1. Pérez-Losada, M., *et al.* Population genetics of microbial pathogens estimated from multilocus sequence typing (MLST) data. *Infect. Genet. Evol.* **6**, 97-112 (2006).
2. Zhang, L., Thomas, J.C., Didelot, X. & Robinson, D.A. Molecular signatures identify a candidate target of balancing selection in an *arcD*-like gene of *Staphylococcus epidermidis*. *J. Mol. Evol.* **75**, 43-54 (2012).
3. Miller, R.R., *et al.* Healthcare-associated outbreak of meticillin-resistant *Staphylococcus aureus* bacteraemia: role of a cryptic variant of an epidemic clone. *J. Hosp. Infect.* **86**, 83-89 (2014).
4. Robinson, D.A. & Enright, M.C. Evolution of *Staphylococcus aureus* by large chromosomal replacements. *J. Bacteriol.* **186**, 1060-1064 (2004).
5. Castillo-Ramírez, S., *et al*. Phylogeographic variation in recombination rates within a global clone of methicillin-resistant *Staphylococcus aureus*. *Genome Biol.* **13**, R126 (2012).
6. Vos, M. & Didelot, X. A comparison of homologous recombination rates in bacteria and archaea. *ISME J.* **3**, 199-208. (2009).
7. Feil, E.J. *et al.* How clonal is *Staphylococcus aureus*? *J. Bacteriol.* **185**, 3307-3316 (2003).
