# Supplementary figures and images for: Mobile elements drive recombination hotspots in the core genome of Staphylococcus aureus (part 2 of 2)
Source: Nat Commun. 2014 May 23;5:3956. doi: 10.1038/ncomms4956 (PMC4036114; doi:10.1038/ncomms4956)

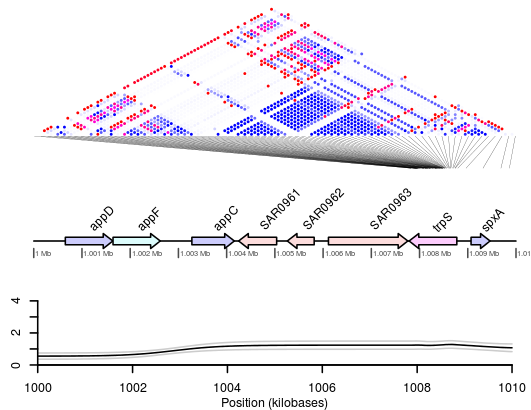

Supplement: Supplementary Data 1 — Homoplasy and linkage disequilibrium in the Staphylococcus aureus core genome. Whole-genome LD plots are illustrated in 10kb windows. Each 10kb window is displayed as in Figure 3, with a single reference genome, MRSA252. Genes are color-coded by COG category or grey if unclassified. An extended coldspot can be seen between 1448-1458kb. [file ncomms4956-s2.zip › EverittSupplementaryDataset1/1000-1010.LD.png]

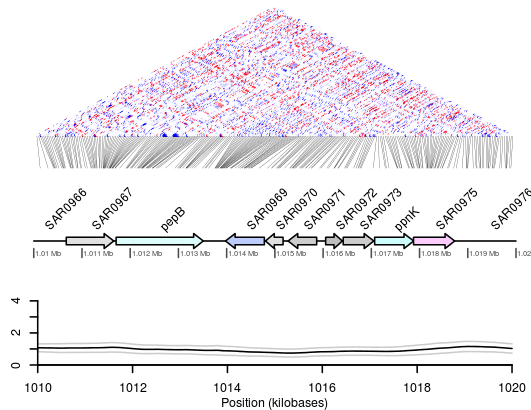

Supplement: Supplementary Data 1 — Homoplasy and linkage disequilibrium in the Staphylococcus aureus core genome. Whole-genome LD plots are illustrated in 10kb windows. Each 10kb window is displayed as in Figure 3, with a single reference genome, MRSA252. Genes are color-coded by COG category or grey if unclassified. An extended coldspot can be seen between 1448-1458kb. [file ncomms4956-s2.zip › EverittSupplementaryDataset1/1010-1020.LD.png]

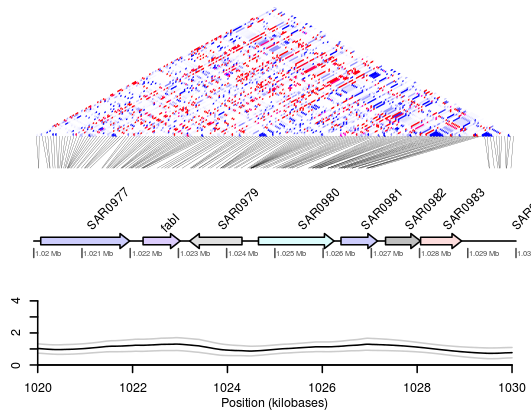

Supplement: Supplementary Data 1 — Homoplasy and linkage disequilibrium in the Staphylococcus aureus core genome. Whole-genome LD plots are illustrated in 10kb windows. Each 10kb window is displayed as in Figure 3, with a single reference genome, MRSA252. Genes are color-coded by COG category or grey if unclassified. An extended coldspot can be seen between 1448-1458kb. [file ncomms4956-s2.zip › EverittSupplementaryDataset1/1020-1030.LD.png]

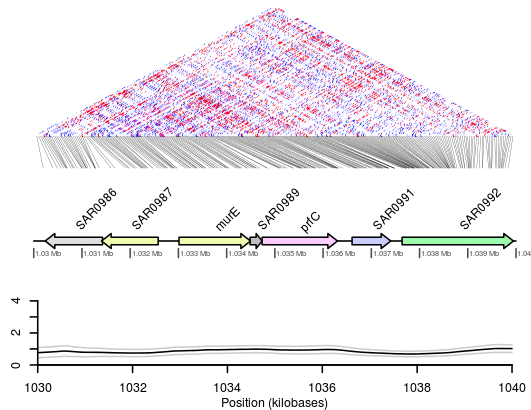

Supplement: Supplementary Data 1 — Homoplasy and linkage disequilibrium in the Staphylococcus aureus core genome. Whole-genome LD plots are illustrated in 10kb windows. Each 10kb window is displayed as in Figure 3, with a single reference genome, MRSA252. Genes are color-coded by COG category or grey if unclassified. An extended coldspot can be seen between 1448-1458kb. [file ncomms4956-s2.zip › EverittSupplementaryDataset1/1030-1040.LD.png]

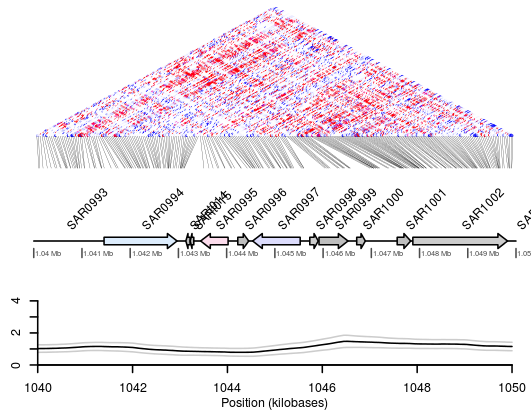

Supplement: Supplementary Data 1 — Homoplasy and linkage disequilibrium in the Staphylococcus aureus core genome. Whole-genome LD plots are illustrated in 10kb windows. Each 10kb window is displayed as in Figure 3, with a single reference genome, MRSA252. Genes are color-coded by COG category or grey if unclassified. An extended coldspot can be seen between 1448-1458kb. [file ncomms4956-s2.zip › EverittSupplementaryDataset1/1040-1050.LD.png]

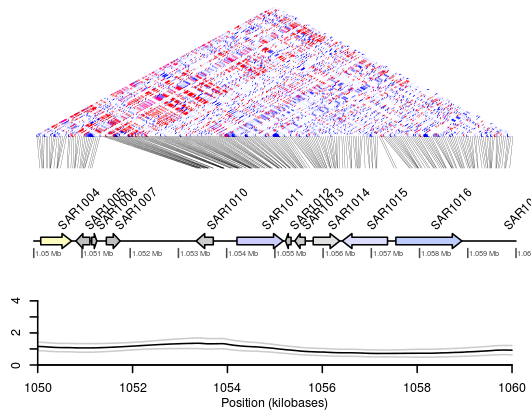

Supplement: Supplementary Data 1 — Homoplasy and linkage disequilibrium in the Staphylococcus aureus core genome. Whole-genome LD plots are illustrated in 10kb windows. Each 10kb window is displayed as in Figure 3, with a single reference genome, MRSA252. Genes are color-coded by COG category or grey if unclassified. An extended coldspot can be seen between 1448-1458kb. [file ncomms4956-s2.zip › EverittSupplementaryDataset1/1050-1060.LD.png]

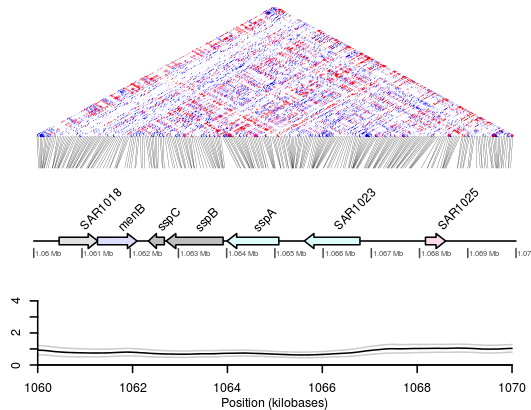

Supplement: Supplementary Data 1 — Homoplasy and linkage disequilibrium in the Staphylococcus aureus core genome. Whole-genome LD plots are illustrated in 10kb windows. Each 10kb window is displayed as in Figure 3, with a single reference genome, MRSA252. Genes are color-coded by COG category or grey if unclassified. An extended coldspot can be seen between 1448-1458kb. [file ncomms4956-s2.zip › EverittSupplementaryDataset1/1060-1070.LD.png]

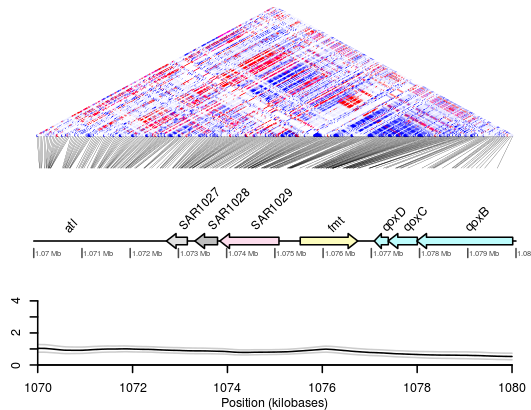

Supplement: Supplementary Data 1 — Homoplasy and linkage disequilibrium in the Staphylococcus aureus core genome. Whole-genome LD plots are illustrated in 10kb windows. Each 10kb window is displayed as in Figure 3, with a single reference genome, MRSA252. Genes are color-coded by COG category or grey if unclassified. An extended coldspot can be seen between 1448-1458kb. [file ncomms4956-s2.zip › EverittSupplementaryDataset1/1070-1080.LD.png]

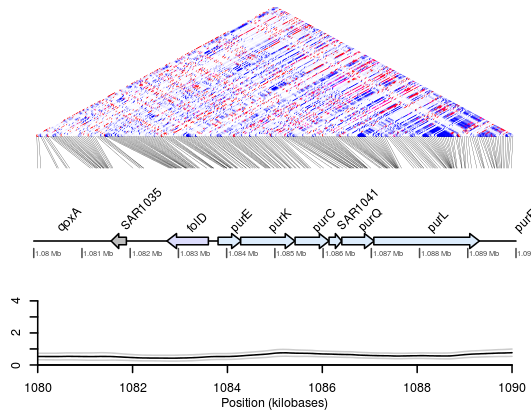

Supplement: Supplementary Data 1 — Homoplasy and linkage disequilibrium in the Staphylococcus aureus core genome. Whole-genome LD plots are illustrated in 10kb windows. Each 10kb window is displayed as in Figure 3, with a single reference genome, MRSA252. Genes are color-coded by COG category or grey if unclassified. An extended coldspot can be seen between 1448-1458kb. [file ncomms4956-s2.zip › EverittSupplementaryDataset1/1080-1090.LD.png]

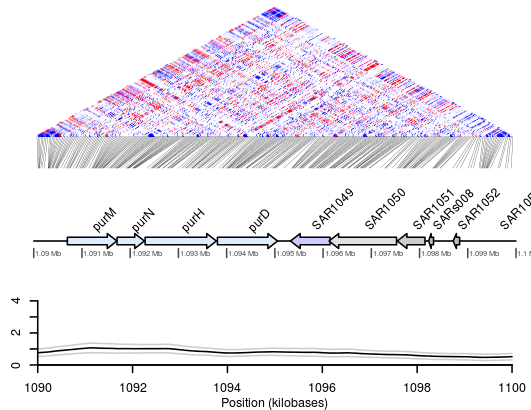

Supplement: Supplementary Data 1 — Homoplasy and linkage disequilibrium in the Staphylococcus aureus core genome. Whole-genome LD plots are illustrated in 10kb windows. Each 10kb window is displayed as in Figure 3, with a single reference genome, MRSA252. Genes are color-coded by COG category or grey if unclassified. An extended coldspot can be seen between 1448-1458kb. [file ncomms4956-s2.zip › EverittSupplementaryDataset1/1090-1100.LD.png]

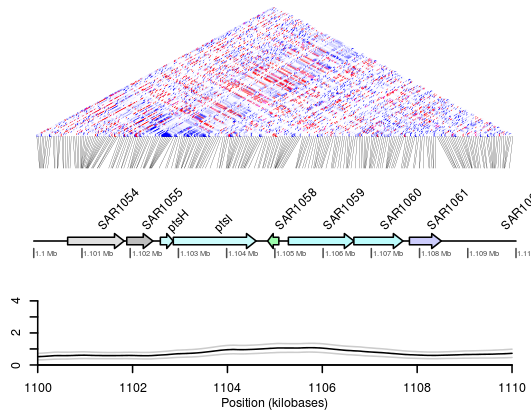

Supplement: Supplementary Data 1 — Homoplasy and linkage disequilibrium in the Staphylococcus aureus core genome. Whole-genome LD plots are illustrated in 10kb windows. Each 10kb window is displayed as in Figure 3, with a single reference genome, MRSA252. Genes are color-coded by COG category or grey if unclassified. An extended coldspot can be seen between 1448-1458kb. [file ncomms4956-s2.zip › EverittSupplementaryDataset1/1100-1110.LD.png]

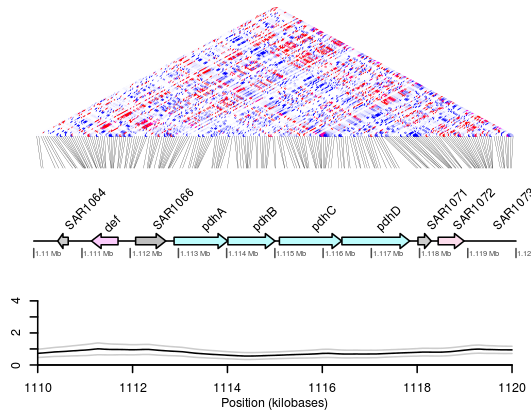

Supplement: Supplementary Data 1 — Homoplasy and linkage disequilibrium in the Staphylococcus aureus core genome. Whole-genome LD plots are illustrated in 10kb windows. Each 10kb window is displayed as in Figure 3, with a single reference genome, MRSA252. Genes are color-coded by COG category or grey if unclassified. An extended coldspot can be seen between 1448-1458kb. [file ncomms4956-s2.zip › EverittSupplementaryDataset1/1110-1120.LD.png]

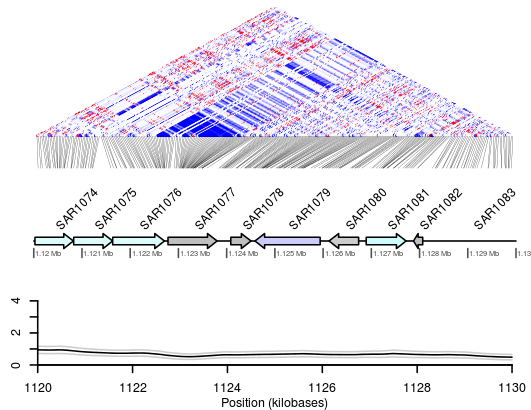

Supplement: Supplementary Data 1 — Homoplasy and linkage disequilibrium in the Staphylococcus aureus core genome. Whole-genome LD plots are illustrated in 10kb windows. Each 10kb window is displayed as in Figure 3, with a single reference genome, MRSA252. Genes are color-coded by COG category or grey if unclassified. An extended coldspot can be seen between 1448-1458kb. [file ncomms4956-s2.zip › EverittSupplementaryDataset1/1120-1130.LD.png]

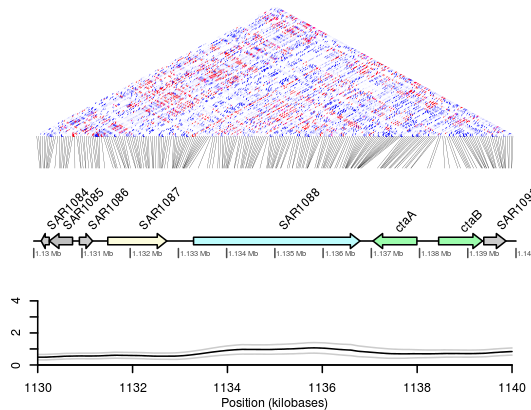

Supplement: Supplementary Data 1 — Homoplasy and linkage disequilibrium in the Staphylococcus aureus core genome. Whole-genome LD plots are illustrated in 10kb windows. Each 10kb window is displayed as in Figure 3, with a single reference genome, MRSA252. Genes are color-coded by COG category or grey if unclassified. An extended coldspot can be seen between 1448-1458kb. [file ncomms4956-s2.zip › EverittSupplementaryDataset1/1130-1140.LD.png]

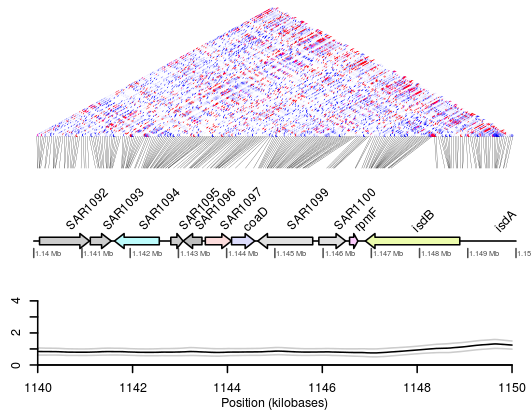

Supplement: Supplementary Data 1 — Homoplasy and linkage disequilibrium in the Staphylococcus aureus core genome. Whole-genome LD plots are illustrated in 10kb windows. Each 10kb window is displayed as in Figure 3, with a single reference genome, MRSA252. Genes are color-coded by COG category or grey if unclassified. An extended coldspot can be seen between 1448-1458kb. [file ncomms4956-s2.zip › EverittSupplementaryDataset1/1140-1150.LD.png]

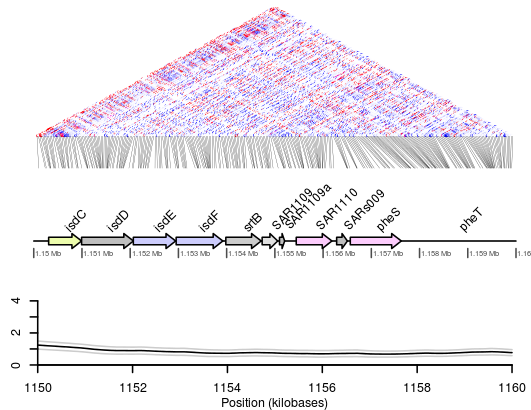

Supplement: Supplementary Data 1 — Homoplasy and linkage disequilibrium in the Staphylococcus aureus core genome. Whole-genome LD plots are illustrated in 10kb windows. Each 10kb window is displayed as in Figure 3, with a single reference genome, MRSA252. Genes are color-coded by COG category or grey if unclassified. An extended coldspot can be seen between 1448-1458kb. [file ncomms4956-s2.zip › EverittSupplementaryDataset1/1150-1160.LD.png]

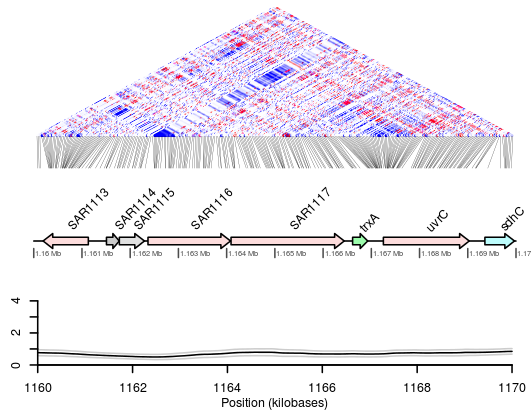

Supplement: Supplementary Data 1 — Homoplasy and linkage disequilibrium in the Staphylococcus aureus core genome. Whole-genome LD plots are illustrated in 10kb windows. Each 10kb window is displayed as in Figure 3, with a single reference genome, MRSA252. Genes are color-coded by COG category or grey if unclassified. An extended coldspot can be seen between 1448-1458kb. [file ncomms4956-s2.zip › EverittSupplementaryDataset1/1160-1170.LD.png]

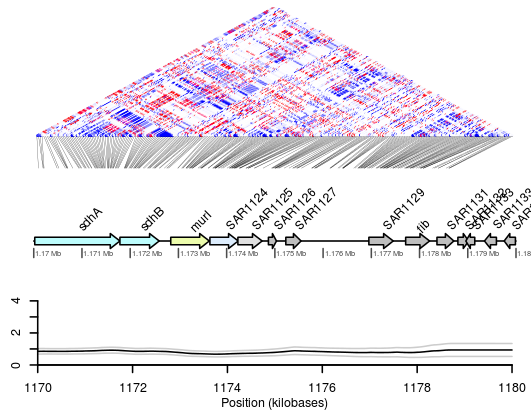

Supplement: Supplementary Data 1 — Homoplasy and linkage disequilibrium in the Staphylococcus aureus core genome. Whole-genome LD plots are illustrated in 10kb windows. Each 10kb window is displayed as in Figure 3, with a single reference genome, MRSA252. Genes are color-coded by COG category or grey if unclassified. An extended coldspot can be seen between 1448-1458kb. [file ncomms4956-s2.zip › EverittSupplementaryDataset1/1170-1180.LD.png]

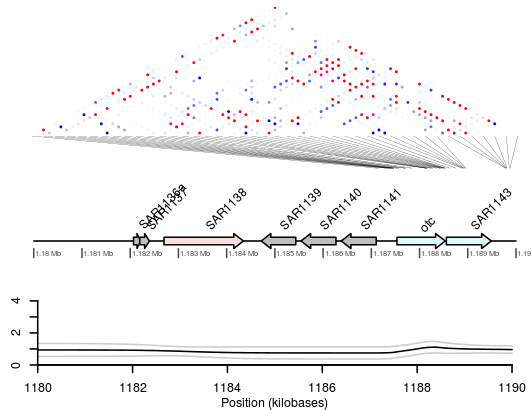

Supplement: Supplementary Data 1 — Homoplasy and linkage disequilibrium in the Staphylococcus aureus core genome. Whole-genome LD plots are illustrated in 10kb windows. Each 10kb window is displayed as in Figure 3, with a single reference genome, MRSA252. Genes are color-coded by COG category or grey if unclassified. An extended coldspot can be seen between 1448-1458kb. [file ncomms4956-s2.zip › EverittSupplementaryDataset1/1180-1190.LD.png]

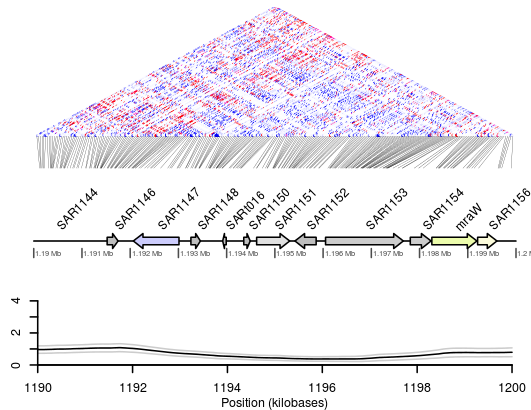

Supplement: Supplementary Data 1 — Homoplasy and linkage disequilibrium in the Staphylococcus aureus core genome. Whole-genome LD plots are illustrated in 10kb windows. Each 10kb window is displayed as in Figure 3, with a single reference genome, MRSA252. Genes are color-coded by COG category or grey if unclassified. An extended coldspot can be seen between 1448-1458kb. [file ncomms4956-s2.zip › EverittSupplementaryDataset1/1190-1200.LD.png]

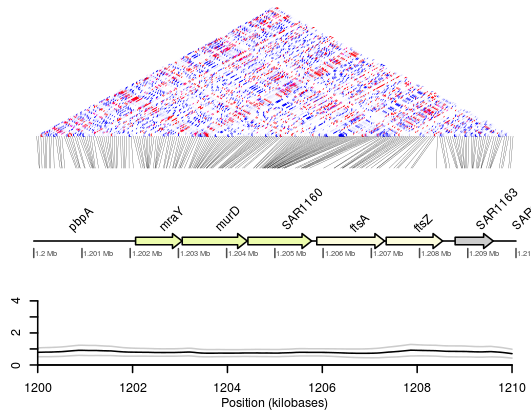

Supplement: Supplementary Data 1 — Homoplasy and linkage disequilibrium in the Staphylococcus aureus core genome. Whole-genome LD plots are illustrated in 10kb windows. Each 10kb window is displayed as in Figure 3, with a single reference genome, MRSA252. Genes are color-coded by COG category or grey if unclassified. An extended coldspot can be seen between 1448-1458kb. [file ncomms4956-s2.zip › EverittSupplementaryDataset1/1200-1210.LD.png]

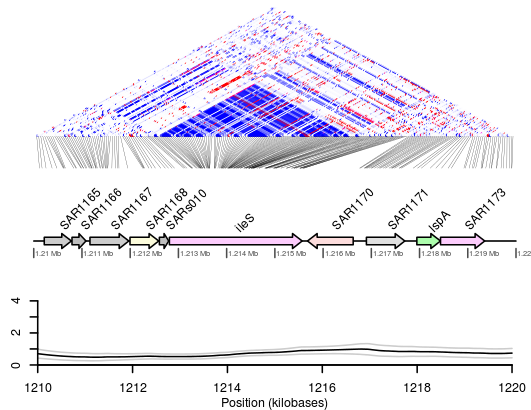

Supplement: Supplementary Data 1 — Homoplasy and linkage disequilibrium in the Staphylococcus aureus core genome. Whole-genome LD plots are illustrated in 10kb windows. Each 10kb window is displayed as in Figure 3, with a single reference genome, MRSA252. Genes are color-coded by COG category or grey if unclassified. An extended coldspot can be seen between 1448-1458kb. [file ncomms4956-s2.zip › EverittSupplementaryDataset1/1210-1220.LD.png]

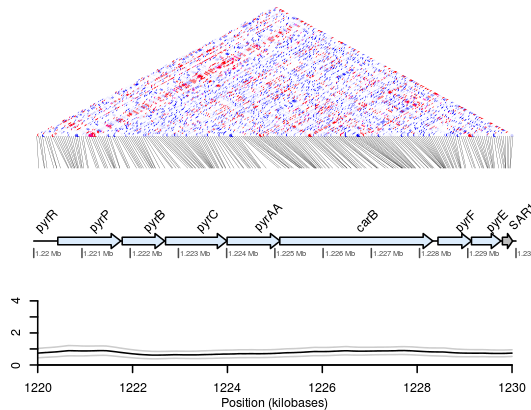

Supplement: Supplementary Data 1 — Homoplasy and linkage disequilibrium in the Staphylococcus aureus core genome. Whole-genome LD plots are illustrated in 10kb windows. Each 10kb window is displayed as in Figure 3, with a single reference genome, MRSA252. Genes are color-coded by COG category or grey if unclassified. An extended coldspot can be seen between 1448-1458kb. [file ncomms4956-s2.zip › EverittSupplementaryDataset1/1220-1230.LD.png]

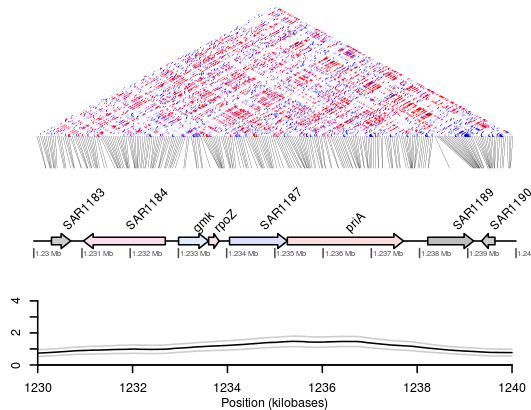

Supplement: Supplementary Data 1 — Homoplasy and linkage disequilibrium in the Staphylococcus aureus core genome. Whole-genome LD plots are illustrated in 10kb windows. Each 10kb window is displayed as in Figure 3, with a single reference genome, MRSA252. Genes are color-coded by COG category or grey if unclassified. An extended coldspot can be seen between 1448-1458kb. [file ncomms4956-s2.zip › EverittSupplementaryDataset1/1230-1240.LD.png]

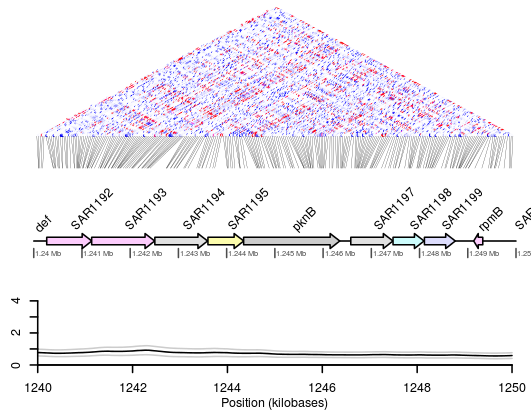

Supplement: Supplementary Data 1 — Homoplasy and linkage disequilibrium in the Staphylococcus aureus core genome. Whole-genome LD plots are illustrated in 10kb windows. Each 10kb window is displayed as in Figure 3, with a single reference genome, MRSA252. Genes are color-coded by COG category or grey if unclassified. An extended coldspot can be seen between 1448-1458kb. [file ncomms4956-s2.zip › EverittSupplementaryDataset1/1240-1250.LD.png]

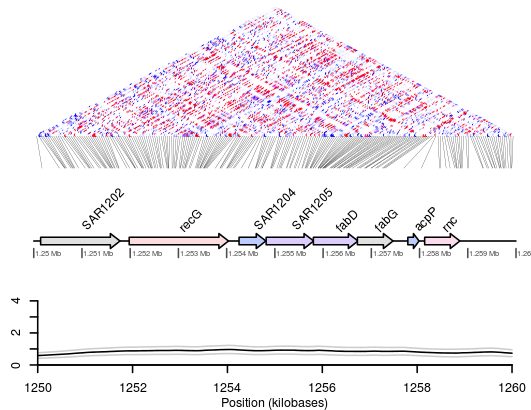

Supplement: Supplementary Data 1 — Homoplasy and linkage disequilibrium in the Staphylococcus aureus core genome. Whole-genome LD plots are illustrated in 10kb windows. Each 10kb window is displayed as in Figure 3, with a single reference genome, MRSA252. Genes are color-coded by COG category or grey if unclassified. An extended coldspot can be seen between 1448-1458kb. [file ncomms4956-s2.zip › EverittSupplementaryDataset1/1250-1260.LD.png]

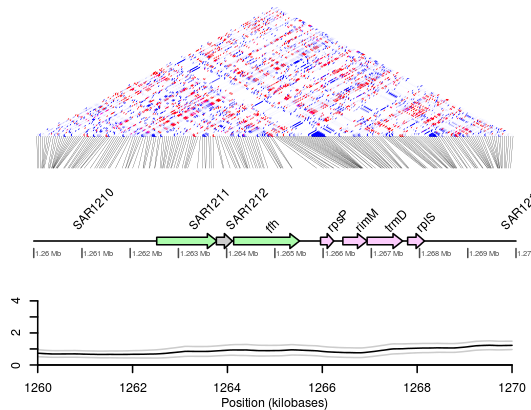

Supplement: Supplementary Data 1 — Homoplasy and linkage disequilibrium in the Staphylococcus aureus core genome. Whole-genome LD plots are illustrated in 10kb windows. Each 10kb window is displayed as in Figure 3, with a single reference genome, MRSA252. Genes are color-coded by COG category or grey if unclassified. An extended coldspot can be seen between 1448-1458kb. [file ncomms4956-s2.zip › EverittSupplementaryDataset1/1260-1270.LD.png]

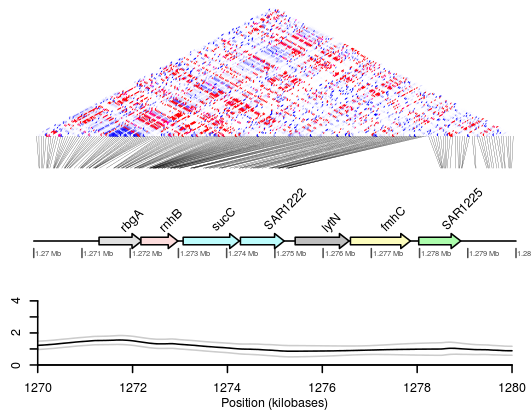

Supplement: Supplementary Data 1 — Homoplasy and linkage disequilibrium in the Staphylococcus aureus core genome. Whole-genome LD plots are illustrated in 10kb windows. Each 10kb window is displayed as in Figure 3, with a single reference genome, MRSA252. Genes are color-coded by COG category or grey if unclassified. An extended coldspot can be seen between 1448-1458kb. [file ncomms4956-s2.zip › EverittSupplementaryDataset1/1270-1280.LD.png]

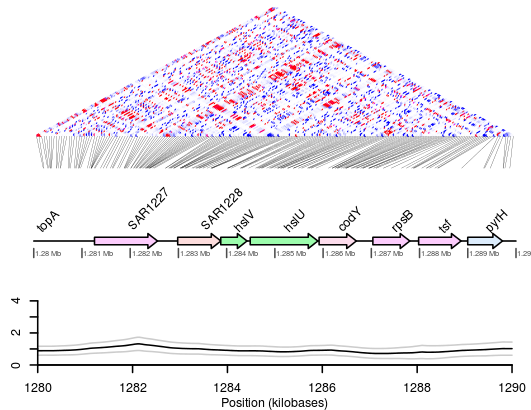

Supplement: Supplementary Data 1 — Homoplasy and linkage disequilibrium in the Staphylococcus aureus core genome. Whole-genome LD plots are illustrated in 10kb windows. Each 10kb window is displayed as in Figure 3, with a single reference genome, MRSA252. Genes are color-coded by COG category or grey if unclassified. An extended coldspot can be seen between 1448-1458kb. [file ncomms4956-s2.zip › EverittSupplementaryDataset1/1280-1290.LD.png]

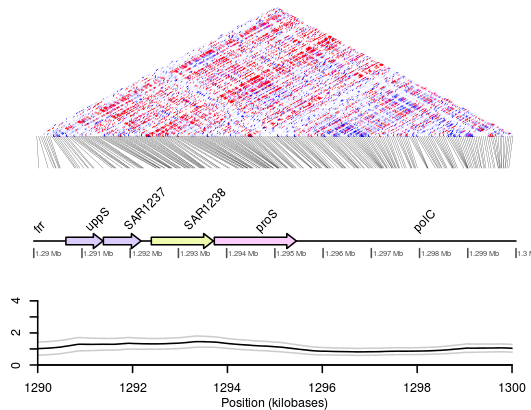

Supplement: Supplementary Data 1 — Homoplasy and linkage disequilibrium in the Staphylococcus aureus core genome. Whole-genome LD plots are illustrated in 10kb windows. Each 10kb window is displayed as in Figure 3, with a single reference genome, MRSA252. Genes are color-coded by COG category or grey if unclassified. An extended coldspot can be seen between 1448-1458kb. [file ncomms4956-s2.zip › EverittSupplementaryDataset1/1290-1300.LD.png]

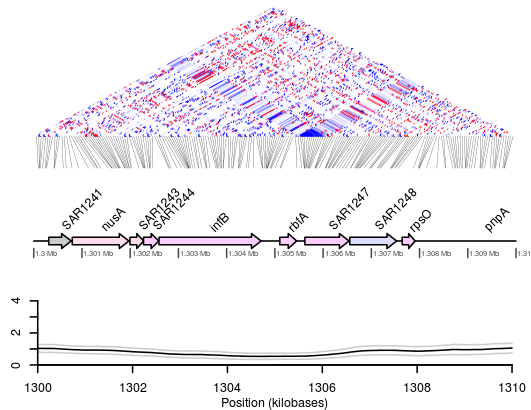

Supplement: Supplementary Data 1 — Homoplasy and linkage disequilibrium in the Staphylococcus aureus core genome. Whole-genome LD plots are illustrated in 10kb windows. Each 10kb window is displayed as in Figure 3, with a single reference genome, MRSA252. Genes are color-coded by COG category or grey if unclassified. An extended coldspot can be seen between 1448-1458kb. [file ncomms4956-s2.zip › EverittSupplementaryDataset1/1300-1310.LD.png]

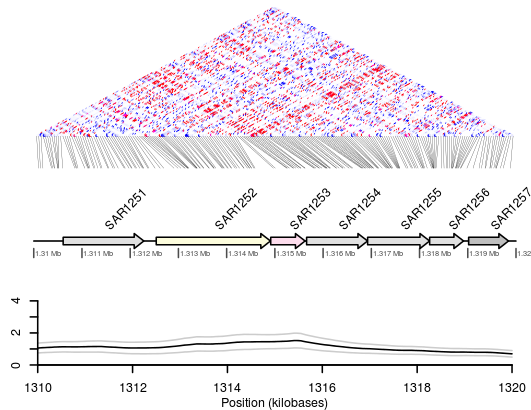

Supplement: Supplementary Data 1 — Homoplasy and linkage disequilibrium in the Staphylococcus aureus core genome. Whole-genome LD plots are illustrated in 10kb windows. Each 10kb window is displayed as in Figure 3, with a single reference genome, MRSA252. Genes are color-coded by COG category or grey if unclassified. An extended coldspot can be seen between 1448-1458kb. [file ncomms4956-s2.zip › EverittSupplementaryDataset1/1310-1320.LD.png]

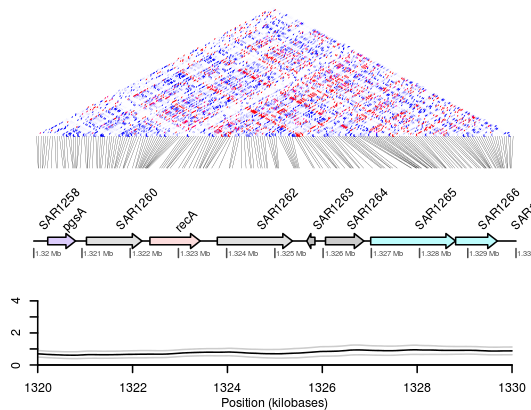

Supplement: Supplementary Data 1 — Homoplasy and linkage disequilibrium in the Staphylococcus aureus core genome. Whole-genome LD plots are illustrated in 10kb windows. Each 10kb window is displayed as in Figure 3, with a single reference genome, MRSA252. Genes are color-coded by COG category or grey if unclassified. An extended coldspot can be seen between 1448-1458kb. [file ncomms4956-s2.zip › EverittSupplementaryDataset1/1320-1330.LD.png]

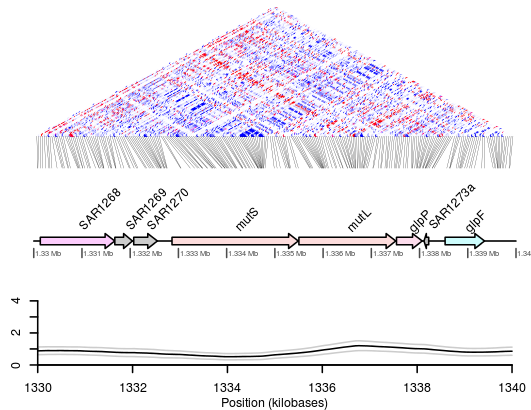

Supplement: Supplementary Data 1 — Homoplasy and linkage disequilibrium in the Staphylococcus aureus core genome. Whole-genome LD plots are illustrated in 10kb windows. Each 10kb window is displayed as in Figure 3, with a single reference genome, MRSA252. Genes are color-coded by COG category or grey if unclassified. An extended coldspot can be seen between 1448-1458kb. [file ncomms4956-s2.zip › EverittSupplementaryDataset1/1330-1340.LD.png]

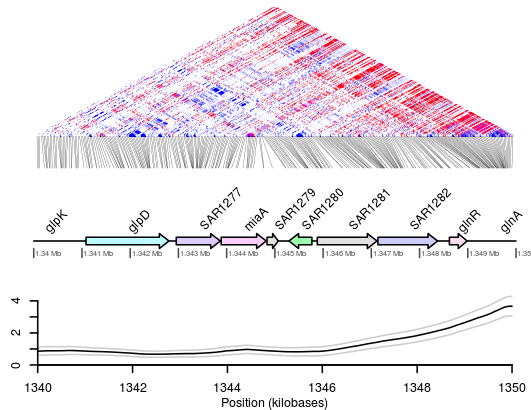

Supplement: Supplementary Data 1 — Homoplasy and linkage disequilibrium in the Staphylococcus aureus core genome. Whole-genome LD plots are illustrated in 10kb windows. Each 10kb window is displayed as in Figure 3, with a single reference genome, MRSA252. Genes are color-coded by COG category or grey if unclassified. An extended coldspot can be seen between 1448-1458kb. [file ncomms4956-s2.zip › EverittSupplementaryDataset1/1340-1350.LD.png]

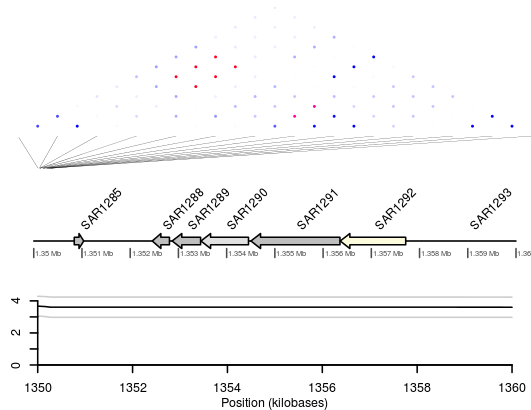

Supplement: Supplementary Data 1 — Homoplasy and linkage disequilibrium in the Staphylococcus aureus core genome. Whole-genome LD plots are illustrated in 10kb windows. Each 10kb window is displayed as in Figure 3, with a single reference genome, MRSA252. Genes are color-coded by COG category or grey if unclassified. An extended coldspot can be seen between 1448-1458kb. [file ncomms4956-s2.zip › EverittSupplementaryDataset1/1350-1360.LD.png]

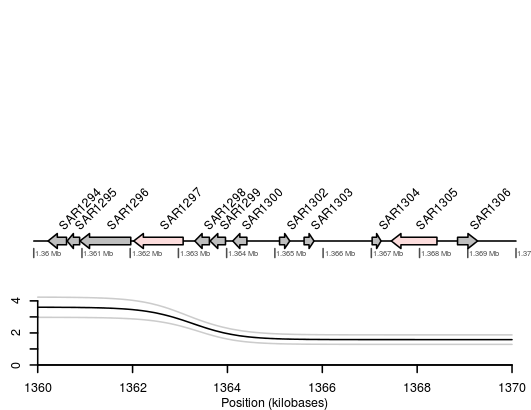

Supplement: Supplementary Data 1 — Homoplasy and linkage disequilibrium in the Staphylococcus aureus core genome. Whole-genome LD plots are illustrated in 10kb windows. Each 10kb window is displayed as in Figure 3, with a single reference genome, MRSA252. Genes are color-coded by COG category or grey if unclassified. An extended coldspot can be seen between 1448-1458kb. [file ncomms4956-s2.zip › EverittSupplementaryDataset1/1360-1370.LD.png]

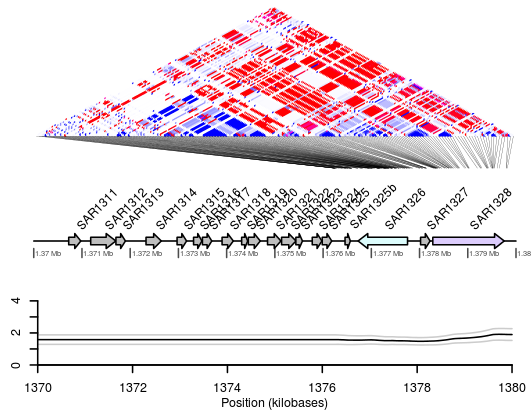

Supplement: Supplementary Data 1 — Homoplasy and linkage disequilibrium in the Staphylococcus aureus core genome. Whole-genome LD plots are illustrated in 10kb windows. Each 10kb window is displayed as in Figure 3, with a single reference genome, MRSA252. Genes are color-coded by COG category or grey if unclassified. An extended coldspot can be seen between 1448-1458kb. [file ncomms4956-s2.zip › EverittSupplementaryDataset1/1370-1380.LD.png]

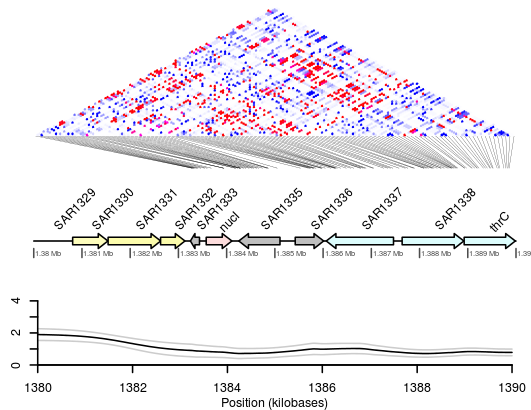

Supplement: Supplementary Data 1 — Homoplasy and linkage disequilibrium in the Staphylococcus aureus core genome. Whole-genome LD plots are illustrated in 10kb windows. Each 10kb window is displayed as in Figure 3, with a single reference genome, MRSA252. Genes are color-coded by COG category or grey if unclassified. An extended coldspot can be seen between 1448-1458kb. [file ncomms4956-s2.zip › EverittSupplementaryDataset1/1380-1390.LD.png]

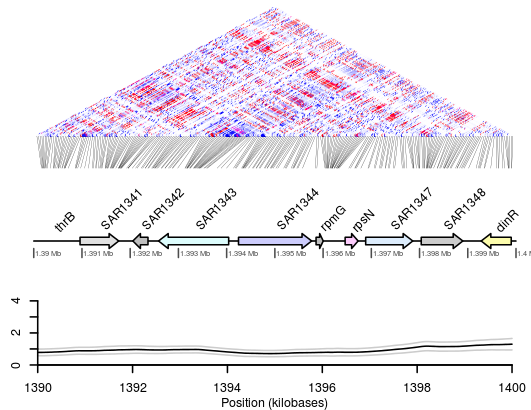

Supplement: Supplementary Data 1 — Homoplasy and linkage disequilibrium in the Staphylococcus aureus core genome. Whole-genome LD plots are illustrated in 10kb windows. Each 10kb window is displayed as in Figure 3, with a single reference genome, MRSA252. Genes are color-coded by COG category or grey if unclassified. An extended coldspot can be seen between 1448-1458kb. [file ncomms4956-s2.zip › EverittSupplementaryDataset1/1390-1400.LD.png]

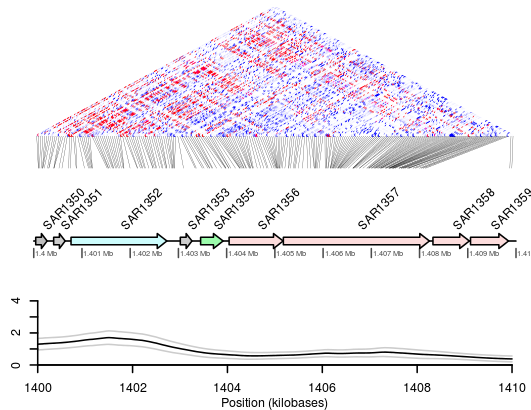

Supplement: Supplementary Data 1 — Homoplasy and linkage disequilibrium in the Staphylococcus aureus core genome. Whole-genome LD plots are illustrated in 10kb windows. Each 10kb window is displayed as in Figure 3, with a single reference genome, MRSA252. Genes are color-coded by COG category or grey if unclassified. An extended coldspot can be seen between 1448-1458kb. [file ncomms4956-s2.zip › EverittSupplementaryDataset1/1400-1410.LD.png]

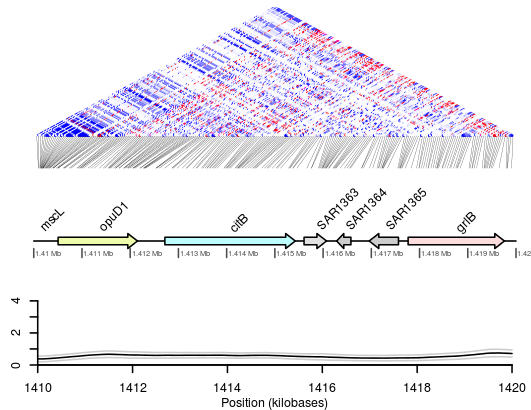

Supplement: Supplementary Data 1 — Homoplasy and linkage disequilibrium in the Staphylococcus aureus core genome. Whole-genome LD plots are illustrated in 10kb windows. Each 10kb window is displayed as in Figure 3, with a single reference genome, MRSA252. Genes are color-coded by COG category or grey if unclassified. An extended coldspot can be seen between 1448-1458kb. [file ncomms4956-s2.zip › EverittSupplementaryDataset1/1410-1420.LD.png]

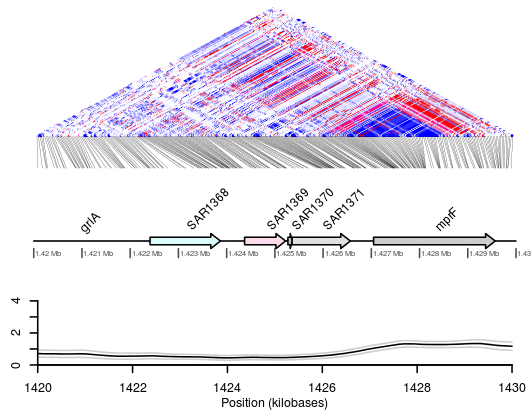

Supplement: Supplementary Data 1 — Homoplasy and linkage disequilibrium in the Staphylococcus aureus core genome. Whole-genome LD plots are illustrated in 10kb windows. Each 10kb window is displayed as in Figure 3, with a single reference genome, MRSA252. Genes are color-coded by COG category or grey if unclassified. An extended coldspot can be seen between 1448-1458kb. [file ncomms4956-s2.zip › EverittSupplementaryDataset1/1420-1430.LD.png]

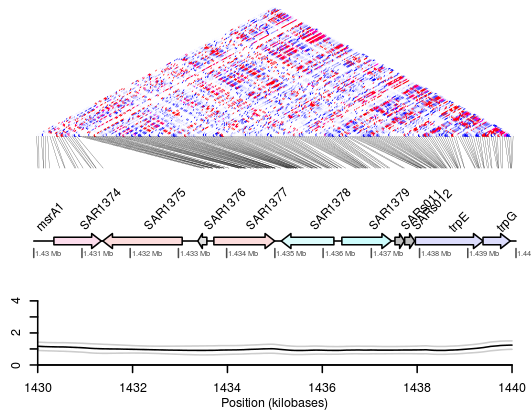

Supplement: Supplementary Data 1 — Homoplasy and linkage disequilibrium in the Staphylococcus aureus core genome. Whole-genome LD plots are illustrated in 10kb windows. Each 10kb window is displayed as in Figure 3, with a single reference genome, MRSA252. Genes are color-coded by COG category or grey if unclassified. An extended coldspot can be seen between 1448-1458kb. [file ncomms4956-s2.zip › EverittSupplementaryDataset1/1430-1440.LD.png]

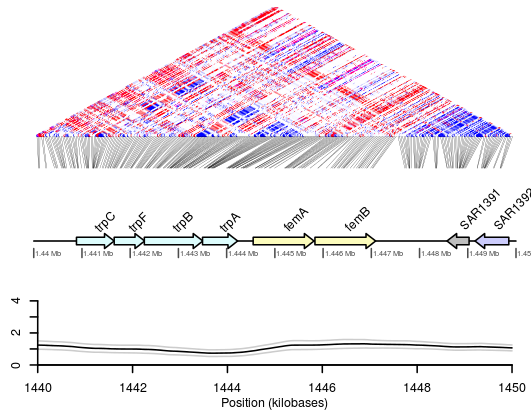

Supplement: Supplementary Data 1 — Homoplasy and linkage disequilibrium in the Staphylococcus aureus core genome. Whole-genome LD plots are illustrated in 10kb windows. Each 10kb window is displayed as in Figure 3, with a single reference genome, MRSA252. Genes are color-coded by COG category or grey if unclassified. An extended coldspot can be seen between 1448-1458kb. [file ncomms4956-s2.zip › EverittSupplementaryDataset1/1440-1450.LD.png]

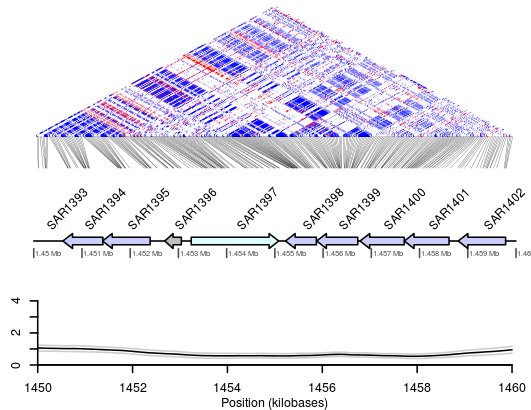

Supplement: Supplementary Data 1 — Homoplasy and linkage disequilibrium in the Staphylococcus aureus core genome. Whole-genome LD plots are illustrated in 10kb windows. Each 10kb window is displayed as in Figure 3, with a single reference genome, MRSA252. Genes are color-coded by COG category or grey if unclassified. An extended coldspot can be seen between 1448-1458kb. [file ncomms4956-s2.zip › EverittSupplementaryDataset1/1450-1460.LD.png]

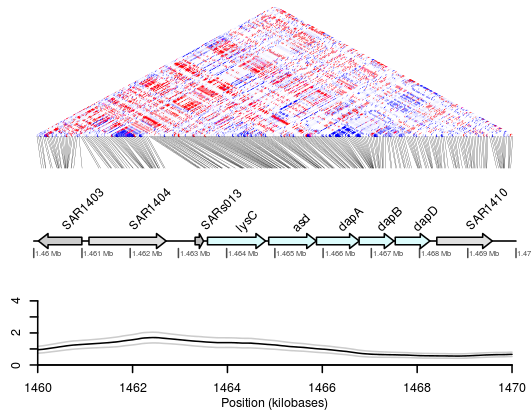

Supplement: Supplementary Data 1 — Homoplasy and linkage disequilibrium in the Staphylococcus aureus core genome. Whole-genome LD plots are illustrated in 10kb windows. Each 10kb window is displayed as in Figure 3, with a single reference genome, MRSA252. Genes are color-coded by COG category or grey if unclassified. An extended coldspot can be seen between 1448-1458kb. [file ncomms4956-s2.zip › EverittSupplementaryDataset1/1460-1470.LD.png]

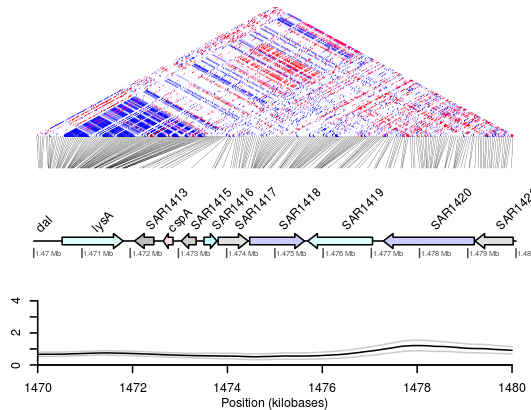

Supplement: Supplementary Data 1 — Homoplasy and linkage disequilibrium in the Staphylococcus aureus core genome. Whole-genome LD plots are illustrated in 10kb windows. Each 10kb window is displayed as in Figure 3, with a single reference genome, MRSA252. Genes are color-coded by COG category or grey if unclassified. An extended coldspot can be seen between 1448-1458kb. [file ncomms4956-s2.zip › EverittSupplementaryDataset1/1470-1480.LD.png]

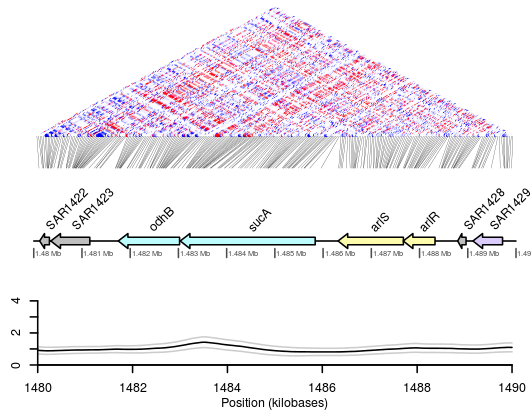

Supplement: Supplementary Data 1 — Homoplasy and linkage disequilibrium in the Staphylococcus aureus core genome. Whole-genome LD plots are illustrated in 10kb windows. Each 10kb window is displayed as in Figure 3, with a single reference genome, MRSA252. Genes are color-coded by COG category or grey if unclassified. An extended coldspot can be seen between 1448-1458kb. [file ncomms4956-s2.zip › EverittSupplementaryDataset1/1480-1490.LD.png]

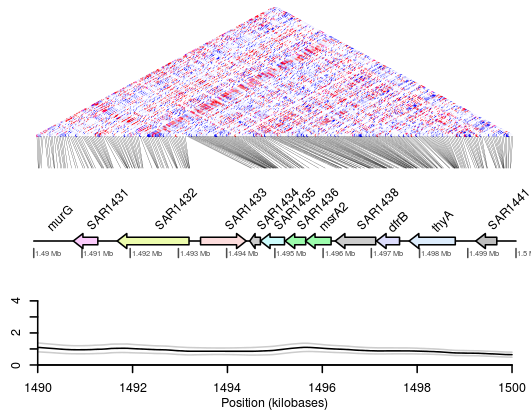

Supplement: Supplementary Data 1 — Homoplasy and linkage disequilibrium in the Staphylococcus aureus core genome. Whole-genome LD plots are illustrated in 10kb windows. Each 10kb window is displayed as in Figure 3, with a single reference genome, MRSA252. Genes are color-coded by COG category or grey if unclassified. An extended coldspot can be seen between 1448-1458kb. [file ncomms4956-s2.zip › EverittSupplementaryDataset1/1490-1500.LD.png]

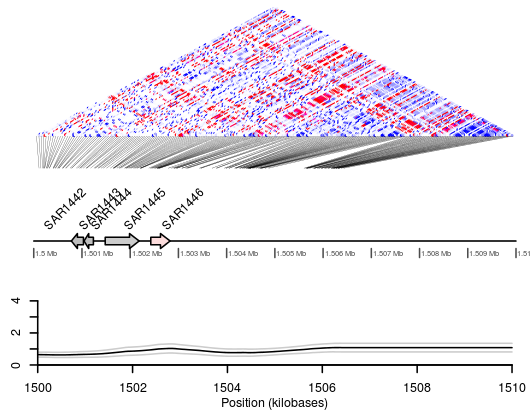

Supplement: Supplementary Data 1 — Homoplasy and linkage disequilibrium in the Staphylococcus aureus core genome. Whole-genome LD plots are illustrated in 10kb windows. Each 10kb window is displayed as in Figure 3, with a single reference genome, MRSA252. Genes are color-coded by COG category or grey if unclassified. An extended coldspot can be seen between 1448-1458kb. [file ncomms4956-s2.zip › EverittSupplementaryDataset1/1500-1510.LD.png]

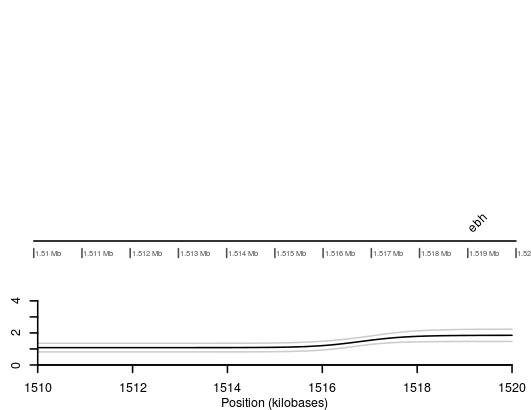

Supplement: Supplementary Data 1 — Homoplasy and linkage disequilibrium in the Staphylococcus aureus core genome. Whole-genome LD plots are illustrated in 10kb windows. Each 10kb window is displayed as in Figure 3, with a single reference genome, MRSA252. Genes are color-coded by COG category or grey if unclassified. An extended coldspot can be seen between 1448-1458kb. [file ncomms4956-s2.zip › EverittSupplementaryDataset1/1510-1520.LD.png]

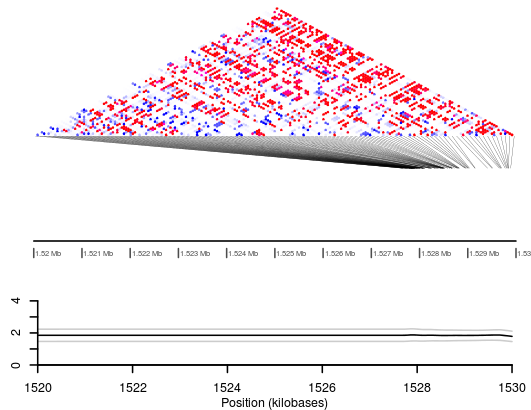

Supplement: Supplementary Data 1 — Homoplasy and linkage disequilibrium in the Staphylococcus aureus core genome. Whole-genome LD plots are illustrated in 10kb windows. Each 10kb window is displayed as in Figure 3, with a single reference genome, MRSA252. Genes are color-coded by COG category or grey if unclassified. An extended coldspot can be seen between 1448-1458kb. [file ncomms4956-s2.zip › EverittSupplementaryDataset1/1520-1530.LD.png]

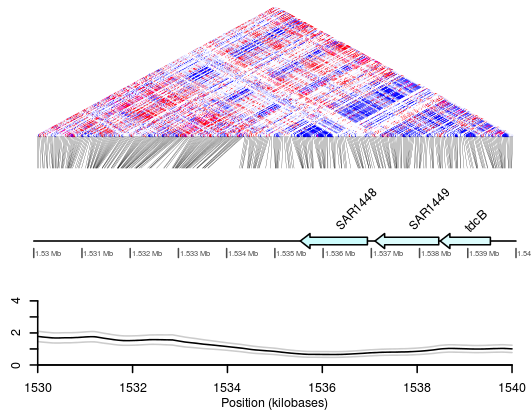

Supplement: Supplementary Data 1 — Homoplasy and linkage disequilibrium in the Staphylococcus aureus core genome. Whole-genome LD plots are illustrated in 10kb windows. Each 10kb window is displayed as in Figure 3, with a single reference genome, MRSA252. Genes are color-coded by COG category or grey if unclassified. An extended coldspot can be seen between 1448-1458kb. [file ncomms4956-s2.zip › EverittSupplementaryDataset1/1530-1540.LD.png]

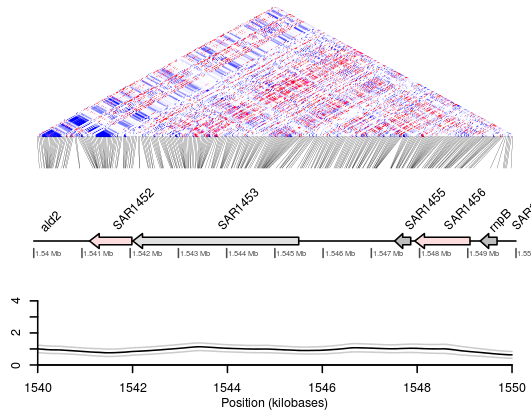

Supplement: Supplementary Data 1 — Homoplasy and linkage disequilibrium in the Staphylococcus aureus core genome. Whole-genome LD plots are illustrated in 10kb windows. Each 10kb window is displayed as in Figure 3, with a single reference genome, MRSA252. Genes are color-coded by COG category or grey if unclassified. An extended coldspot can be seen between 1448-1458kb. [file ncomms4956-s2.zip › EverittSupplementaryDataset1/1540-1550.LD.png]

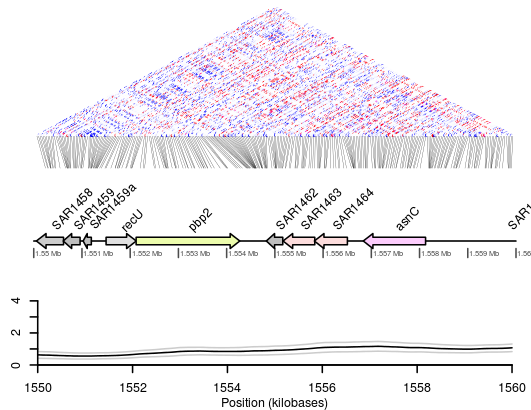

Supplement: Supplementary Data 1 — Homoplasy and linkage disequilibrium in the Staphylococcus aureus core genome. Whole-genome LD plots are illustrated in 10kb windows. Each 10kb window is displayed as in Figure 3, with a single reference genome, MRSA252. Genes are color-coded by COG category or grey if unclassified. An extended coldspot can be seen between 1448-1458kb. [file ncomms4956-s2.zip › EverittSupplementaryDataset1/1550-1560.LD.png]

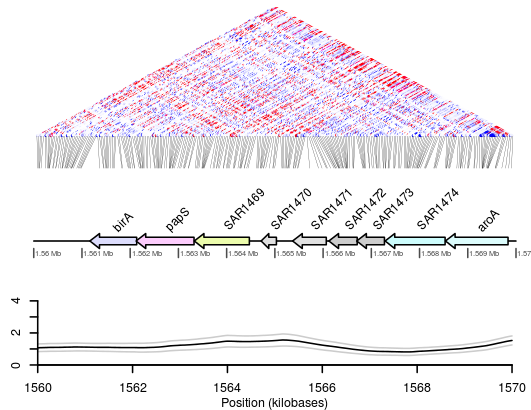

Supplement: Supplementary Data 1 — Homoplasy and linkage disequilibrium in the Staphylococcus aureus core genome. Whole-genome LD plots are illustrated in 10kb windows. Each 10kb window is displayed as in Figure 3, with a single reference genome, MRSA252. Genes are color-coded by COG category or grey if unclassified. An extended coldspot can be seen between 1448-1458kb. [file ncomms4956-s2.zip › EverittSupplementaryDataset1/1560-1570.LD.png]

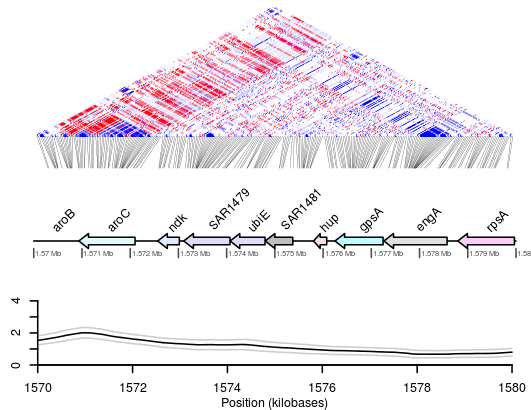

Supplement: Supplementary Data 1 — Homoplasy and linkage disequilibrium in the Staphylococcus aureus core genome. Whole-genome LD plots are illustrated in 10kb windows. Each 10kb window is displayed as in Figure 3, with a single reference genome, MRSA252. Genes are color-coded by COG category or grey if unclassified. An extended coldspot can be seen between 1448-1458kb. [file ncomms4956-s2.zip › EverittSupplementaryDataset1/1570-1580.LD.png]

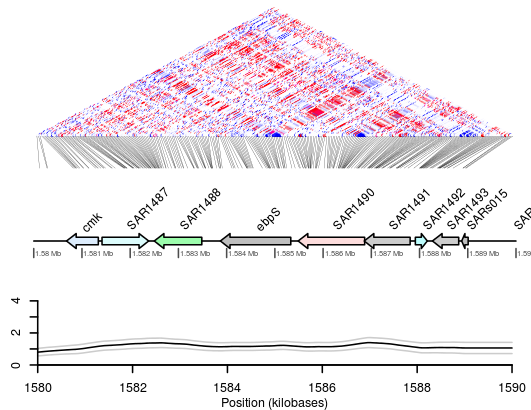

Supplement: Supplementary Data 1 — Homoplasy and linkage disequilibrium in the Staphylococcus aureus core genome. Whole-genome LD plots are illustrated in 10kb windows. Each 10kb window is displayed as in Figure 3, with a single reference genome, MRSA252. Genes are color-coded by COG category or grey if unclassified. An extended coldspot can be seen between 1448-1458kb. [file ncomms4956-s2.zip › EverittSupplementaryDataset1/1580-1590.LD.png]

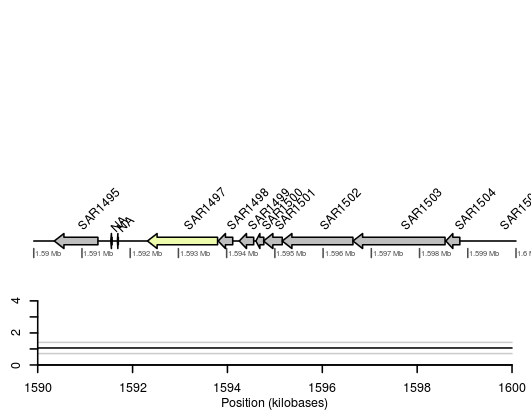

Supplement: Supplementary Data 1 — Homoplasy and linkage disequilibrium in the Staphylococcus aureus core genome. Whole-genome LD plots are illustrated in 10kb windows. Each 10kb window is displayed as in Figure 3, with a single reference genome, MRSA252. Genes are color-coded by COG category or grey if unclassified. An extended coldspot can be seen between 1448-1458kb. [file ncomms4956-s2.zip › EverittSupplementaryDataset1/1590-1600.LD.png]

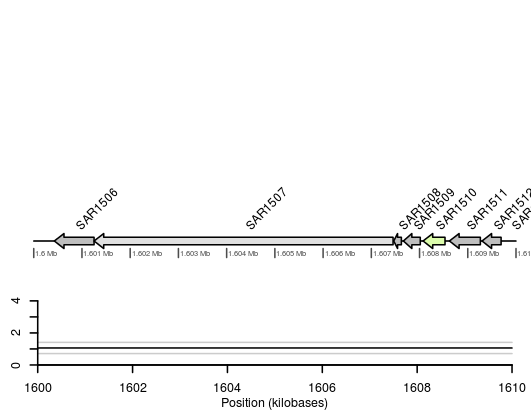

Supplement: Supplementary Data 1 — Homoplasy and linkage disequilibrium in the Staphylococcus aureus core genome. Whole-genome LD plots are illustrated in 10kb windows. Each 10kb window is displayed as in Figure 3, with a single reference genome, MRSA252. Genes are color-coded by COG category or grey if unclassified. An extended coldspot can be seen between 1448-1458kb. [file ncomms4956-s2.zip › EverittSupplementaryDataset1/1600-1610.LD.png]

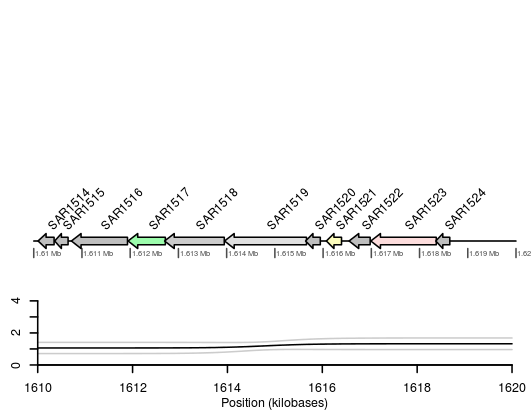

Supplement: Supplementary Data 1 — Homoplasy and linkage disequilibrium in the Staphylococcus aureus core genome. Whole-genome LD plots are illustrated in 10kb windows. Each 10kb window is displayed as in Figure 3, with a single reference genome, MRSA252. Genes are color-coded by COG category or grey if unclassified. An extended coldspot can be seen between 1448-1458kb. [file ncomms4956-s2.zip › EverittSupplementaryDataset1/1610-1620.LD.png]

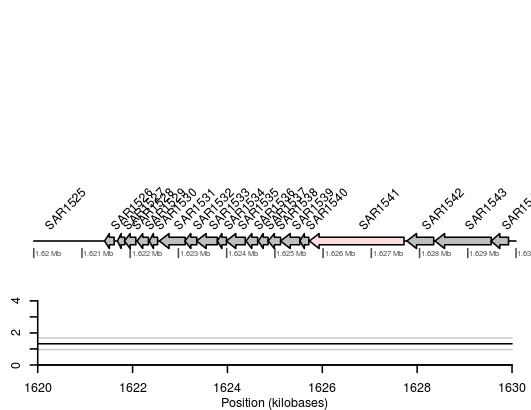

Supplement: Supplementary Data 1 — Homoplasy and linkage disequilibrium in the Staphylococcus aureus core genome. Whole-genome LD plots are illustrated in 10kb windows. Each 10kb window is displayed as in Figure 3, with a single reference genome, MRSA252. Genes are color-coded by COG category or grey if unclassified. An extended coldspot can be seen between 1448-1458kb. [file ncomms4956-s2.zip › EverittSupplementaryDataset1/1620-1630.LD.png]

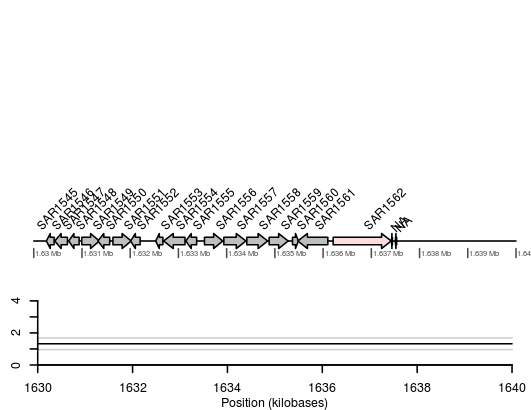

Supplement: Supplementary Data 1 — Homoplasy and linkage disequilibrium in the Staphylococcus aureus core genome. Whole-genome LD plots are illustrated in 10kb windows. Each 10kb window is displayed as in Figure 3, with a single reference genome, MRSA252. Genes are color-coded by COG category or grey if unclassified. An extended coldspot can be seen between 1448-1458kb. [file ncomms4956-s2.zip › EverittSupplementaryDataset1/1630-1640.LD.png]

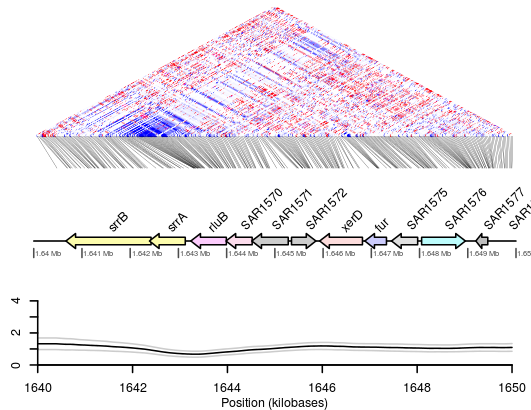

Supplement: Supplementary Data 1 — Homoplasy and linkage disequilibrium in the Staphylococcus aureus core genome. Whole-genome LD plots are illustrated in 10kb windows. Each 10kb window is displayed as in Figure 3, with a single reference genome, MRSA252. Genes are color-coded by COG category or grey if unclassified. An extended coldspot can be seen between 1448-1458kb. [file ncomms4956-s2.zip › EverittSupplementaryDataset1/1640-1650.LD.png]

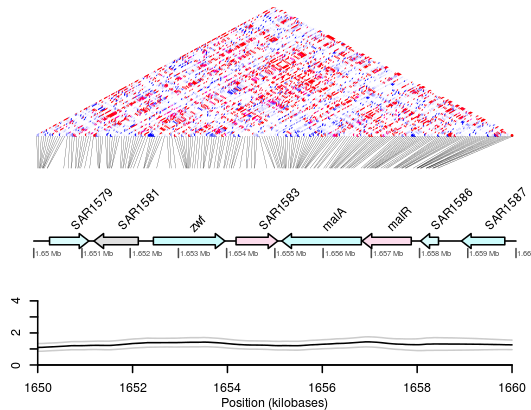

Supplement: Supplementary Data 1 — Homoplasy and linkage disequilibrium in the Staphylococcus aureus core genome. Whole-genome LD plots are illustrated in 10kb windows. Each 10kb window is displayed as in Figure 3, with a single reference genome, MRSA252. Genes are color-coded by COG category or grey if unclassified. An extended coldspot can be seen between 1448-1458kb. [file ncomms4956-s2.zip › EverittSupplementaryDataset1/1650-1660.LD.png]

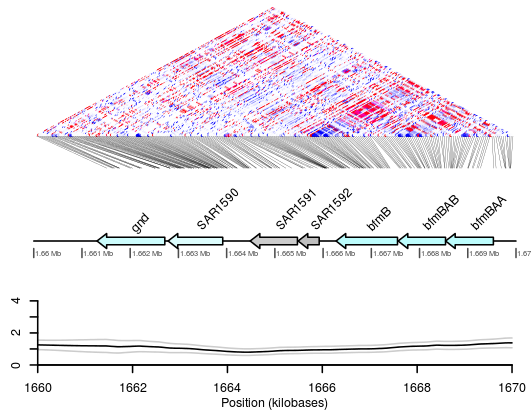

Supplement: Supplementary Data 1 — Homoplasy and linkage disequilibrium in the Staphylococcus aureus core genome. Whole-genome LD plots are illustrated in 10kb windows. Each 10kb window is displayed as in Figure 3, with a single reference genome, MRSA252. Genes are color-coded by COG category or grey if unclassified. An extended coldspot can be seen between 1448-1458kb. [file ncomms4956-s2.zip › EverittSupplementaryDataset1/1660-1670.LD.png]

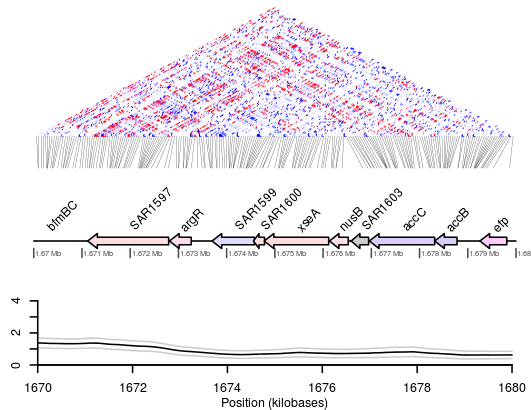

Supplement: Supplementary Data 1 — Homoplasy and linkage disequilibrium in the Staphylococcus aureus core genome. Whole-genome LD plots are illustrated in 10kb windows. Each 10kb window is displayed as in Figure 3, with a single reference genome, MRSA252. Genes are color-coded by COG category or grey if unclassified. An extended coldspot can be seen between 1448-1458kb. [file ncomms4956-s2.zip › EverittSupplementaryDataset1/1670-1680.LD.png]

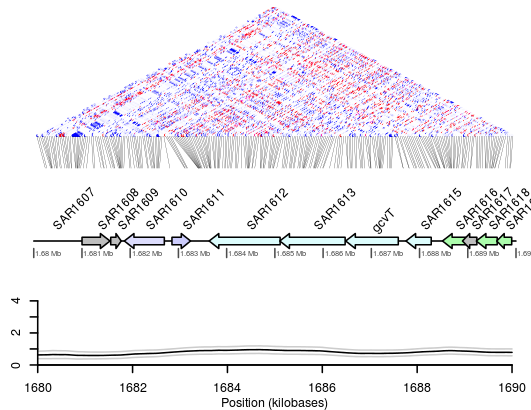

Supplement: Supplementary Data 1 — Homoplasy and linkage disequilibrium in the Staphylococcus aureus core genome. Whole-genome LD plots are illustrated in 10kb windows. Each 10kb window is displayed as in Figure 3, with a single reference genome, MRSA252. Genes are color-coded by COG category or grey if unclassified. An extended coldspot can be seen between 1448-1458kb. [file ncomms4956-s2.zip › EverittSupplementaryDataset1/1680-1690.LD.png]

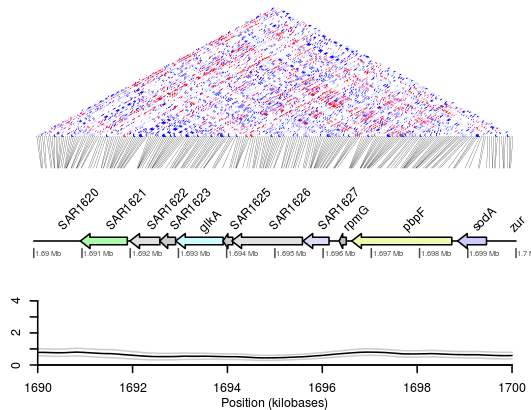

Supplement: Supplementary Data 1 — Homoplasy and linkage disequilibrium in the Staphylococcus aureus core genome. Whole-genome LD plots are illustrated in 10kb windows. Each 10kb window is displayed as in Figure 3, with a single reference genome, MRSA252. Genes are color-coded by COG category or grey if unclassified. An extended coldspot can be seen between 1448-1458kb. [file ncomms4956-s2.zip › EverittSupplementaryDataset1/1690-1700.LD.png]

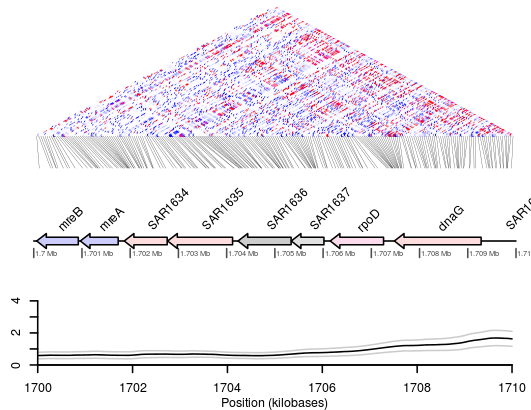

Supplement: Supplementary Data 1 — Homoplasy and linkage disequilibrium in the Staphylococcus aureus core genome. Whole-genome LD plots are illustrated in 10kb windows. Each 10kb window is displayed as in Figure 3, with a single reference genome, MRSA252. Genes are color-coded by COG category or grey if unclassified. An extended coldspot can be seen between 1448-1458kb. [file ncomms4956-s2.zip › EverittSupplementaryDataset1/1700-1710.LD.png]

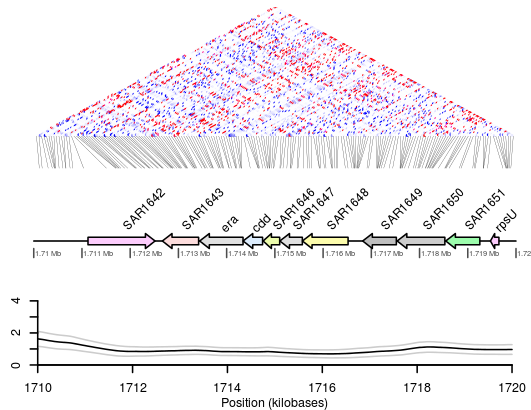

Supplement: Supplementary Data 1 — Homoplasy and linkage disequilibrium in the Staphylococcus aureus core genome. Whole-genome LD plots are illustrated in 10kb windows. Each 10kb window is displayed as in Figure 3, with a single reference genome, MRSA252. Genes are color-coded by COG category or grey if unclassified. An extended coldspot can be seen between 1448-1458kb. [file ncomms4956-s2.zip › EverittSupplementaryDataset1/1710-1720.LD.png]

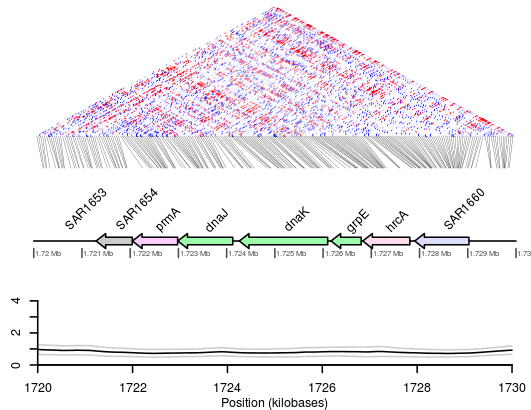

Supplement: Supplementary Data 1 — Homoplasy and linkage disequilibrium in the Staphylococcus aureus core genome. Whole-genome LD plots are illustrated in 10kb windows. Each 10kb window is displayed as in Figure 3, with a single reference genome, MRSA252. Genes are color-coded by COG category or grey if unclassified. An extended coldspot can be seen between 1448-1458kb. [file ncomms4956-s2.zip › EverittSupplementaryDataset1/1720-1730.LD.png]

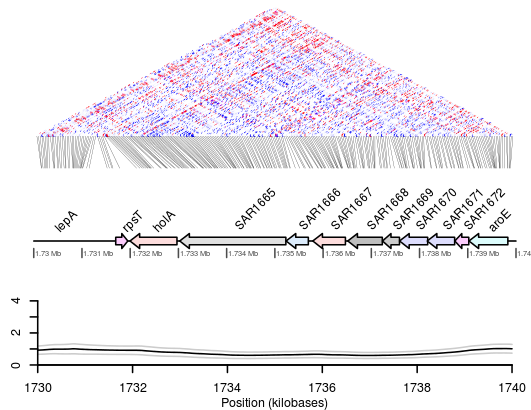

Supplement: Supplementary Data 1 — Homoplasy and linkage disequilibrium in the Staphylococcus aureus core genome. Whole-genome LD plots are illustrated in 10kb windows. Each 10kb window is displayed as in Figure 3, with a single reference genome, MRSA252. Genes are color-coded by COG category or grey if unclassified. An extended coldspot can be seen between 1448-1458kb. [file ncomms4956-s2.zip › EverittSupplementaryDataset1/1730-1740.LD.png]

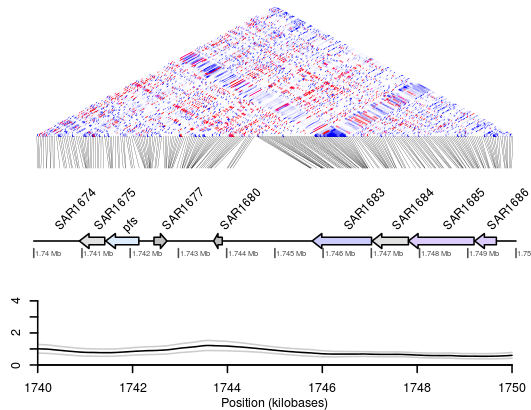

Supplement: Supplementary Data 1 — Homoplasy and linkage disequilibrium in the Staphylococcus aureus core genome. Whole-genome LD plots are illustrated in 10kb windows. Each 10kb window is displayed as in Figure 3, with a single reference genome, MRSA252. Genes are color-coded by COG category or grey if unclassified. An extended coldspot can be seen between 1448-1458kb. [file ncomms4956-s2.zip › EverittSupplementaryDataset1/1740-1750.LD.png]

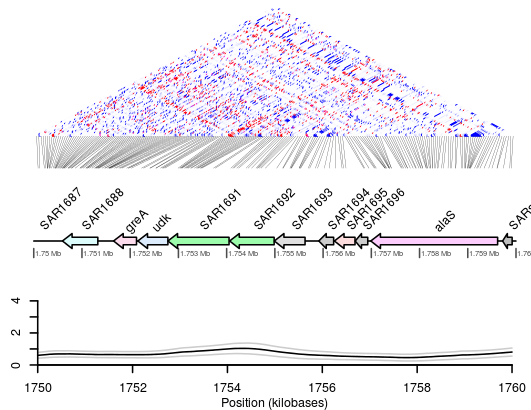

Supplement: Supplementary Data 1 — Homoplasy and linkage disequilibrium in the Staphylococcus aureus core genome. Whole-genome LD plots are illustrated in 10kb windows. Each 10kb window is displayed as in Figure 3, with a single reference genome, MRSA252. Genes are color-coded by COG category or grey if unclassified. An extended coldspot can be seen between 1448-1458kb. [file ncomms4956-s2.zip › EverittSupplementaryDataset1/1750-1760.LD.png]

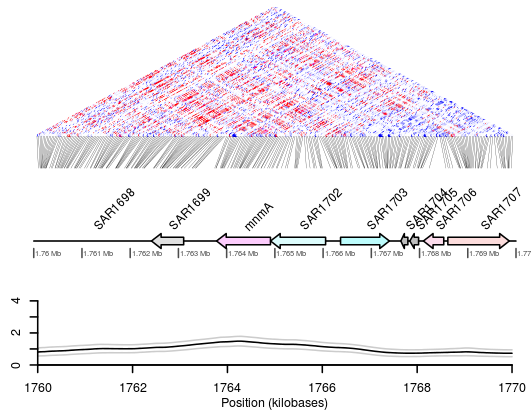

Supplement: Supplementary Data 1 — Homoplasy and linkage disequilibrium in the Staphylococcus aureus core genome. Whole-genome LD plots are illustrated in 10kb windows. Each 10kb window is displayed as in Figure 3, with a single reference genome, MRSA252. Genes are color-coded by COG category or grey if unclassified. An extended coldspot can be seen between 1448-1458kb. [file ncomms4956-s2.zip › EverittSupplementaryDataset1/1760-1770.LD.png]

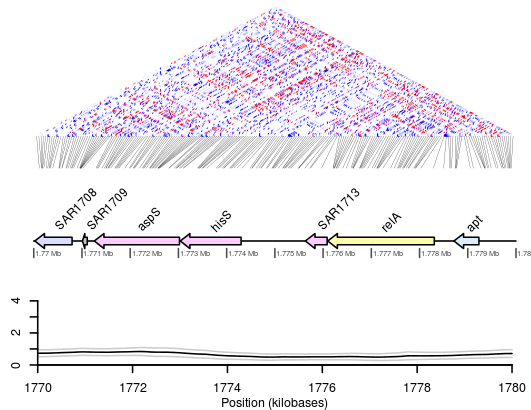

Supplement: Supplementary Data 1 — Homoplasy and linkage disequilibrium in the Staphylococcus aureus core genome. Whole-genome LD plots are illustrated in 10kb windows. Each 10kb window is displayed as in Figure 3, with a single reference genome, MRSA252. Genes are color-coded by COG category or grey if unclassified. An extended coldspot can be seen between 1448-1458kb. [file ncomms4956-s2.zip › EverittSupplementaryDataset1/1770-1780.LD.png]

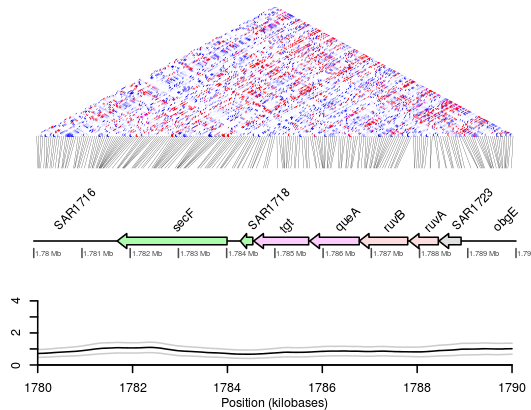

Supplement: Supplementary Data 1 — Homoplasy and linkage disequilibrium in the Staphylococcus aureus core genome. Whole-genome LD plots are illustrated in 10kb windows. Each 10kb window is displayed as in Figure 3, with a single reference genome, MRSA252. Genes are color-coded by COG category or grey if unclassified. An extended coldspot can be seen between 1448-1458kb. [file ncomms4956-s2.zip › EverittSupplementaryDataset1/1780-1790.LD.png]

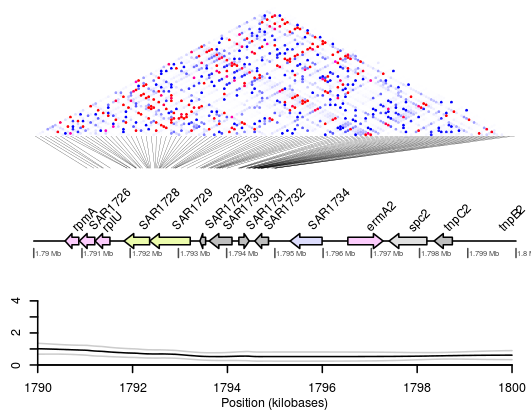

Supplement: Supplementary Data 1 — Homoplasy and linkage disequilibrium in the Staphylococcus aureus core genome. Whole-genome LD plots are illustrated in 10kb windows. Each 10kb window is displayed as in Figure 3, with a single reference genome, MRSA252. Genes are color-coded by COG category or grey if unclassified. An extended coldspot can be seen between 1448-1458kb. [file ncomms4956-s2.zip › EverittSupplementaryDataset1/1790-1800.LD.png]

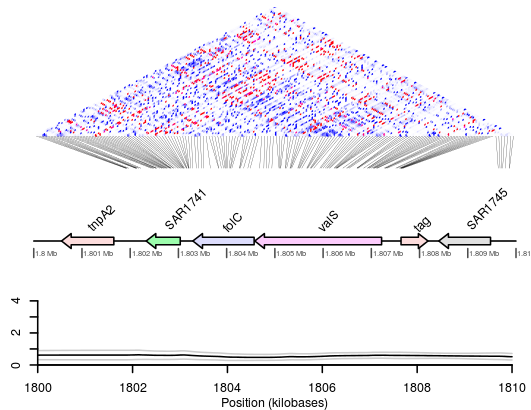

Supplement: Supplementary Data 1 — Homoplasy and linkage disequilibrium in the Staphylococcus aureus core genome. Whole-genome LD plots are illustrated in 10kb windows. Each 10kb window is displayed as in Figure 3, with a single reference genome, MRSA252. Genes are color-coded by COG category or grey if unclassified. An extended coldspot can be seen between 1448-1458kb. [file ncomms4956-s2.zip › EverittSupplementaryDataset1/1800-1810.LD.png]

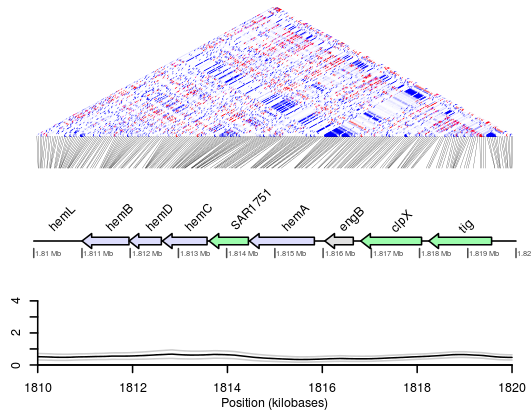

Supplement: Supplementary Data 1 — Homoplasy and linkage disequilibrium in the Staphylococcus aureus core genome. Whole-genome LD plots are illustrated in 10kb windows. Each 10kb window is displayed as in Figure 3, with a single reference genome, MRSA252. Genes are color-coded by COG category or grey if unclassified. An extended coldspot can be seen between 1448-1458kb. [file ncomms4956-s2.zip › EverittSupplementaryDataset1/1810-1820.LD.png]

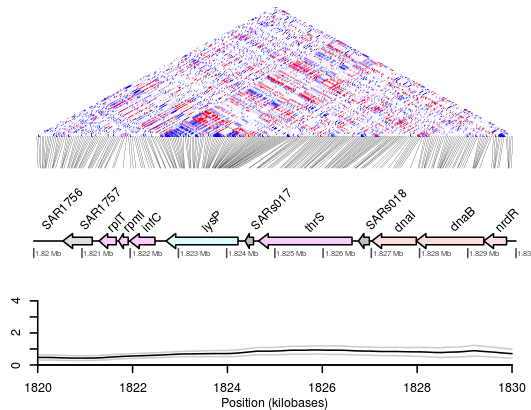

Supplement: Supplementary Data 1 — Homoplasy and linkage disequilibrium in the Staphylococcus aureus core genome. Whole-genome LD plots are illustrated in 10kb windows. Each 10kb window is displayed as in Figure 3, with a single reference genome, MRSA252. Genes are color-coded by COG category or grey if unclassified. An extended coldspot can be seen between 1448-1458kb. [file ncomms4956-s2.zip › EverittSupplementaryDataset1/1820-1830.LD.png]

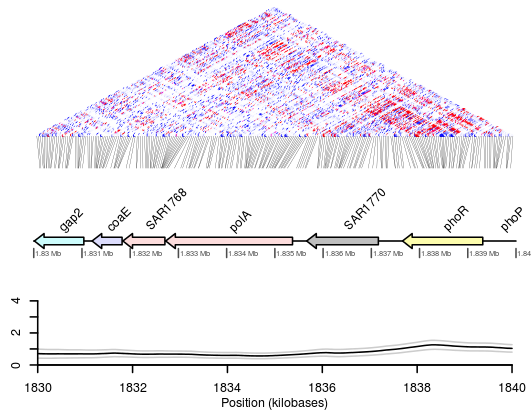

Supplement: Supplementary Data 1 — Homoplasy and linkage disequilibrium in the Staphylococcus aureus core genome. Whole-genome LD plots are illustrated in 10kb windows. Each 10kb window is displayed as in Figure 3, with a single reference genome, MRSA252. Genes are color-coded by COG category or grey if unclassified. An extended coldspot can be seen between 1448-1458kb. [file ncomms4956-s2.zip › EverittSupplementaryDataset1/1830-1840.LD.png]

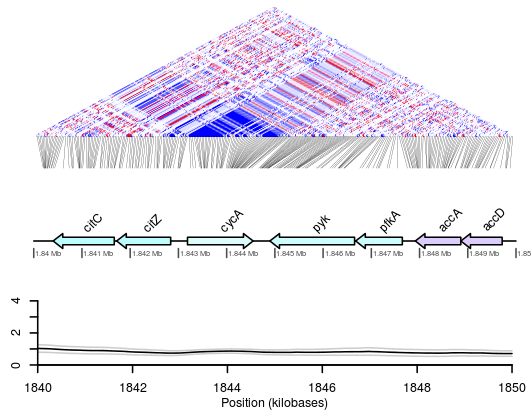

Supplement: Supplementary Data 1 — Homoplasy and linkage disequilibrium in the Staphylococcus aureus core genome. Whole-genome LD plots are illustrated in 10kb windows. Each 10kb window is displayed as in Figure 3, with a single reference genome, MRSA252. Genes are color-coded by COG category or grey if unclassified. An extended coldspot can be seen between 1448-1458kb. [file ncomms4956-s2.zip › EverittSupplementaryDataset1/1840-1850.LD.png]

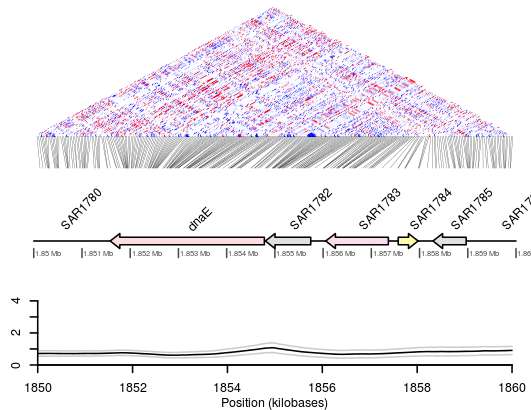

Supplement: Supplementary Data 1 — Homoplasy and linkage disequilibrium in the Staphylococcus aureus core genome. Whole-genome LD plots are illustrated in 10kb windows. Each 10kb window is displayed as in Figure 3, with a single reference genome, MRSA252. Genes are color-coded by COG category or grey if unclassified. An extended coldspot can be seen between 1448-1458kb. [file ncomms4956-s2.zip › EverittSupplementaryDataset1/1850-1860.LD.png]

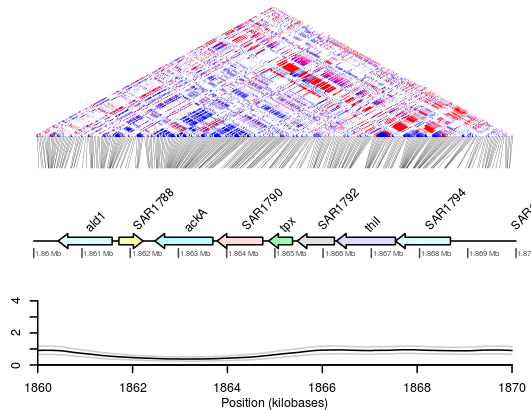

Supplement: Supplementary Data 1 — Homoplasy and linkage disequilibrium in the Staphylococcus aureus core genome. Whole-genome LD plots are illustrated in 10kb windows. Each 10kb window is displayed as in Figure 3, with a single reference genome, MRSA252. Genes are color-coded by COG category or grey if unclassified. An extended coldspot can be seen between 1448-1458kb. [file ncomms4956-s2.zip › EverittSupplementaryDataset1/1860-1870.LD.png]

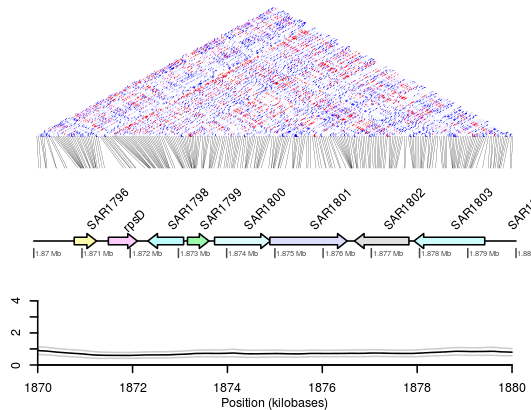

Supplement: Supplementary Data 1 — Homoplasy and linkage disequilibrium in the Staphylococcus aureus core genome. Whole-genome LD plots are illustrated in 10kb windows. Each 10kb window is displayed as in Figure 3, with a single reference genome, MRSA252. Genes are color-coded by COG category or grey if unclassified. An extended coldspot can be seen between 1448-1458kb. [file ncomms4956-s2.zip › EverittSupplementaryDataset1/1870-1880.LD.png]

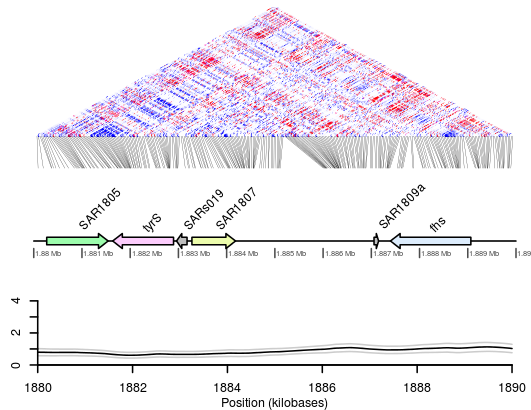

Supplement: Supplementary Data 1 — Homoplasy and linkage disequilibrium in the Staphylococcus aureus core genome. Whole-genome LD plots are illustrated in 10kb windows. Each 10kb window is displayed as in Figure 3, with a single reference genome, MRSA252. Genes are color-coded by COG category or grey if unclassified. An extended coldspot can be seen between 1448-1458kb. [file ncomms4956-s2.zip › EverittSupplementaryDataset1/1880-1890.LD.png]

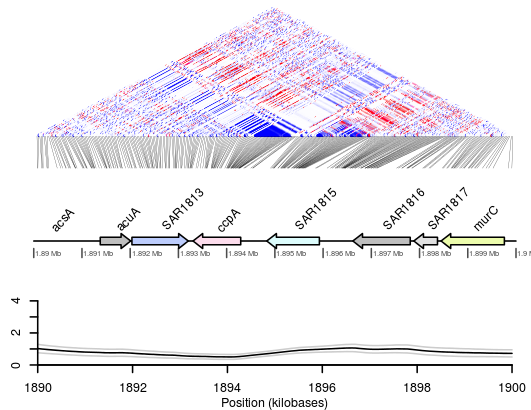

Supplement: Supplementary Data 1 — Homoplasy and linkage disequilibrium in the Staphylococcus aureus core genome. Whole-genome LD plots are illustrated in 10kb windows. Each 10kb window is displayed as in Figure 3, with a single reference genome, MRSA252. Genes are color-coded by COG category or grey if unclassified. An extended coldspot can be seen between 1448-1458kb. [file ncomms4956-s2.zip › EverittSupplementaryDataset1/1890-1900.LD.png]

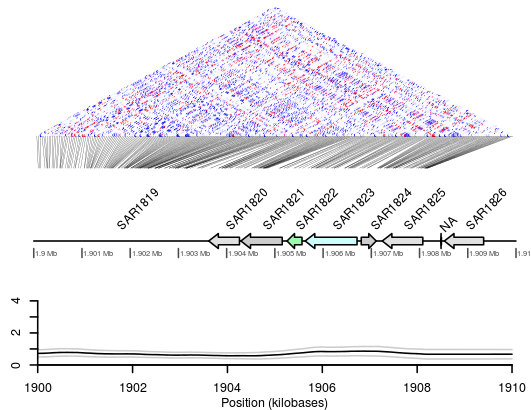

Supplement: Supplementary Data 1 — Homoplasy and linkage disequilibrium in the Staphylococcus aureus core genome. Whole-genome LD plots are illustrated in 10kb windows. Each 10kb window is displayed as in Figure 3, with a single reference genome, MRSA252. Genes are color-coded by COG category or grey if unclassified. An extended coldspot can be seen between 1448-1458kb. [file ncomms4956-s2.zip › EverittSupplementaryDataset1/1900-1910.LD.png]

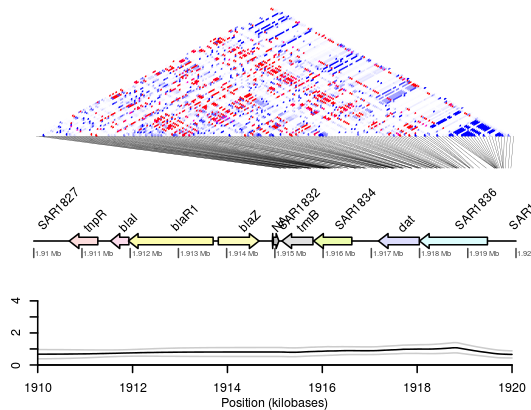

Supplement: Supplementary Data 1 — Homoplasy and linkage disequilibrium in the Staphylococcus aureus core genome. Whole-genome LD plots are illustrated in 10kb windows. Each 10kb window is displayed as in Figure 3, with a single reference genome, MRSA252. Genes are color-coded by COG category or grey if unclassified. An extended coldspot can be seen between 1448-1458kb. [file ncomms4956-s2.zip › EverittSupplementaryDataset1/1910-1920.LD.png]

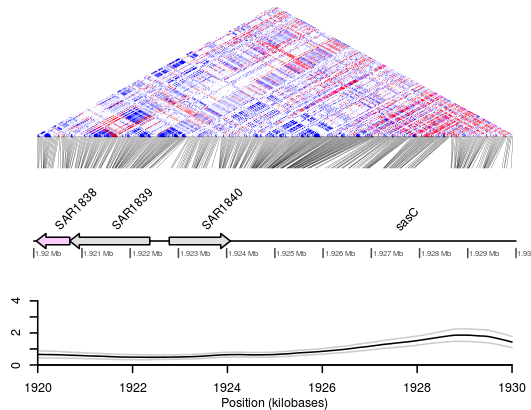

Supplement: Supplementary Data 1 — Homoplasy and linkage disequilibrium in the Staphylococcus aureus core genome. Whole-genome LD plots are illustrated in 10kb windows. Each 10kb window is displayed as in Figure 3, with a single reference genome, MRSA252. Genes are color-coded by COG category or grey if unclassified. An extended coldspot can be seen between 1448-1458kb. [file ncomms4956-s2.zip › EverittSupplementaryDataset1/1920-1930.LD.png]

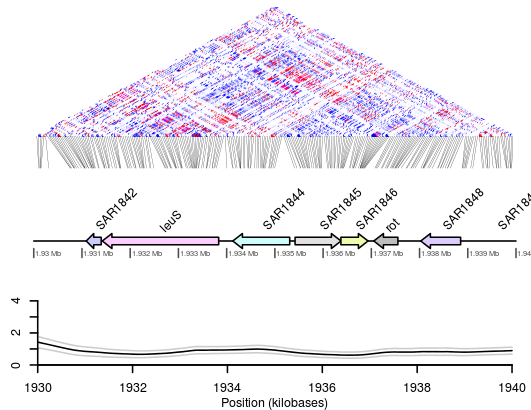

Supplement: Supplementary Data 1 — Homoplasy and linkage disequilibrium in the Staphylococcus aureus core genome. Whole-genome LD plots are illustrated in 10kb windows. Each 10kb window is displayed as in Figure 3, with a single reference genome, MRSA252. Genes are color-coded by COG category or grey if unclassified. An extended coldspot can be seen between 1448-1458kb. [file ncomms4956-s2.zip › EverittSupplementaryDataset1/1930-1940.LD.png]

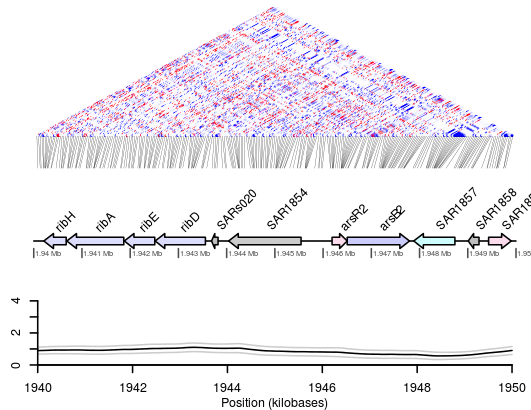

Supplement: Supplementary Data 1 — Homoplasy and linkage disequilibrium in the Staphylococcus aureus core genome. Whole-genome LD plots are illustrated in 10kb windows. Each 10kb window is displayed as in Figure 3, with a single reference genome, MRSA252. Genes are color-coded by COG category or grey if unclassified. An extended coldspot can be seen between 1448-1458kb. [file ncomms4956-s2.zip › EverittSupplementaryDataset1/1940-1950.LD.png]

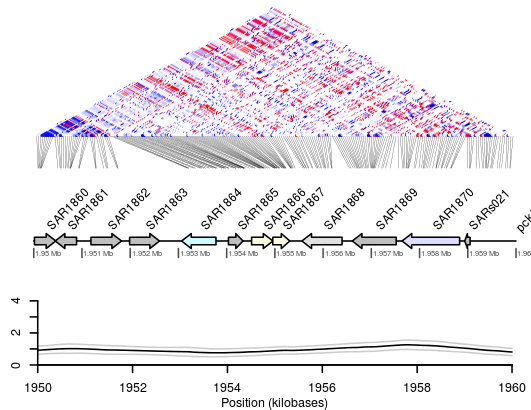

Supplement: Supplementary Data 1 — Homoplasy and linkage disequilibrium in the Staphylococcus aureus core genome. Whole-genome LD plots are illustrated in 10kb windows. Each 10kb window is displayed as in Figure 3, with a single reference genome, MRSA252. Genes are color-coded by COG category or grey if unclassified. An extended coldspot can be seen between 1448-1458kb. [file ncomms4956-s2.zip › EverittSupplementaryDataset1/1950-1960.LD.png]

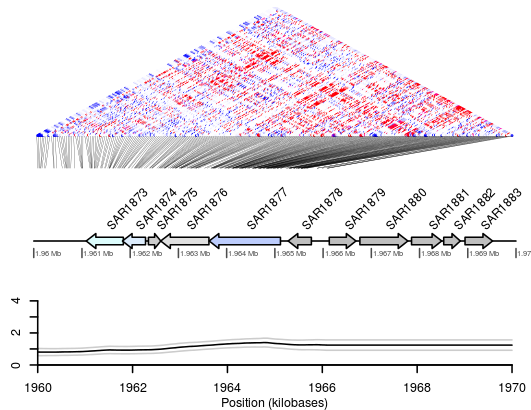

Supplement: Supplementary Data 1 — Homoplasy and linkage disequilibrium in the Staphylococcus aureus core genome. Whole-genome LD plots are illustrated in 10kb windows. Each 10kb window is displayed as in Figure 3, with a single reference genome, MRSA252. Genes are color-coded by COG category or grey if unclassified. An extended coldspot can be seen between 1448-1458kb. [file ncomms4956-s2.zip › EverittSupplementaryDataset1/1960-1970.LD.png]

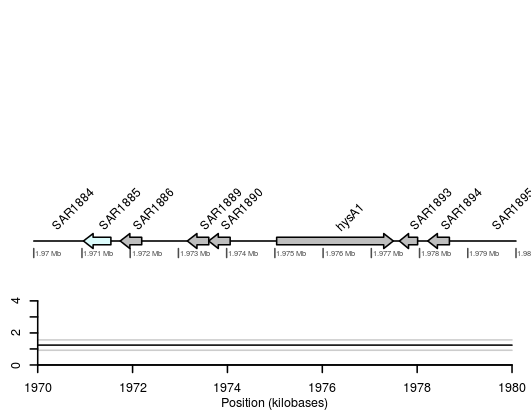

Supplement: Supplementary Data 1 — Homoplasy and linkage disequilibrium in the Staphylococcus aureus core genome. Whole-genome LD plots are illustrated in 10kb windows. Each 10kb window is displayed as in Figure 3, with a single reference genome, MRSA252. Genes are color-coded by COG category or grey if unclassified. An extended coldspot can be seen between 1448-1458kb. [file ncomms4956-s2.zip › EverittSupplementaryDataset1/1970-1980.LD.png]

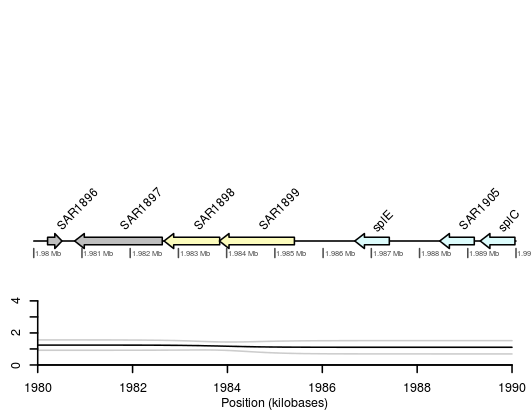

Supplement: Supplementary Data 1 — Homoplasy and linkage disequilibrium in the Staphylococcus aureus core genome. Whole-genome LD plots are illustrated in 10kb windows. Each 10kb window is displayed as in Figure 3, with a single reference genome, MRSA252. Genes are color-coded by COG category or grey if unclassified. An extended coldspot can be seen between 1448-1458kb. [file ncomms4956-s2.zip › EverittSupplementaryDataset1/1980-1990.LD.png]

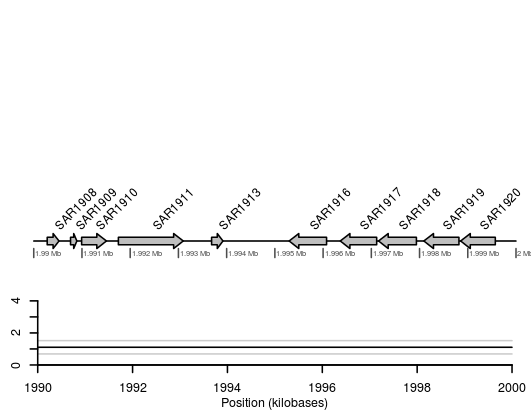

Supplement: Supplementary Data 1 — Homoplasy and linkage disequilibrium in the Staphylococcus aureus core genome. Whole-genome LD plots are illustrated in 10kb windows. Each 10kb window is displayed as in Figure 3, with a single reference genome, MRSA252. Genes are color-coded by COG category or grey if unclassified. An extended coldspot can be seen between 1448-1458kb. [file ncomms4956-s2.zip › EverittSupplementaryDataset1/1990-2000.LD.png]
