# Supplementary figures and images for: Mobile elements drive recombination hotspots in the core genome of Staphylococcus aureus (part 1 of 2)
Source: Nat Commun. 2014 May 23;5:3956. doi: 10.1038/ncomms4956 (PMC4036114; doi:10.1038/ncomms4956)

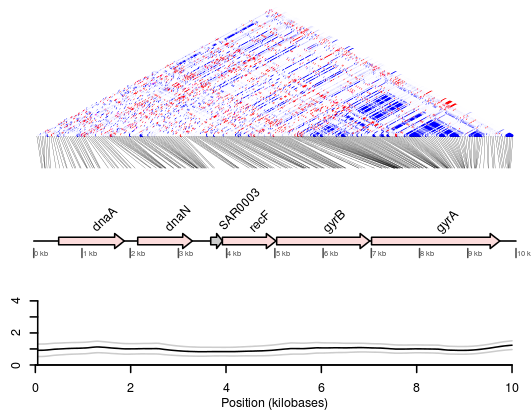

Supplement: Supplementary Data 1 — Homoplasy and linkage disequilibrium in the Staphylococcus aureus core genome. Whole-genome LD plots are illustrated in 10kb windows. Each 10kb window is displayed as in Figure 3, with a single reference genome, MRSA252. Genes are color-coded by COG category or grey if unclassified. An extended coldspot can be seen between 1448-1458kb. [file ncomms4956-s2.zip › EverittSupplementaryDataset1/0000-0010.LD.png]

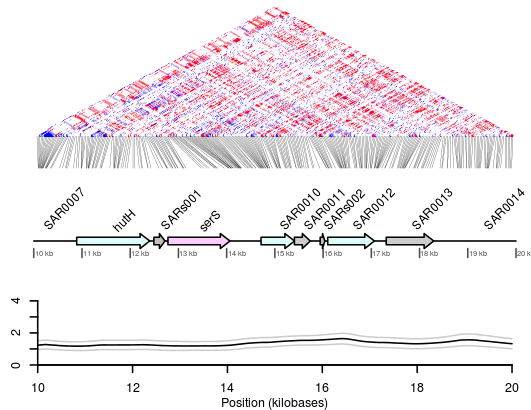

Supplement: Supplementary Data 1 — Homoplasy and linkage disequilibrium in the Staphylococcus aureus core genome. Whole-genome LD plots are illustrated in 10kb windows. Each 10kb window is displayed as in Figure 3, with a single reference genome, MRSA252. Genes are color-coded by COG category or grey if unclassified. An extended coldspot can be seen between 1448-1458kb. [file ncomms4956-s2.zip › EverittSupplementaryDataset1/0010-0020.LD.png]

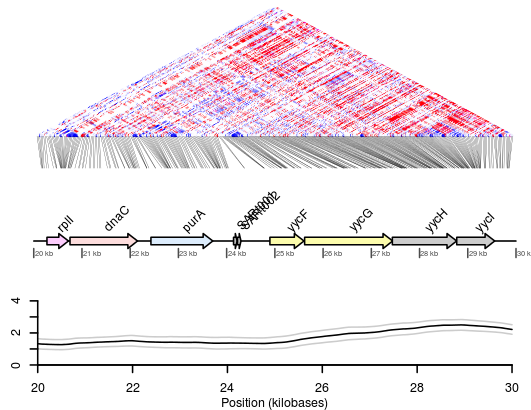

Supplement: Supplementary Data 1 — Homoplasy and linkage disequilibrium in the Staphylococcus aureus core genome. Whole-genome LD plots are illustrated in 10kb windows. Each 10kb window is displayed as in Figure 3, with a single reference genome, MRSA252. Genes are color-coded by COG category or grey if unclassified. An extended coldspot can be seen between 1448-1458kb. [file ncomms4956-s2.zip › EverittSupplementaryDataset1/0020-0030.LD.png]

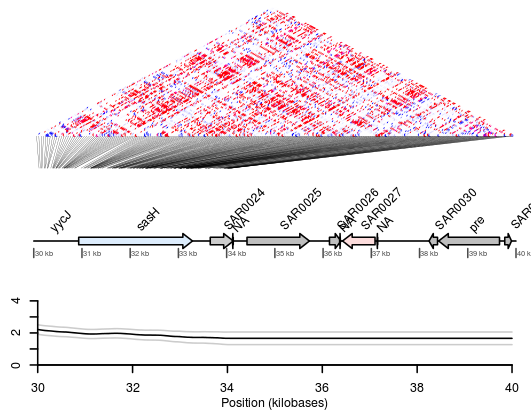

Supplement: Supplementary Data 1 — Homoplasy and linkage disequilibrium in the Staphylococcus aureus core genome. Whole-genome LD plots are illustrated in 10kb windows. Each 10kb window is displayed as in Figure 3, with a single reference genome, MRSA252. Genes are color-coded by COG category or grey if unclassified. An extended coldspot can be seen between 1448-1458kb. [file ncomms4956-s2.zip › EverittSupplementaryDataset1/0030-0040.LD.png]

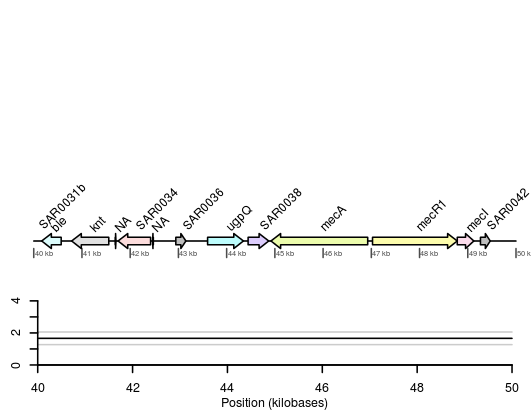

Supplement: Supplementary Data 1 — Homoplasy and linkage disequilibrium in the Staphylococcus aureus core genome. Whole-genome LD plots are illustrated in 10kb windows. Each 10kb window is displayed as in Figure 3, with a single reference genome, MRSA252. Genes are color-coded by COG category or grey if unclassified. An extended coldspot can be seen between 1448-1458kb. [file ncomms4956-s2.zip › EverittSupplementaryDataset1/0040-0050.LD.png]

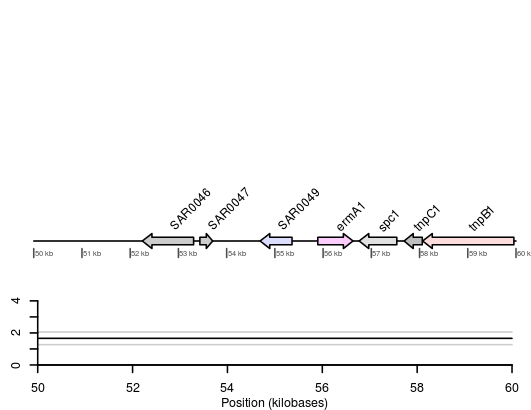

Supplement: Supplementary Data 1 — Homoplasy and linkage disequilibrium in the Staphylococcus aureus core genome. Whole-genome LD plots are illustrated in 10kb windows. Each 10kb window is displayed as in Figure 3, with a single reference genome, MRSA252. Genes are color-coded by COG category or grey if unclassified. An extended coldspot can be seen between 1448-1458kb. [file ncomms4956-s2.zip › EverittSupplementaryDataset1/0050-0060.LD.png]

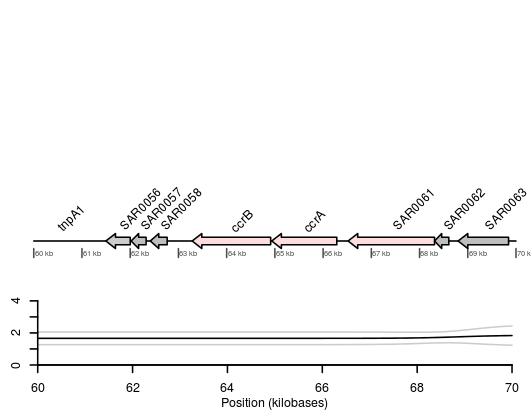

Supplement: Supplementary Data 1 — Homoplasy and linkage disequilibrium in the Staphylococcus aureus core genome. Whole-genome LD plots are illustrated in 10kb windows. Each 10kb window is displayed as in Figure 3, with a single reference genome, MRSA252. Genes are color-coded by COG category or grey if unclassified. An extended coldspot can be seen between 1448-1458kb. [file ncomms4956-s2.zip › EverittSupplementaryDataset1/0060-0070.LD.png]

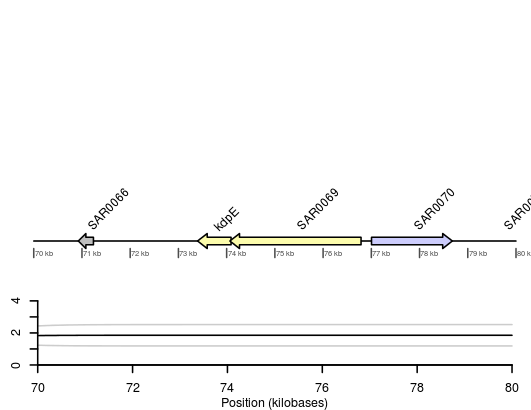

Supplement: Supplementary Data 1 — Homoplasy and linkage disequilibrium in the Staphylococcus aureus core genome. Whole-genome LD plots are illustrated in 10kb windows. Each 10kb window is displayed as in Figure 3, with a single reference genome, MRSA252. Genes are color-coded by COG category or grey if unclassified. An extended coldspot can be seen between 1448-1458kb. [file ncomms4956-s2.zip › EverittSupplementaryDataset1/0070-0080.LD.png]

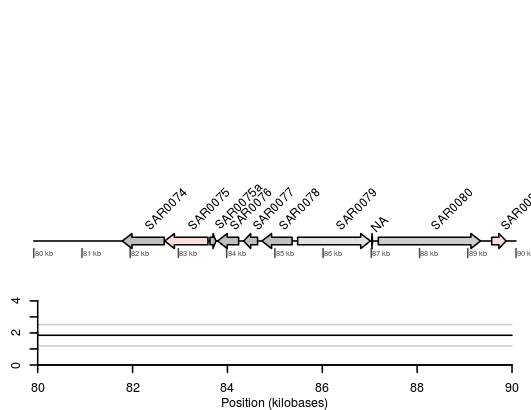

Supplement: Supplementary Data 1 — Homoplasy and linkage disequilibrium in the Staphylococcus aureus core genome. Whole-genome LD plots are illustrated in 10kb windows. Each 10kb window is displayed as in Figure 3, with a single reference genome, MRSA252. Genes are color-coded by COG category or grey if unclassified. An extended coldspot can be seen between 1448-1458kb. [file ncomms4956-s2.zip › EverittSupplementaryDataset1/0080-0090.LD.png]

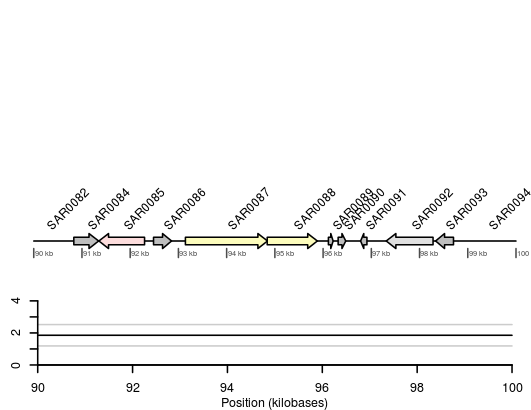

Supplement: Supplementary Data 1 — Homoplasy and linkage disequilibrium in the Staphylococcus aureus core genome. Whole-genome LD plots are illustrated in 10kb windows. Each 10kb window is displayed as in Figure 3, with a single reference genome, MRSA252. Genes are color-coded by COG category or grey if unclassified. An extended coldspot can be seen between 1448-1458kb. [file ncomms4956-s2.zip › EverittSupplementaryDataset1/0090-0100.LD.png]

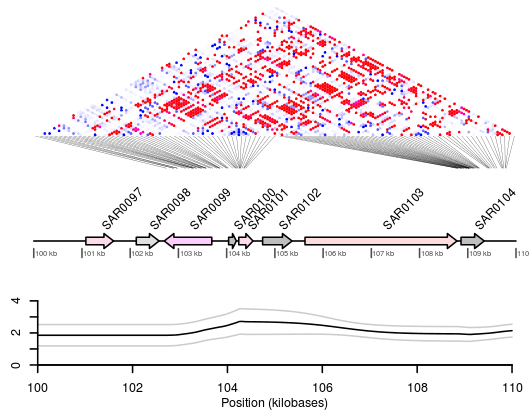

Supplement: Supplementary Data 1 — Homoplasy and linkage disequilibrium in the Staphylococcus aureus core genome. Whole-genome LD plots are illustrated in 10kb windows. Each 10kb window is displayed as in Figure 3, with a single reference genome, MRSA252. Genes are color-coded by COG category or grey if unclassified. An extended coldspot can be seen between 1448-1458kb. [file ncomms4956-s2.zip › EverittSupplementaryDataset1/0100-0110.LD.png]

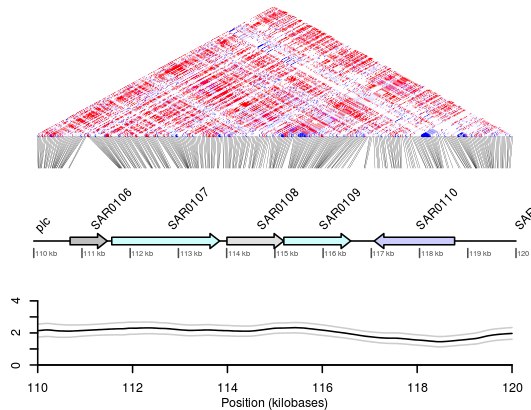

Supplement: Supplementary Data 1 — Homoplasy and linkage disequilibrium in the Staphylococcus aureus core genome. Whole-genome LD plots are illustrated in 10kb windows. Each 10kb window is displayed as in Figure 3, with a single reference genome, MRSA252. Genes are color-coded by COG category or grey if unclassified. An extended coldspot can be seen between 1448-1458kb. [file ncomms4956-s2.zip › EverittSupplementaryDataset1/0110-0120.LD.png]

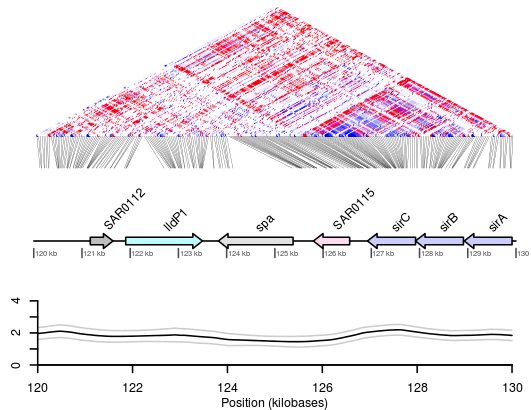

Supplement: Supplementary Data 1 — Homoplasy and linkage disequilibrium in the Staphylococcus aureus core genome. Whole-genome LD plots are illustrated in 10kb windows. Each 10kb window is displayed as in Figure 3, with a single reference genome, MRSA252. Genes are color-coded by COG category or grey if unclassified. An extended coldspot can be seen between 1448-1458kb. [file ncomms4956-s2.zip › EverittSupplementaryDataset1/0120-0130.LD.png]

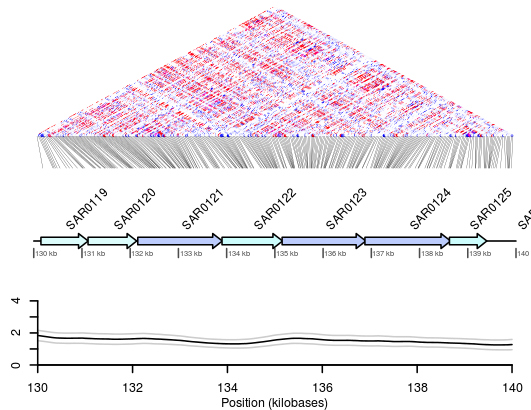

Supplement: Supplementary Data 1 — Homoplasy and linkage disequilibrium in the Staphylococcus aureus core genome. Whole-genome LD plots are illustrated in 10kb windows. Each 10kb window is displayed as in Figure 3, with a single reference genome, MRSA252. Genes are color-coded by COG category or grey if unclassified. An extended coldspot can be seen between 1448-1458kb. [file ncomms4956-s2.zip › EverittSupplementaryDataset1/0130-0140.LD.png]

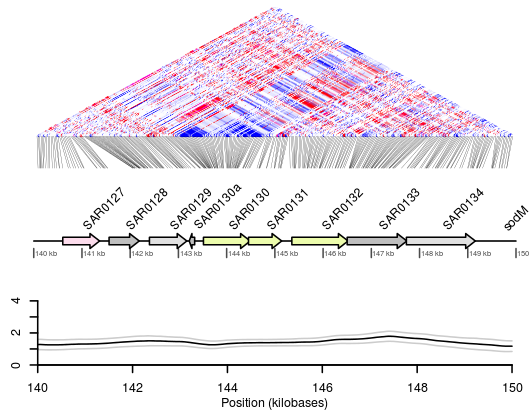

Supplement: Supplementary Data 1 — Homoplasy and linkage disequilibrium in the Staphylococcus aureus core genome. Whole-genome LD plots are illustrated in 10kb windows. Each 10kb window is displayed as in Figure 3, with a single reference genome, MRSA252. Genes are color-coded by COG category or grey if unclassified. An extended coldspot can be seen between 1448-1458kb. [file ncomms4956-s2.zip › EverittSupplementaryDataset1/0140-0150.LD.png]

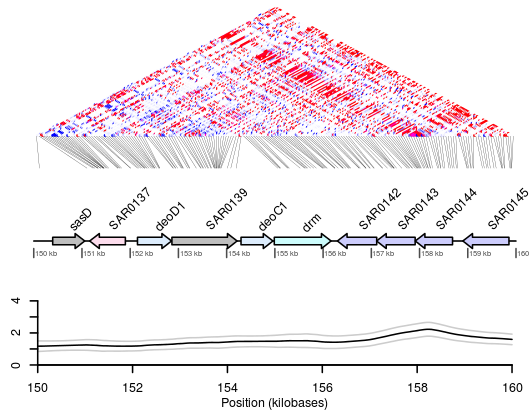

Supplement: Supplementary Data 1 — Homoplasy and linkage disequilibrium in the Staphylococcus aureus core genome. Whole-genome LD plots are illustrated in 10kb windows. Each 10kb window is displayed as in Figure 3, with a single reference genome, MRSA252. Genes are color-coded by COG category or grey if unclassified. An extended coldspot can be seen between 1448-1458kb. [file ncomms4956-s2.zip › EverittSupplementaryDataset1/0150-0160.LD.png]

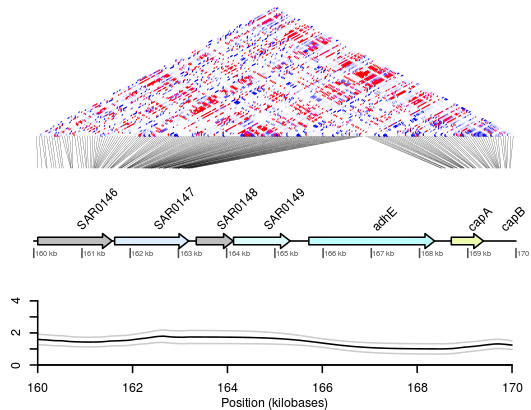

Supplement: Supplementary Data 1 — Homoplasy and linkage disequilibrium in the Staphylococcus aureus core genome. Whole-genome LD plots are illustrated in 10kb windows. Each 10kb window is displayed as in Figure 3, with a single reference genome, MRSA252. Genes are color-coded by COG category or grey if unclassified. An extended coldspot can be seen between 1448-1458kb. [file ncomms4956-s2.zip › EverittSupplementaryDataset1/0160-0170.LD.png]

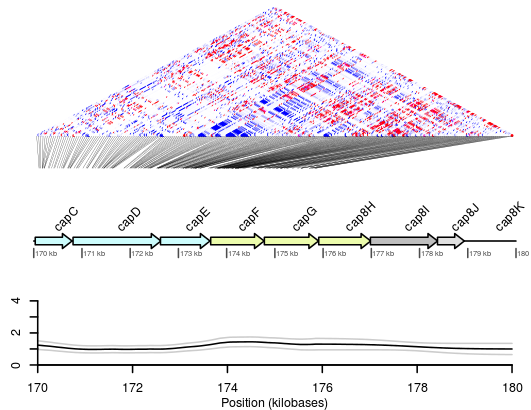

Supplement: Supplementary Data 1 — Homoplasy and linkage disequilibrium in the Staphylococcus aureus core genome. Whole-genome LD plots are illustrated in 10kb windows. Each 10kb window is displayed as in Figure 3, with a single reference genome, MRSA252. Genes are color-coded by COG category or grey if unclassified. An extended coldspot can be seen between 1448-1458kb. [file ncomms4956-s2.zip › EverittSupplementaryDataset1/0170-0180.LD.png]

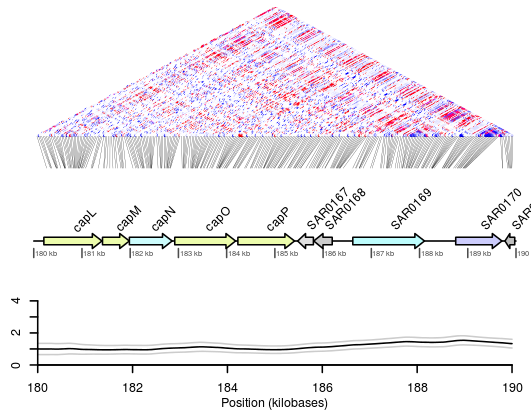

Supplement: Supplementary Data 1 — Homoplasy and linkage disequilibrium in the Staphylococcus aureus core genome. Whole-genome LD plots are illustrated in 10kb windows. Each 10kb window is displayed as in Figure 3, with a single reference genome, MRSA252. Genes are color-coded by COG category or grey if unclassified. An extended coldspot can be seen between 1448-1458kb. [file ncomms4956-s2.zip › EverittSupplementaryDataset1/0180-0190.LD.png]

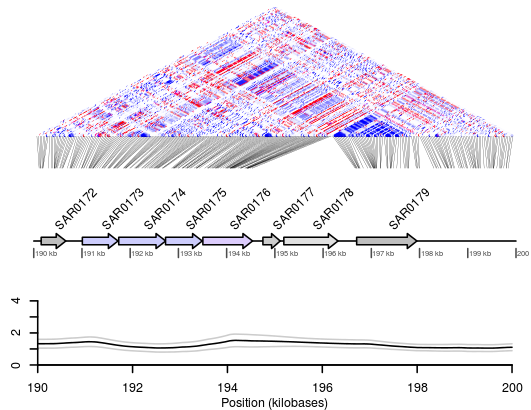

Supplement: Supplementary Data 1 — Homoplasy and linkage disequilibrium in the Staphylococcus aureus core genome. Whole-genome LD plots are illustrated in 10kb windows. Each 10kb window is displayed as in Figure 3, with a single reference genome, MRSA252. Genes are color-coded by COG category or grey if unclassified. An extended coldspot can be seen between 1448-1458kb. [file ncomms4956-s2.zip › EverittSupplementaryDataset1/0190-0200.LD.png]

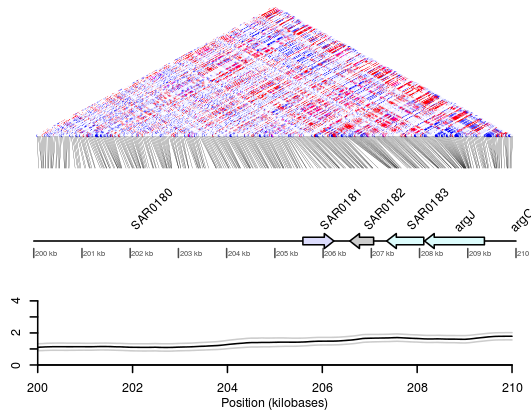

Supplement: Supplementary Data 1 — Homoplasy and linkage disequilibrium in the Staphylococcus aureus core genome. Whole-genome LD plots are illustrated in 10kb windows. Each 10kb window is displayed as in Figure 3, with a single reference genome, MRSA252. Genes are color-coded by COG category or grey if unclassified. An extended coldspot can be seen between 1448-1458kb. [file ncomms4956-s2.zip › EverittSupplementaryDataset1/0200-0210.LD.png]

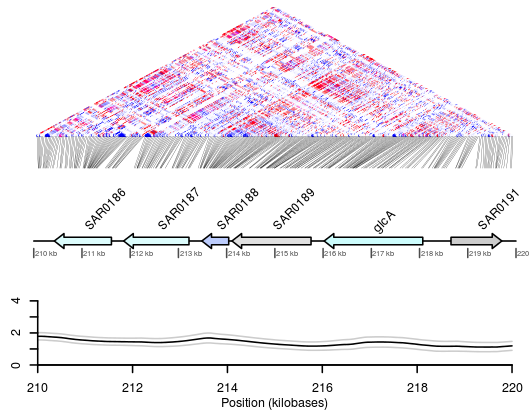

Supplement: Supplementary Data 1 — Homoplasy and linkage disequilibrium in the Staphylococcus aureus core genome. Whole-genome LD plots are illustrated in 10kb windows. Each 10kb window is displayed as in Figure 3, with a single reference genome, MRSA252. Genes are color-coded by COG category or grey if unclassified. An extended coldspot can be seen between 1448-1458kb. [file ncomms4956-s2.zip › EverittSupplementaryDataset1/0210-0220.LD.png]

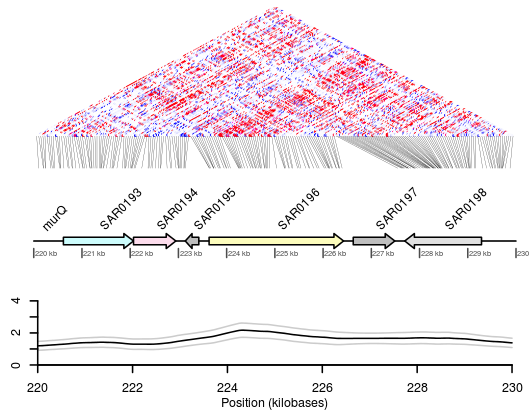

Supplement: Supplementary Data 1 — Homoplasy and linkage disequilibrium in the Staphylococcus aureus core genome. Whole-genome LD plots are illustrated in 10kb windows. Each 10kb window is displayed as in Figure 3, with a single reference genome, MRSA252. Genes are color-coded by COG category or grey if unclassified. An extended coldspot can be seen between 1448-1458kb. [file ncomms4956-s2.zip › EverittSupplementaryDataset1/0220-0230.LD.png]

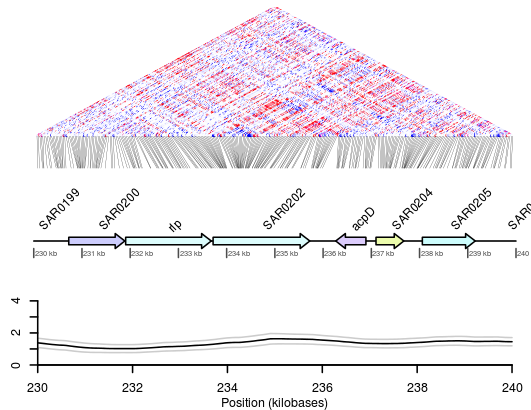

Supplement: Supplementary Data 1 — Homoplasy and linkage disequilibrium in the Staphylococcus aureus core genome. Whole-genome LD plots are illustrated in 10kb windows. Each 10kb window is displayed as in Figure 3, with a single reference genome, MRSA252. Genes are color-coded by COG category or grey if unclassified. An extended coldspot can be seen between 1448-1458kb. [file ncomms4956-s2.zip › EverittSupplementaryDataset1/0230-0240.LD.png]

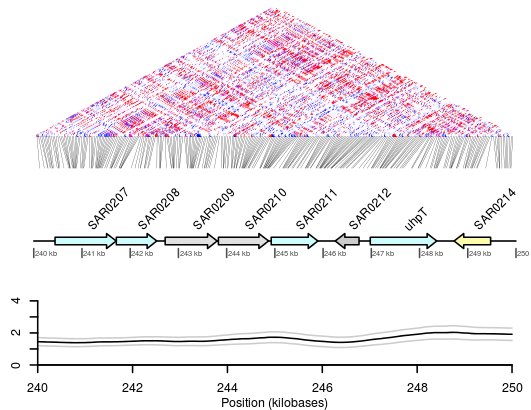

Supplement: Supplementary Data 1 — Homoplasy and linkage disequilibrium in the Staphylococcus aureus core genome. Whole-genome LD plots are illustrated in 10kb windows. Each 10kb window is displayed as in Figure 3, with a single reference genome, MRSA252. Genes are color-coded by COG category or grey if unclassified. An extended coldspot can be seen between 1448-1458kb. [file ncomms4956-s2.zip › EverittSupplementaryDataset1/0240-0250.LD.png]

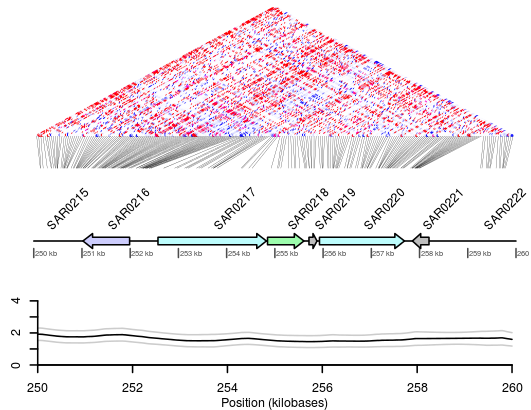

Supplement: Supplementary Data 1 — Homoplasy and linkage disequilibrium in the Staphylococcus aureus core genome. Whole-genome LD plots are illustrated in 10kb windows. Each 10kb window is displayed as in Figure 3, with a single reference genome, MRSA252. Genes are color-coded by COG category or grey if unclassified. An extended coldspot can be seen between 1448-1458kb. [file ncomms4956-s2.zip › EverittSupplementaryDataset1/0250-0260.LD.png]

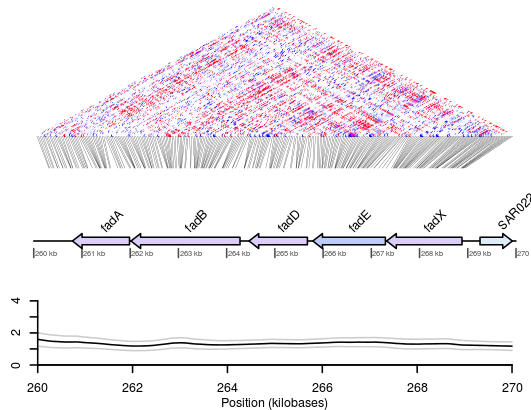

Supplement: Supplementary Data 1 — Homoplasy and linkage disequilibrium in the Staphylococcus aureus core genome. Whole-genome LD plots are illustrated in 10kb windows. Each 10kb window is displayed as in Figure 3, with a single reference genome, MRSA252. Genes are color-coded by COG category or grey if unclassified. An extended coldspot can be seen between 1448-1458kb. [file ncomms4956-s2.zip › EverittSupplementaryDataset1/0260-0270.LD.png]

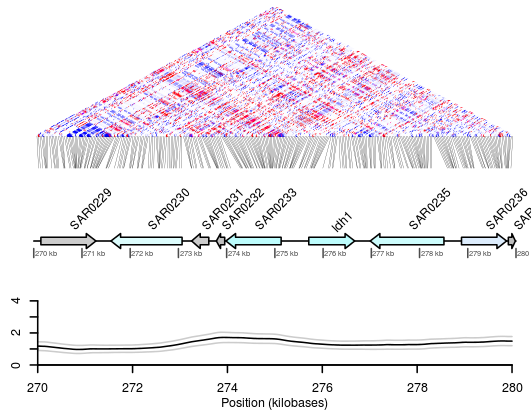

Supplement: Supplementary Data 1 — Homoplasy and linkage disequilibrium in the Staphylococcus aureus core genome. Whole-genome LD plots are illustrated in 10kb windows. Each 10kb window is displayed as in Figure 3, with a single reference genome, MRSA252. Genes are color-coded by COG category or grey if unclassified. An extended coldspot can be seen between 1448-1458kb. [file ncomms4956-s2.zip › EverittSupplementaryDataset1/0270-0280.LD.png]

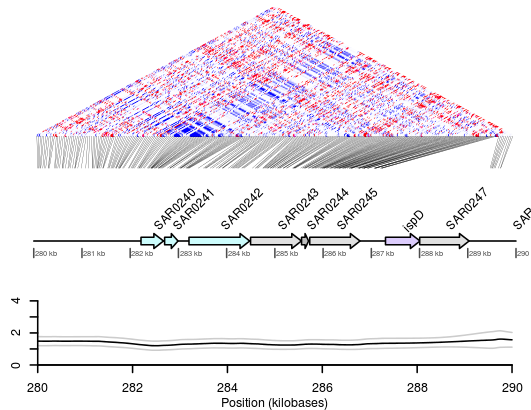

Supplement: Supplementary Data 1 — Homoplasy and linkage disequilibrium in the Staphylococcus aureus core genome. Whole-genome LD plots are illustrated in 10kb windows. Each 10kb window is displayed as in Figure 3, with a single reference genome, MRSA252. Genes are color-coded by COG category or grey if unclassified. An extended coldspot can be seen between 1448-1458kb. [file ncomms4956-s2.zip › EverittSupplementaryDataset1/0280-0290.LD.png]

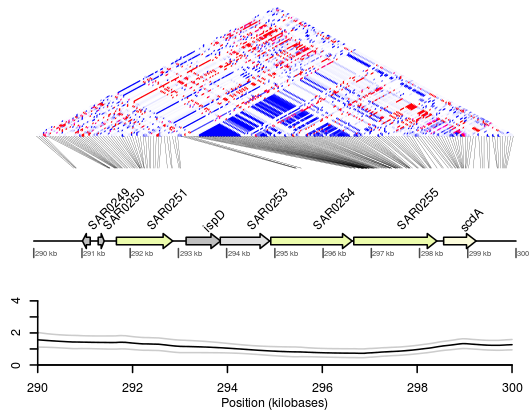

Supplement: Supplementary Data 1 — Homoplasy and linkage disequilibrium in the Staphylococcus aureus core genome. Whole-genome LD plots are illustrated in 10kb windows. Each 10kb window is displayed as in Figure 3, with a single reference genome, MRSA252. Genes are color-coded by COG category or grey if unclassified. An extended coldspot can be seen between 1448-1458kb. [file ncomms4956-s2.zip › EverittSupplementaryDataset1/0290-0300.LD.png]

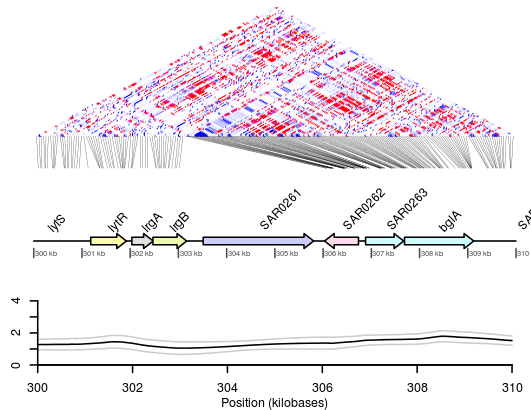

Supplement: Supplementary Data 1 — Homoplasy and linkage disequilibrium in the Staphylococcus aureus core genome. Whole-genome LD plots are illustrated in 10kb windows. Each 10kb window is displayed as in Figure 3, with a single reference genome, MRSA252. Genes are color-coded by COG category or grey if unclassified. An extended coldspot can be seen between 1448-1458kb. [file ncomms4956-s2.zip › EverittSupplementaryDataset1/0300-0310.LD.png]

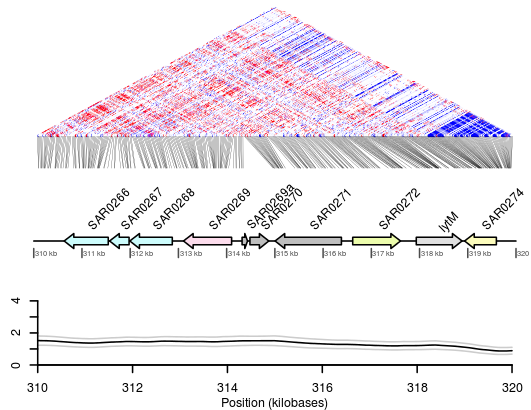

Supplement: Supplementary Data 1 — Homoplasy and linkage disequilibrium in the Staphylococcus aureus core genome. Whole-genome LD plots are illustrated in 10kb windows. Each 10kb window is displayed as in Figure 3, with a single reference genome, MRSA252. Genes are color-coded by COG category or grey if unclassified. An extended coldspot can be seen between 1448-1458kb. [file ncomms4956-s2.zip › EverittSupplementaryDataset1/0310-0320.LD.png]

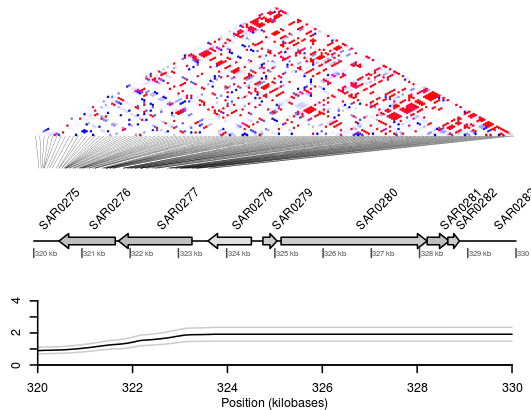

Supplement: Supplementary Data 1 — Homoplasy and linkage disequilibrium in the Staphylococcus aureus core genome. Whole-genome LD plots are illustrated in 10kb windows. Each 10kb window is displayed as in Figure 3, with a single reference genome, MRSA252. Genes are color-coded by COG category or grey if unclassified. An extended coldspot can be seen between 1448-1458kb. [file ncomms4956-s2.zip › EverittSupplementaryDataset1/0320-0330.LD.png]

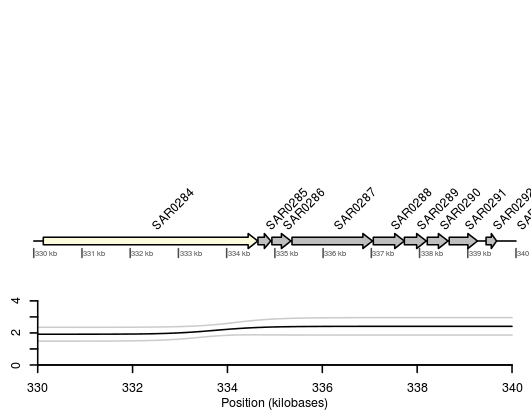

Supplement: Supplementary Data 1 — Homoplasy and linkage disequilibrium in the Staphylococcus aureus core genome. Whole-genome LD plots are illustrated in 10kb windows. Each 10kb window is displayed as in Figure 3, with a single reference genome, MRSA252. Genes are color-coded by COG category or grey if unclassified. An extended coldspot can be seen between 1448-1458kb. [file ncomms4956-s2.zip › EverittSupplementaryDataset1/0330-0340.LD.png]

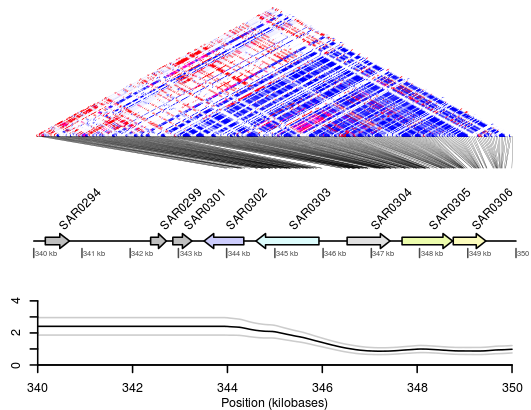

Supplement: Supplementary Data 1 — Homoplasy and linkage disequilibrium in the Staphylococcus aureus core genome. Whole-genome LD plots are illustrated in 10kb windows. Each 10kb window is displayed as in Figure 3, with a single reference genome, MRSA252. Genes are color-coded by COG category or grey if unclassified. An extended coldspot can be seen between 1448-1458kb. [file ncomms4956-s2.zip › EverittSupplementaryDataset1/0340-0350.LD.png]

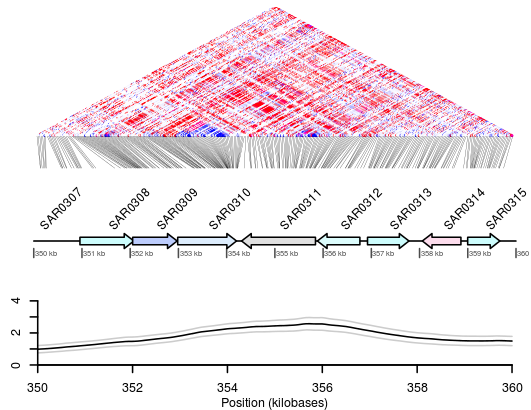

Supplement: Supplementary Data 1 — Homoplasy and linkage disequilibrium in the Staphylococcus aureus core genome. Whole-genome LD plots are illustrated in 10kb windows. Each 10kb window is displayed as in Figure 3, with a single reference genome, MRSA252. Genes are color-coded by COG category or grey if unclassified. An extended coldspot can be seen between 1448-1458kb. [file ncomms4956-s2.zip › EverittSupplementaryDataset1/0350-0360.LD.png]

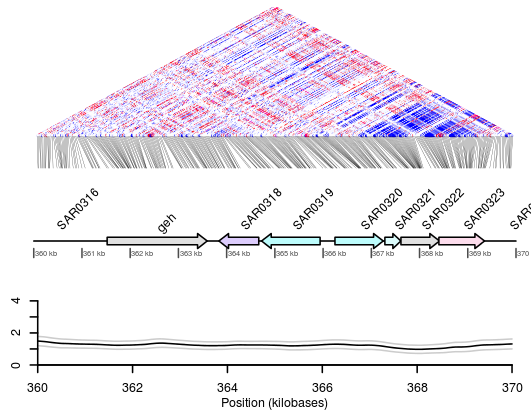

Supplement: Supplementary Data 1 — Homoplasy and linkage disequilibrium in the Staphylococcus aureus core genome. Whole-genome LD plots are illustrated in 10kb windows. Each 10kb window is displayed as in Figure 3, with a single reference genome, MRSA252. Genes are color-coded by COG category or grey if unclassified. An extended coldspot can be seen between 1448-1458kb. [file ncomms4956-s2.zip › EverittSupplementaryDataset1/0360-0370.LD.png]

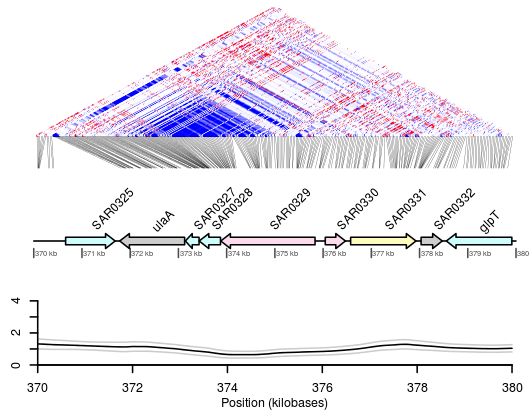

Supplement: Supplementary Data 1 — Homoplasy and linkage disequilibrium in the Staphylococcus aureus core genome. Whole-genome LD plots are illustrated in 10kb windows. Each 10kb window is displayed as in Figure 3, with a single reference genome, MRSA252. Genes are color-coded by COG category or grey if unclassified. An extended coldspot can be seen between 1448-1458kb. [file ncomms4956-s2.zip › EverittSupplementaryDataset1/0370-0380.LD.png]

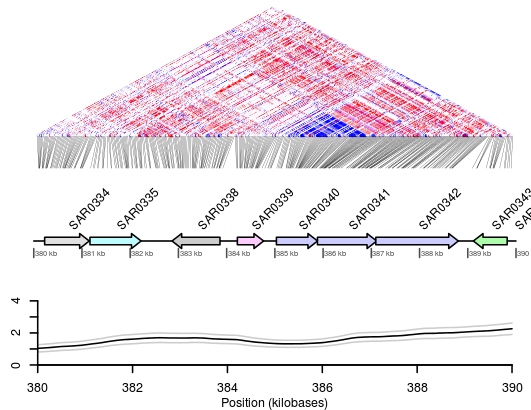

Supplement: Supplementary Data 1 — Homoplasy and linkage disequilibrium in the Staphylococcus aureus core genome. Whole-genome LD plots are illustrated in 10kb windows. Each 10kb window is displayed as in Figure 3, with a single reference genome, MRSA252. Genes are color-coded by COG category or grey if unclassified. An extended coldspot can be seen between 1448-1458kb. [file ncomms4956-s2.zip › EverittSupplementaryDataset1/0380-0390.LD.png]

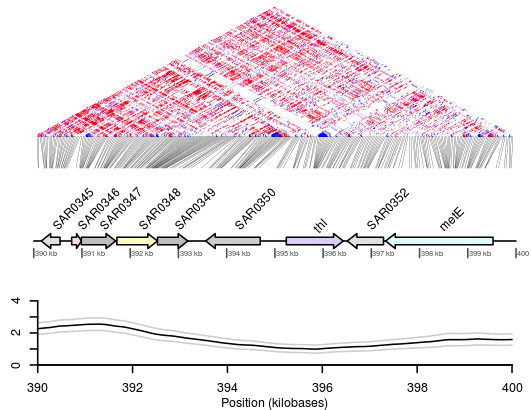

Supplement: Supplementary Data 1 — Homoplasy and linkage disequilibrium in the Staphylococcus aureus core genome. Whole-genome LD plots are illustrated in 10kb windows. Each 10kb window is displayed as in Figure 3, with a single reference genome, MRSA252. Genes are color-coded by COG category or grey if unclassified. An extended coldspot can be seen between 1448-1458kb. [file ncomms4956-s2.zip › EverittSupplementaryDataset1/0390-0400.LD.png]

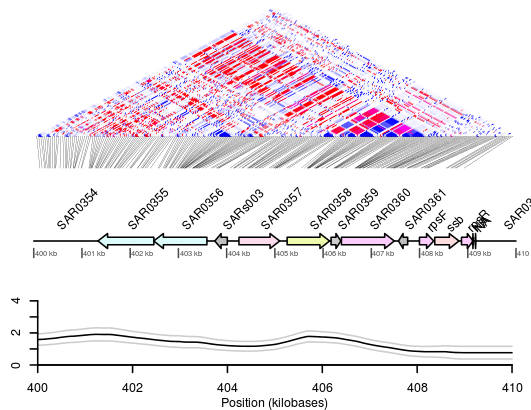

Supplement: Supplementary Data 1 — Homoplasy and linkage disequilibrium in the Staphylococcus aureus core genome. Whole-genome LD plots are illustrated in 10kb windows. Each 10kb window is displayed as in Figure 3, with a single reference genome, MRSA252. Genes are color-coded by COG category or grey if unclassified. An extended coldspot can be seen between 1448-1458kb. [file ncomms4956-s2.zip › EverittSupplementaryDataset1/0400-0410.LD.png]

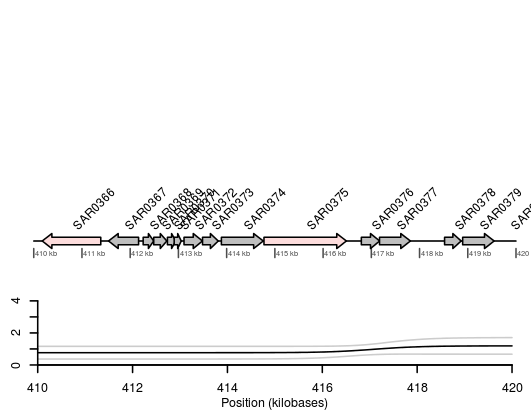

Supplement: Supplementary Data 1 — Homoplasy and linkage disequilibrium in the Staphylococcus aureus core genome. Whole-genome LD plots are illustrated in 10kb windows. Each 10kb window is displayed as in Figure 3, with a single reference genome, MRSA252. Genes are color-coded by COG category or grey if unclassified. An extended coldspot can be seen between 1448-1458kb. [file ncomms4956-s2.zip › EverittSupplementaryDataset1/0410-0420.LD.png]

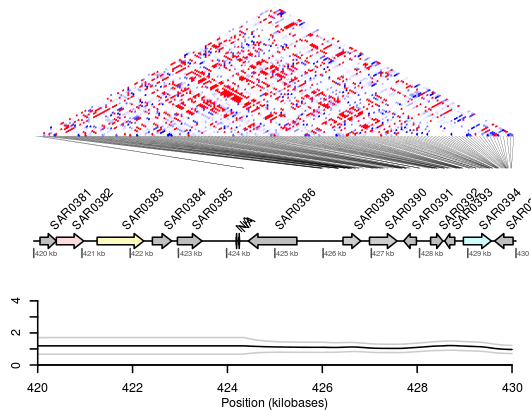

Supplement: Supplementary Data 1 — Homoplasy and linkage disequilibrium in the Staphylococcus aureus core genome. Whole-genome LD plots are illustrated in 10kb windows. Each 10kb window is displayed as in Figure 3, with a single reference genome, MRSA252. Genes are color-coded by COG category or grey if unclassified. An extended coldspot can be seen between 1448-1458kb. [file ncomms4956-s2.zip › EverittSupplementaryDataset1/0420-0430.LD.png]

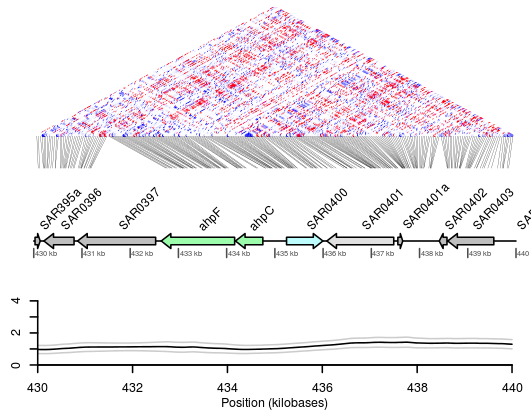

Supplement: Supplementary Data 1 — Homoplasy and linkage disequilibrium in the Staphylococcus aureus core genome. Whole-genome LD plots are illustrated in 10kb windows. Each 10kb window is displayed as in Figure 3, with a single reference genome, MRSA252. Genes are color-coded by COG category or grey if unclassified. An extended coldspot can be seen between 1448-1458kb. [file ncomms4956-s2.zip › EverittSupplementaryDataset1/0430-0440.LD.png]

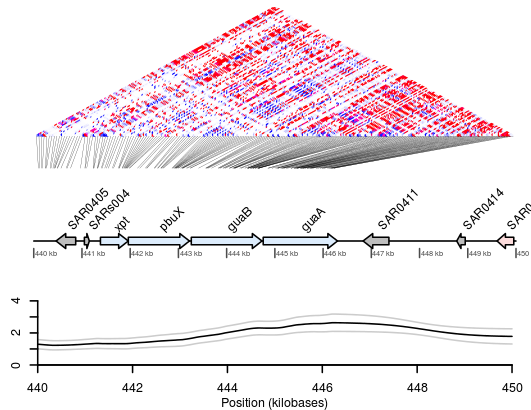

Supplement: Supplementary Data 1 — Homoplasy and linkage disequilibrium in the Staphylococcus aureus core genome. Whole-genome LD plots are illustrated in 10kb windows. Each 10kb window is displayed as in Figure 3, with a single reference genome, MRSA252. Genes are color-coded by COG category or grey if unclassified. An extended coldspot can be seen between 1448-1458kb. [file ncomms4956-s2.zip › EverittSupplementaryDataset1/0440-0450.LD.png]

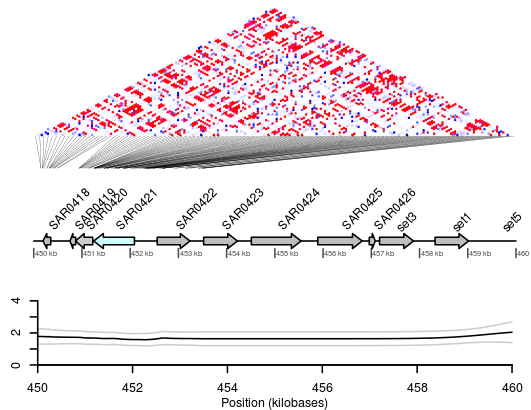

Supplement: Supplementary Data 1 — Homoplasy and linkage disequilibrium in the Staphylococcus aureus core genome. Whole-genome LD plots are illustrated in 10kb windows. Each 10kb window is displayed as in Figure 3, with a single reference genome, MRSA252. Genes are color-coded by COG category or grey if unclassified. An extended coldspot can be seen between 1448-1458kb. [file ncomms4956-s2.zip › EverittSupplementaryDataset1/0450-0460.LD.png]

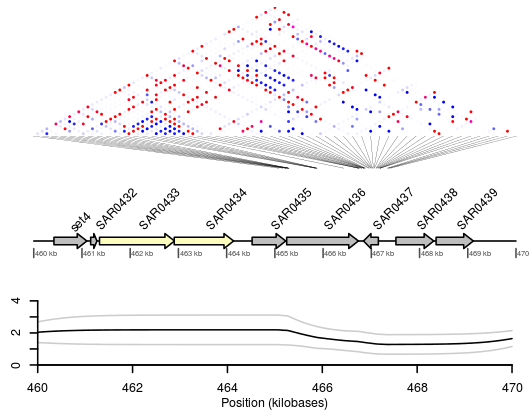

Supplement: Supplementary Data 1 — Homoplasy and linkage disequilibrium in the Staphylococcus aureus core genome. Whole-genome LD plots are illustrated in 10kb windows. Each 10kb window is displayed as in Figure 3, with a single reference genome, MRSA252. Genes are color-coded by COG category or grey if unclassified. An extended coldspot can be seen between 1448-1458kb. [file ncomms4956-s2.zip › EverittSupplementaryDataset1/0460-0470.LD.png]

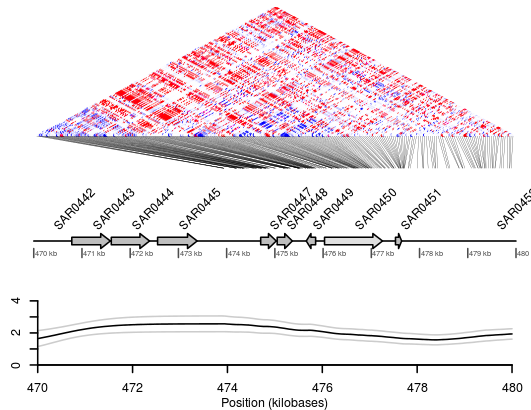

Supplement: Supplementary Data 1 — Homoplasy and linkage disequilibrium in the Staphylococcus aureus core genome. Whole-genome LD plots are illustrated in 10kb windows. Each 10kb window is displayed as in Figure 3, with a single reference genome, MRSA252. Genes are color-coded by COG category or grey if unclassified. An extended coldspot can be seen between 1448-1458kb. [file ncomms4956-s2.zip › EverittSupplementaryDataset1/0470-0480.LD.png]

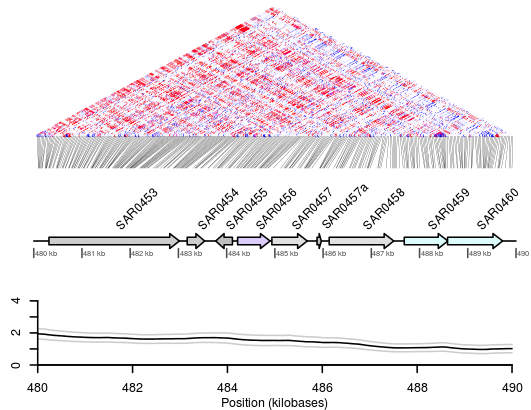

Supplement: Supplementary Data 1 — Homoplasy and linkage disequilibrium in the Staphylococcus aureus core genome. Whole-genome LD plots are illustrated in 10kb windows. Each 10kb window is displayed as in Figure 3, with a single reference genome, MRSA252. Genes are color-coded by COG category or grey if unclassified. An extended coldspot can be seen between 1448-1458kb. [file ncomms4956-s2.zip › EverittSupplementaryDataset1/0480-0490.LD.png]

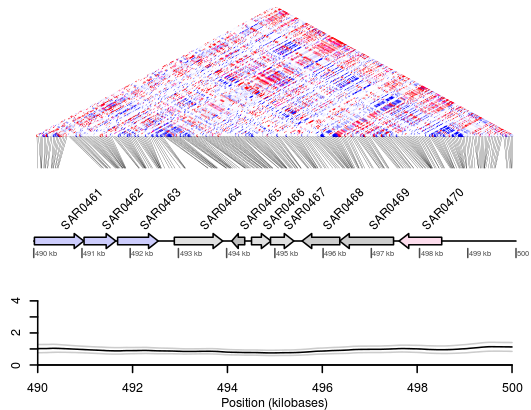

Supplement: Supplementary Data 1 — Homoplasy and linkage disequilibrium in the Staphylococcus aureus core genome. Whole-genome LD plots are illustrated in 10kb windows. Each 10kb window is displayed as in Figure 3, with a single reference genome, MRSA252. Genes are color-coded by COG category or grey if unclassified. An extended coldspot can be seen between 1448-1458kb. [file ncomms4956-s2.zip › EverittSupplementaryDataset1/0490-0500.LD.png]

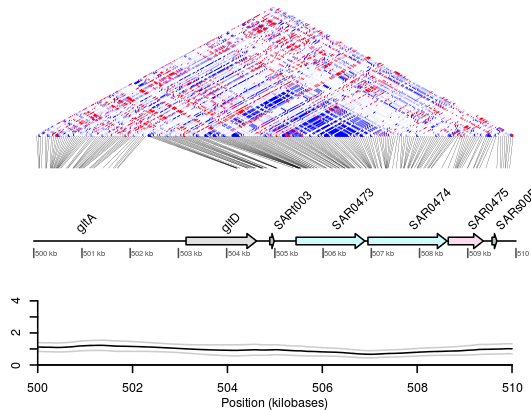

Supplement: Supplementary Data 1 — Homoplasy and linkage disequilibrium in the Staphylococcus aureus core genome. Whole-genome LD plots are illustrated in 10kb windows. Each 10kb window is displayed as in Figure 3, with a single reference genome, MRSA252. Genes are color-coded by COG category or grey if unclassified. An extended coldspot can be seen between 1448-1458kb. [file ncomms4956-s2.zip › EverittSupplementaryDataset1/0500-0510.LD.png]

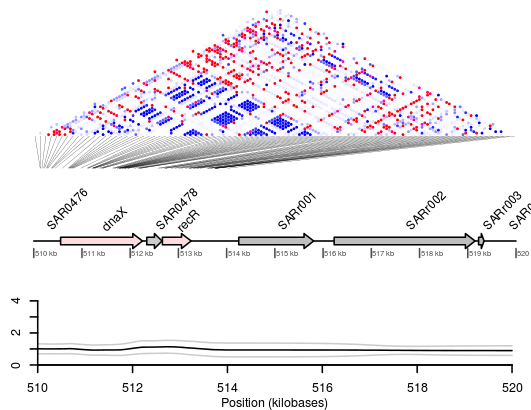

Supplement: Supplementary Data 1 — Homoplasy and linkage disequilibrium in the Staphylococcus aureus core genome. Whole-genome LD plots are illustrated in 10kb windows. Each 10kb window is displayed as in Figure 3, with a single reference genome, MRSA252. Genes are color-coded by COG category or grey if unclassified. An extended coldspot can be seen between 1448-1458kb. [file ncomms4956-s2.zip › EverittSupplementaryDataset1/0510-0520.LD.png]

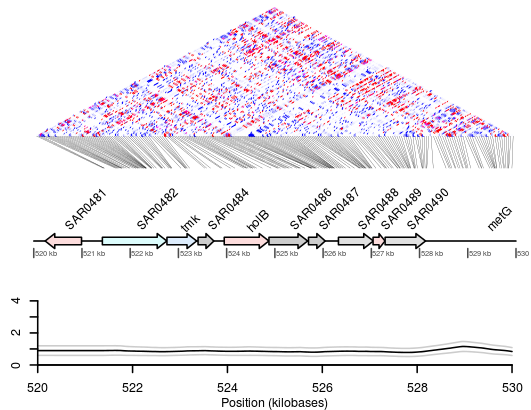

Supplement: Supplementary Data 1 — Homoplasy and linkage disequilibrium in the Staphylococcus aureus core genome. Whole-genome LD plots are illustrated in 10kb windows. Each 10kb window is displayed as in Figure 3, with a single reference genome, MRSA252. Genes are color-coded by COG category or grey if unclassified. An extended coldspot can be seen between 1448-1458kb. [file ncomms4956-s2.zip › EverittSupplementaryDataset1/0520-0530.LD.png]

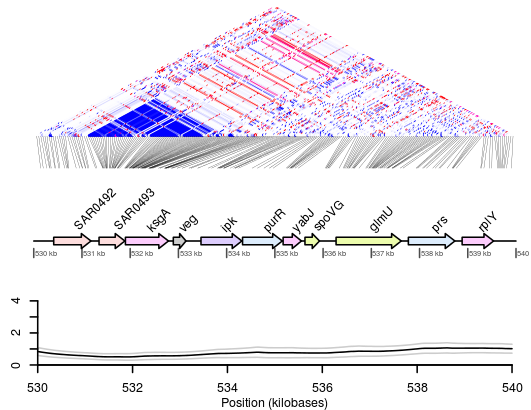

Supplement: Supplementary Data 1 — Homoplasy and linkage disequilibrium in the Staphylococcus aureus core genome. Whole-genome LD plots are illustrated in 10kb windows. Each 10kb window is displayed as in Figure 3, with a single reference genome, MRSA252. Genes are color-coded by COG category or grey if unclassified. An extended coldspot can be seen between 1448-1458kb. [file ncomms4956-s2.zip › EverittSupplementaryDataset1/0530-0540.LD.png]

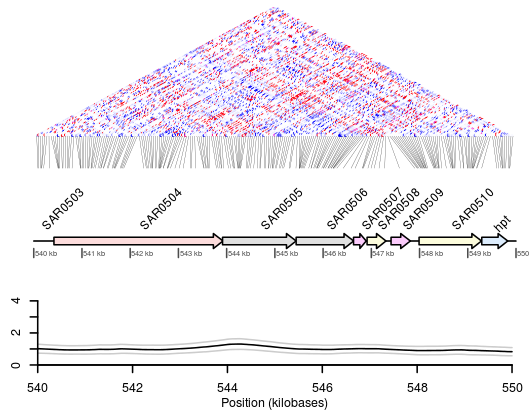

Supplement: Supplementary Data 1 — Homoplasy and linkage disequilibrium in the Staphylococcus aureus core genome. Whole-genome LD plots are illustrated in 10kb windows. Each 10kb window is displayed as in Figure 3, with a single reference genome, MRSA252. Genes are color-coded by COG category or grey if unclassified. An extended coldspot can be seen between 1448-1458kb. [file ncomms4956-s2.zip › EverittSupplementaryDataset1/0540-0550.LD.png]

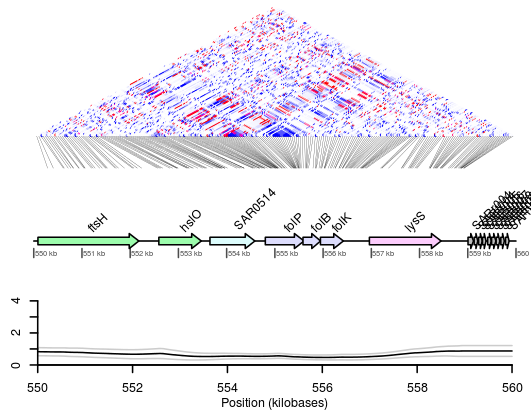

Supplement: Supplementary Data 1 — Homoplasy and linkage disequilibrium in the Staphylococcus aureus core genome. Whole-genome LD plots are illustrated in 10kb windows. Each 10kb window is displayed as in Figure 3, with a single reference genome, MRSA252. Genes are color-coded by COG category or grey if unclassified. An extended coldspot can be seen between 1448-1458kb. [file ncomms4956-s2.zip › EverittSupplementaryDataset1/0550-0560.LD.png]

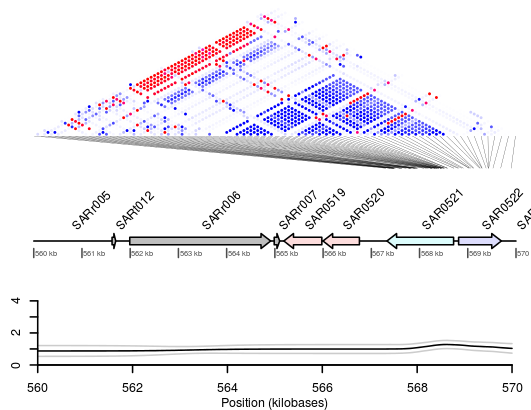

Supplement: Supplementary Data 1 — Homoplasy and linkage disequilibrium in the Staphylococcus aureus core genome. Whole-genome LD plots are illustrated in 10kb windows. Each 10kb window is displayed as in Figure 3, with a single reference genome, MRSA252. Genes are color-coded by COG category or grey if unclassified. An extended coldspot can be seen between 1448-1458kb. [file ncomms4956-s2.zip › EverittSupplementaryDataset1/0560-0570.LD.png]

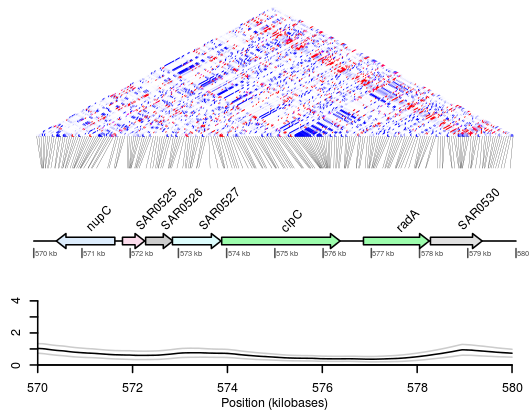

Supplement: Supplementary Data 1 — Homoplasy and linkage disequilibrium in the Staphylococcus aureus core genome. Whole-genome LD plots are illustrated in 10kb windows. Each 10kb window is displayed as in Figure 3, with a single reference genome, MRSA252. Genes are color-coded by COG category or grey if unclassified. An extended coldspot can be seen between 1448-1458kb. [file ncomms4956-s2.zip › EverittSupplementaryDataset1/0570-0580.LD.png]

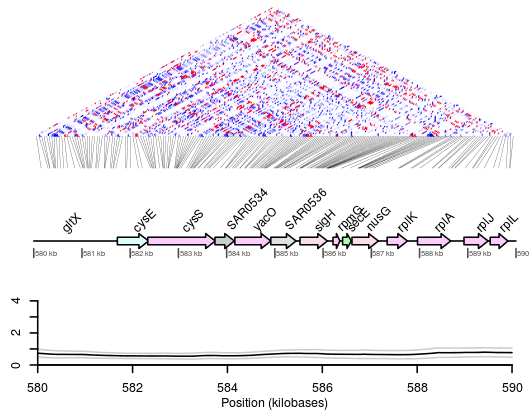

Supplement: Supplementary Data 1 — Homoplasy and linkage disequilibrium in the Staphylococcus aureus core genome. Whole-genome LD plots are illustrated in 10kb windows. Each 10kb window is displayed as in Figure 3, with a single reference genome, MRSA252. Genes are color-coded by COG category or grey if unclassified. An extended coldspot can be seen between 1448-1458kb. [file ncomms4956-s2.zip › EverittSupplementaryDataset1/0580-0590.LD.png]

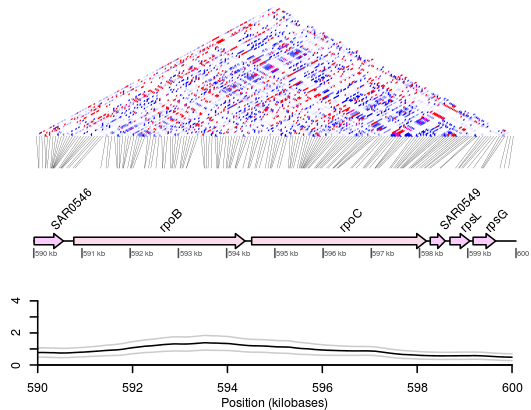

Supplement: Supplementary Data 1 — Homoplasy and linkage disequilibrium in the Staphylococcus aureus core genome. Whole-genome LD plots are illustrated in 10kb windows. Each 10kb window is displayed as in Figure 3, with a single reference genome, MRSA252. Genes are color-coded by COG category or grey if unclassified. An extended coldspot can be seen between 1448-1458kb. [file ncomms4956-s2.zip › EverittSupplementaryDataset1/0590-0600.LD.png]

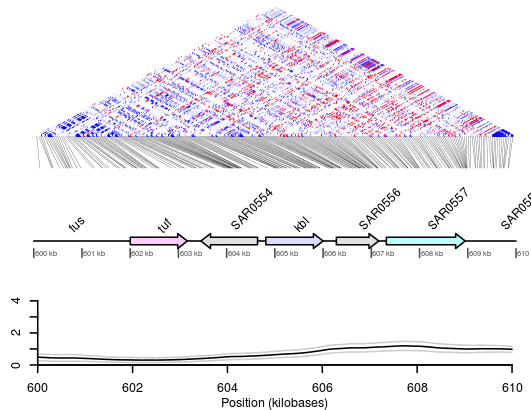

Supplement: Supplementary Data 1 — Homoplasy and linkage disequilibrium in the Staphylococcus aureus core genome. Whole-genome LD plots are illustrated in 10kb windows. Each 10kb window is displayed as in Figure 3, with a single reference genome, MRSA252. Genes are color-coded by COG category or grey if unclassified. An extended coldspot can be seen between 1448-1458kb. [file ncomms4956-s2.zip › EverittSupplementaryDataset1/0600-0610.LD.png]

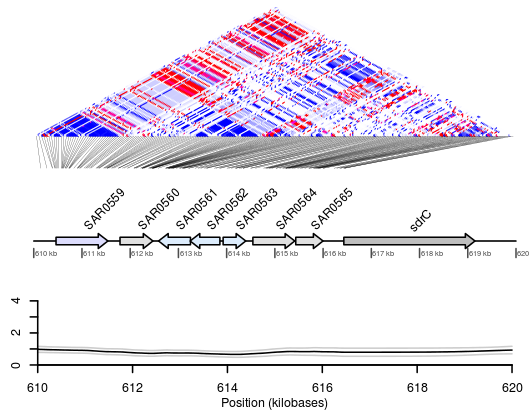

Supplement: Supplementary Data 1 — Homoplasy and linkage disequilibrium in the Staphylococcus aureus core genome. Whole-genome LD plots are illustrated in 10kb windows. Each 10kb window is displayed as in Figure 3, with a single reference genome, MRSA252. Genes are color-coded by COG category or grey if unclassified. An extended coldspot can be seen between 1448-1458kb. [file ncomms4956-s2.zip › EverittSupplementaryDataset1/0610-0620.LD.png]

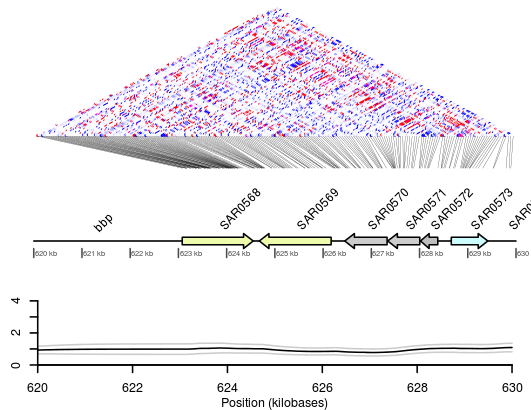

Supplement: Supplementary Data 1 — Homoplasy and linkage disequilibrium in the Staphylococcus aureus core genome. Whole-genome LD plots are illustrated in 10kb windows. Each 10kb window is displayed as in Figure 3, with a single reference genome, MRSA252. Genes are color-coded by COG category or grey if unclassified. An extended coldspot can be seen between 1448-1458kb. [file ncomms4956-s2.zip › EverittSupplementaryDataset1/0620-0630.LD.png]

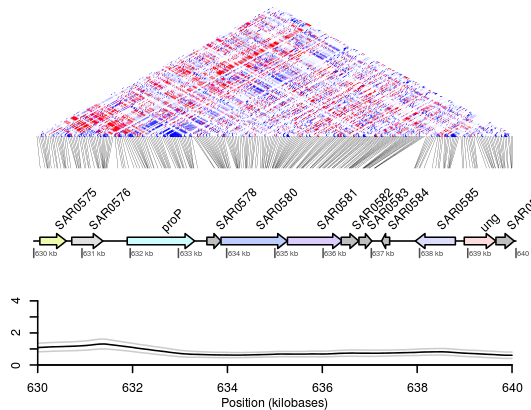

Supplement: Supplementary Data 1 — Homoplasy and linkage disequilibrium in the Staphylococcus aureus core genome. Whole-genome LD plots are illustrated in 10kb windows. Each 10kb window is displayed as in Figure 3, with a single reference genome, MRSA252. Genes are color-coded by COG category or grey if unclassified. An extended coldspot can be seen between 1448-1458kb. [file ncomms4956-s2.zip › EverittSupplementaryDataset1/0630-0640.LD.png]

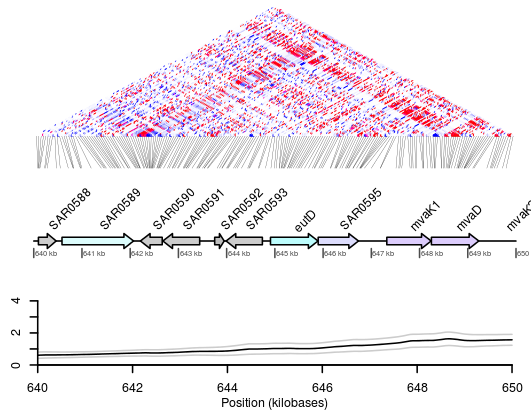

Supplement: Supplementary Data 1 — Homoplasy and linkage disequilibrium in the Staphylococcus aureus core genome. Whole-genome LD plots are illustrated in 10kb windows. Each 10kb window is displayed as in Figure 3, with a single reference genome, MRSA252. Genes are color-coded by COG category or grey if unclassified. An extended coldspot can be seen between 1448-1458kb. [file ncomms4956-s2.zip › EverittSupplementaryDataset1/0640-0650.LD.png]

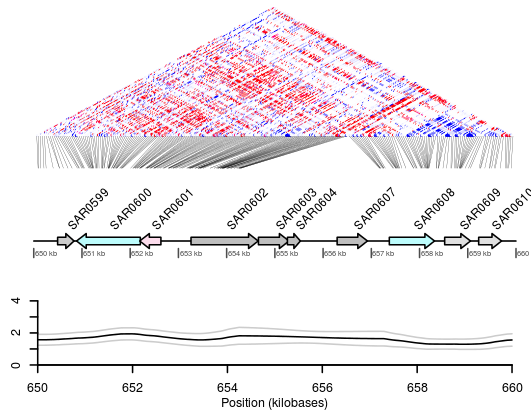

Supplement: Supplementary Data 1 — Homoplasy and linkage disequilibrium in the Staphylococcus aureus core genome. Whole-genome LD plots are illustrated in 10kb windows. Each 10kb window is displayed as in Figure 3, with a single reference genome, MRSA252. Genes are color-coded by COG category or grey if unclassified. An extended coldspot can be seen between 1448-1458kb. [file ncomms4956-s2.zip › EverittSupplementaryDataset1/0650-0660.LD.png]

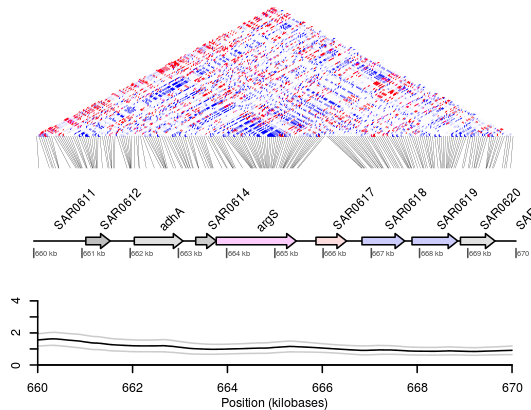

Supplement: Supplementary Data 1 — Homoplasy and linkage disequilibrium in the Staphylococcus aureus core genome. Whole-genome LD plots are illustrated in 10kb windows. Each 10kb window is displayed as in Figure 3, with a single reference genome, MRSA252. Genes are color-coded by COG category or grey if unclassified. An extended coldspot can be seen between 1448-1458kb. [file ncomms4956-s2.zip › EverittSupplementaryDataset1/0660-0670.LD.png]

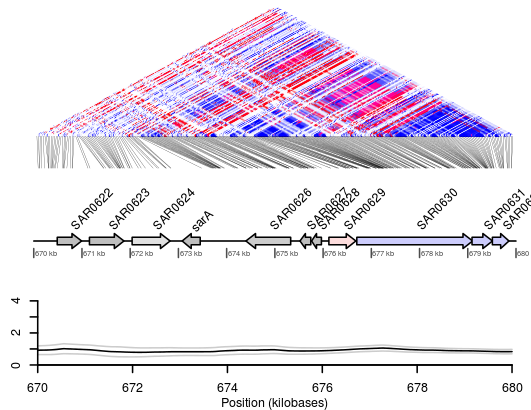

Supplement: Supplementary Data 1 — Homoplasy and linkage disequilibrium in the Staphylococcus aureus core genome. Whole-genome LD plots are illustrated in 10kb windows. Each 10kb window is displayed as in Figure 3, with a single reference genome, MRSA252. Genes are color-coded by COG category or grey if unclassified. An extended coldspot can be seen between 1448-1458kb. [file ncomms4956-s2.zip › EverittSupplementaryDataset1/0670-0680.LD.png]

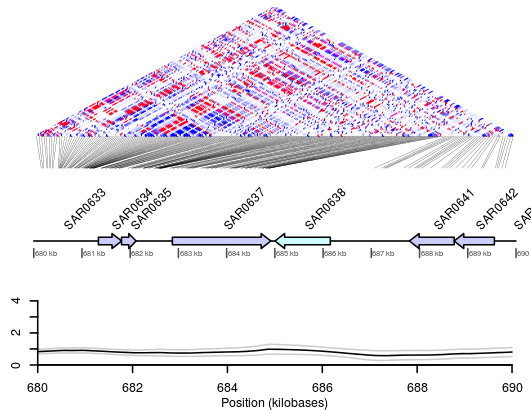

Supplement: Supplementary Data 1 — Homoplasy and linkage disequilibrium in the Staphylococcus aureus core genome. Whole-genome LD plots are illustrated in 10kb windows. Each 10kb window is displayed as in Figure 3, with a single reference genome, MRSA252. Genes are color-coded by COG category or grey if unclassified. An extended coldspot can be seen between 1448-1458kb. [file ncomms4956-s2.zip › EverittSupplementaryDataset1/0680-0690.LD.png]

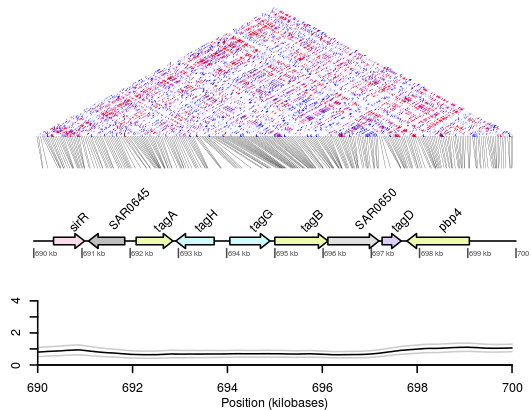

Supplement: Supplementary Data 1 — Homoplasy and linkage disequilibrium in the Staphylococcus aureus core genome. Whole-genome LD plots are illustrated in 10kb windows. Each 10kb window is displayed as in Figure 3, with a single reference genome, MRSA252. Genes are color-coded by COG category or grey if unclassified. An extended coldspot can be seen between 1448-1458kb. [file ncomms4956-s2.zip › EverittSupplementaryDataset1/0690-0700.LD.png]

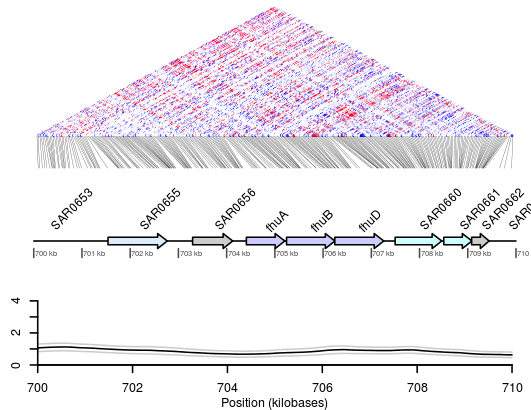

Supplement: Supplementary Data 1 — Homoplasy and linkage disequilibrium in the Staphylococcus aureus core genome. Whole-genome LD plots are illustrated in 10kb windows. Each 10kb window is displayed as in Figure 3, with a single reference genome, MRSA252. Genes are color-coded by COG category or grey if unclassified. An extended coldspot can be seen between 1448-1458kb. [file ncomms4956-s2.zip › EverittSupplementaryDataset1/0700-0710.LD.png]

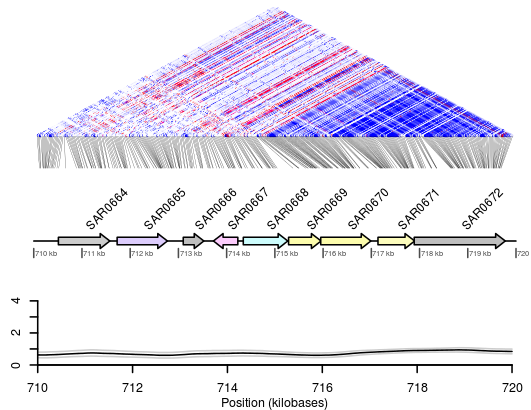

Supplement: Supplementary Data 1 — Homoplasy and linkage disequilibrium in the Staphylococcus aureus core genome. Whole-genome LD plots are illustrated in 10kb windows. Each 10kb window is displayed as in Figure 3, with a single reference genome, MRSA252. Genes are color-coded by COG category or grey if unclassified. An extended coldspot can be seen between 1448-1458kb. [file ncomms4956-s2.zip › EverittSupplementaryDataset1/0710-0720.LD.png]

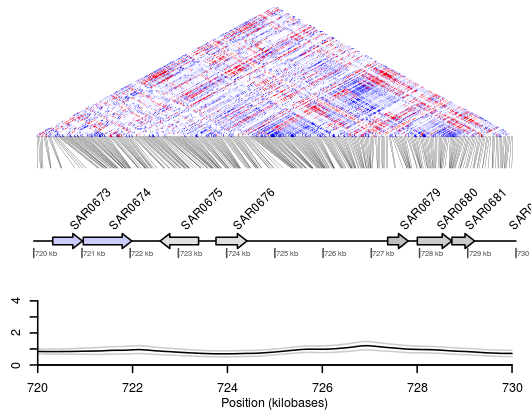

Supplement: Supplementary Data 1 — Homoplasy and linkage disequilibrium in the Staphylococcus aureus core genome. Whole-genome LD plots are illustrated in 10kb windows. Each 10kb window is displayed as in Figure 3, with a single reference genome, MRSA252. Genes are color-coded by COG category or grey if unclassified. An extended coldspot can be seen between 1448-1458kb. [file ncomms4956-s2.zip › EverittSupplementaryDataset1/0720-0730.LD.png]

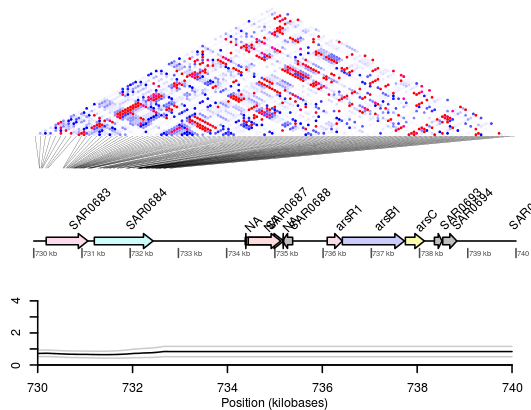

Supplement: Supplementary Data 1 — Homoplasy and linkage disequilibrium in the Staphylococcus aureus core genome. Whole-genome LD plots are illustrated in 10kb windows. Each 10kb window is displayed as in Figure 3, with a single reference genome, MRSA252. Genes are color-coded by COG category or grey if unclassified. An extended coldspot can be seen between 1448-1458kb. [file ncomms4956-s2.zip › EverittSupplementaryDataset1/0730-0740.LD.png]

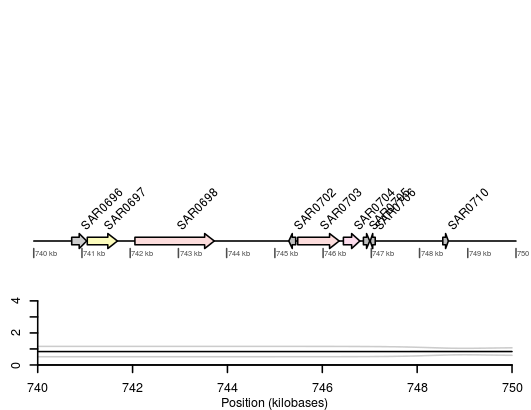

Supplement: Supplementary Data 1 — Homoplasy and linkage disequilibrium in the Staphylococcus aureus core genome. Whole-genome LD plots are illustrated in 10kb windows. Each 10kb window is displayed as in Figure 3, with a single reference genome, MRSA252. Genes are color-coded by COG category or grey if unclassified. An extended coldspot can be seen between 1448-1458kb. [file ncomms4956-s2.zip › EverittSupplementaryDataset1/0740-0750.LD.png]

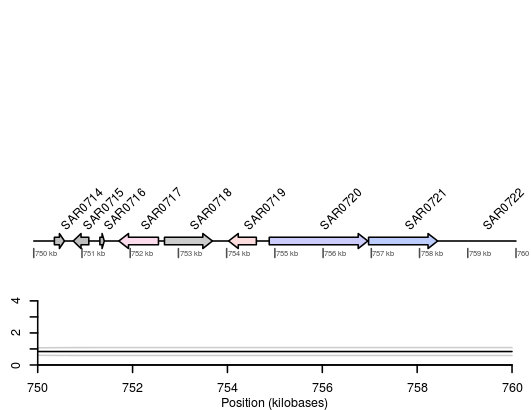

Supplement: Supplementary Data 1 — Homoplasy and linkage disequilibrium in the Staphylococcus aureus core genome. Whole-genome LD plots are illustrated in 10kb windows. Each 10kb window is displayed as in Figure 3, with a single reference genome, MRSA252. Genes are color-coded by COG category or grey if unclassified. An extended coldspot can be seen between 1448-1458kb. [file ncomms4956-s2.zip › EverittSupplementaryDataset1/0750-0760.LD.png]

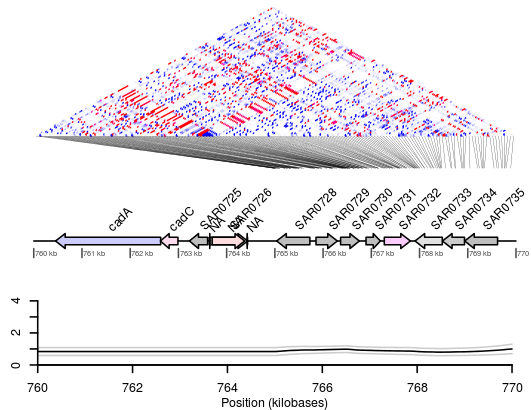

Supplement: Supplementary Data 1 — Homoplasy and linkage disequilibrium in the Staphylococcus aureus core genome. Whole-genome LD plots are illustrated in 10kb windows. Each 10kb window is displayed as in Figure 3, with a single reference genome, MRSA252. Genes are color-coded by COG category or grey if unclassified. An extended coldspot can be seen between 1448-1458kb. [file ncomms4956-s2.zip › EverittSupplementaryDataset1/0760-0770.LD.png]

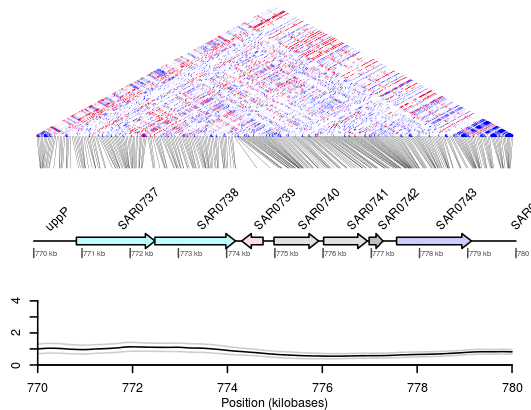

Supplement: Supplementary Data 1 — Homoplasy and linkage disequilibrium in the Staphylococcus aureus core genome. Whole-genome LD plots are illustrated in 10kb windows. Each 10kb window is displayed as in Figure 3, with a single reference genome, MRSA252. Genes are color-coded by COG category or grey if unclassified. An extended coldspot can be seen between 1448-1458kb. [file ncomms4956-s2.zip › EverittSupplementaryDataset1/0770-0780.LD.png]

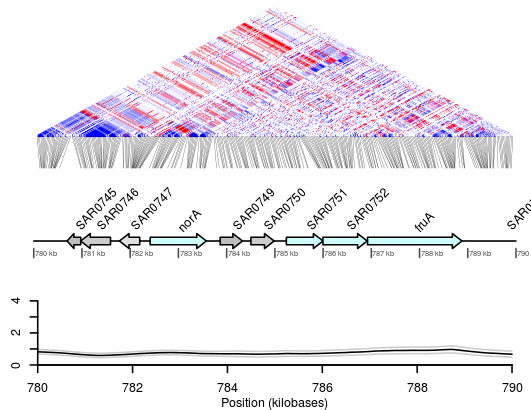

Supplement: Supplementary Data 1 — Homoplasy and linkage disequilibrium in the Staphylococcus aureus core genome. Whole-genome LD plots are illustrated in 10kb windows. Each 10kb window is displayed as in Figure 3, with a single reference genome, MRSA252. Genes are color-coded by COG category or grey if unclassified. An extended coldspot can be seen between 1448-1458kb. [file ncomms4956-s2.zip › EverittSupplementaryDataset1/0780-0790.LD.png]

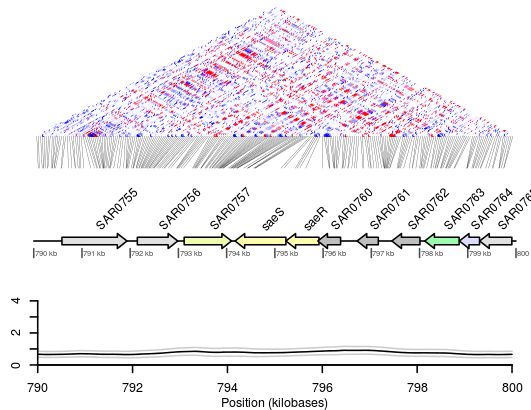

Supplement: Supplementary Data 1 — Homoplasy and linkage disequilibrium in the Staphylococcus aureus core genome. Whole-genome LD plots are illustrated in 10kb windows. Each 10kb window is displayed as in Figure 3, with a single reference genome, MRSA252. Genes are color-coded by COG category or grey if unclassified. An extended coldspot can be seen between 1448-1458kb. [file ncomms4956-s2.zip › EverittSupplementaryDataset1/0790-0800.LD.png]

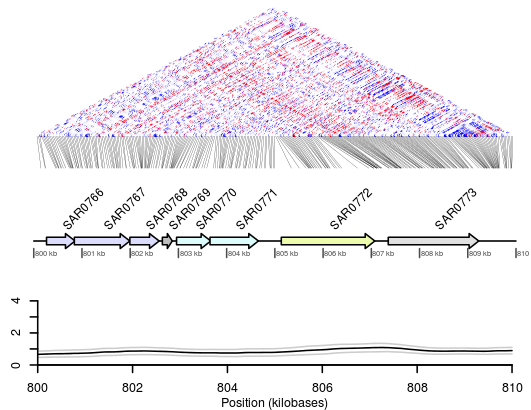

Supplement: Supplementary Data 1 — Homoplasy and linkage disequilibrium in the Staphylococcus aureus core genome. Whole-genome LD plots are illustrated in 10kb windows. Each 10kb window is displayed as in Figure 3, with a single reference genome, MRSA252. Genes are color-coded by COG category or grey if unclassified. An extended coldspot can be seen between 1448-1458kb. [file ncomms4956-s2.zip › EverittSupplementaryDataset1/0800-0810.LD.png]

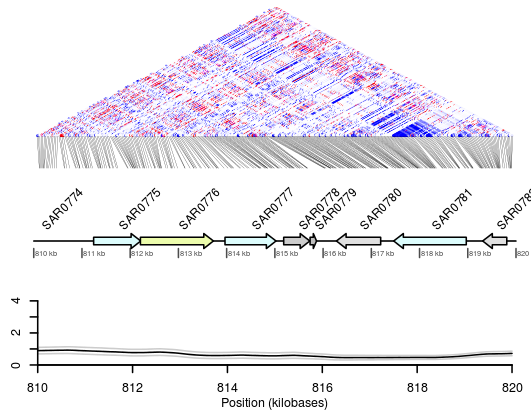

Supplement: Supplementary Data 1 — Homoplasy and linkage disequilibrium in the Staphylococcus aureus core genome. Whole-genome LD plots are illustrated in 10kb windows. Each 10kb window is displayed as in Figure 3, with a single reference genome, MRSA252. Genes are color-coded by COG category or grey if unclassified. An extended coldspot can be seen between 1448-1458kb. [file ncomms4956-s2.zip › EverittSupplementaryDataset1/0810-0820.LD.png]

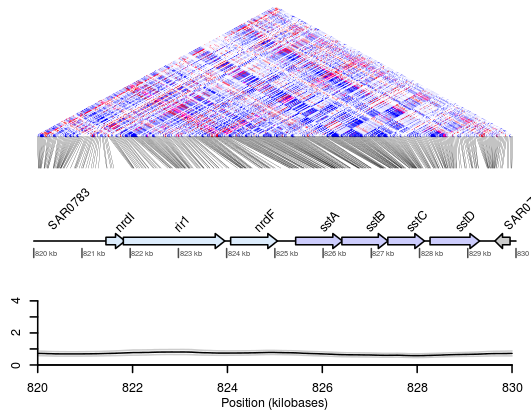

Supplement: Supplementary Data 1 — Homoplasy and linkage disequilibrium in the Staphylococcus aureus core genome. Whole-genome LD plots are illustrated in 10kb windows. Each 10kb window is displayed as in Figure 3, with a single reference genome, MRSA252. Genes are color-coded by COG category or grey if unclassified. An extended coldspot can be seen between 1448-1458kb. [file ncomms4956-s2.zip › EverittSupplementaryDataset1/0820-0830.LD.png]

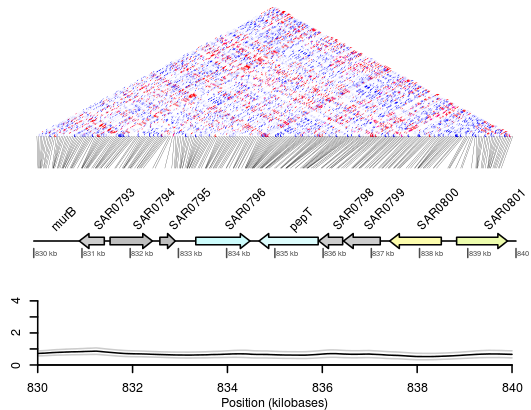

Supplement: Supplementary Data 1 — Homoplasy and linkage disequilibrium in the Staphylococcus aureus core genome. Whole-genome LD plots are illustrated in 10kb windows. Each 10kb window is displayed as in Figure 3, with a single reference genome, MRSA252. Genes are color-coded by COG category or grey if unclassified. An extended coldspot can be seen between 1448-1458kb. [file ncomms4956-s2.zip › EverittSupplementaryDataset1/0830-0840.LD.png]

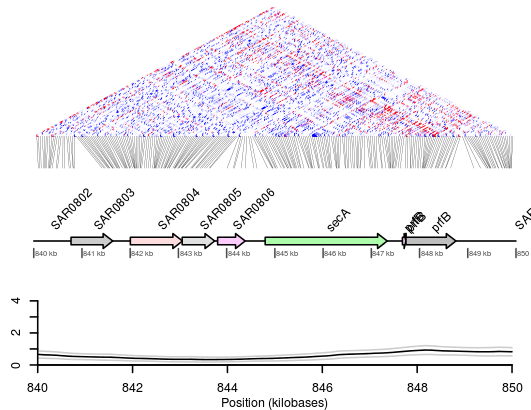

Supplement: Supplementary Data 1 — Homoplasy and linkage disequilibrium in the Staphylococcus aureus core genome. Whole-genome LD plots are illustrated in 10kb windows. Each 10kb window is displayed as in Figure 3, with a single reference genome, MRSA252. Genes are color-coded by COG category or grey if unclassified. An extended coldspot can be seen between 1448-1458kb. [file ncomms4956-s2.zip › EverittSupplementaryDataset1/0840-0850.LD.png]

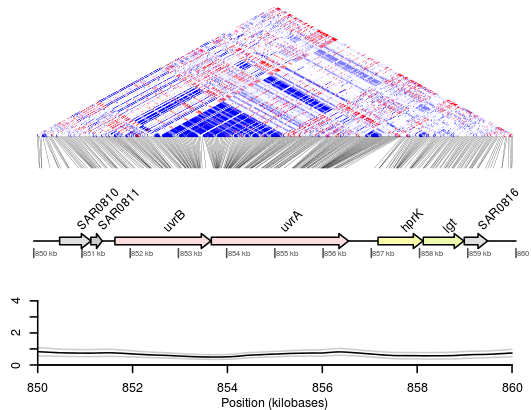

Supplement: Supplementary Data 1 — Homoplasy and linkage disequilibrium in the Staphylococcus aureus core genome. Whole-genome LD plots are illustrated in 10kb windows. Each 10kb window is displayed as in Figure 3, with a single reference genome, MRSA252. Genes are color-coded by COG category or grey if unclassified. An extended coldspot can be seen between 1448-1458kb. [file ncomms4956-s2.zip › EverittSupplementaryDataset1/0850-0860.LD.png]

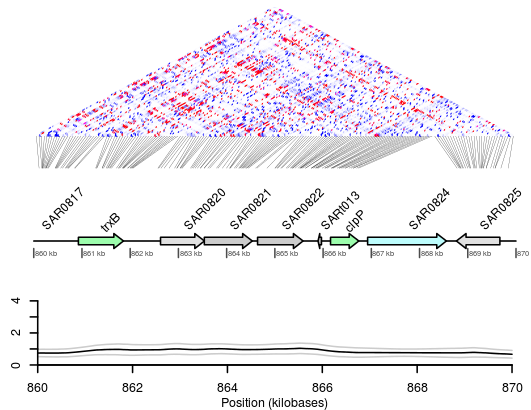

Supplement: Supplementary Data 1 — Homoplasy and linkage disequilibrium in the Staphylococcus aureus core genome. Whole-genome LD plots are illustrated in 10kb windows. Each 10kb window is displayed as in Figure 3, with a single reference genome, MRSA252. Genes are color-coded by COG category or grey if unclassified. An extended coldspot can be seen between 1448-1458kb. [file ncomms4956-s2.zip › EverittSupplementaryDataset1/0860-0870.LD.png]

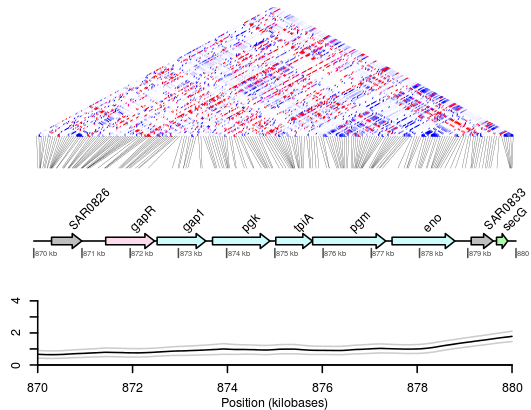

Supplement: Supplementary Data 1 — Homoplasy and linkage disequilibrium in the Staphylococcus aureus core genome. Whole-genome LD plots are illustrated in 10kb windows. Each 10kb window is displayed as in Figure 3, with a single reference genome, MRSA252. Genes are color-coded by COG category or grey if unclassified. An extended coldspot can be seen between 1448-1458kb. [file ncomms4956-s2.zip › EverittSupplementaryDataset1/0870-0880.LD.png]

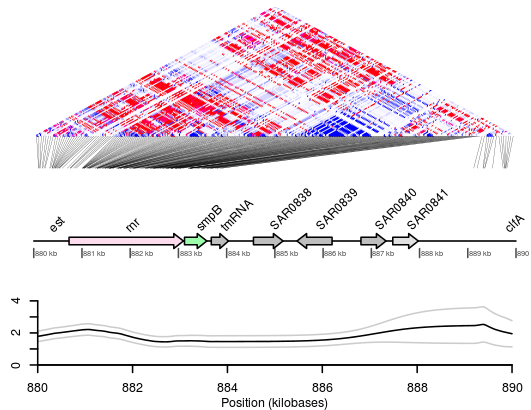

Supplement: Supplementary Data 1 — Homoplasy and linkage disequilibrium in the Staphylococcus aureus core genome. Whole-genome LD plots are illustrated in 10kb windows. Each 10kb window is displayed as in Figure 3, with a single reference genome, MRSA252. Genes are color-coded by COG category or grey if unclassified. An extended coldspot can be seen between 1448-1458kb. [file ncomms4956-s2.zip › EverittSupplementaryDataset1/0880-0890.LD.png]

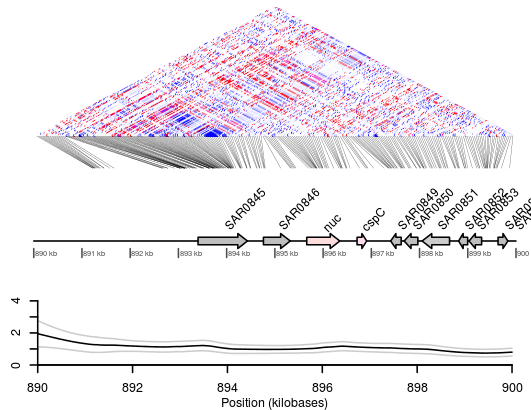

Supplement: Supplementary Data 1 — Homoplasy and linkage disequilibrium in the Staphylococcus aureus core genome. Whole-genome LD plots are illustrated in 10kb windows. Each 10kb window is displayed as in Figure 3, with a single reference genome, MRSA252. Genes are color-coded by COG category or grey if unclassified. An extended coldspot can be seen between 1448-1458kb. [file ncomms4956-s2.zip › EverittSupplementaryDataset1/0890-0900.LD.png]

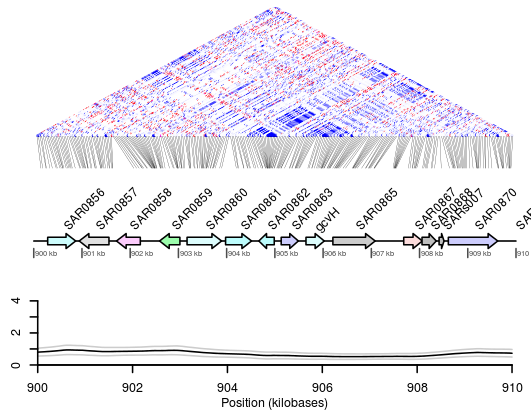

Supplement: Supplementary Data 1 — Homoplasy and linkage disequilibrium in the Staphylococcus aureus core genome. Whole-genome LD plots are illustrated in 10kb windows. Each 10kb window is displayed as in Figure 3, with a single reference genome, MRSA252. Genes are color-coded by COG category or grey if unclassified. An extended coldspot can be seen between 1448-1458kb. [file ncomms4956-s2.zip › EverittSupplementaryDataset1/0900-0910.LD.png]

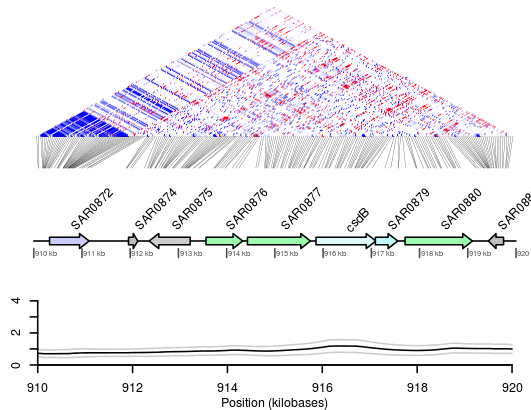

Supplement: Supplementary Data 1 — Homoplasy and linkage disequilibrium in the Staphylococcus aureus core genome. Whole-genome LD plots are illustrated in 10kb windows. Each 10kb window is displayed as in Figure 3, with a single reference genome, MRSA252. Genes are color-coded by COG category or grey if unclassified. An extended coldspot can be seen between 1448-1458kb. [file ncomms4956-s2.zip › EverittSupplementaryDataset1/0910-0920.LD.png]

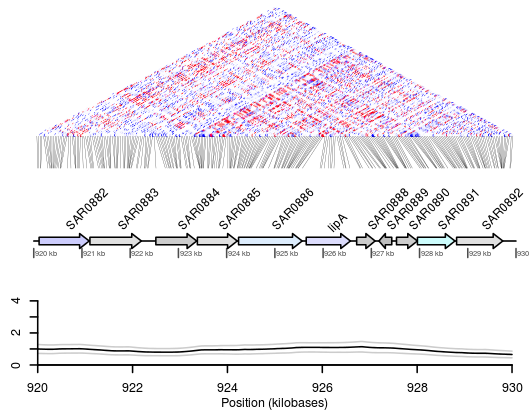

Supplement: Supplementary Data 1 — Homoplasy and linkage disequilibrium in the Staphylococcus aureus core genome. Whole-genome LD plots are illustrated in 10kb windows. Each 10kb window is displayed as in Figure 3, with a single reference genome, MRSA252. Genes are color-coded by COG category or grey if unclassified. An extended coldspot can be seen between 1448-1458kb. [file ncomms4956-s2.zip › EverittSupplementaryDataset1/0920-0930.LD.png]

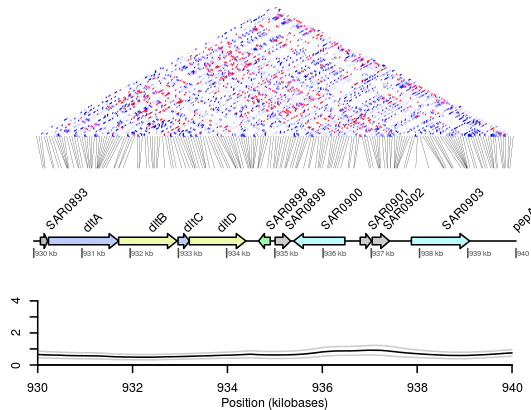

Supplement: Supplementary Data 1 — Homoplasy and linkage disequilibrium in the Staphylococcus aureus core genome. Whole-genome LD plots are illustrated in 10kb windows. Each 10kb window is displayed as in Figure 3, with a single reference genome, MRSA252. Genes are color-coded by COG category or grey if unclassified. An extended coldspot can be seen between 1448-1458kb. [file ncomms4956-s2.zip › EverittSupplementaryDataset1/0930-0940.LD.png]

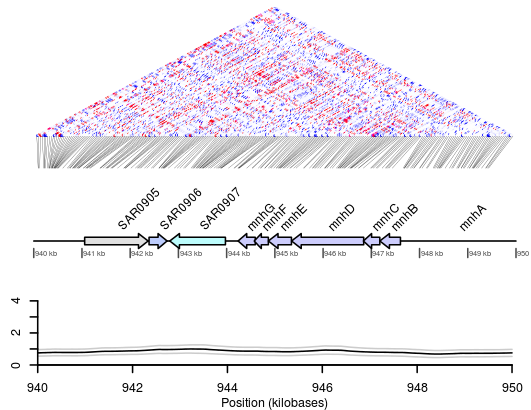

Supplement: Supplementary Data 1 — Homoplasy and linkage disequilibrium in the Staphylococcus aureus core genome. Whole-genome LD plots are illustrated in 10kb windows. Each 10kb window is displayed as in Figure 3, with a single reference genome, MRSA252. Genes are color-coded by COG category or grey if unclassified. An extended coldspot can be seen between 1448-1458kb. [file ncomms4956-s2.zip › EverittSupplementaryDataset1/0940-0950.LD.png]

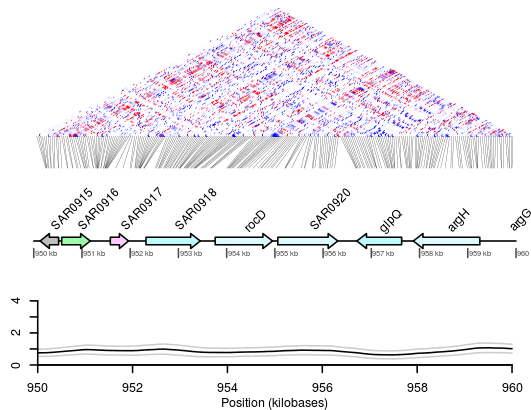

Supplement: Supplementary Data 1 — Homoplasy and linkage disequilibrium in the Staphylococcus aureus core genome. Whole-genome LD plots are illustrated in 10kb windows. Each 10kb window is displayed as in Figure 3, with a single reference genome, MRSA252. Genes are color-coded by COG category or grey if unclassified. An extended coldspot can be seen between 1448-1458kb. [file ncomms4956-s2.zip › EverittSupplementaryDataset1/0950-0960.LD.png]

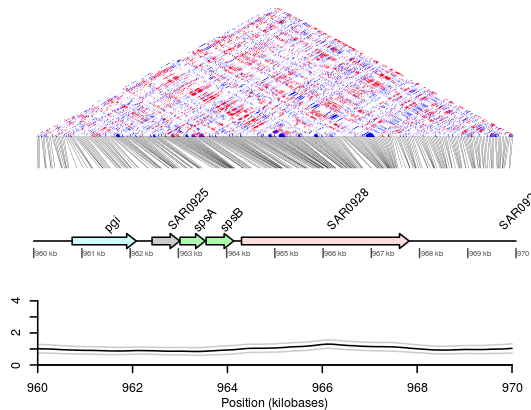

Supplement: Supplementary Data 1 — Homoplasy and linkage disequilibrium in the Staphylococcus aureus core genome. Whole-genome LD plots are illustrated in 10kb windows. Each 10kb window is displayed as in Figure 3, with a single reference genome, MRSA252. Genes are color-coded by COG category or grey if unclassified. An extended coldspot can be seen between 1448-1458kb. [file ncomms4956-s2.zip › EverittSupplementaryDataset1/0960-0970.LD.png]

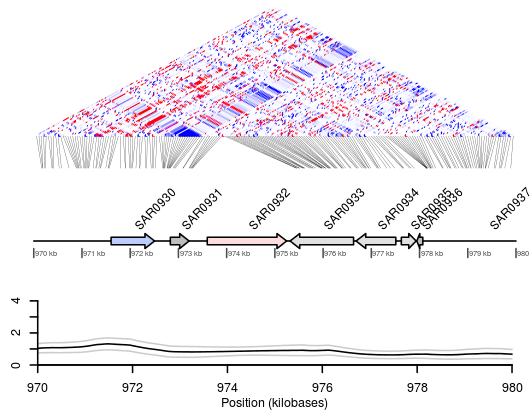

Supplement: Supplementary Data 1 — Homoplasy and linkage disequilibrium in the Staphylococcus aureus core genome. Whole-genome LD plots are illustrated in 10kb windows. Each 10kb window is displayed as in Figure 3, with a single reference genome, MRSA252. Genes are color-coded by COG category or grey if unclassified. An extended coldspot can be seen between 1448-1458kb. [file ncomms4956-s2.zip › EverittSupplementaryDataset1/0970-0980.LD.png]

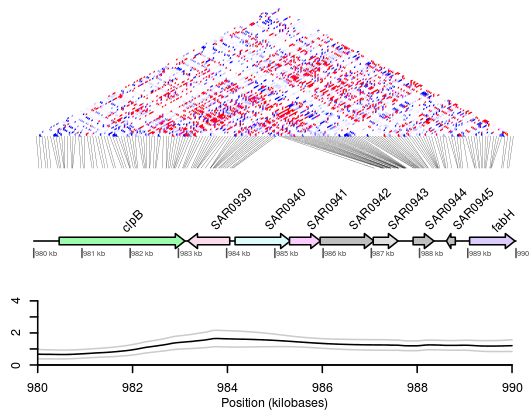

Supplement: Supplementary Data 1 — Homoplasy and linkage disequilibrium in the Staphylococcus aureus core genome. Whole-genome LD plots are illustrated in 10kb windows. Each 10kb window is displayed as in Figure 3, with a single reference genome, MRSA252. Genes are color-coded by COG category or grey if unclassified. An extended coldspot can be seen between 1448-1458kb. [file ncomms4956-s2.zip › EverittSupplementaryDataset1/0980-0990.LD.png]

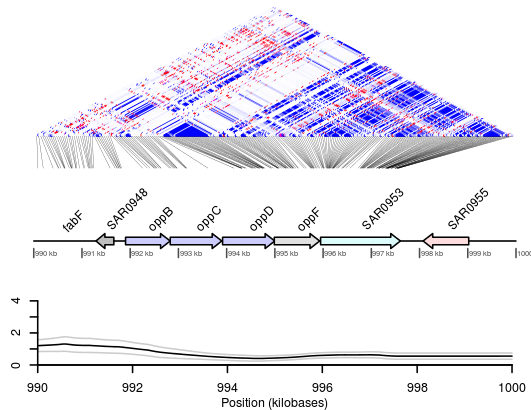

Supplement: Supplementary Data 1 — Homoplasy and linkage disequilibrium in the Staphylococcus aureus core genome. Whole-genome LD plots are illustrated in 10kb windows. Each 10kb window is displayed as in Figure 3, with a single reference genome, MRSA252. Genes are color-coded by COG category or grey if unclassified. An extended coldspot can be seen between 1448-1458kb. [file ncomms4956-s2.zip › EverittSupplementaryDataset1/0990-1000.LD.png]
